# Supplementary material for: Direct synthesis of bicyclo[1.1.1]pentane (BCP) boronates from carboxylic acids
Source: Nat Commun. 2026 Feb 23;17:3070. doi: 10.1038/s41467-026-69851-w (PMC13039371; doi:10.1038/s41467-026-69851-w)
Supplement: Supplementary file 1 — Supplementary Information [file 41467_2026_69851_MOESM1_ESM.pdf]

Direct synthesis of bicyclo[1.1.1]pentane (BCP) boronates from carboxylic acids

Yongchen Wang[1], Jess C. Tang[1], Gang Wu[2], and Julian G. West*[1]

[1]Department of Chemistry, Biosciences Research Collaborative, Rice University; Houston, TX 77030, USA.
[2]Department of Internal Medicine, University of Texas- McGovern Medical School; Houston, TX 77030, USA.

*Corresponding author, jgwest@rice.edu

Supplementary Information

Table of contents

1. Experimental. 3

1.1 General Experimental. 3

2. Optimization of reaction conditions (decarboxylative borylation for BCP boronic ester synthesis). 4

2.1 Optimization of equivalents of iron (Table S1). 4

2.2 Optimization of equivalents of Cs_2CO_3 (Table S2). 5

2.3 Optimization of solvent (Table S3). 6

3. General procedures for the synthesis of BCP boronic esters and characteristic data of corresponding product. 7

3.1 General procedure for the synthesis of BCP boronic esters by synergistic HAT and LMCT. 7

3.2 List of carboxylic acids for decarboxylative borylation reactions. 10

3.3 Characterization of MCR product. 11

3.4. Scalability of the standard reaction. 37

3.5. Mechanism study. 38

4. NMR spectra of novel compounds. 52

5. References. 122

1. Experimental.

1.1 General Experimental.

Regents, solvents and reaction conditions

All reactions were performed in dry glassware under a nitrogen atmosphere unless otherwise stated. Solvents and commercially available reagents were dried and purified before use where appropriate using standard procedures. Dichloromethane and acetonitrile (MeCN) were obtained anhydrous from solvent dispenser units having been passed through an activated alumina column under nitrogen. Anhydrous dimethylacetamide (DMA) and dimethyl sulfoxide (DMSO) were obtained commercially and used directly without further purification. Chloroform-d_1 from sigma-aldrich and Chloroform-d_1 containing 0.05% V/V TMS from Cambridge Isotope Labs Inc were used for obtaining NMR spectra.

Chromatography methods

Reactions were monitored by thin layer chromatography using F_{254} 0.25 mm precoated silica plates purchased from Silicycle, Quebec, Canada. Visualisation was carried out under UV light (λ = 254 nm) or stains such as vanillin and phosphomolybdic acid (PMA). Flash column chromatography was performed on Silicycle P60 silica gel (40-63 µm, 230-400 mesh particle size) as static phase and under increased pressure.

Instrumental analytical methods

NMR spectra were recorded on a Bruker DRX-600 spectrometers (600 MHz for proton nuclei ^{1}H, 151 MHz for carbon nuclei ^{13}C, 192 MHz for ^{11}B and 564 MHz for ^{19}F). The analysis of the NMR spectra was carried out with MestReNova®. Chemical shifts δ are given in parts per million (ppm) to the nearest 0.01 ppm for ^{1}H and 0.1 ppm for ^{13}C, ^{11}B and ^{19}F spectra, with the solvent resonance as internal standard: chloroform-d_1: 7.26 (^{1}H NMR) and 77.2 (^{13}C NMR). Coupling constants J are reported to the nearest 0.1 Hz. Multiplicities are reported as follows: s = singlet, br s = broad singlet, d = doublet, dd = doublet of doublets etc., t = triplet, q = quartet, sept = septet, m = multiplet. Assignments of compounds were based on two-dimensional NMR (COSY, HSQC, HMBC). Note: for ^{13}C NMR, the carbon of BCP direct attached to boron was not able to be detected due to quadrupolar broadening.

High resolution mass spectra (HRMS) were recorded under ESI conditions on a Agilent UHPLC TOF mass spectrometer using electrospray ionization time-of-flight (ESI-TOF), chemical ionization time-of-flight (CI-TOF) or atmospheric pressure chemical ionization (ACPI). Values are calculated to 4 decimal places from the molecular formula. The parent ion $[M+H]^{+}$and $[M+Na]^{+}$ are quoted.

X-band electron paramagnetic resonance (EPR) spectra of DMPO-radical adducts were recorded on a Bruker EMX spectrometer. EPR parameters were: frequency, 9.3 GHz; microwave power, 10 mW; scan range, 80 G; modulation frequency, 100 kHz; modulation amplitude, 1.0 G, time constant, 0.16 s, and receiver gain, 1 x 10^{5} or 7.1 x 10^{4}.

2. Optimization of reaction conditions (decarboxylative borylation for BCP boronic ester synthesis).

2.1 Optimization of equivalents of iron (Table S1).

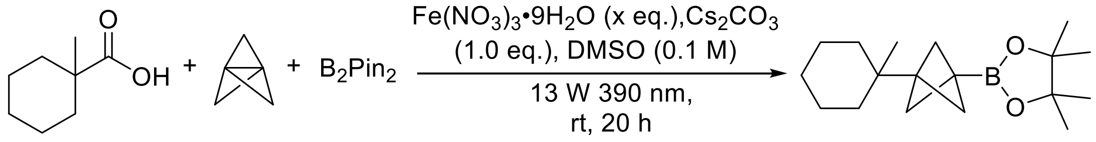

2.0 equiv. 1.0 equiv. 3.0 equiv.

Entry	Equivalence of $Fe(NO_3)_3 \cdot 9H_2O$	NMR yield (%)
1	0	70
2	0.05	89
3	0.1	95
4	0.15	76
5	0.2	52

2.2 Optimization of equivalents of Cs_2CO_3 (Table S2).

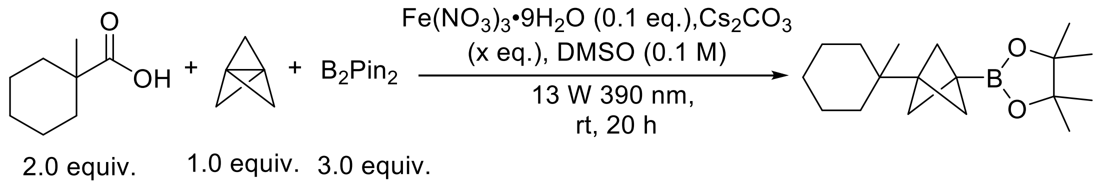

Entry	Equivalence of Cs_2CO_3	NMR yield (%)
1	0	58
2	0.1	82
3	0.2	98
4	0.4	93
5	0.5	93
6	1.0	95

2.3 Optimization of solvent (Table S3).

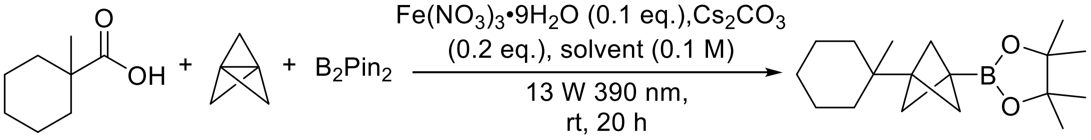

2.0 equiv. 1.0 equiv. 3.0 equiv.

Entry	solvent	NMR yield (%)
1	MeCN	31
2	CH_2Cl_2	8
3	DMA	14
4	DMSO	98

3. General procedures for the synthesis of BCP boronic esters and characteristic data of corresponding product.

3.1 General procedure for the synthesis of BCP boronic esters by synergistic HAT and LMCT.

To a solution of carboxylic acids (0.2 mmol, 2.0 equiv.), bis(pinacolato)diboron (0.3 mmol, 3.0 equiv.), Cs_2CO_3 (0.02 mmol, 0.2 equiv.) and $Fe(NO_3)_3 \cdot 9H_2O$ (0.01 mmol, 0.1 equiv.) in DMSO (1.0 mL) under N_2 atmosphere was added [1.1.1]propellane **1** (0.1 mmol, 1.0 equiv.) The punctured holes of the vial cap were sealed with vacuum grease and electric tape/parafilm for better air-tight protection. The reaction mixture was sonicated for about 60 seconds until it was almost clear. The reaction mixture was then placed under 390 nm Kessil® light (25% intensity, 13 W) with a cooling fan. After stirring at room temperature for 20 h, the reaction mixture was quenched with water (30 mL). The layers were separated and the aqueous layer was extracted with Et_2O (3X5 mL). The combined organic layers were washed with brine (30 mL), dried over Na_2SO_4, filtered and concentrated under reduced pressure (The crude residue was passed through a short silica gel plug prepared in a glass pipette and eluted with CH_2Cl_2 to remove iron salts, affording material suitable for crude NMR analysis when required). The crude residue was purified by flash column chromatography (Et_2O/hexane) to give corresponding BCP boronic esters as white solids or colorless oil.

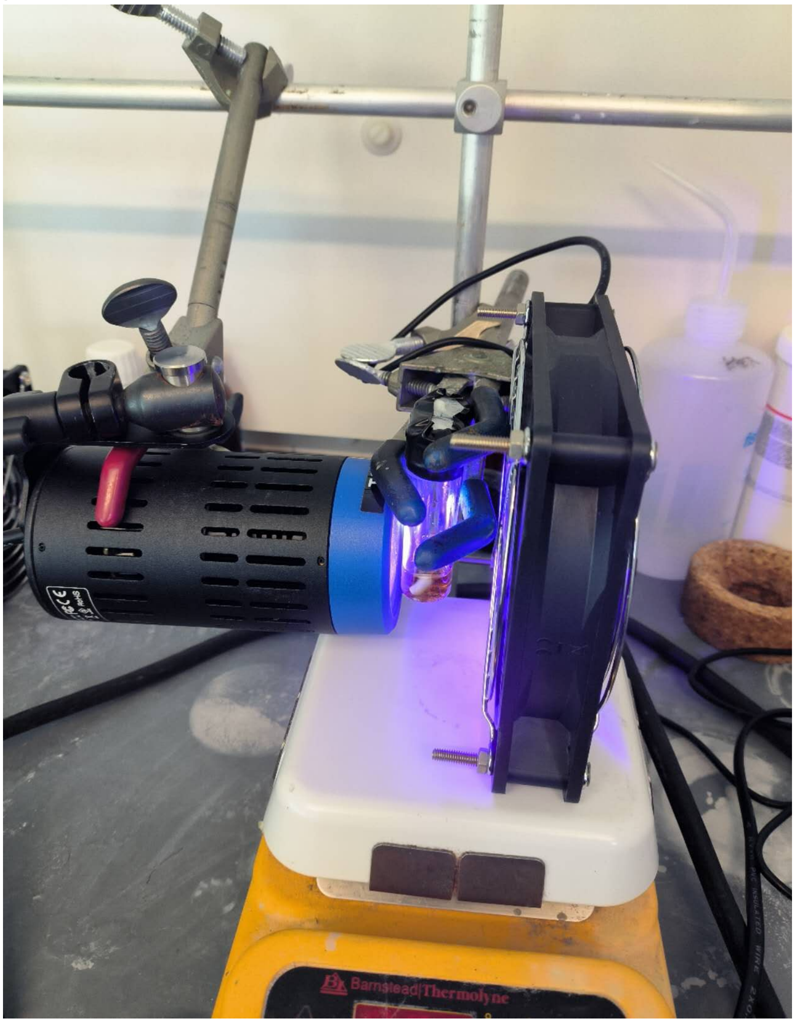

[1.1.1]propellane was prepared according to literature reported procedure[1]

Concentration was determined by ^1H NMR spectroscopy using a sample of stock solution (200 μL), DCE (50 μL) and $CDCl_3$ in NMR tube.

Calculation of concentration of [1.1.1]propellane batch 1:

2.40 (DCE, 4H) : 1.00 ([1.1.1]propellane, 6H)

(2.40/4) : (1.00/6) = 0.6 : 0.167

(50 μL x 1.253 g/mL)/98.96 g/mol = 0.63 mmol

(0.63 mmol/(0.6/0.167))/0.2 mL = 0.88 M

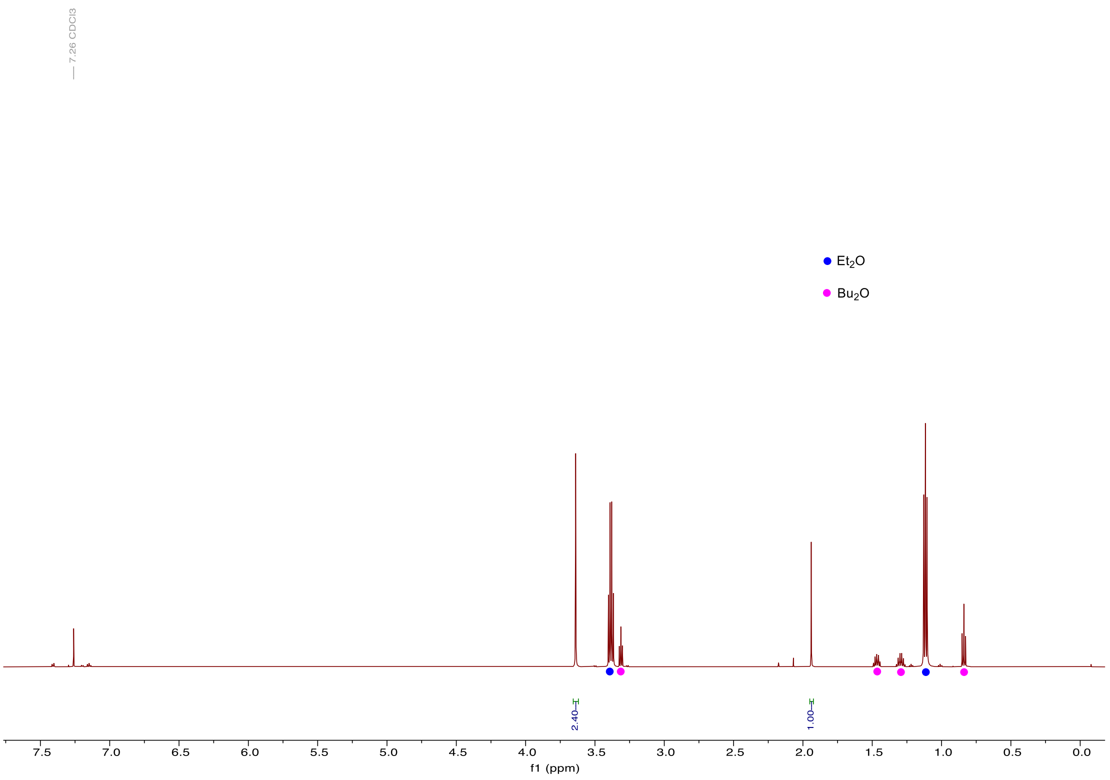

Calculation of concentration of [1.1.1]propellane batch 2:
3.07 (DCE, 4H) : 1.00 ([1.1.1]propellane, 6H)
(3.07/4) : (1.00/6) = 0.7675 : 0.167
(50 μL x 1.253 g/mL)/98.96 g/mol = 0.63 mmol
(0.63 mmol/(0.7675/0.167))/0.2 mL = 0.69 M

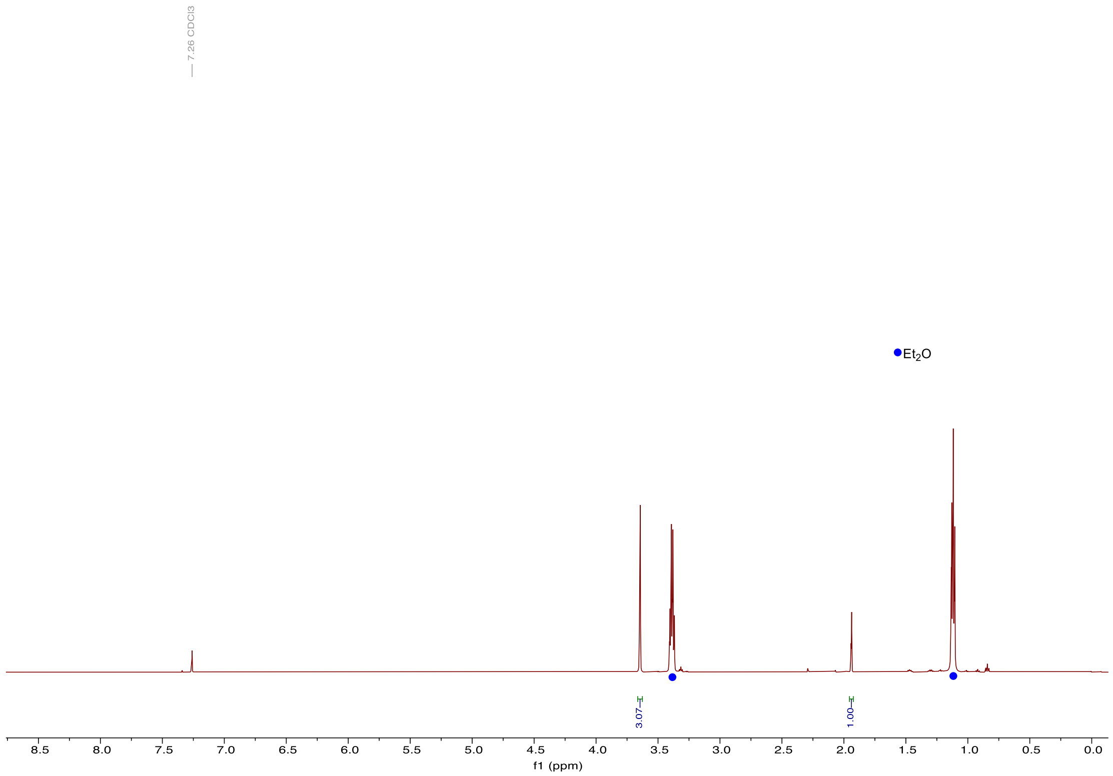

Both [1.1.1]propellane from batch 1 and batch 2 were tested following the general procedure with carboxylic acid **2j** and rendered the desired product **3j** in 90% and 88% isolated yield respectively. Both Et_2O and Bu_2O do not affect the reactions.

3.2 List of carboxylic acids for decarboxylative borylation reactions.

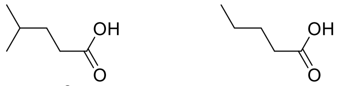
2a
2b

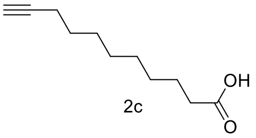
2c

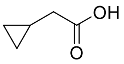
2d

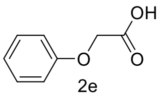
2e

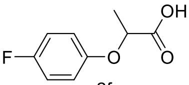
2f

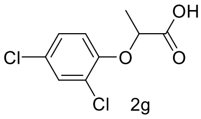
2g

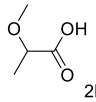
2h

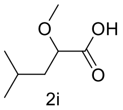
2i

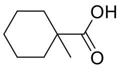
2j

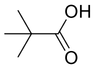
2k

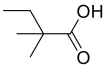
2l

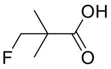
2m

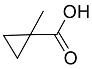
2n

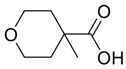
2o

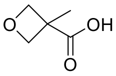
2p

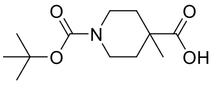
2q

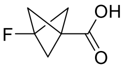
2r

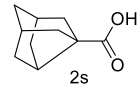
2s

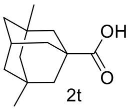
2t

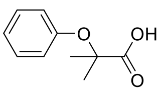
2u

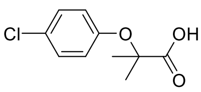
2v

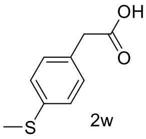
2w

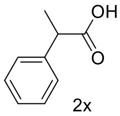
2x

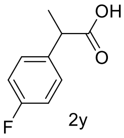
2y

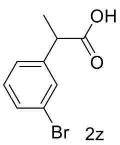
2z

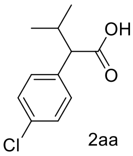
2aa

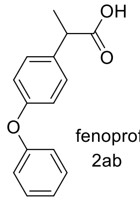
fenoprofen
2ab

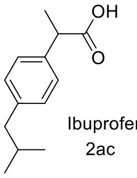
Ibuprofen
2ac

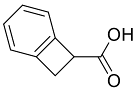
2ad

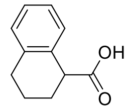
2ae

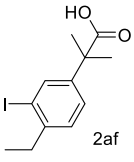
2af

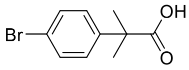
2ag

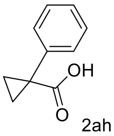
2ah

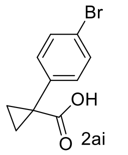
2ai

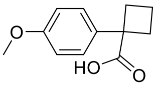
2aj

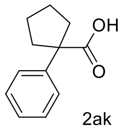
2ak

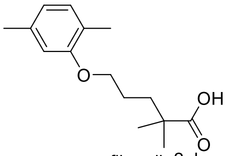
gemfibrozil 2al

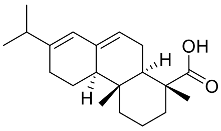
abietic acid 2am

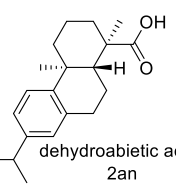
dehydroabietic acid
2an

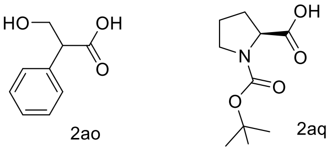
2ao
2aq

3.3 Characterization of MCR product.

2-(3-isopentylbicyclo[1.1.1]pentan-1-yl)-4,4,5,5-tetramethyl-1,3,2-dioxaborolane (3a)

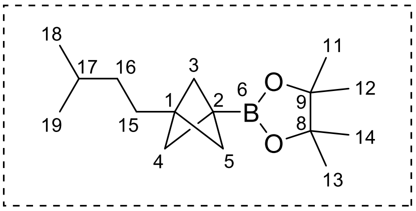

Synthesized according to *General Procedure* using carboxylic acid **2a** (23.2 mg, 0.2 mmol, 2.0 equiv.), [1.1.1]propellane (0.11 mL, 0.88 M in Et_2O solution, 0.1 mmol, 1.0 equiv.), bis(pinacolato)diboron (76.2 mg, 0.3 mmol, 3.0 equiv.), Cs_2CO_3 (6.5 mg, 0.02 mmol, 0.2 equiv.) and $Fe(NO_3)_3\cdot 9H_2O$ (4.0 mg, 0.01 mmol, 0.1 equiv.). Purification is through flash column chromatography (0-10% Et_2O/Hexane) to give product **3a** (16.4 mg, 0.062 mmol, 62%) as a white solid.

R_f 0.48 (6% Et_2O/hexane); **^{1}H NMR** (600 MHz, $CDCl_3$) δ 1.71 (s, 6H, H3, H4 and H5), 1.48 (dp, J = 13.3, 6.6 Hz, 1H, H17), 1.31 – 1.27 (m, 2H, H15), 1.22 (s, 12H, H11, H12, H13 and H14), 1.10 – 1.05 (m, 2H, H16), 0.84 (d, J = 6.6 Hz, 6H, H18 and H19); **^{13}C NMR** (151 MHz, $CDCl_3$) δ 83.3, 51.4, 46.4, 35.2, 31.2, 28.2, 24.9, 22.7; **^{11}B NMR** (193 MHz, $CDCl_3$); **^{11}B NMR** (192 MHz, $CDCl_3$) δ 30.6; **HRMS** (ESI^+) calc. for $C_{16}H_{29}BO_2$ $[M+H]^+$265.2336, found 265.2334.

2-(3-butylbicyclo[1.1.1]pentan-1-yl)-4,4,5,5-tetramethyl-1,3,2-dioxaborolane (3b)

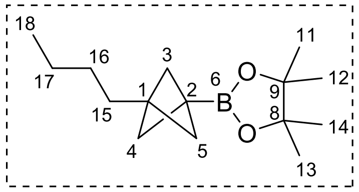

Synthesized according to *General Procedure* using carboxylic acid **2b** (20.4 mg, 0.2 mmol, 2.0 equiv.), [1.1.1]propellane (0.11 mL, 0.88 M in Et_2O solution, 0.1 mmol, 1.0 equiv.), bis(pinacolato)diboron (76.2 mg, 0.3 mmol, 3.0 equiv.), Cs_2CO_3 (6.5 mg, 0.02 mmol, 0.2 equiv.) and $Fe(NO_3)_3\cdot 9H_2O$ (4.0 mg, 0.01 mmol, 0.1 equiv.). Purification is through flash column chromatography (0-10% Et_2O/Hexane) to give product **3b** (20.3 mg, 0.081 mmol, 81%) as a colorless oil.

R_f 0.49 (6% Et_2O/hexane); **^{1}H NMR** (600 MHz, $CDCl_3$) δ 1.74 – 1.70 (m, 6H, H3, H4 and H5), 1.32 – 1.16 (m, 18H, H11, H12, H13, H14, H15, H16 and H17), 0.86 (t, J = 6.6 Hz, 3H, H18); **^{13}C NMR** (151 MHz, $CDCl_3$) δ 83.3, 51.5, 46.5, 33.1, 28.4, 24.9, 23.0, 14.2; **^{11}B NMR** (193 MHz, $CDCl_3$); **^{11}B NMR** (192 MHz, $CDCl_3$) δ 30.7; **HRMS** (ESI^+) calc. for $C_{15}H_{27}BO_2$ $[M+H]^+$251.2180, found 251.2179.

2-(3-(dec-9-yn-1-yl)bicyclo[1.1.1]pentan-1-yl)-4,4,5,5-tetramethyl-1,3,2-dioxaborolane (3c)

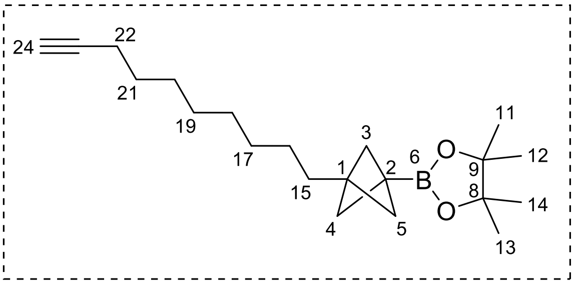

Synthesized according to *General Procedure* using carboxylic acid **2c** (36.5 mg, 0.2 mmol, 2.0 equiv.), [1.1.1]propellane (0.11 mL, 0.88 M in Et_2O solution, 0.1 mmol, 1.0 equiv.), bis(pinacolato)diboron (76.2 mg, 0.3 mmol, 3.0 equiv.), Cs_2CO_3 (6.5 mg, 0.02 mmol, 0.2 equiv.) and $Fe(NO_3)_3 \cdot 9H_2O$ (4.0 mg, 0.01 mmol, 0.1 equiv.). Purification is through flash column chromatography (0-10% Et_2O/Hexane) to give product **3c** (23.1 mg, 0.070 mmol, 70%) as a colorless oil.

R_f 0.43 (6% Et_2O/hexane); **^{1}H NMR** (600 MHz, $CDCl_3$) δ 2.19 – 2.14 (m, 2H, H22), 1.93 (s, 1H, H24), 1.72 (s, 6H, H3, H4 and H5), 1.54 – 1.47 (m, 2H, H21), 1.37 (t, *J* = 7.7 Hz, 2H, H15), 1.31 – 1.17 (m, 22H, H11, H12, H13, H14, H16, H17, H18, H19 and H20); **^{13}C NMR** (151 MHz, $CDCl_3$) δ 85.0, 83.3, 68.2, 51.6, 46.4, 33.4, 29.9, 29.6, 29.2, 28.9, 28.6, 26.2, 25.0, 24.9, 18.5; **^{11}B NMR** (192 MHz, $CDCl_3$) δ 30.8; **HRMS** (ESI^+) calc. for $C_{21}H_{35}BO_2$ $[M+H]^+$331.2807, found 331.2797.

2-(3-(but-3-en-1-yl)bicyclo[1.1.1]pentan-1-yl)-4,4,5,5-tetramethyl-1,3,2-dioxaborolane (3d)

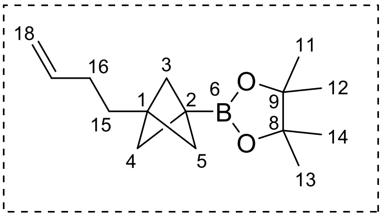

Synthesized according to *General Procedure* using carboxylic acid **2d** (20.0 mg, 0.2 mmol, 2.0 equiv.), [1.1.1]propellane (0.11 mL, 0.88 M in Et_2O solution, 0.1 mmol, 1.0 equiv.), bis(pinacolato)diboron (76.2 mg, 0.3 mmol, 3.0 equiv.), Cs_2CO_3 (6.5 mg, 0.02 mmol, 0.2 equiv.) and $Fe(NO_3)_3 \cdot 9H_2O$ (4.0 mg, 0.01 mmol, 0.1 equiv.). Purification is through flash column chromatography (0-10% Et_2O/Hexane) to give product **3d** (22.1 mg, 0.089 mmol, 89%) as a colorless oil.

R_f 0.45 (6% Et_2O/hexane); **^{1}H NMR** (600 MHz, $CDCl_3$) δ 5.81 (ddt, *J* = 16.8, 10.2, 6.5 Hz, 1H, H17), 4.98 (dq, *J* = 17.1, 1.8 Hz, 1H, H18), 4.90 (ddt, *J* = 10.2, 2.2, 1.3 Hz, 1H, H18'), 2.02 – 1.93 (m, 2H, H16), 1.74 (s, 6H, H3, H4 and H5), 1.45 – 1.38 (m, 2H, H15), 1.23 (s, 12H, H11, H12, H13 and H14); **^{13}C NMR** (151 MHz, $CDCl_3$) δ 139.3, 114.1, 83.3, 51.6, 46.1, 32.6, 30.6, 24.9; **^{11}B NMR** (192 MHz, $CDCl_3$) δ 30.6; **HRMS** (ESI^+) calc. for $C_{15}H_{25}BO_2$ $[M+H]^+$249.2023, found 249.2014.

4,4,5,5-tetramethyl-2-(3-(phenoxymethyl)bicyclo[1.1.1]pentan-1-yl)-1,3,2-dioxaborolane (3e)

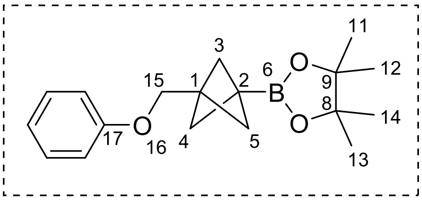

Synthesized according to *General Procedure* using carboxylic acid **2e** (30.4 mg, 0.2 mmol, 2.0 equiv.), [1.1.1]propellane (0.11 mL, 0.88 M in Et_2O solution, 0.1 mmol, 1.0 equiv.), bis(pinacolato)diboron (76.2 mg, 0.3 mmol, 3.0 equiv.), Cs_2CO_3 (6.5 mg, 0.02 mmol, 0.2 equiv.) and $Fe(NO_3)_3{\cdot}9H_2O$ (4.0 mg, 0.01 mmol, 0.1 equiv.). Purification is through flash column chromatography (0-10% Et_2O/Hexane) to give product **3e** (13.8 mg, 0.046 mmol, 46%) as a white solid.

$\mathbf{R}_f$ 0.35(10% Et_2O/hexane); **^{1}H NMR** (600 MHz, $CDCl_3$) δ 7.26 – 7.22 (m, 2H, ArH), 6.92 – 6.85 (m, 3H, ArH), 3.84 (s, 2H, H15), 1.92 (s, 6H, H3, H4 and H5), 1.24 (s, 12H, H11, H12, H13 and H14); **^{13}C NMR** (151 MHz, $CDCl_3$) 159.3, 129.4, 120.6, 114.7, 83.5, 68.5, 51.9, 51.0, 24.9; **^{11}B NMR** (192 MHz, $CDCl_3$) δ 30.6; **HRMS** (ESI$^+$) calc. for $C_{18}H_{25}BO_3$ $[M+Na]^+$323.1792, found 323.1791.

2-(3-(1-(4-fluorophenoxy)ethyl)bicyclo[1.1.1]pentan-1-yl)-4,4,5,5-tetramethyl-1,3,2-dioxaborolane (3f)

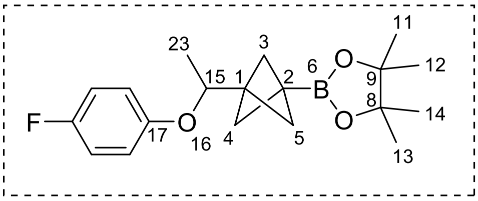

Synthesized according to *General Procedure* using carboxylic acid **2f** (36.8 mg, 0.2 mmol, 2.0 equiv.), [1.1.1]propellane (0.11 mL, 0.88 M in Et_2O solution, 0.1 mmol, 1.0 equiv.), bis(pinacolato)diboron (76.2 mg, 0.3 mmol, 3.0 equiv.), Cs_2CO_3 (6.5 mg, 0.02 mmol, 0.2 equiv.) and $Fe(NO_3)_3{\cdot}9H_2O$ (4.0 mg, 0.01 mmol, 0.1 equiv.). Purification is through flash column chromatography (0-10% Et_2O/Hexane) to give product **3f** (23.6 mg, 0.071 mmol, 71%) as a white solid.

$\mathbf{R}_f$ 0.32(10% Et_2O/hexane); **^{1}H NMR** (600 MHz, $CDCl_3$) δ 6.91 (td, J = 8.6, 1.0 Hz, 2H, ArH), 6.83 – 6.78 (m, 2H, ArH), 4.16 (q, J = 6.3 Hz, 1H, H15), 1.84 – 1.78 (m, 6H, H3, H4 and H5), 1.22 (s, 12H, H11, H12, H13 and H14), 1.18 (d, J = 5.3 Hz, 3H, H23); **^{13}C NMR** (151 MHz, $CDCl_3$) δ 157.2 (d, J = 237.7 Hz), 155.0 (d, J = 2.2 Hz), 117.2 (d, J = 7.8 Hz), 115.7 (d, J = 22.9 Hz), 83.5, 73.8, 51.9, 49.8, 47.8, 24.9, 16.5; **^{19}F NMR** (564 MHz, CDCl3) δ -124.3; **^{11}B NMR** (192 MHz, $CDCl_3$) δ 30.6; **HRMS** (ESI$^+$) calc. for $C_{19}H_{26}BFO_3$ $[M+Na]^+$355.1855, found 355.1849.

2-(3-(1-(2,4-dichlorophenoxy)ethyl)bicyclo[1.1.1]pentan-1-yl)-4,4,5,5-tetramethyl-1,3,2-dioxaborolane (3g)

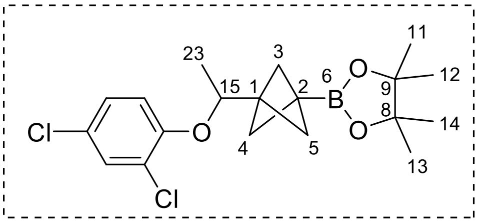

Synthesized according to *General Procedure* using carboxylic acid **2g** (47.0 mg, 0.2 mmol, 2.0 equiv.), [1.1.1]propellane (0.11 mL, 0.88 M in Et_2O solution, 0.1 mmol, 1.0 equiv.), bis(pinacolato)diboron (76.2 mg, 0.3 mmol, 3.0 equiv.), Cs_2CO_3 (6.5 mg, 0.02 mmol, 0.2 equiv.) and $Fe(NO_3)_3 \cdot 9H_2O$ (4.0 mg, 0.01 mmol, 0.1 equiv.). Purification is through flash column chromatography (0-10% Et_2O/Hexane) to give product **3g** (27.3 mg, 0.071mmol, 71%) as a white solid.

$\mathbf{R}_f$ 0.25(6% Et_2O/hexane); **^{1}H NMR** (600 MHz, $CDCl_3$) δ 7.32 (dd, *J* = 2.6, 1.0 Hz, 1H, ArH), 7.10 (ddd, *J* = 8.8, 2.6, 1.0 Hz, 1H, ArH), 6.84 (d, *J* = 8.8 Hz, 1H, ArH), 4.24 (q, *J* = 6.3 Hz, 1H, H15), 1.88 – 1.78 (m, 6H, H3, H4 and H5), 1.24-1.21 (m, 15H, H11, H12, H13, H14 and H23); **^{13}C NMR** (151 MHz, $CDCl_3$) δ 153.3, 130.1, 127.3, 125.5, 124.8, 116.3, 83.5, 75.0, 51.9, 49.8, 47.5, 24.9, 16.4; **^{11}B NMR** (192 MHz, $CDCl_3$) δ 30.8; **HRMS** (ESI^+) calc. for $C_{19}H_{25}BCl_2O_3$ $[M+Na]^+$405.1169, found 405.1164.

2-(3-(1-methoxyethyl)bicyclo[1.1.1]pentan-1-yl)-4,4,5,5-tetramethyl-1,3,2-dioxaborolane (3h)

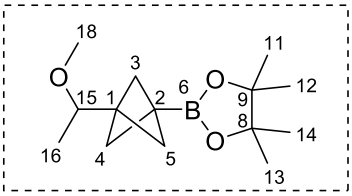

Synthesized according to *General Procedure* using carboxylic acid **2h** (20.8 mg, 0.2 mmol, 2.0 equiv.), [1.1.1]propellane (0.11 mL, 0.88 M in Et_2O solution, 0.1 mmol, 1.0 equiv.), bis(pinacolato)diboron (76.2 mg, 0.3 mmol, 3.0 equiv.), Cs_2CO_3 (6.5 mg, 0.02 mmol, 0.2 equiv.) and $Fe(NO_3)_3 \cdot 9H_2O$ (4.0 mg, 0.01 mmol, 0.1 equiv.). To the crude residue in EtOAc (1.0 mL) was added IBX (84 mg, 0.3 mmol, 3.0 equiv.). After stirring at 80 °C for 2 h, the reaction mixture was filtered off and concentrated under reduced pressure. Purification is through flash column chromatography (0-15% EtOAc/Hexane) to give product **3h** (20.7 mg, 0.082mmol, 82%) as a white solid.

$\mathbf{R}_f$ 0.40 (10% EtOAc/hexane); **^{1}H NMR** (600 MHz, $CDCl_3$) δ 3.34 (s, 3H, H18), 3.13 (q, *J* = 6.3 Hz, 1H, H15), 1.86 – 1.77 (m, 6H, H3, H4 and H5), 1.23 (s, 12H, H11, H12, H13 and H14), 1.01 (d, *J* = 6.3 Hz, 3H, H16); **^{13}C NMR** (151 MHz, $CDCl_3$) δ 83.4, 76.4, 57.3, 49.8, 24.9, 16.0; **^{11}B NMR** (192 MHz, $CDCl_3$) δ 30.7; **HRMS** (ESI^+) calc. for $C_{14}H_{25}BO_3$ $[M+Na]^+$275.1792, found 275.1784.

2-(3-(1-methoxy-3-methylbutyl)bicyclo[1.1.1]pentan-1-yl)-4,4,5,5-tetramethyl-1,3,2-dioxaborolane (3i)

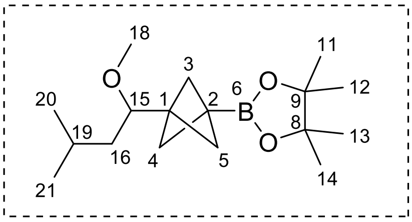

Synthesized according to *General Procedure* using carboxylic acid **2i** (29.2 mg, 0.2 mmol, 2.0 equiv.), [1.1.1]propellane (0.11 mL, 0.88 M in Et_2O solution, 0.1 mmol, 1.0 equiv.), bis(pinacolato)diboron (76.2 mg, 0.3 mmol, 3.0 equiv.), Cs_2CO_3 (6.5 mg, 0.02 mmol, 0.2 equiv.) and $Fe(NO_3)_3{\cdot}9H_2O$ (4.0 mg, 0.01 mmol, 0.1 equiv.). To the crude residue in EtOAc (1.0 mL) was added IBX (84 mg, 0.3 mmol, 3.0 equiv.). After stirring at 80 °C for 2 h, the reaction mixture was filtered off and concentrated under reduced pressure. Purification is through flash column chromatography (0-15% EtOAc/Hexane) to give product **3i** (23.8 mg, 0.081mmol, 81%) as a colorless oil.

$\mathbf{R}_f$ 0.45 (12% EtOAc/hexane); **^{1}H NMR** (600 MHz, $CDCl_3$) δ 3.37 (s, 3H, H18), 2.99 (dd, *J* = 9.6, 3.6 Hz, 1H, H15), 1.87 – 1.78 (m, 6H, H3, H4 and H5), 1.72 (dddd, *J* = 13.5, 11.6, 9.2, 5.5 Hz, 1H, H19), 1.29 – 1.20 (m, 13H, H11, H12, H13, H14 and H16), 1.05 (ddd, *J* = 14.1, 9.0, 3.6 Hz, 1H, H16'), 0.87 (dd, *J* = 17.9, 6.6 Hz, 6H; **^{13}C NMR** (151 MHz, $CDCl_3$) δ 83.4, 78.6, 58.5, 50.3, 48.0, 40.9, 24.9, 24.5, 23.7, 22.1; **^{11}B NMR** (192 MHz, $CDCl_3$) δ 30.8; **HRMS** (ESI^+) calc. for $C_{17}H_{31}BO_3$ $[M+Na]^+$317.2262, found 317.2257.

4,4,5,5-tetramethyl-2-(3-(1-methylcyclohexyl)bicyclo[1.1.1]pentan-1-yl)-1,3,2-dioxaborolane (3j)

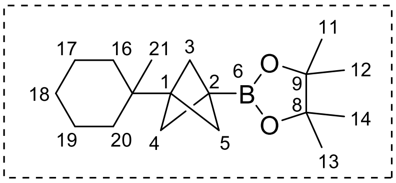

Synthesized according to *General Procedure* using carboxylic acid **2j** (28.4 mg, 0.2 mmol, 2.0 equiv.), [1.1.1]propellane (0.11 mL, 0.88 M in Et_2O solution, 0.1 mmol, 1.0 equiv.), bis(pinacolato)diboron (76.2 mg, 0.3 mmol, 3.0 equiv.), Cs_2CO_3 (6.5 mg, 0.02 mmol, 0.2 equiv.) and $Fe(NO_3)_3{\cdot}9H_2O$ (4.0 mg, 0.01 mmol, 0.1 equiv.). Purification is through flash column chromatography (0-10% Et_2O/Hexane) to give product **3j** (26.1 mg, 0.090 mmol, 90%) as a white solid.

$\mathbf{R}_f$ 0.58 (10% Et_2O/hexane); **^{1}H NMR** (600 MHz, $CDCl_3$) δ 1.68 (s, 6H, H3, H4 and H5), 1.57 – 1.52 (m, 1H, cyclo-Hex), 1.51-1.44 (m, 2H, cyclo-hex), 1.38 – 1.29 (m, 2H, cyclo-hex), 1.23 (s, 12H, H11, H12, H13 and H14), 1.16 – 1.07 (m, 5H, cyclo-hex), 0.75 (s, 3H, H21); **^{13}C NMR** (151 MHz, $CDCl_3$) δ 83.2, 54.5, 47.4, 33.0, 32.2, 26.5, 24.8, 22.0, 19.2; **^{11}B NMR** (192 MHz, $CDCl_3$) δ 31.1; **HRMS** (ESI^+) calc. for $C_{18}H_{31}BO_2$ $[M+H]^+$291.2493, found 291.2487.

2-(3-(*tert*-butyl)bicyclo[1.1.1]pentan-1-yl)-4,4,5,5-tetramethyl-1,3,2-dioxaborolane (3k)

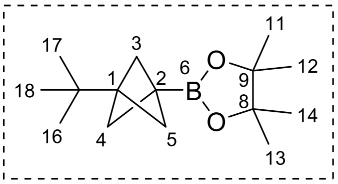

Synthesized according to *General Procedure* using carboxylic acid **2k** (20.4 mg, 0.2 mmol, 2.0 equiv.), [1.1.1]propellane (0.11 mL, 0.88 M in Et_2O solution, 0.1 mmol, 1.0 equiv.), bis(pinacolato)diboron (76.2 mg, 0.3 mmol, 3.0 equiv.), Cs_2CO_3 (6.5 mg, 0.02 mmol, 0.2 equiv.) and $Fe(NO_3)_3 \cdot 9H_2O$ (4.0 mg, 0.01 mmol, 0.1 equiv.). Purification is through flash column chromatography (0-10% Et_2O/Hexane) to give product **3k** (22.0 mg, 0.088 mmol, 88%) as a white solid.

$\mathbf{R_f}$ 0.41 (5% Et_2O/hexane); **^{1}H NMR** (600 MHz, $CDCl_3$) δ 1.67 (s, 6H, H3, H4 and H5), 1.23 (s, 12H, H11, H12, H13 and H14), 0.77 (s, 9H, H17, H18 and H19); **^{13}C NMR** (151 MHz, $CDCl_3$) δ 83.3, 53.9, 47.6, 30.2, 25.5, 24.9; **^{11}B NMR** (192 MHz, $CDCl_3$) δ 31.0; **HRMS** (ESI^+) calc. for $C_{15}H_{27}BO_2$ $[M+H]^+$251.2180, found 251.2178.

4,4,5,5-tetramethyl-2-(3-(tert-pentyl)bicyclo[1.1.1]pentan-1-yl)-1,3,2-dioxaborolane (3l)

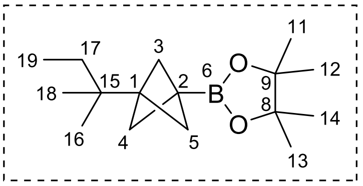

Synthesized according to *General Procedure* using carboxylic acid **2l** (23.2mg, 0.2 mmol, 2.0 equiv.), [1.1.1]propellane (0.11 mL, 0.88 M in Et_2O solution, 0.1 mmol, 1.0 equiv.), bis(pinacolato)diboron (76.2 mg, 0.3 mmol, 3.0 equiv.), Cs_2CO_3 (6.5 mg, 0.02 mmol, 0.2 equiv.) and $Fe(NO_3)_3 \cdot 9H_2O$ (4.0 mg, 0.01 mmol, 0.1 equiv.). Purification is through flash column chromatography (0-10% Et_2O/Hexane) to give product **3l** (22.4 mg, 0.085 mmol, 85%) as a white solid.

$\mathbf{R_f}$ 0.41 (5% Et_2O/hexane); **^{1}H NMR** (600 MHz, $CDCl_3$) δ 1.69 (s, 6H, H3, H4 and H5), 1.23 (s, 12H, H11, H12, H13 and H14), 1.17 (q, *J* = 7.6 Hz, 2H, H17), 0.79 (t, *J* = 7.5 Hz, 3H, H19), 0.70 (s, 6H, H16 and H18); **^{13}C NMR** (151 MHz, $CDCl_3$) δ 83.3, 54.3, 48.0, 32.6, 30.7, 24.9, 22.0, 8.9; **^{11}B NMR** (192 MHz, $CDCl_3$) δ 31.1; **HRMS** (ESI^+) calc. for $C_{16}H_{29}BO_2$ $[M+H]^+$265.2336, found 265.2333.

2-(3-(1-fluoro-2-methylpropan-2-yl)bicyclo[1.1.1]pentan-1-yl)-4,4,5,5-tetramethyl-1,3,2-dioxaborolane (3m)

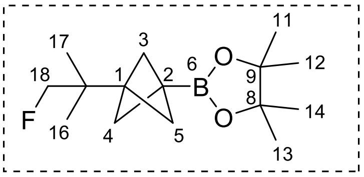

Synthesized according to *General Procedure* using carboxylic acid **2m** (24.0 mg, 0.2 mmol, 2.0 equiv.), [1.1.1]propellane (0.11 mL, 0.88 M in Et_2O solution, 0.1 mmol, 1.0 equiv.), bis(pinacolato)diboron (76.2 mg, 0.3 mmol, 3.0 equiv.), Cs_2CO_3 (6.5 mg, 0.02 mmol, 0.2 equiv.) and $Fe(NO_3)_3{\cdot}9H_2O$ (4.0 mg, 0.01 mmol, 0.1 equiv.). Purification is through flash column chromatography (0-10% Et_2O/Hexane) to give product **3m** (16.6 mg, 0.062 mmol, 62%) as a white solid.

R*f* 0.22 (5% Et_2O/hexane); **^{1}H NMR** (600 MHz, $CDCl_3$) δ 4.09 (d, *J* = 48.1 Hz, 2H, H18), 1.74 (s, 6H, H3, H4 and H5), 1.23 (s, 12H, H11, H12, H13 and H14), 0.82 (s, 6H, H16 and H17); **13C NMR** (151 MHz, $CDCl_3$) δ 90.1 (d, *J* = 173.4 Hz), 83.4, 50.6 (d, *J* = 4.0 Hz), 48.6, 34.9 (d, *J* = 16.9 Hz), 24.9, 19.8 (d, *J* = 5.4 Hz); **^{19}F NMR** (564 MHz, $CDCl_3$) δ -225.8； **^{11}B NMR** (192 MHz, $CDCl_3$) δ 30.4; **HRMS** (ESI^+) calc. for $C_{15}H_{26}BFO_2$ $[M+H]^+$269.2085, found 269.2085.

4,4,5,5-tetramethyl-2-(3-(1-methylcyclopropyl)bicyclo[1.1.1]pentan-1-yl)-1,3,2-dioxaborolane (3n)

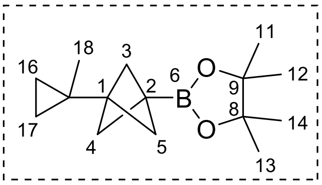

Synthesized according to *General Procedure* using carboxylic acid **2n** (24.0 mg, 0.2 mmol, 2.0 equiv.), [1.1.1]propellane (0.11 mL, 0.88 M in Et_2O solution, 0.1 mmol, 1.0 equiv.), bis(pinacolato)diboron (76.2 mg, 0.3 mmol, 3.0 equiv.), Cs_2CO_3 (6.5 mg, 0.02 mmol, 0.2 equiv.) and $Fe(NO_3)_3{\cdot}9H_2O$ (4.0 mg, 0.01 mmol, 0.1 equiv.). Purification is through flash column chromatography (0-10% Et_2O/Hexane) to give product **3n** (7.9 mg, 0.032 mmol, 32%) as a white solid.

R*f* 0.37 (5% Et_2O/hexane); **^{1}H NMR** (600 MHz, $CDCl_3$) δ 1.63 (s, 6H, H3, H4 and H5), 1.22 (s, 12H, H11, H12, H13 and H14), 0.96 (s, 3H, H18), 0.32 – 0.29 (m, 2H, H16), 0.11 – 0.07 (m, 2H, H17); **13C NMR** (151 MHz, $CDCl_3$) δ 83.3, 53.4, 50.0, 49.2, 24.9, 19.9, 16.4, 13.0, 9.9; **^{11}B NMR** (192 MHz, $CDCl_3$) δ 30.8; **HRMS** (ESI^+) calc. for $C_{15}H_{25}BO_2$ $[M+H]^+$249.2023, found 249.2022.

4,4,5,5-tetramethyl-2-(3-(4-methyltetrahydro-2H-pyran-4-yl)bicyclo[1.1.1]pentan-1-yl)-1,3,2-dioxaborolane (3o)

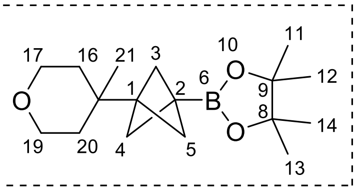

Synthesized according to *General Procedure* using carboxylic acid **2o** (28.8 mg, 0.2 mmol, 2.0 equiv.), [1.1.1]propellane (0.11 mL, 0.88 M in Et_2O solution, 0.1 mmol, 1.0 equiv.), bis(pinacolato)diboron (76.2 mg, 0.3 mmol, 3.0 equiv.), Cs_2CO_3 (6.5 mg, 0.02 mmol, 0.2 equiv.) and $Fe(NO_3)_3{\cdot}9H_2O$ (4.0 mg, 0.01 mmol, 0.1 equiv.). To the crude residue in EtOAc (1.0 mL) was added IBX (84 mg, 0.3 mmol, 3.0 equiv.). After stirring at 80 °C for 2 h, the reaction mixture was filtered off and concentrated under reduced pressure. Purification is through flash column chromatography (0-30% Et_2O/Hexane) to give product **3o** (23.4 mg, 0.080 mmol, 80%) as a white solid.

R_f 0.21 (15% Et_2O/hexane); **^{1}H NMR** (600 MHz, $CDCl_3$) δ 3.75 (dt, *J* = 11.5, 3.9 Hz, 2H, H17), 3.52 (td, *J* = 11.5, 2.5 Hz, 2H, H19), 1.70 (s, 6H, H3, H4 and H5), 1.57-1.50 (m, 2H, H16), 1.23 (s, 12H), 1.08 – 1.00 (m, 2H, H20), 0.87 (s, 3H, H21); **^{13}C NMR** (151 MHz, $CDCl_3$)δ 83.4, 64.3, 53.7, 47.4, 33.1, 30.4, 24.9, 18.9; **^{11}B NMR** (192 MHz, $CDCl_3$) δ 30.5; **HRMS** (ESI$^+$) calc. for $C_{17}H_{29}BO_3$ $[M+H]^+$293.2286, found 293.2283.

4,4,5,5-tetramethyl-2-(3-(3-methyloxetan-3-yl)bicyclo[1.1.1]pentan-1-yl)-1,3,2-dioxaborolane (3p)

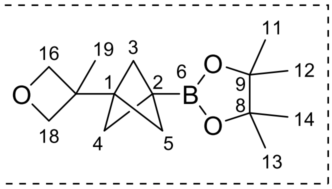

Synthesized according to *General Procedure* using carboxylic acid **2p** (23.2 mg, 0.2 mmol, 2.0 equiv.), [1.1.1]propellane (0.11 mL, 0.88 M in Et_2O solution, 0.1 mmol, 1.0 equiv.), bis(pinacolato)diboron (76.2 mg, 0.3 mmol, 3.0 equiv.), Cs_2CO_3 (6.5 mg, 0.02 mmol, 0.2 equiv.) and $Fe(NO_3)_3{\cdot}9H_2O$ (4.0 mg, 0.01 mmol, 0.1 equiv.). To the crude residue in EtOAc (1.0 mL) was added IBX (84 mg, 0.3 mmol, 3.0 equiv.). After stirring at 80 °C for 2 h, the reaction mixture was filtered off and concentrated under reduced pressure. Purification is through flash column chromatography (0-20% EtOAc/Hexane) to give product **3p** (18.0 mg, 0.068 mmol, 68%) as a white solid.

R_f 0.32 (15% EtOAc/hexane); **^{1}H NMR** (600 MHz, $CDCl_3$) δ 4.52 (d, *J* = 5.6 Hz, 2H, H16), 4.25 (d, *J* = 5.6 Hz, 2H, H18), 1.82 (s, 6H, H3, H4 and H5), 1.25 (s, 12H, H11, H12, H13 and H14), 1.18 (s, 3H); **^{13}C NMR** (151 MHz, $CDCl_3$) δ 83.5, 79.2, 49.3, 48.0, 39.4, 24.9, 20.3; **^{11}B NMR** (192 MHz, $CDCl_3$) δ 30.7; **HRMS** (ESI$^+$) calc. for $C_{15}H_{25}BO_3$ $[M+H]^+$265.1972, found 265.1968.

tert-butyl 4-methyl-4-(3-(4,4,5,5-tetramethyl-1,3,2-dioxaborolan-2-yl)bicyclo[1.1.1]pentan-1-yl)piperidine-1-carboxylate (3q)

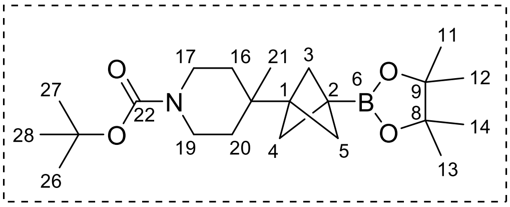

Synthesized according to *General Procedure* using carboxylic acid **2q** (48.7 mg, 0.2 mmol, 2.0 equiv.), [1.1.1]propellane (0.11 mL, 0.88 M in Et_2O solution, 0.1 mmol, 1.0 equiv.), bis(pinacolato)diboron (76.2 mg, 0.3 mmol, 3.0 equiv.), Cs_2CO_3 (6.5 mg, 0.02 mmol, 0.2 equiv.) and $Fe(NO_3)_3 \cdot 9H_2O$ (4.0 mg, 0.01 mmol, 0.1 equiv.). To the crude residue in EtOAc (1.0 mL) was added IBX (84 mg, 0.3 mmol, 3.0 equiv.). After stirring at 80 °C for 2 h, the reaction mixture was filtered off and concentrated under reduced pressure. Purification is through flash column chromatography (0-20% EtOAc/Hexane) to give product **3q** (32.5 mg, 0.083 mmol, 83%) as a white solid.

R_f 0.43 (15% EtOAc/hexane); **^{1}H NMR** (600 MHz, $CDCl_3$) δ 3.79 (brs, 2H, H17), 2.89 (t, J = 12.7 Hz, 2H, H19), 1.69 (s, 6H, H3, H4 and H5), 1.44 (s, 9H, H26, H27 and H28), 1.39 – 1.31 (m, 2H, H16), 1.23 (s, 12H, H11, H12, H13 and H14), 1.14 – 1.06 (m, 2H, H20), 0.82 (s, 3H, H21); **^{13}C NMR** (151 MHz, $CDCl_3$) δ 155.2, 83.4, 79.3, 53.5, 47.5, 31.1, 28.6, 24.9, 18.4; **^{11}B NMR** (192 MHz, $CDCl_3$) δ 31.1; **HRMS** (ESI$^+$) calc. for $C_{22}H_{25}BNO_4$ $[M+Na]^+$414.2790, found 414.2795.

2-(3'-fluoro-[1,1'-bi(bicyclo[1.1.1]pentan)]-3-yl)-4,4,5,5-tetramethyl-1,3,2-dioxaborolane (3r)

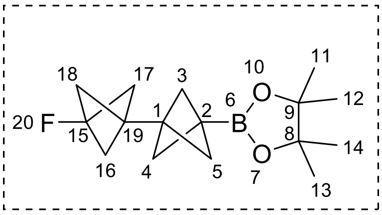

Synthesized according to *General Procedure* using carboxylic acid **2r** (26.0 mg, 0.2 mmol, 2.0 equiv.), [1.1.1]propellane (0.11 mL, 0.88 M in Et_2O solution, 0.1 mmol, 1.0 equiv.), bis(pinacolato)diboron (76.2 mg, 0.3 mmol, 3.0 equiv.), Cs_2CO_3 (6.5 mg, 0.02 mmol, 0.2 equiv.) and $Fe(NO_3)_3 \cdot 9H_2O$ (4.0 mg, 0.01 mmol, 0.1 equiv.). To the crude residue in EtOAc (1.0 mL) was added IBX (84 mg, 0.3 mmol, 3.0 equiv.). After stirring at 80 °C for 2 h, the reaction mixture was filtered off and concentrated under reduced pressure. Purification is through flash column chromatography (0-15% EtOAc/Hexane) to give product **3r** (18.1 mg, 0.065 mmol, 65%) as a white solid.

R_f 0.52 (12% EtOAc/hexane); **^{1}H NMR** (600 MHz, $CDCl_3$) δ 1.86 (d, J = 2.7 Hz, 6H, H16, H17 and H18), 1.75 (s, 6H, H3, H4 and H5), 1.23 (s, 12H, H11, H12, H13 and H14); **^{13}C NMR** (151 MHz, $CDCl_3$) δ 83.5, 75.6 (d, J = 328.7 Hz), 52.6 (d, J = 20.7 Hz), 50.9, 42.9, 29.9, 28.3, 24.9; **^{19}F NMR** (564 MHz, $CDCl_3$) δ -145.7; **^{11}B NMR** (192 MHz, $CDCl_3$) δ 30.7; **HRMS** (ESI$^+$) calc. for $C_{16}H_{24}BFO_2$ $[M+H]^+$279.1929, found 279.1929.

2-(3-((2*R*,3as,5*S*,6as)-hexahydro-2,5-methanopentalen-3a(1H)-yl)bicyclo[1.1.1]pentan-1-yl)-4,4,5,5-tetramethyl-1,3,2-dioxaborolane (3s)

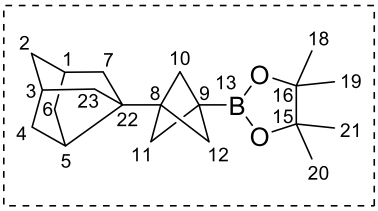

Synthesized according to *General Procedure* using carboxylic acid **2s** (33.2 mg, 0.2 mmol, 2.0 equiv.), [1.1.1]propellane (0.11 mL, 0.88 M in Et_2O solution, 0.1 mmol, 1.0 equiv.), bis(pinacolato)diboron (76.2 mg, 0.3 mmol, 3.0 equiv.), Cs_2CO_3 (6.5 mg, 0.02 mmol, 0.2 equiv.) and $Fe(NO_3)_3{\cdot}9H_2O$ (4.0 mg, 0.01 mmol, 0.1 equiv.). Purification is through flash column chromatography (0-10% Et_2O/Hexane) to give product **3s** (17.6 mg, 0.056 mmol, 56%) as a white solid.

R*f* 0.47 (5% Et_2O/hexane); **^{1}H NMR** (600 MHz, $CDCl_3$) δ 2.17 (brs, 2H, H23), 2.09 (t, *J* = 6.8 Hz, 1H, H5), 1.70 (s, 6H, H10, H11 and H12), 1.60 – 1.48 (m, 8H, H1, H2, H3, H4 and H7), 1.37 (dd, *J* = 10.6, 3.1 Hz, 2H, H6), 1.23 (s, 12H, H18, H19, H20 and H21); **13C NMR** (151 MHz, $CDCl_3$) δ 83.3, 50.4, 49.4, 48.7, 46.0, 45.0, 44.4, 39.6, 37.8, 37.5, 35.3, 24.9, 24.8; **^{11}B NMR** (192 MHz, $CDCl_3$) δ 31.0; **HRMS** (ESI^+) calc. for $C_{20}H_{31}BO_2$ $[M+H]^+$315.2494, found 315.2487.

2-(3-((1*r*,3*R*,5*S*,7*r*)-3,5-dimethyladamantan-1-yl)bicyclo[1.1.1]pentan-1-yl)-4,4,5,5-tetramethyl-1,3,2-dioxaborolane (3t)

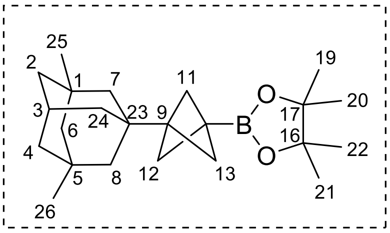

Synthesized according to *General Procedure* using carboxylic acid **2t** (41.7 mg, 0.2 mmol, 2.0 equiv.), [1.1.1]propellane (0.11 mL, 0.88 M in Et_2O solution, 0.1 mmol, 1.0 equiv.), bis(pinacolato)diboron (76.2 mg, 0.3 mmol, 3.0 equiv.), Cs_2CO_3 (6.5 mg, 0.02 mmol, 0.2 equiv.) and $Fe(NO_3)_3{\cdot}9H_2O$ (4.0 mg, 0.01 mmol, 0.1 equiv.). Purification is through flash column chromatography (0-10% Et_2O/Hexane) to give product **3t** (31.0 mg, 0.087 mmol, 87%) as a white solid.

R*f* 0.52 (6% Et_2O/hexane); **^{1}H NMR** (600 MHz, $CDCl_3$) δ 2.01 (p, *J* = 3.2 Hz, 1H, H3), 1.63 (s, 6H, H11, H12 and H13), 1.26 – 1.21 (m, 16H, H7, H8, H19, H20, H21 and H22), 1.20 – 1.17 (m, 2H, H24), 1.09 – 0.92 (m, 6H, H2, H4 and H6), 0.78 (s, 6H, H25 and H26); **13C NMR** (151 MHz, $CDCl_3$) δ 83.3, 53.0, 51.3, 47.0, 44.3, 43.4, 36.6, 33.7, 30.9, 30.9, 29.6, 24.9; **^{11}B NMR** (192 MHz, $CDCl_3$) δ 30.3; **HRMS** (ESI^+) calc. for $C_{23}H_{37}BO_2$ $[M+Na]^+$379.2783, found 379.2774.

4,4,5,5-tetramethyl-2-(3-(2-phenoxypropan-2-yl)bicyclo[1.1.1]pentan-1-yl)-1,3,2-dioxaborolane (3u)

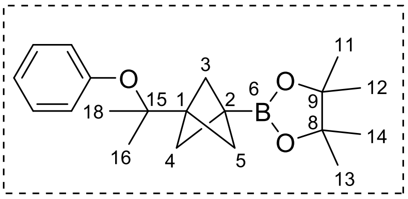

Synthesized according to *General Procedure* using carboxylic acid **2u** (36.0 mg, 0.2 mmol, 2.0 equiv.), [1.1.1]propellane (0.11 mL, 0.88 M in Et_2O solution, 0.1 mmol, 1.0 equiv.), bis(pinacolato)diboron (76.2 mg, 0.3 mmol, 3.0 equiv.), Cs_2CO_3 (6.5 mg, 0.02 mmol, 0.2 equiv.) and $Fe(NO_3)_3·9H_2O$ (4.0 mg, 0.01 mmol, 0.1 equiv.). Purification is through flash column chromatography (0-10% Et_2O/Hexane) to give product **3u** (27.2mg, 0.083 mmol, 83%) as a white solid.

$\mathbf{R}_f$ 0.24 (6% Et_2O/hexane); **^{1}H NMR** (600 MHz, $CDCl_3$) δ 7.24-7.20 (m, 2H, ArH), 7.02 (td, *J* = 7.5, 1.2 Hz, 1H, ArH), 6.98 – 6.93 (m, 2H, ArH), 1.87 (s, 6H, H3, H4 and H5), 1.24 (s, 12H, H11, H12, H13 and H14), 1.17 (s, 6H, H16 and H18); **13C NMR** (151 MHz, $CDCl_3$) δ 156.1, 128.9, 123.9, 123.0, 83.4, 78.3, 51.8, 48.8, 24.9, 23.0; **^{11}B NMR** (192 MHz, $CDCl_3$) δ 31.0; **HRMS** (ESI^+) calc. for $C_{20}H_{29}BO_3$ $[M+H]^+$329.2286, found 329.2279.

2-(3-(2-(4-chlorophenoxy)propan-2-yl)bicyclo[1.1.1]pentan-1-yl)-4,4,5,5-tetramethyl-1,3,2-dioxaborolane (3v)

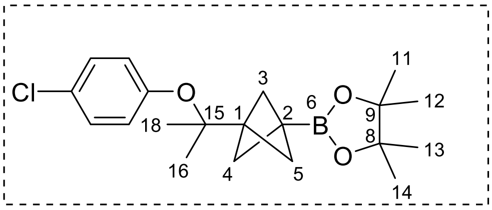

Synthesized according to *General Procedure* using carboxylic acid **2v** (42.9 mg, 0.2 mmol, 2.0 equiv.), [1.1.1]propellane (0.11 mL, 0.88 M in Et_2O solution, 0.1 mmol, 1.0 equiv.), bis(pinacolato)diboron (76.2 mg, 0.3 mmol, 3.0 equiv.), Cs_2CO_3 (6.5 mg, 0.02 mmol, 0.2 equiv.) and $Fe(NO_3)_3·9H_2O$ (4.0 mg, 0.01 mmol, 0.1 equiv.). Purification is through flash column chromatography (0-10% Et_2O/Hexane) to give product **3v** (26.5 mg, 0.073 mmol, 73%) as a white solid.

$\mathbf{R}_f$ 0.28 (6% Et_2O/hexane); **^{1}H NMR** (600 MHz, $CDCl_3$) δ 7.19-7.16 (m, 2H, ArH), 6.90 – 6.86 (m, 2H, ArH), 1.85 (s, 6H, H3, H4 and H5), 1.24 (s, 12H, H11, H12, H13 and H14), 1.15 (s, 6H, H16 and H18); **13C NMR** (151 MHz, $CDCl_3$) δ 154.8, 128.9, 125.1, 116.8, 83.5, 78.9, 51.8, 48.9, 24.9, 22.9; **^{11}B NMR** (192 MHz, $CDCl_3$) δ 30.7; **HRMS** (ESI^+) calc. for $C_{20}H_{28}BClO_3$ $[M+H]^+$363.1896, found 363.1887.

4,4,5,5-tetramethyl-2-(3-(4-(methylthio)benzyl)bicyclo[1.1.1]pentan-1-yl)-1,3,2-dioxaborolane (3w)

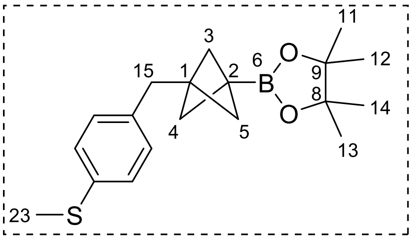

Synthesized according to *General Procedure* using carboxylic acid **2w** (36.4 mg, 0.2 mmol, 2.0 equiv.), [1.1.1]propellane (0.11 mL, 0.88 M in Et_2O solution, 0.1 mmol, 1.0 equiv.), bis(pinacolato)diboron (76.2 mg, 0.3 mmol, 3.0 equiv.), Cs_2CO_3 (6.5 mg, 0.02 mmol, 0.2 equiv.) and $Fe(NO_3)_3{\cdot}9H_2O$ (4.0 mg, 0.01 mmol, 0.1 equiv.). Purification is through flash column chromatography (0-10% Et_2O/Hexane) to give product **3w** (19.8 mg, 0.060 mmol, 60%) as a white solid.

***R*_f_** 0.30 (5% Et_2O/hexane); **^{1}H NMR** (600 MHz, $CDCl_3$) δ 7.19 – 7.14 (m, 2H, ArH), 7.01 – 6.96 (m, 2H, ArH), 2.61 (s, 2H, H15), 2.46 (s, 3H, H23), 1.69 (s, 6H, H3, H4 and H5), 1.20 (s, 12H, H11, H12, H13 and H14); **13C NMR** (151 MHz, $CDCl_3$) δ 136.8, 135.1, 129.6, 127.2, 83.3, 51.3, 45.8, 40.2, 24.8, 16.6; **^{11}B NMR** (192 MHz, $CDCl_3$) δ 30.6; **HRMS** (ESI^+) calc. for $C_{19}H_{27}BO_2S$ $[M+H]^+$331.1901, found 331.1895.

4,4,5,5-tetramethyl-2-(3-(1-phenylethyl)bicyclo[1.1.1]pentan-1-yl)-1,3,2-dioxaborolane (3x)

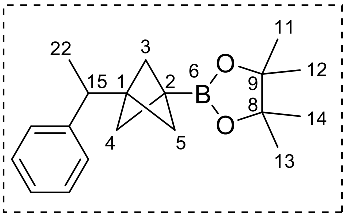

Synthesized according to *General Procedure* using carboxylic acid **2x** (30.0 mg, 0.2 mmol, 2.0 equiv.), [1.1.1]propellane (0.11 mL, 0.88 M in Et_2O solution, 0.1 mmol, 1.0 equiv.), bis(pinacolato)diboron (76.2 mg, 0.3 mmol, 3.0 equiv.), Cs_2CO_3 (6.5 mg, 0.02 mmol, 0.2 equiv.) and $Fe(NO_3)_3{\cdot}9H_2O$ (4.0 mg, 0.01 mmol, 0.1 equiv.). Purification is through flash column chromatography (0-10% Et_2O/Hexane) to give product **3x** (16.1 mg, 0.054 mmol, 54%) as a white solid.

***R*_f_** 0.45 (5% Et_2O/hexane); **^{1}H NMR** (600 MHz, $CDCl_3$) δ 7.27 – 7.23 (m, 2H, ArH), 7.15 (td, *J* = 7.4, 1.9 Hz, 1H, ArH), 7.11 – 7.08 (m, 2H, ArH), 2.76 – 2.70 (m, 1H, H15), 1.68 – 1.62 (m, 6H, H3, H4 and H5), 1.22-1,17 (m, 15H, H11, H12, H13, H14 and H22); **13C NMR** (151 MHz, $CDCl_3$) δ 144.2, 128.1, 127.6, 125.9, 83.3, 49.8, 49.5, 42.1, 24.8, 16.3; **^{11}B NMR** (192 MHz, $CDCl_3$) δ 31.0; **HRMS** (ESI^+) calc. for $C_{19}H_{27}BO_2$ $[M+H]^+$299.2180, found 299.2171.

2-(3-(1-(4-fluorophenyl)ethyl)bicyclo[1.1.1]pentan-1-yl)-4,4,5,5-tetramethyl-1,3,2-dioxaborolane (3y)

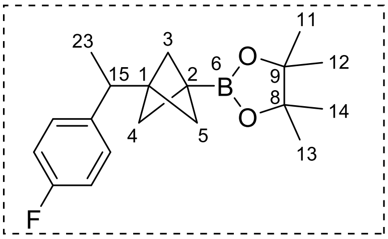

Synthesized according to *General Procedure* using carboxylic acid **2y** (33.6 mg, 0.2 mmol, 2.0 equiv.), [1.1.1]propellane (0.11 mL, 0.88 M in Et_2O solution, 0.1 mmol, 1.0 equiv.), bis(pinacolato)diboron (76.2 mg, 0.3 mmol, 3.0 equiv.), Cs_2CO_3 (6.5 mg, 0.02 mmol, 0.2 equiv.) and $Fe(NO_3)_3{\cdot}9H_2O$ (4.0 mg, 0.01 mmol, 0.1 equiv.). Purification is through flash column chromatography (0-10% Et_2O/Hexane) to give product **3y** (19.0 mg, 0.060 mmol, 60%) as a white solid.

$\mathbf{R_f}$ 0.42 (6% Et_2O/hexane); ^{1}**H NMR** (600 MHz, $CDCl_3$) δ 7.06 – 7.01 (m, 2H, ArH), 6.96 – 6.91 (m, 2H, ArH), 2.72 (q, *J* = 7.1 Hz, 1H, H15), 1.66-1.60 (m, 6H, H3, H4 and H5), 1.20 (s, 12H, H11, H12, H13 and H14), 1.17 (d, *J* = 7.1, 3H, H23); **13C NMR** (151 MHz, $CDCl_3$) δ161.4 (d, *J* = 242.8 Hz), 139.8 (d, *J* = 3.1 Hz), 128.8 (d, *J* = 7.7 Hz), 114.8 (d, *J* = 20.9 Hz), 83.4, 49.7, 49.4, 41.4, 24.9, 16.4; ^{19}**F NMR** (564 MHz, $CDCl_3$) δ -118.2; ^{11}**B NMR** (192 MHz, $CDCl_3$) δ 30.9; **HRMS** (ESI^+) calc. for $C_{19}H_{26}BFO_2$ $[M+H]^+$317.2086, found 317.2079.

2-(3-(1-(3-bromophenyl)ethyl)bicyclo[1.1.1]pentan-1-yl)-4,4,5,5-tetramethyl-1,3,2-dioxaborolane (3z)

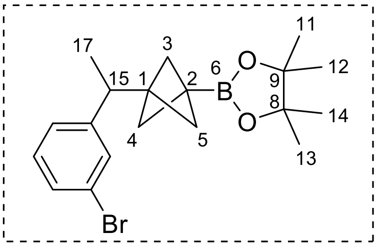

Synthesized according to *General Procedure* using carboxylic acid **2z** (45.8 mg, 0.2 mmol, 2.0 equiv.), [1.1.1]propellane (0.11 mL, 0.88 M in Et_2O solution, 0.1 mmol, 1.0 equiv.), bis(pinacolato)diboron (76.2 mg, 0.3 mmol, 3.0 equiv.), Cs_2CO_3 (6.5 mg, 0.02 mmol, 0.2 equiv.) and $Fe(NO_3)_3{\cdot}9H_2O$ (4.0 mg, 0.01 mmol, 0.1 equiv.). Purification is through flash column chromatography (0-10% Et_2O/Hexane) to give product **3z** (23.0 mg, 0.061 mmol, 61%) as a colorless oil.

$\mathbf{R_f}$ 0.33 (5% Et_2O/hexane); ^{1}**H NMR** (600 MHz, $CDCl_3$) δ 7.28 (dd, *J* = 8.2, 1.8 Hz, 1H, ArH), 7.23 (s, 1H, ArH), 7.12 (td, *J* = 7.8, 1.3 Hz, 1H, ArH), 7.01 (dd, *J* = 7.7, 1.6 Hz, 1H, ArH), 2.70 (q, *J* = 7.1 Hz, 1H, H15), 1.68 – 1.62 (m, 6H, H3, H4 and H5), 1.20 (s, 12H, H11, H12, H13 and H14), 1.17 (d, *J* = 7.1, 3H, H17); **13C NMR** (151 MHz, $CDCl_3$) δ 146.6, 130.5, 129.7, 129.0, 126.3, 122.3, 83.4, 49.5, 49.4, 41.9, 24.8, 16.2; ^{11}**B NMR** (192 MHz, $CDCl_3$) δ 30.8; **HRMS** (ESI^+) calc. for $C_{19}H_{26}BBrO_2$ $[M+H]^+$377.1285, found 377.1286.

2-(3-(1-(4-chlorophenyl)-2-methylpropyl)bicyclo[1.1.1]pentan-1-yl)-4,4,5,5-tetramethyl-1,3,2-dioxaborolane (3aa)

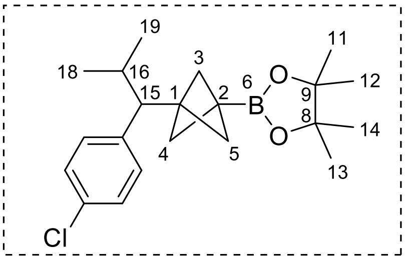

Synthesized according to *General Procedure* using carboxylic acid **2aa** (42.5 mg, 0.2 mmol, 2.0 equiv.), [1.1.1]propellane (0.11 mL, 0.88 M in Et_2O solution, 0.1 mmol, 1.0 equiv.), bis(pinacolato)diboron (76.2 mg, 0.3 mmol, 3.0 equiv.), Cs_2CO_3 (6.5 mg, 0.02 mmol, 0.2 equiv.) and $Fe(NO_3)_3·9H_2O$ (4.0 mg, 0.01 mmol, 0.1 equiv.). Purification is through flash column chromatography (0-10% Et_2O/Hexane) to give product **3aa** (17.7 mg, 0.049 mmol, 49%) as a white solid.

$\mathbf{R}_f$ 0.26 (5% Et_2O/hexane); **^{1}H NMR** (600 MHz, $CDCl_3$) δ 7.20 (d, *J* = 8.6 Hz, 2H, ArH), 6.96 (d, *J* = 8.7 Hz, 2H, ArH), 2.23 (d, *J* = 8.7 Hz, 1H, H15), 1.81 – 1.69 (m, 6H, H3, H4 and H5), 1.19 (s, 12H, H11, H12, H13 and H14), 1.02 (d, *J* = 6.7 Hz, 3H, H18), 0.66 (d, *J* = 6.7 Hz, 3H, H19); **^{13}C NMR** (151 MHz, $CDCl_3$) δ 141.4, 131.4, 130.2, 128.0, 83.4, 55.5, 51.8, 48.7, 30.4, 24.8, 22.5, 21.8; **^{11}B NMR** (192 MHz, $CDCl_3$) δ 30.2; **HRMS** (ESI$^+$) calc. for $C_{21}H_{30}BClO_2$ $[M+H]^+$361.2105, found 361.2104.

4,4,5,5-tetramethyl-2-(3-(1-(4-phenoxyphenyl)ethyl)bicyclo[1.1.1]pentan-1-yl)-1,3,2-dioxaborolane (3ab)

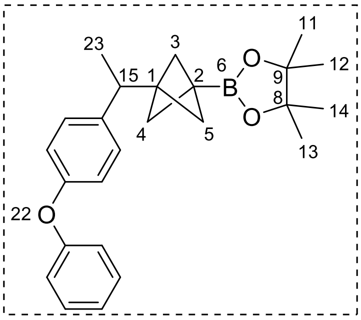

Synthesized according to *General Procedure* using carboxylic acid fenoprofen **2ab** (48.5 mg, 0.2 mmol, 2.0 equiv.), [1.1.1]propellane (0.11 mL, 0.88 M in Et_2O solution, 0.1 mmol, 1.0 equiv.), bis(pinacolato)diboron (76.2 mg, 0.3 mmol, 3.0 equiv.), Cs_2CO_3 (6.5 mg, 0.02 mmol, 0.2 equiv.) and $Fe(NO_3)_3·9H_2O$ (4.0 mg, 0.01 mmol, 0.1 equiv.). Purification is through flash column chromatography (0-10% Et_2O/Hexane) to give product **3ab** (24.6 mg, 0.063 mmol, 63%) as a white solid.

$\mathbf{R}_f$ 0.29 (5% Et_2O/hexane); **^{1}H NMR** (600 MHz, $CDCl_3$) δ 7.35 – 7.30 (m, 2H, ArH), 7.21 (td, *J* = 7.7, 1.7 Hz, 1H, ArH), 7.07 (t, *J* = 7.4 Hz, 1H, ArH), 7.00 – 6.95 (m, 2H, ArH), 6.85 (d, *J* = 7.6 Hz, 1H, ArH), 6.82 – 6.77 (m, 2H, ArH), 2.71 (q, *J* = 7.1 Hz, 1H, H15), 1.68 – 1.61 (m, 6H, H3, H4 and H5), 1.21 (s, 12H, H11, H12, H13 and H14), 1.17 (d, *J* = 7.1, 3H, H23); **^{13}C NMR** (151 MHz, $CDCl_3$) δ 157.8, 156.9, 146.4, 129.8, 129.2, 123.0, 122.8, 118.6, 118.4, 116.6, 83.4, 49.6, 49.5, 42.0, 24.9, 16.1; **^{11}B NMR** (192 MHz, $CDCl_3$) δ 30.9; **HRMS** (ESI$^+$) calc. for $C_{25}H_{31}BO_3$ $[M+H]^+$391.2444, found 391.2438.

2-(3-(1-(4-isobutylphenyl)ethyl)bicyclo[1.1.1]pentan-1-yl)-4,4,5,5-tetramethyl-1,3,2-dioxaborolane (3ac)

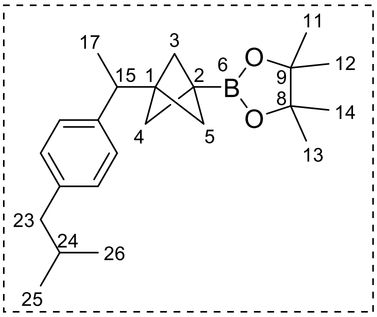

Synthesized according to *General Procedure* using carboxylic acid Ibuprofen **2ac** (41.3 mg, 0.2 mmol, 2.0 equiv.), [1.1.1]propellane (0.11 mL, 0.88 M in Et_2O solution, 0.1 mmol, 1.0 equiv.), bis(pinacolato)diboron (76.2 mg, 0.3 mmol, 3.0 equiv.), Cs_2CO_3 (6.5 mg, 0.02 mmol, 0.2 equiv.) and $Fe(NO_3)_3·9H_2O$ (4.0 mg, 0.01 mmol, 0.1 equiv.). Purification is through flash column chromatography (0-10% Et_2O/Hexane) to give product **3ac** (20.2 mg, 0.057 mmol, 57%) as a white solid.

R*f* 0.35 (5% Et_2O/hexane); **^{1}H NMR** (600 MHz, $CDCl_3$) δ 7.04 – 6.95 (m, 4H, ArH), 2.70 (q, *J* = 7.2 Hz, 1H, H15), 2.42 (dd, *J* = 7.2, 1.9 Hz, 2H, H23), 1.82 (hept, *J* = 6.4 Hz, 1H, H24), 1.67 – 1.60 (m, 6H, H3, H4 and H5), 1.20 (s, 12H, H11, H12, H13 and H14), 1.18 (d, *J* = 7.2 Hz, 3H, H17), 0.88 (d, *J* = 6.7 Hz, 6H, H25 and H26); **^{13}C NMR** (151 MHz, $CDCl_3$) δ 141.3, 139.1, 128.8, 127.2, 83.3, 49.9, 49.5, 45.2, 41.6, 30.4, 24.8, 22.6, 16.3; **^{11}B NMR** (192 MHz, $CDCl_3$) δ 31.1; **HRMS** (ESI$^+$) calc. for $C_{23}H_{35}BO_2$ $[M+H]^+$355.2807, found 355.2806.

2-(3-(bicyclo[4.2.0]octa-1(6),2,4-trien-7-yl)bicyclo[1.1.1]pentan-1-yl)-4,4,5,5-tetramethyl-1,3,2-dioxaborolane (3ad)

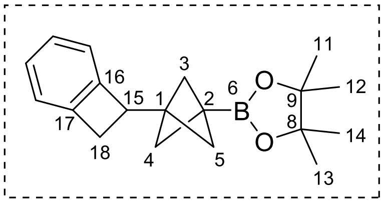

Synthesized according to *General Procedure* using carboxylic acid **2ad** (29.6 mg, 0.2 mmol, 2.0 equiv.), [1.1.1]propellane (0.11 mL, 0.88 M in Et_2O solution, 0.1 mmol, 1.0 equiv.), bis(pinacolato)diboron (76.2 mg, 0.3 mmol, 3.0 equiv.), Cs_2CO_3 (6.5 mg, 0.02 mmol, 0.2 equiv.) and $Fe(NO_3)_3·9H_2O$ (4.0 mg, 0.01 mmol, 0.1 equiv.). Purification is through flash column chromatography (0-10% Et_2O/Hexane) to give product **3ad** (15.1 mg, 0.051 mmol, 51%) as a white solid.

R*f* 0.35 (6% Et_2O/hexane); **^{1}H NMR** (600 MHz, $CDCl_3$) δ 7.18 – 7.12 (m, 2H, ArH), 7.04 (d, *J* = 6.2 Hz, 1H, ArH), 7.00 (d, *J* = 5.9 Hz, 1H, ArH), 3.47 (dd, *J* = 5.6, 2.5 Hz, 1H, H15), 3.13 (dd, *J* = 13.9, 5.4 Hz, 1H, H18), 2.84 (dd, *J* = 13.9, 2.6 Hz, 1H, H18'), 1.83-1.74 (m, 6H, H3, H4 and H5), 1.22 (s, 12H, H11, H12, H13 and H14); **^{13}C NMR** (151 MHz, $CDCl_3$) δ 147.3, 144.6, 127.0, 126.6, 122.9, 122.2, 83.4, 50.2, 46.8, 45.6, 32.4, 24.9; **^{11}B NMR** (192 MHz, $CDCl_3$) δ 30.2; **HRMS** (ESI$^+$) calc. for $C_{19}H_{25}BO_2$ $[M+H]^+$297.2024, found 297.2020.

4,4,5,5-tetramethyl-2-(3-(1,2,3,4-tetrahydronaphthalen-1-yl)bicyclo[1.1.1]pentan-1-yl)-1,3,2-dioxaborolane (3ae)

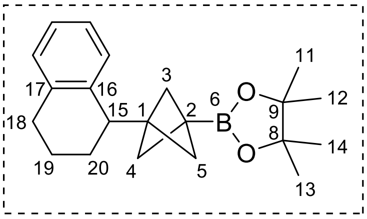

Synthesized according to *General Procedure* using carboxylic acid **2ae** (35.2 mg, 0.2 mmol, 2.0 equiv.), [1.1.1]propellane (0.11 mL, 0.88 M in Et_2O solution, 0.1 mmol, 1.0 equiv.), bis(pinacolato)diboron (76.2 mg, 0.3 mmol, 3.0 equiv.), Cs_2CO_3 (6.5 mg, 0.02 mmol, 0.2 equiv.) and $Fe(NO_3)_3 \cdot 9H_2O$ (4.0 mg, 0.01 mmol, 0.1 equiv.). Purification is through flash column chromatography (0-10% Et_2O/Hexane) to give product **3ae** (16.2 mg, 0.050 mmol, 50%) as a white solid.

R_f 0.22 (5% Et_2O/hexane); **^{1}H NMR** (600 MHz, $CDCl_3$) δ 7.10 – 7.02 (m, 4H, ArH), 2.77 – 2.74 (m, 1H, H15), 2.73 – 2.63 (m, 2H, H18), 1.86 – 1.70 (m, 9H, H3, H4, H5, H19 and H20), 1.69 – 1.62 (m, 1H, H20'), 1.21 (s, 12H, H11, H12, H13 and H14); **^{13}C NMR** (151 MHz, $CDCl_3$) δ 137.5, 137.4, 130.2, 129.1, 125.5, 125.0, 83.3, 51.0, 50.1, 39.7, 29.8, 25.9, 24.9, 24.8, 20.2; **^{11}B NMR** (192 MHz, $CDCl_3$) δ 30.6; **HRMS** (ESI^+) calc. for $C_{21}H_{29}BO_2$ $[M+H]^+$325.2337, found 325.2334.

2-(3-(2-(4-ethyl-3-iodophenyl)propan-2-yl)bicyclo[1.1.1]pentan-1-yl)-4,4,5,5-tetramethyl-1,3,2-dioxaborolane (3af)

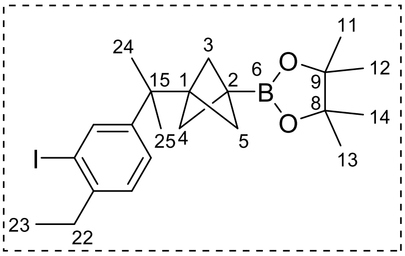

Synthesized according to *General Procedure* using carboxylic acid **2af** (63.6 mg, 0.2 mmol, 2.0 equiv.), [1.1.1]propellane (0.11 mL, 0.88 M in Et_2O solution, 0.1 mmol, 1.0 equiv.), bis(pinacolato)diboron (76.2 mg, 0.3 mmol, 3.0 equiv.), Cs_2CO_3 (6.5 mg, 0.02 mmol, 0.2 equiv.) and $Fe(NO_3)_3 \cdot 9H_2O$ (4.0 mg, 0.01 mmol, 0.1 equiv.). Purification is through flash column chromatography (0-10% Et_2O/Hexane) to give product **3af** (29.8 mg, 0.064 mmol, 64%) as a yellow oil.

R_f 0.39 (5% Et_2O/hexane); **^{1}H NMR** (600 MHz, $CDCl_3$) δ 7.68 (s, 1H, ArH), 7.17 (d, *J* = 7.7 Hz, 1H, ArH), 7.10 (d, *J* = 8.0 Hz, 1H, ArH), 2.71 – 2.67 (m, 2H, H22), 1.62 (s, 6H, H3, H4 and H5), 1.21 – 1.17 (m, 21H, H11, H12, H13, H14, H23, H24 and H25); **^{13}C NMR** (151 MHz, $CDCl_3$) δ 146.3, 143.5, 137.6, 127.6, 126.7, 100.4, 83.4, 53.3, 48.2, 37.8, 33.7, 24.9, 24.2, 14.7, 0.1; **^{11}B NMR** (192 MHz, $CDCl_3$) δ 30.9; **HRMS** (ESI^+) calc. for $C_{22}H_{32}BIO_2$ $[M+H]^+$467.1613, not found.

2-(3-(2-(4-bromophenyl)propan-2-yl)bicyclo[1.1.1]pentan-1-yl)-4,4,5,5-tetramethyl-1,3,2-dioxaborolane (3ag)

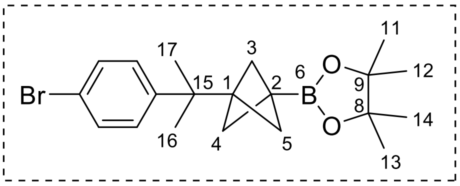

Synthesized according to *General Procedure* using carboxylic acid **2ag** (48.6 mg, 0.2 mmol, 2.0 equiv.), [1.1.1]propellane (0.11 mL, 0.88 M in Et_2O solution, 0.1 mmol, 1.0 equiv.), bis(pinacolato)diboron (76.2 mg, 0.3 mmol, 3.0 equiv.), Cs_2CO_3 (6.5 mg, 0.02 mmol, 0.2 equiv.) and $Fe(NO_3)_3{\cdot}9H_2O$ (4.0 mg, 0.01 mmol, 0.1 equiv.). Purification is through flash column chromatography (0-10% Et_2O/Hexane) to give product **3ag** (21.1 mg, 0.054 mmol, 54%) as a white solid.

$\mathbf{R}_f$ 0.32 (5% Et_2O/hexane); **^{1}H NMR** (600 MHz, $CDCl_3$) δ 7.37 (d, J = 6.6 Hz, 2H, ArH), 7.13 (d, J = 6.8 Hz, 2H, ArH), 1.60 (s, 6H, H3, H4 and H5), 1.22 (s, 6H, H16 and H17), 1.20 (s, 12H, H11, H12, H13 and H14); **^{13}C NMR** (151 MHz, $CDCl_3$) δ 145.7, 130.7, 128.4, 119.4, 83.4, 53.2, 48.1, 38.1, 24.8, 24.1; **^{11}B NMR** (192 MHz, $CDCl_3$) δ 31.0; **HRMS** (ESI^+) calc. for $C_{20}H_{28}BBrO_2$ $[M+H]^+$391.1442, found 391.1435.

4,4,5,5-tetramethyl-2-(3-(1-phenylcyclopropyl)bicyclo[1.1.1]pentan-1-yl)-1,3,2-dioxaborolane (3ah)

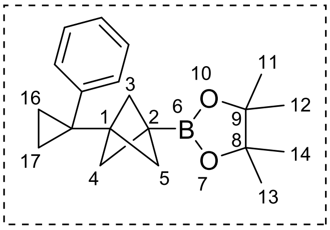

Synthesized according to *General Procedure* using carboxylic acid **2ah** (32.4 mg, 0.2 mmol, 2.0 equiv.), [1.1.1]propellane (0.11 mL, 0.88 M in Et_2O solution, 0.1 mmol, 1.0 equiv.), bis(pinacolato)diboron (76.2 mg, 0.3 mmol, 3.0 equiv.), Cs_2CO_3 (6.5 mg, 0.02 mmol, 0.2 equiv.) and $Fe(NO_3)_3{\cdot}9H_2O$ (4.0 mg, 0.01 mmol, 0.1 equiv.). Purification is through flash column chromatography (0-10% Et_2O/Hexane) to give product **3ah** (16.7 mg, 0.054 mmol, 54%) as a white solid.

$\mathbf{R}_f$ 0.33 (5% Et_2O/hexane); **^{1}H NMR** (600 MHz, $CDCl_3$) δ 7.25 – 7.21 (m, 4H, ArH), 7.18-7.13 (m, 1H), 1.60 (s, 6H, H3, H4 and H5), 1.19 (s, 12H, H11, H12, H13 and H14), 0.73 – 0.70 (m, 2H, H16), 0.67 – 0.64 (m, 2H, H17); **^{13}C NMR** (151 MHz, $CDCl_3$) δ 143.2, 130.1, 128.0, 126.1, 83.3, 49.8, 49.6, 27.9, 24.8, 9.4; **^{11}B NMR** (192 MHz, $CDCl_3$) δ 31.1; **HRMS** (ESI^+) calc. for $C_{20}H_{27}BO_2$ $[M+H]^+$311.2181, found 311.2176.

2-(3-(1-(4-bromophenyl)cyclopropyl)bicyclo[1.1.1]pentan-1-yl)-4,4,5,5-tetramethyl-1,3,2-dioxaborolane (3ai)

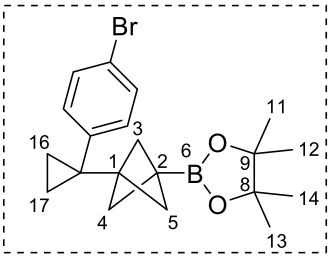

Synthesized according to *General Procedure* using carboxylic acid **2ai** (48.2 mg, 0.2 mmol, 2.0 equiv.), [1.1.1]propellane (0.11 mL, 0.88 M in Et_2O solution, 0.1 mmol, 1.0 equiv.), bis(pinacolato)diboron (76.2 mg, 0.3 mmol, 3.0 equiv.), Cs_2CO_3 (6.5 mg, 0.02 mmol, 0.2 equiv.) and $Fe(NO_3)_3·9H_2O$ (4.0 mg, 0.01 mmol, 0.1 equiv.). Purification is through flash column chromatography (0-10% Et_2O/Hexane) to give product **3ai** (27.6 mg, 0.071 mmol, 71%) as a white solid.

R*f* 0.38 (6% Et_2O/hexane); **^{1}H NMR** (600 MHz, $CDCl_3$) δ 7.36 (d, *J* = 6.8 Hz, 2H, ArH), 7.11 (d, *J* = 6.8 Hz, 2H, ArH), 1.59 (s, 6H, H3, H4 and H5), 1.19 (s, 12H, H11, H12, H13 and H14), 0.74-0.70 (m, 2H, H16), 0.64-0.60 (m, 2H, H17); **^{13}C NMR** (151 MHz, $CDCl_3$) δ 142.3, 131.8, 131.1, 120.0, 83.4, 49.6, 49.4, 27.5, 24.8, 9.5; **^{11}B NMR** (192 MHz, $CDCl_3$) δ 30.7; **HRMS** (ESI^+) calc. for $C_{20}H_{26}BBrO_2$ $[M+H]^+$389.1286, found 389.1280.

2-(3-(1-(4-methoxyphenyl)cyclobutyl)bicyclo[1.1.1]pentan-1-yl)-4,4,5,5-tetramethyl-1,3,2-dioxaborolane (3aj)

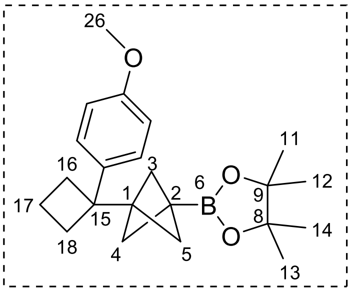

Synthesized according to *General Procedure* using carboxylic acid **2aj** (41.2 mg, 0.2 mmol, 2.0 equiv.), [1.1.1]propellane (0.11 mL, 0.88 M in Et_2O solution, 0.1 mmol, 1.0 equiv.), bis(pinacolato)diboron (76.2 mg, 0.3 mmol, 3.0 equiv.), Cs_2CO_3 (6.5 mg, 0.02 mmol, 0.2 equiv.) and $Fe(NO_3)_3·9H_2O$ (4.0 mg, 0.01 mmol, 0.1 equiv.). Purification is through flash column chromatography (0-10% Et_2O/Hexane) to give product **3aj** (28.3 mg, 0.080 mmol, 80%) as a white solid.

R*f* 0.28 (5% Et_2O/hexane); **^{1}H NMR** (600 MHz, $CDCl_3$) δ 6.87 (d, *J* = 8.7 Hz, 2H, ArH), 6.79 (d, *J* = 8.6 Hz, 2H, ArH), 3.77 (s, 3H, H26), 2.30 – 2.16 (m, 4H, H16 and H18), 1.93 – 1.84 (m, 1H, H17), 1.81 – 1.73 (m, 1H, H17'), 1.65 (s, 6H, H3, H4 and H5), 1.20 (s, 12H, H11, H12, H13 and H14); **^{13}C NMR** (151 MHz, $CDCl_3$) δ 157.3, 140.3, 126.9, 113.1, 83.3, 55.4, 51.9, 51.6, 47.7, 46.3, 29.4, 24.8, 16.2; **^{11}B NMR** (192 MHz, $CDCl_3$) δ 31.0; **HRMS** (ESI^+) calc. for $C_{22}H_{31}BO_3$ $[M+H]^+$355.2443, found 355.2433.

4,4,5,5-tetramethyl-2-(3-(1-phenylcyclopentyl)bicyclo[1.1.1]pentan-1-yl)-1,3,2-dioxaborolane (3ak)

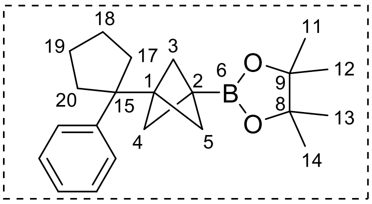

Synthesized according to *General Procedure* using carboxylic acid **2ak** (38.0 mg, 0.2 mmol, 2.0 equiv.), [1.1.1]propellane (0.11 mL, 0.88 M in Et_2O solution, 0.1 mmol, 1.0 equiv.), bis(pinacolato)diboron (76.2 mg, 0.3 mmol, 3.0 equiv.), Cs_2CO_3 (6.5 mg, 0.02 mmol, 0.2 equiv.) and $Fe(NO_3)_3{\cdot}9H_2O$ (4.0 mg, 0.01 mmol, 0.1 equiv.). Purification is through flash column chromatography (0-10% Et_2O/Hexane) to give product **3ak** (10.1 mg, 0.030 mmol, 30%) as a white solid.

R_f 0.39 (5% Et_2O/hexane); **^{1}H NMR** (600 MHz, $CDCl_3$) δ 7.29-7.25 (m, 2H, ArH), 7.22 – 7.18 (m, 2H, ArH), 7.15 – 7.11 (m, 1H, ArH), 2.20-2.14 (m, 2H, H20), 1.55-1.48 (m, 8H, H3, H4, H5 and H17), 1.40 – 1.32 (m, 2H, H19), 1.25 – 1.19 (m, 2H, H18), 1.17 (s, 12H, H11, H12, H13 and H14); **^{13}C NMR** (151 MHz, $CDCl_3$) δ 142.5, 128.0, 127.7, 125.1, 83.3, 53.7, 47.7, 42.5, 31.2, 26.7, 24.8, 22.3; **^{11}B NMR** (192 MHz, $CDCl_3$) δ 31.2; **HRMS** (ESI$^+$) calc. for $C_{22}H_{31}BO_2$ $[M+H]^+$339.2494, found 339.2494.

2-(3-(5-(2,5-dimethylphenoxy)-2-methylpentan-2-yl)bicyclo[1.1.1]pentan-1-yl)-4,4,5,5-tetramethyl-1,3,2-dioxaborolane (3al)

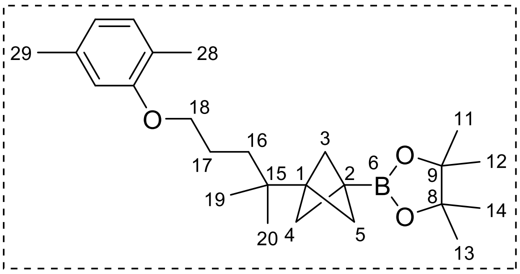

Synthesized according to *General Procedure* using carboxylic acid **2al** (50.0 mg, 0.2 mmol, 2.0 equiv.), [1.1.1]propellane (0.11 mL, 0.88 M in Et_2O solution, 0.1 mmol, 1.0 equiv.), bis(pinacolato)diboron (76.2 mg, 0.3 mmol, 3.0 equiv.), Cs_2CO_3 (6.5 mg, 0.02 mmol, 0.2 equiv.) and $Fe(NO_3)_3{\cdot}9H_2O$ (4.0 mg, 0.01 mmol, 0.1 equiv.). Purification is through flash column chromatography (0-10% Et_2O/Hexane) to give product **3al** (32.6 mg, 0.082 mmol, 82%) as a white solid.

R_f 0.32 (5% Et_2O/hexane); **^{1}H NMR** (600 MHz, $CDCl_3$) δ 7.01 (d, J = 5.1 Hz, 1H, ArH), 6.66 (d, J = 8.9 Hz, 1H, ArH), 6.63 (s, 1H, ArH), 3.90 (t, J = 6.7 Hz, 2H, H18), 2.32 (s, 3H, H29), 2.19 (s, 3H, H28), 1.79 – 1.70 (m, 8H, H3, H4, H5 and H17), 1.34-1.29 (m, 2H, H16), 1.25 (s, 12H, H11, H12, H13 and H14), 0.80 (s, 6H, H19 and H20); **^{13}C NMR** (151 MHz, $CDCl_3$) δ 157.3, 136.5, 130.4, 123.8, 120.7, 112.2, 83.3, 68.9, 54.1, 48.0, 34.8, 32.4, 29.8, 24.9, 24.8, 22.6, 21.5, 15.9; **^{11}B NMR** (192 MHz, $CDCl_3$) δ 31.0; **HRMS** (ESI$^+$) calc. for $C_{25}H_{39}BO_3$ $[M+H]^+$421.2889, found 421.2882.

2-(3-((1R,4aR,4bR,10aR)-7-isopropyl-1,4a-dimethyl-1,2,3,4,4a,4b,5,6,10,10a-decahydrophenanthren-1-yl)bicyclo[1.1.1]pentan-1-yl)-4,4,5,5-tetramethyl-1,3,2-dioxaborolane (3am)

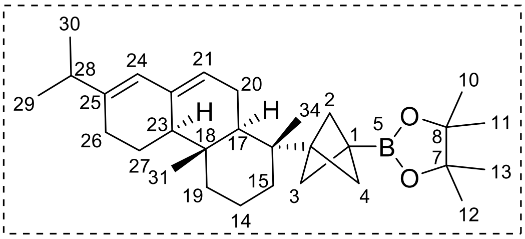

Synthesized according to *General Procedure* using abietic acid **2am** (60.5 mg, 0.2 mmol, 2.0 equiv.), [1.1.1]propellane (0.11 mL, 0.88 M in Et_2O solution, 0.1 mmol, 1.0 equiv.), bis(pinacolato)diboron (76.2 mg, 0.3 mmol, 3.0 equiv.), Cs_2CO_3 (6.5 mg, 0.02 mmol, 0.2 equiv.) and $Fe(NO_3)_3{\cdot}9H_2O$ (4.0 mg, 0.01 mmol, 0.1 equiv.). Purification is through flash column chromatography (0-10% Et_2O/Hexane) to give product **3am** (28.4 mg, 0.063 mmol, 63%) as a white solid.

R*f* 0.48 (5% Et_2O/hexane); **^{1}H NMR** (600 MHz, $CDCl_3$) δ 5.77 (s, 1H, H24), 5.36 (s, 1H, H21), 2.24-2.16 (m, 2H, H28 and H26), 2.09-2.04 (m, 2H, H26' and H20), 1.97 – 1.76 (m, 5H, H17, H20', H23 and H27), 1.73 (s, 6H, H2, H3 and H4), 1.51 – 1.42 (m, 2H, H15 and H19), 1.38 – 1.34 (m, 1H, H14), 1.22 (s, 12H, H10, H11, H12 and H13), 1.19 – 1.11 (m, 3H, H14', H15' and H19'), 1.01 (ddd, J = 6.7, 4.2, 1.6 Hz, 6H, H29 and H30), 0.84 (s, 3H, H34), 0.78 (s, 3H, H31); **^{13}C NMR** (151 MHz, $CDCl_3$) δ 145.0, 135.3, 122.7, 121.9, 83.3, 55.8, 50.7, 49.1, 45.9, 39.1, 36.4, 35.0, 35.0, 34.9, 27.6, 25.7, 24.9, 22.8, 21.5, 21.0, 18.5, 16.3, 14.3; **^{11}B NMR** (192 MHz, $CDCl_3$) δ 31.1; **HRMS** (ESI$^+$) calc. for $C_{30}H_{47}BO_2$ $[M+H]^+$451.3747, found 451.3741.

2-(3-((1R,4aS,10aS)-7-isopropyl-1,4a-dimethyl-1,2,3,4,4a,9,10,10a-octahydrophenanthren-1-yl)bicyclo[1.1.1]pentan-1-yl)-4,4,5,5-tetramethyl-1,3,2-dioxaborolane (3an)

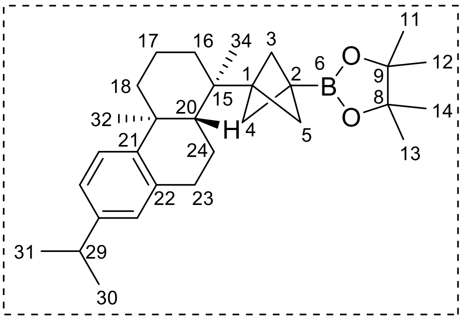

Synthesized according to *General Procedure* using dehydroabietic acid **2am** (60.1 mg, 0.2 mmol, 2.0 equiv.), [1.1.1]propellane (0.11 mL, 0.88 M in Et_2O solution, 0.1 mmol, 1.0 equiv.), bis(pinacolato)diboron (76.2 mg, 0.3 mmol, 3.0 equiv.), Cs_2CO_3 (6.5 mg, 0.02 mmol, 0.2 equiv.) and $Fe(NO_3)_3{\cdot}9H_2O$ (4.0 mg, 0.01 mmol, 0.1 equiv.). Purification is through flash column chromatography (0-10% Et_2O/Hexane) to give product **3am** (33.2 mg, 0.074 mmol, 74%) as a colorless oil.

R*f* 0.42 (6% Et_2O/hexane); **^{1}H NMR** (600 MHz, $CDCl_3$) δ 7.16 (d, *J* = 8.1 Hz, 1H, ArH), 6.98 (d, *J* = 8.1 Hz, 1H, ArH), 6.89 (s, 1H, ArH), 2.89-2.78 (m, 3H, H29 and H23), 2.22 (dt, *J* = 12.7, 3.5 Hz, 1H, H18), 1.98 (ddt, *J* = 10.9, 6.8, 2.2 Hz, 1H, H24), 1.82 – 1.76 (m, 6H, H3, H4 and H5), 1.74 – 1.58 (m, 3H, H18', H24' and H17), 1.50 (dd, *J* = 12.3, 2.0 Hz, 1H, H20), 1.36 – 1.30 (m, 1H, H16), 1.25-1.21 (m, 18H, H11, H12, H13, H14, H30 and H31), 1.21-1.18 (m, 2H, H17' and H16'), 1.18 (s, 3H, H32), 0.87 (s, 3H, H34); **^{13}C NMR** (151 MHz, $CDCl_3$) δ 147.8, 145.6, 135.2, 127.1, 124.4, 123.8, 83.4, 56.0, 49.3, 46.2, 38.6, 37.7, 35.6, 35.2, 33.6, 30.3, 25.7, 24.9, 24.1, 24.1, 20.6, 19.0, 16.1; **^{11}B NMR** (192 MHz, $CDCl_3$) δ 31.2; **HRMS** (ESI$^+$) calc. for $C_{30}H_{45}BO_2$ $[M+H]^+$ 449.3591, found 449.3584.

2-phenyl-2-(3-(4,4,5,5-tetramethyl-1,3,2-dioxaborolan-2-yl)bicyclo[1.1.1]pentan-1-yl)ethan-1-ol (3ao)

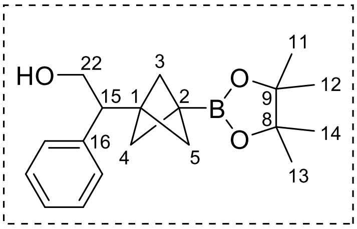

Synthesized according to *General Procedure* using carboxylic acid **2ao** (33.2 mg, 0.2 mmol, 2.0 equiv.), [1.1.1]propellane (0.11 mL, 0.88 M in Et_2O solution, 0.1 mmol, 1.0 equiv.), bis(pinacolato)diboron (76.2 mg, 0.3 mmol, 3.0 equiv.), Cs_2CO_3 (6.5 mg, 0.02 mmol, 0.2 equiv.) and $Fe(NO_3)_3{\cdot}9H_2O$ (4.0 mg, 0.01 mmol, 0.1 equiv.). Purification is through flash column chromatography (0-30% EtOAc/Hexane) to give product **3am** (21.7 mg, 0.069 mmol, 69%) as a white solid.
$\mathbf{R}_{\mathbf{f}}$ 0.39 (30% EtOAc/hexane); ^{1}**H NMR** (600 MHz, $CDCl_3$) δ 7.33 – 7.27 (m, 2H, ArH), 7.24 – 7.19 (m, 1H, ArH), 7.16 – 7.10 (m, 2H, ArH), 3.92 – 3.80 (m, 2H, H22), 2.85 (dd, J = 9.4, 5.8 Hz, 1H, H15), 1.79 – 1.68 (m, 6H, H3, H4 and H5), 1.19 (s, 12H, H11, H12, H13 and H14); ^{13}**C NMR** (151 MHz, $CDCl_3$) δ 139.7, 128.6, 128.4, 126.8, 83.4, 64.1, 51.1, 50.7, 46.6, 24.8; ^{11}**B NMR** (192 MHz, $CDCl_3$) δ 30.6; **HRMS** (ESI^+) calc. for $C_{19}H_{27}BO_3$ $[M+Na]^+$337.1949, found 337.1963.

2-phenyl-2-(3-(4,4,5,5-tetramethyl-1,3,2-dioxaborolan-2-yl)bicyclo[1.1.1]pentan-1-yl)acetaldehyde (3ap)

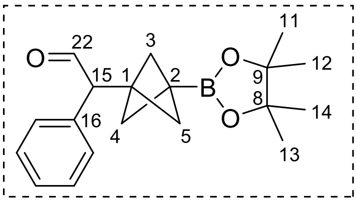

Synthesized according to *General Procedure* using carboxylic acid **2ao** (33.2 mg, 0.2 mmol, 2.0 equiv.), [1.1.1]propellane (0.11 mL, 0.88 M in Et_2O solution, 0.1 mmol, 1.0 equiv.), bis(pinacolato)diboron (76.2 mg, 0.3 mmol, 3.0 equiv.), Cs_2CO_3 (6.5 mg, 0.02 mmol, 0.2 equiv.) and $Fe(NO_3)_3{\cdot}9H_2O$ (4.0 mg, 0.01 mmol, 0.1 equiv.).To the crude residue in EtOAc (1.0 mL) was added IBX (84 mg, 0.3 mmol, 3.0 equiv.). After stirring at 80 °C for 2 h, the reaction mixture was filtered off and concentrated under reduced pressure. Purification is through flash column chromatography (0-10% EtOAc/Hexane) to give product **3ap** (14.7 mg, 0.047 mmol, 47% over two steps) as a white solid.
$\mathbf{R}_{\mathbf{f}}$ 0.50 (20% EtOAc/hexane); ^{1}**H NMR** (600 MHz, $CDCl_3$) δ 9.80 (d, J = 2.5 Hz, 1H, H22), 7.36 – 7.32 (m, 2H, ArH), 7.29 – 7.26 (m, 1H, ArH), 7.17 – 7.11 (m, 2H, ArH), 3.54 (d, J = 2.5 Hz, 1H, H15), 1.93 – 1.82 (m, 6H, H3, H4 and H5), 1.20 (s, 12H, H11, H12, H13 and H14); ^{13}**C NMR** (151 MHz, $CDCl_3$) δ 200.7, 135.0, 129.1, 128.8, 127.4, 83.5, 61.7, 51.4, 44.7, 24.8; ^{11}**B NMR** (192 MHz, $CDCl_3$) δ 30.5; **HRMS** (ESI^+) calc. for $C_{19}H_{25}BO_3$ $[M+H]^+$313.1973, found 313.1976.

***tert*-butyl (*S*)-2-(3-(4,4,5,5-tetramethyl-1,3,2-dioxaborolan-2-yl)bicyclo[1.1.1]pentan-1-yl)pyrrolidine-1-carboxylate (3aq)**

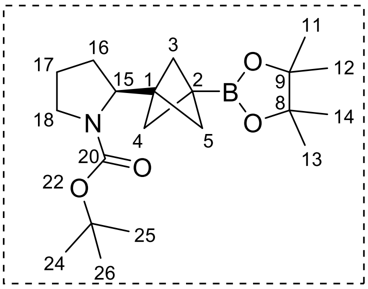

Synthesized according to *General Procedure* using Boc-Pro-OH **2aq** (43.1 mg, 0.2 mmol, 2.0 equiv.), [1.1.1]propellane (0.11 mL, 0.88 M in Et_2O solution, 0.1 mmol, 1.0 equiv.), bis(pinacolato)diboron (76.2 mg, 0.3 mmol, 3.0 equiv.), Cs_2CO_3 (6.5 mg, 0.02 mmol, 0.2 equiv.) and $Fe(NO_3)_3{\cdot}9H_2O$ (4.0 mg, 0.01 mmol, 0.1 equiv.).To the crude residue in EtOAc (1.0 mL) was added IBX (84 mg, 0.3 mmol, 3.0 equiv.). After stirring at 80 °C for 2 h, the reaction mixture was filtered off and concentrated under reduced pressure. Purification is through flash column chromatography (0-15% EtOAc/Hexane) to give product **3aq** (23.6 mg, 0.065 mmol, 65%) as a white solid.

R*f* 0.25 (15% EtOAc/hexane); **^{1}H NMR** (600 MHz, $CDCl_3$) (600 MHz, $CDCl_3$) δ 3.79 – 3.63 (m, 1H, H15), 3.39 – 3.18 (m, 2H, H18), 1.82-1.63 (m, 10H, H3, H4, H5, H16 and H17), 1.45 (s, 9H, H24, H25 and H26), 1.22 (s, 12H, H11, H12, H13 and H14); **^{13}C NMR** (151 MHz, $CDCl_3$) δ 155.2, 83.4, 79.1, 57.7, 50.4, 47.8, 46.3, 28.6, 28.0, 24.9, 23.3; **^{11}B NMR** (192 MHz, $CDCl_3$) δ 30.8; **HRMS** (ESI$^+$) calc. for $C_{20}H_{34}BNO_4$ $[M+Na]^+$386.2477, found 386.2486.

1-(3-(*tert*-butyl)bicyclo[1.1.1]pentan-1-yl)-*N*-methyl-*N*-(naphthalen-2-ylmethyl)methanamine (3ke, bioisostere of butenafine) was synthesized according to the following route

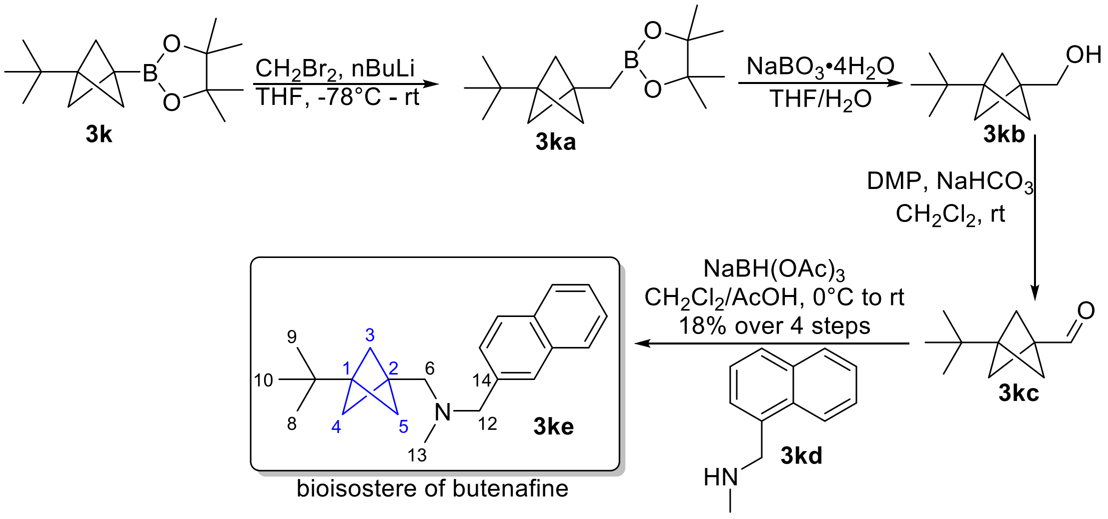

The homologation of boronate was carried out according to modified literature reported procedure.[2-4]

To a solution of **3k** (70 mg, 0.28 mmol, 1.0 equiv.) in THF (2.8 mL) was added CH_2Br_2 (49 µL, 0.70 mmol, 2.5 equiv.) at room temperature under nitrogen atmosphere. The reaction mixture was cooled to -78 °C and nBuLi (0.25 mL, 0.62 mmol, 2.5 M in hexane, 2.2 equiv.) was added dropwise. After stirring at -78 °C for 1 h, the reaction mixture was warmed to 0 °C and stirred for another 30 min. The reaction mixture was further warmed to room temperature. After stirring at room temperature for 1 h, the reaction mixture was quenched with sat. aq. NH_4Cl solution (10 mL) and extracted with EtOAc (3 x 10 mL). The combined organic layers were dried over Na_2SO_4, filtered and concentrated under reduced pressure. The crude residue was used for next step directly without any further purification. (Note, the reaction could not go full conversion although different conditions were tried. The crude residue is the mixture of **3k** and **3ka**)

To a solution of the crude residue in THF and water (2.8 mL, 1:1) was added $NaBO_3 \cdot 4H_2O$ (215 mg, 1.4 mmol, 5.0 equiv.). After stirring at room temperature overnight, the reaction mixture was quenched with water (10 mL) and extracted with Et_2O (3 x 10 mL). The combined organic layers were dried over Na_2SO_4, filtered and concentrated under reduced pressure. The residue was quickly purified by flash column chromatography (0-20% EtOAc/Hexane) to give product **3kb** containing inseparable impurity (33 mg), which was carried out for next step.

To a solution of alcohol **3kb** (33 mg, 0.21 mmol, 1.0 equiv.) in CH_2Cl_2 (2.1 mL) was added sodium bicarbonate (180 mg, 2.1 mmol, 10.0 equiv.), followed by Dess-Martin periodinane (136 mg, 0.32 mmol, 1.5 equiv.). After stirring at room temperature for 1h, the reaction mixture was quenched with sat. aq. $NaHCO_3$ solution (10 mL) and sat. aq. $Na_2S_2O_3$ solution (10 mL) at 0 °C. The resulting mixture was extracted with CH_2Cl_2 (3

x 10 mL) and the combined organic layers were washed with brine (10 mL), dried over Na_2SO_4, filtered and concentrated under reduced pressure. The crude residue was used for next step without further purification.

To a solution of crude residue and amine **3kd** (36 mg, 0.21 mmol, 1.0 equiv.) in CH_2Cl_2 (1 mL) was added acetic acid (one drop). The reaction was cooled to 0 °C and $NaBH(OAc)_3$ (89 mg, 0.42 mmol, 2.0 equiv.) was added portionwise. After stirring at room temperature for 1 h, the reaction mixture was quenched with sat. aq. $NaHCO_3$ solution (10 mL) and extracted with CH_2Cl_2 (3 x 10 mL). The combined organic layers were dried over Na_2SO_4, filtered and concentrated under reduced pressure. The residue was purified by flash column chromatography (0-20% EtOAc/Hexane) to give product **3ke** (15.5 mg, 0.05 mmol, 18% over four steps) as a colorless oil.
$\mathbf{R_f}$ 0.72 (25% EtOAc/hexane); **^{1}H NMR** (600 MHz, $CDCl_3$) δ 8.35 (d, J = 8.3 Hz, 1H, ArH), 7.85 (d, J = 8.0 Hz, 1H, ArH), 7.77 (d, J = 8.1 Hz, 1H, ArH), 7.53 – 7.46 (m, 3H, ArH), 7.42 (t, J = 7.6 Hz, 1H, ArH), 3.95 (s, 2H, H12), 2.58 (s, 2H, H6), 2.26 (s, 3H, H13), 1.56 (s, 6H, H3, H4 and H5), 0.84 (s, 9H, H8, H9 and H10); **^{13}C NMR** (151 MHz, $CDCl_3$) δ 135.5, 134.0, 132.6, 128.5, 127.7, 127.0, 125.7, 125.6, 125.3, 124.8, 61.2, 59.7, 48.7, 47.5, 43.4, 36.1, 29.6, 26.1; **HRMS** (ESI$^+$) calc. for $C_{22}H_{29}N$ $[M+H]^+$308.2373, found 308.2370.

1-((3-(*tert*-butyl)bicyclo[1.1.1]pentan-1-yl)methyl)-4-((4-chlorophenyl)(phenyl)methyl)piperazine (3kg, bioisostere of butenafine) was synthesized according to the following route

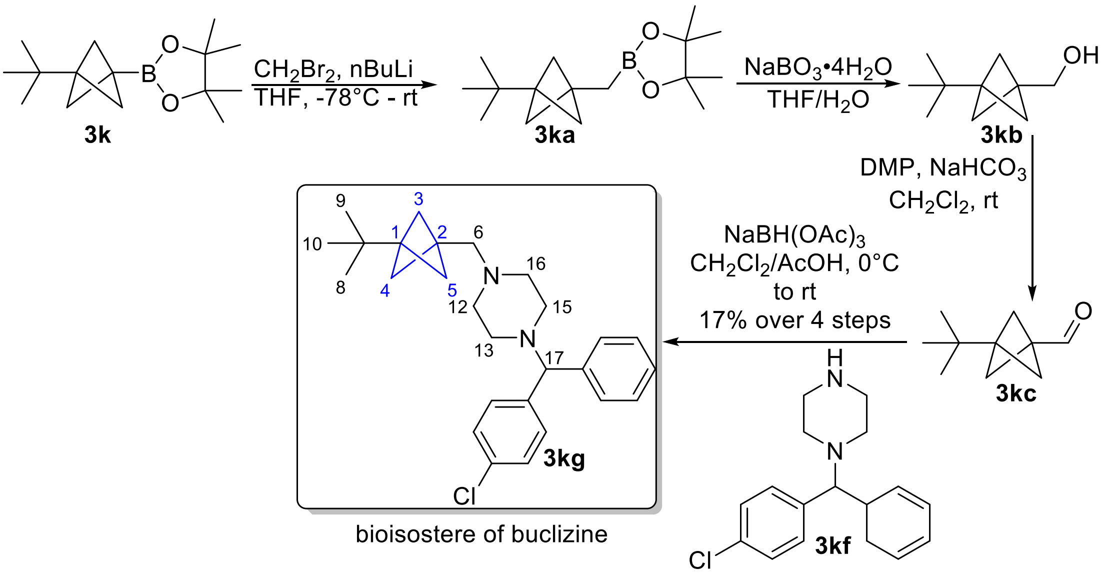

To a solution of **3k** (70 mg, 0.28 mmol, 1.0 equiv.) in THF (2.8 mL) was added CH_2Br_2 (49 µL, 0.70 mmol, 2.5 equiv.) at room temperature under nitrogen atmosphere. The reaction mixture was cooled to -78 °C and nBuLi (0.25 mL, 0.62 mmol, 2.5 M in hexane, 2.2 equiv.) was added dropwise. After stirring at -78 °C for 1 h, the reaction mixture was warmed to 0 °C and stirred for another 30 min. The reaction mixture was further

warmed to room temperature. After stirring at room temperature for 1 h, the reaction mixture was quenched with sat. aq. NH_4Cl solution (10 mL) and extracted with EtOAc (3 x 10 mL). The combined organic layers were dried over Na_2SO_4, filtered and concentrated under reduced pressure. The crude residue was used for next step directly without any further purification. (Note, the reaction could not go full conversion although different conditions were tried. The crude residue is the mixture of **3k** and **3ka**)

To a solution of the crude residue in THF and water (2.8 mL, 1:1) was added $NaBO_3 \cdot 4H_2O$ (215 mg, 1.4 mmol, 5.0 equiv.). After stirring at room temperature overnight, the reaction mixture was quenched with water (10 mL) and extracted with Et_2O (3 x 10 mL). The combined organic layers were dried over Na_2SO_4, filtered and concentrated under reduced pressure. The residue was quickly purified by flash column chromatography (0-20% EtOAc/Hexane) to give product **3kb** containing inseparable impurity (35 mg), which was carried out for next step.

To a solution of alcohol **3kb** (35 mg, 0.23 mmol, 1.0 equiv.) in CH_2Cl_2 (2.1 mL) was added sodium bicarbonate (191 mg, 2.3 mmol, 10.0 equiv.), followed by Dess-Martin periodinane (144 mg, 0.34 mmol, 1.5 equiv.). After stirring at room temperature for 1h, the reaction mixture was quenched with sat. aq. $NaHCO_3$ solution (10 mL) and sat. aq. $Na_2S_2O_3$ solution (10 mL) at 0 °C. The resulting mixture was extracted with CH_2Cl_2 (3 x 10 mL) and the combined organic layers were washed with brine (10 mL), dried over Na_2SO_4, filtered and concentrated under reduced pressure. The crude residue was used for next step without further purification.

To a solution of crude residue and amine **3kf** (65 mg, 0.23 mmol, 1.0 equiv.) in CH_2Cl_2 (1 mL) was added acetic acid (one drop). The reaction was cooled to 0 °C and $NaBH(OAc)_3$ (97 mg, 0.46 mmol, 2.0 equiv.) was added portionwise. After stirring at room temperature for 1 h, the reaction mixture was quenched with sat. aq. $NaHCO_3$ solution (10 mL) and extracted with CH_2Cl_2 (3 x 10 mL). The combined organic layers were dried over Na_2SO_4, filtered and concentrated under reduced pressure. The residue was purified by flash column chromatography (0-20% EtOAc/Hexane) to give product **3kg** (20.1 mg, 0.048 mmol, 17% over four steps) as a colorless oil.
R_f 0.56 (25% EtOAc/hexane); **^{1}H NMR** (600 MHz, $CDCl_3$) δ 7.38 – 7.33 (m, 4H, ArH), 7.28 – 7.21 (m, 4H, ArH), 7.20 – 7.15 (m, 1H, ArH), 4.19 (s, 1H, H17), 2.44 (s, 10H, H6, H12, H13, H15 and H16), 1.49 (s, 6H, H3, H4 and H5), 0.80 (s, 9H, H8, H9 and H10); **^{13}C NMR** (151 MHz, $CDCl_3$) δ 142.4, 141.6, 132.6, 129.4, 128.7, 128.7, 128.0, 127.2, 75.7, 60.3, 54.1, 51.9, 48.8, 47.7, 35.4, 29.6, 26.0; **HRMS** (ESI^+) calc. for $C_{27}H_{35}ClN_2$ $[M+H]^+$ 423.2562, found 423.2578.

3.4. Scalability of the standard reaction.

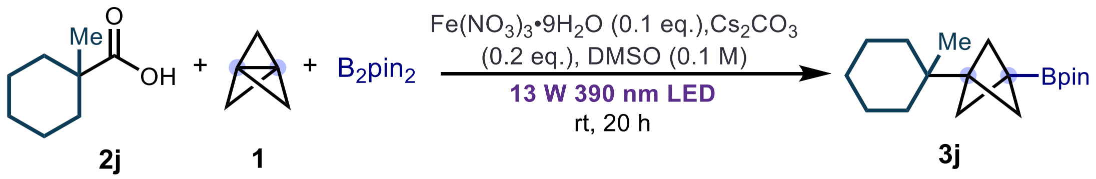

First, reaction was carried out following the general procedure with 0.2 mmol scale. Carboxylic acid **2j** (56.9 mg, 0.4 mmol, 2.0 equiv.), bis(pinacolato)diboron (152.4 mg, 0.6 mmol, 3.0 equiv.), Cs_2CO_3 (13.0 mg, 0.04 mmol, 0.2 equiv.) and $Fe(NO_3)_3{\cdot}9H_2O$ (8.1 mg, 0.02 mmol, 0.1 equiv.) in DMSO (2.0 mL) under N_2 atmosphere was added [1.1.1]propellane **1** (0.23 mL, 0.2 mmol, 0.88 M in Et_2O, 1.0 equiv.). The isolated yield of the product **3j** is (51.1 mg, 0.176 mmol, 88%).

Then reaction was carried out following the general procedure with 1.0 mmol scale while two 390 nm Kessil® lamps (25% intensity, 13 W) and a cooling fan were used. Carboxylic acid **2j** (284.4 mg, 2.0 mmol, 2.0 equiv.), bis(pinacolato)diboron (761.8 mg, 3.0 mmol, 3.0 equiv.), Cs_2CO_3 (65.2 mg, 0.2 mmol, 0.2 equiv.) and $Fe(NO_3)_3{\cdot}9H_2O$ (40.4 mg, 0.1 mmol, 0.1 equiv.) in DMSO (10.0 mL) under N_2 atmosphere was added [1.1.1]propellane **1** (1.1 mL, 1.0 mmol, 0.88 M in Et_2O, 1.0 equiv.). The isolated yield of the product **3j** is (246.7 mg, 0.85 mmol, 85%). The reaction setting up is as followings:

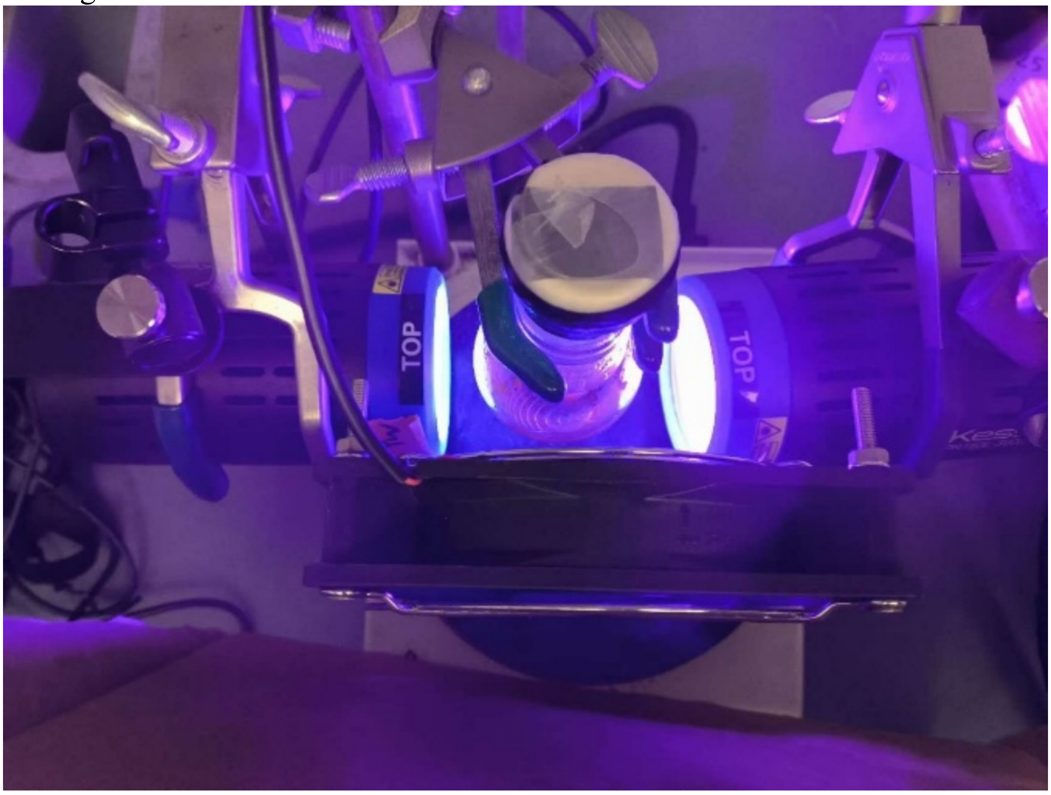

3.5. Mechanism study.

HRMS study

Before conducting the following experiments, we submitted the commercial DMSO (>=99.9% ACS, VWR Chemicals BDH) that was used for our reactions for HRMS analysis and no residue DMS was found in the DMSO by HRMS.

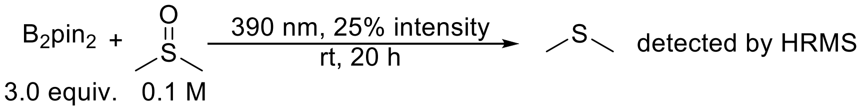

After irradiating the mixture of B_2pin_2 (76.2 mg, 0.3 mmol, 3.0 equiv.) in DMSO (1 mL) under 390 nm light at room temperature, the reaction mixture was transferred to a vial and diluted with MeOH for HRMS analysis. The desired DMS molecule was found. However, no trace of DMS was found without light irradiation.

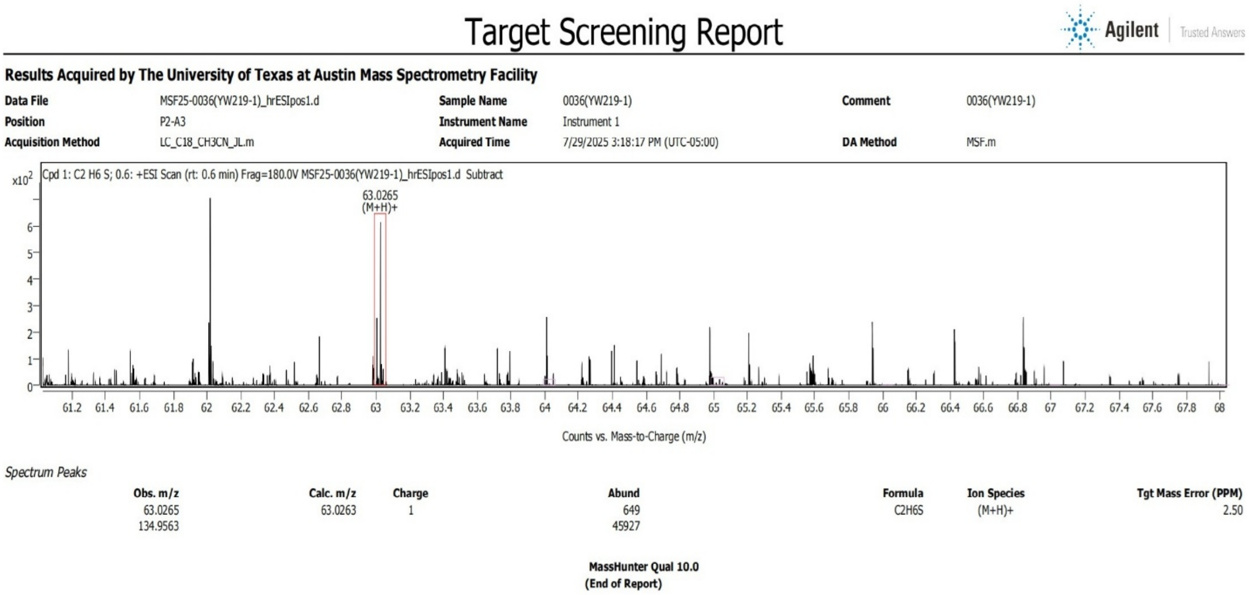

HRMS (ESI^+) calc. for C_2H_6S $[M+H]^+$63.0263, found 63.0265.

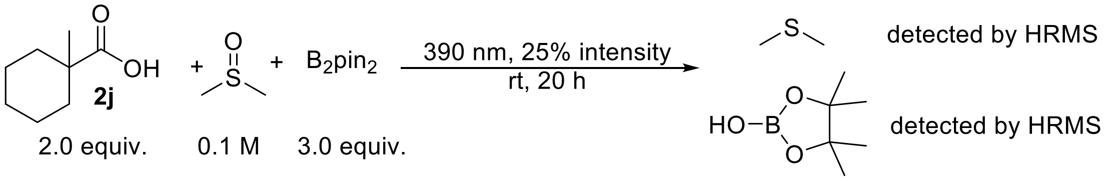

After irradiating the mixture of B_2pin_2 (76.2 mg, 0.3 mmol, 3.0 equiv.) and carboxylic acid (28.4 mg, 0.2 mmol, 2.0 equiv.) in DMSO (1 mL) under 390 nm light at room temperature, the reaction mixture was transferred to a vial and diluted with MeOH for HRMS analysis. The desired DMS and HOBpin molecule was found.

Target Screening Report

Results Acquired by The University of Texas at Austin Mass Spectrometry Facility

**Data File**	MSF25-0036(YW219-2)_hrESIpos1.d	**Sample Name**	0036(YW219-2)	**Comment**	0036(YW219-2)
**Position**	P2-A4	**Instrument Name**	Instrument 1		
**Acquisition Method**	LC_C18_CH3CN_JL.m	**Acquired Time**	7/29/2025 3:40:15 PM (UTC-05:00)	**DA Method**	MSF.m

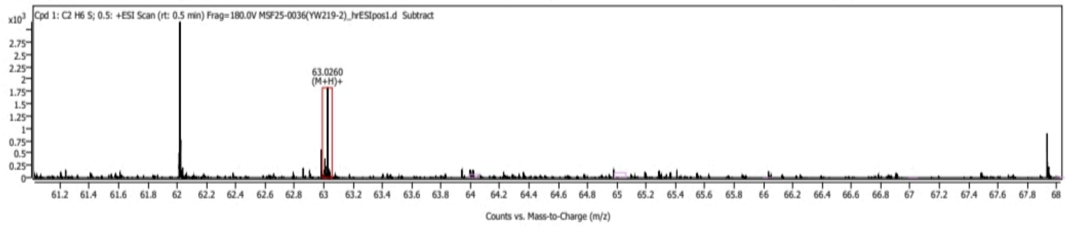

Spectrum Peaks

Obs. m/z	Calc. m/z	Charge	Abund	Formula	Ion Species	Tgt Mass Error (PPM)
63.0260	63.0263	1	1829	C2H6S	(M+H)+	-4.28
116.9095			31156			

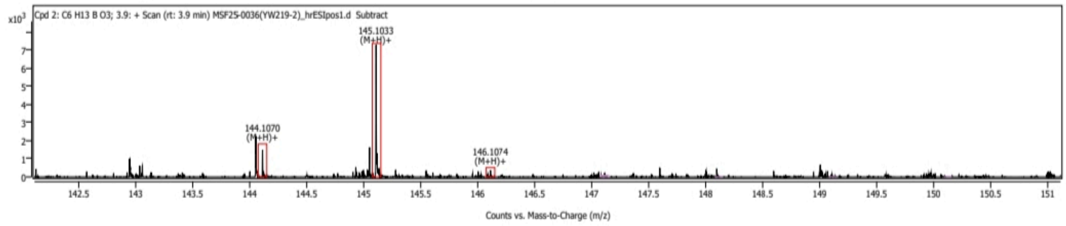

Spectrum Peaks

Obs. m/z	Calc. m/z	Charge	Abund	Formula	Ion Species	Tgt Mass Error (PPM)
144.1070	144.1067	1	1533	C6H13BO3	(M+H)+	1.96
145.1033	145.1032	1	7318	C6H13BO3	(M+H)+	0.93
146.1074	146.1066	1	369	C6H13BO3	(M+H)+	4.94
349.1950			54709			

MassHunter Qual 10.0
(End of Report)

HRMS (ESI^+) calc. for C_2H_6S $[M+H]^+$63.0263, found 63.0260, for $C_6H_{13}BO_3$[M+H]+145.1032, found 145.1033.

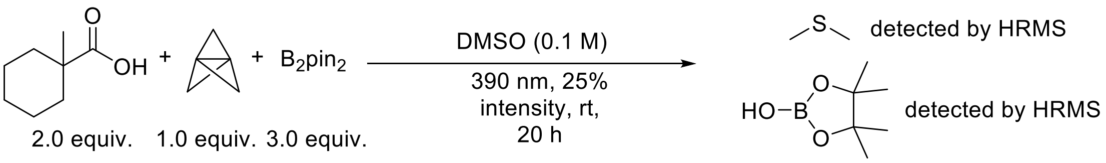

After irradiating the mixture of B_2pin_2 (76.2 mg, 0.3 mmol, 3.0 equiv.), carboxylic acid (28.4 mg, 0.2 mmol, 2.0 equiv.) and [1.1.1]propellane (0.11 mL, 0.88 M, 0.1 mmol, 1.0 equiv.) in DMSO (1 mL) under 390 nm light at room temperature, the reaction mixture was transferred to a vial and diluted with MeOH for HRMS analysis. The desired DMS and HOBpin molecule was found.

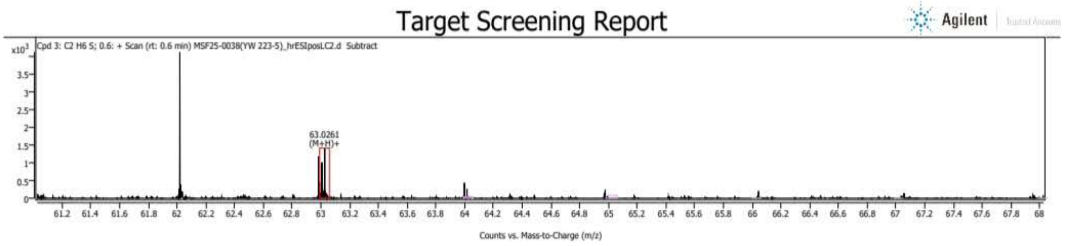

Spectrum Peaks

Obs. m/z	Calc. m/z	Charge	Abund	Formula	Ion Species	Tgt Mass Error (PPM)
63.0261	63.0263	1	1408	C2H6S	(M+H)+	-2.83
168.9171			139717			

MassHunter Qual 10.0
(End of Report)

HRMS (ESI$^+$) calc. for C_2H_6S $[M+H]^+$63.0263, found 63.0261.

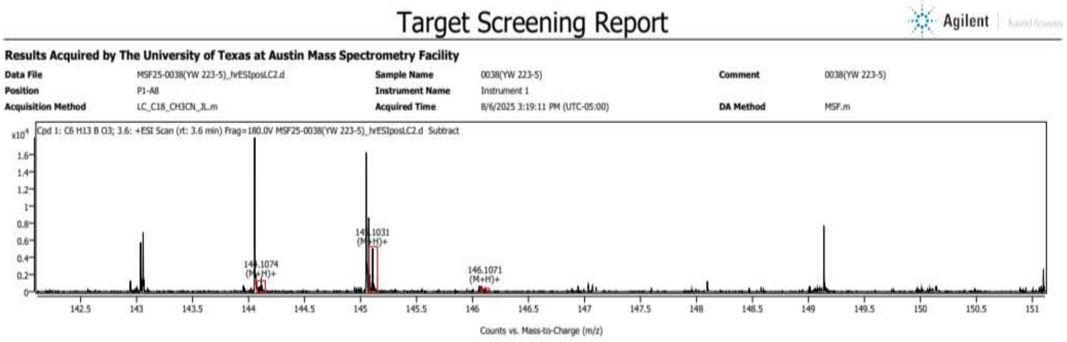

Spectrum Peaks

Obs. m/z	Calc. m/z	Charge	Abund	Formula	Ion Species	Tgt Mass Error (PPM)
144.1074	144.1067	1	1454	C6H13BO3	(M+H)+	4.75
145.1031	145.1032	1	5045	C6H13BO3	(M+H)+	-0.31
146.1071	146.1066	1	288	C6H13BO3	(M+H)+	2.89
348.1988			1117728			

HRMS (ESI$^+$) calc. for $C_6H_{13}BO_3$[M+H]+145.1032, found 145.1031.

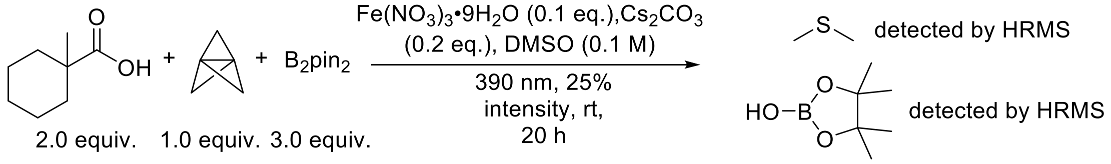

After irradiating the mixture of B_2pin_2 (76.2 mg, 0.3 mmol, 3.0 equiv.), carboxylic acid (28.4 mg, 0.2 mmol, 2.0 equiv.), [1.1.1]propellane (0.11 mL, 0.88 M, 0.1 mmol, 1.0 equiv.). $Fe(NO_3)_3·9H_2O$ (4.0 mg, 0.01 mmol, 0.1 equiv.) and Cs_2CO_3 (6.5 mg, 0.02 mmol, 0.2 equiv.) in DMSO (1 mL) under 390 nm light at room temperature, the reaction mixture was transferred to a vial and diluted with MeOH for HRMS analysis. The desired DMS and HOBpin molecule was found.

Target Compound Screening Report

Results Acquired by The University of Texas at Austin Mass Spectrometry Facility

Data File MSF25-0034(YW 218-2)_hrESIposLC2.d **Sample Name** 0034(YW 218-2) **Comment** 0034(YW 218-2)
Position P1-F6 **Instrument Name** 6530 **User Name**
Acq Method LC_C18_pos_jl.m **Acquired Time** 7/24/2025 8:46:53 AM **DA Method** MSF.m

MS Zoomed Spectrum

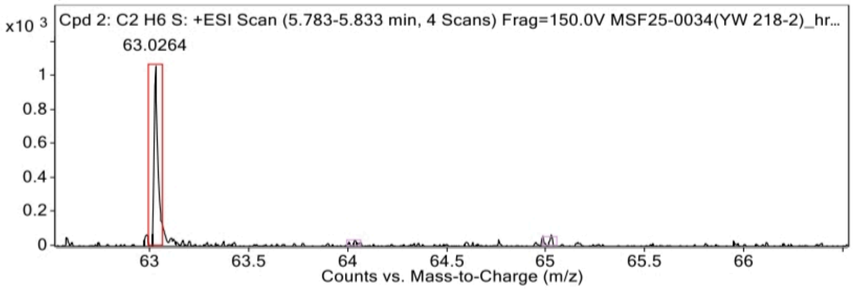

MS Spectrum Peak List

Obs. m/z	Calc. m/z	Charge	Abundance	Formula	Ion Species	Tgt Mass Error (ppm)
63.0264	63.0263	1	1063	C2H6S	(M+H)+	-1.55
266.2115			12719			

--- End Of Report ---

HRMS (ESI+) calc. for C_2H_6S $[M+H]^+$63.0263, found 63.0264.

Target Compound Screening Report

Results Acquired by The University of Texas at Austin Mass Spectrometry Facility

Data File MSF25-0034(YW 218-2)_hrESIposLC2.d **Sample Name** 0034(YW 218-2) **Comment** 0034(YW 218-2)
Position P1-F6 **Instrument Name** 6530 **User Name**
Acq Method LC_C18_pos_jl.m **Acquired Time** 7/24/2025 8:46:53 AM **DA Method** MSF.m

MS Zoomed Spectrum

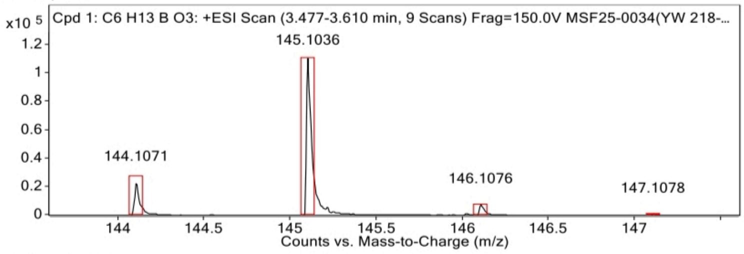

MS Spectrum Peak List

Obs. m/z	Calc. m/z	Charge	Abundance	Formula	Ion Species	Tgt Mass Error (ppm)
144.1071	144.1067	1	23429	C6H13BO3	(M+H)+	-2.66
145.1036	145.1032	1	110915	C6H13BO3	(M+H)+	-2.78
146.1076	146.1066	1	7665	C6H13BO3	(M+H)+	-6.25
147.1078	147.1080	1	1004	C6H13BO3	(M+H)+	1.32
195.0980			182742			

--- End Of Report ---

HRMS (ESI+) calc. for $C_6H_{13}BO_3$[M+H]+145.1032, found 145.1036.

NMR study: The reaction mixture in the vial was diluted with water and diethyl ether was added. After shaking, the organic layer was sucked a bit and transferred to the NMR tube for NMR analysis. Note: DMS is volatile and be careful with the workup.

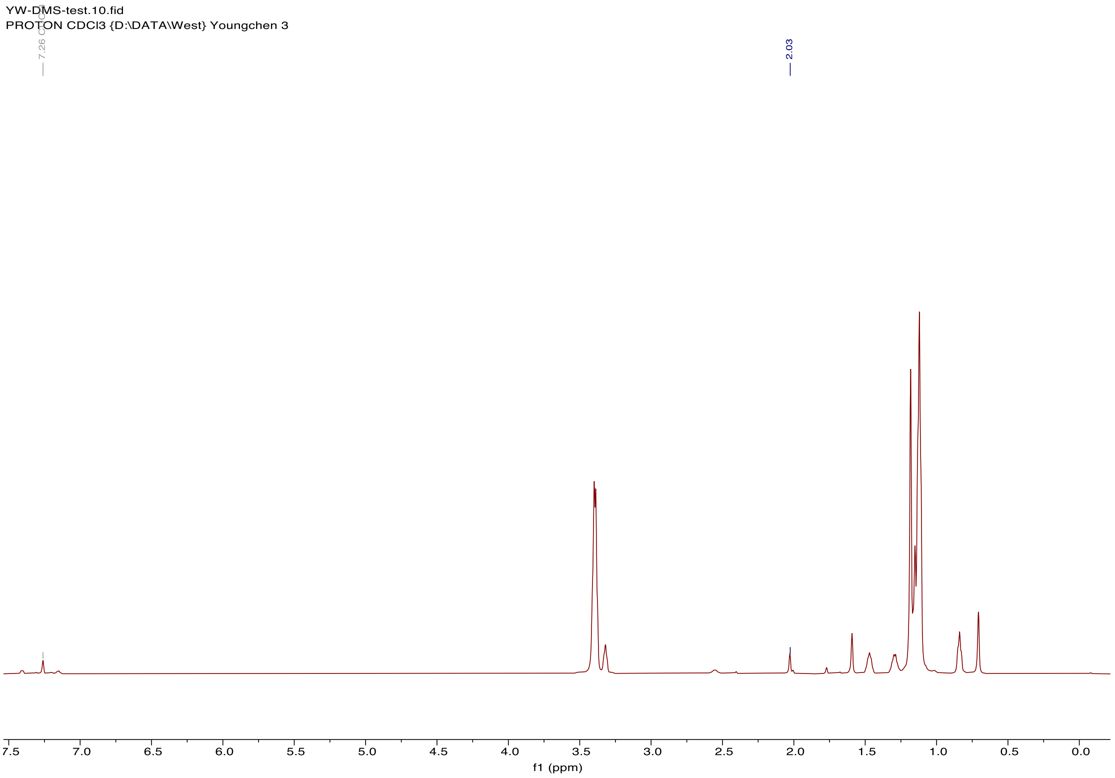

^{1}H NMR (600 MHz, $CDCl_3$) of crude reaction mixture (DMS: 2.03 ppm)

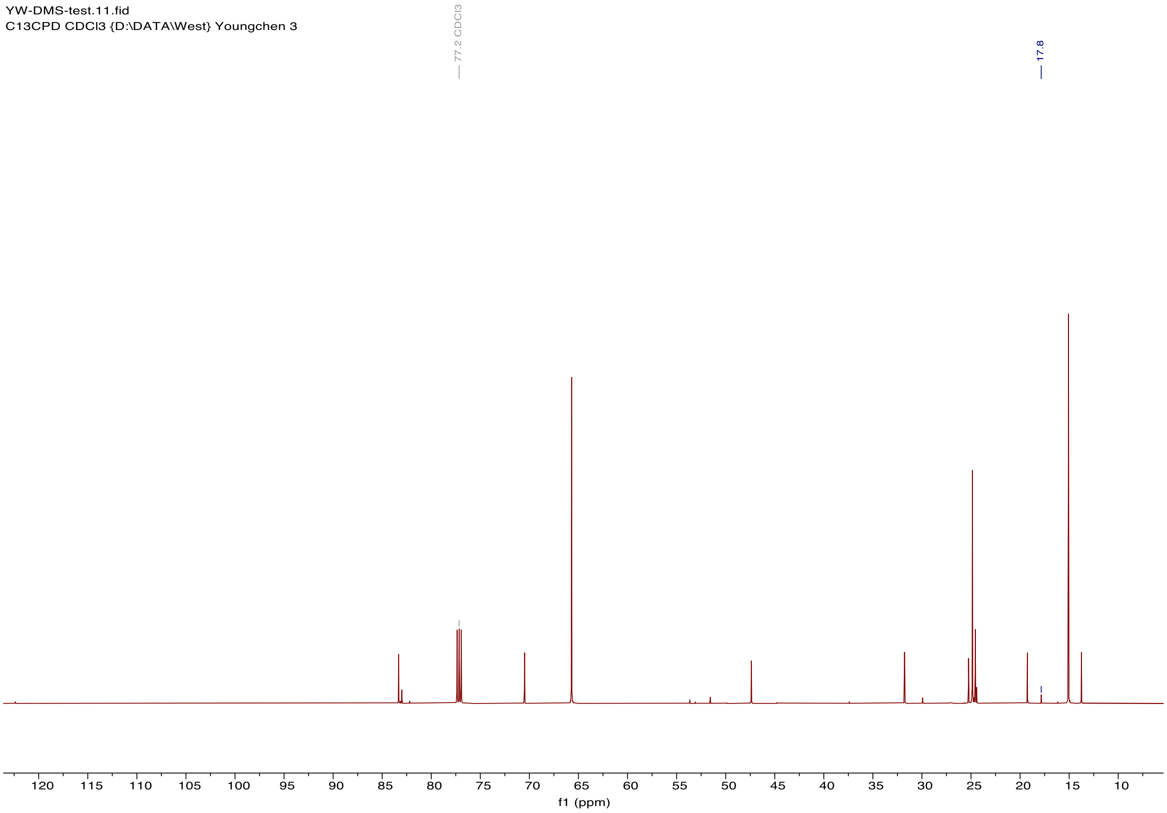

^{13}C NMR (151 MHz, $CDCl_3$) of crude reaction mixture (DMS: 17.8 ppm)

UV-Vis study:

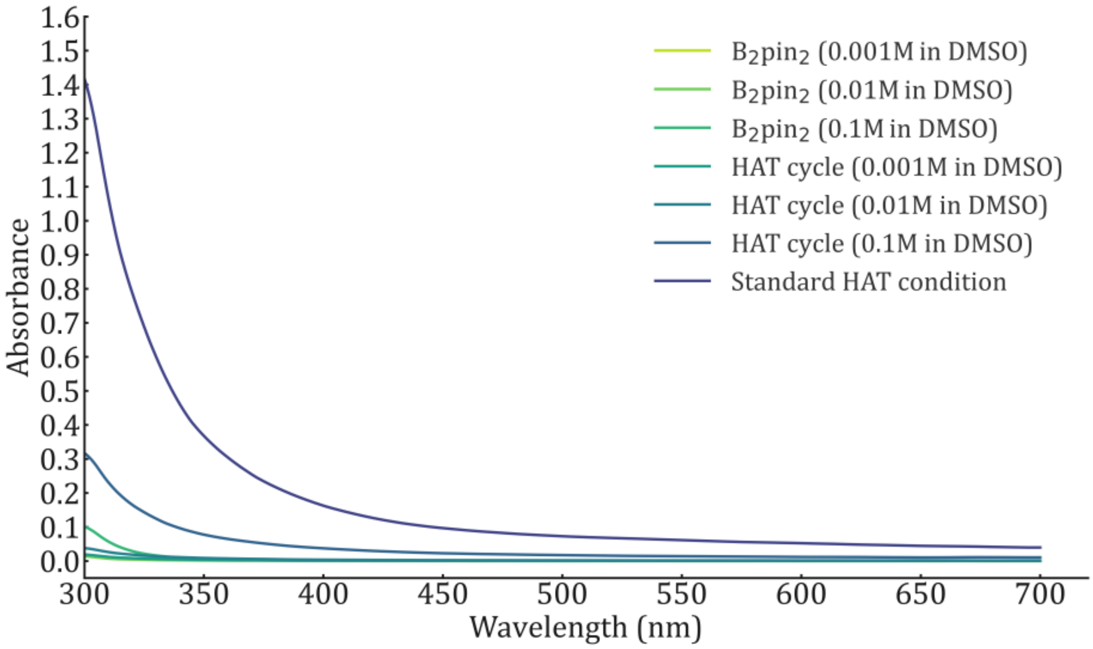

HAT cycle (0.001 M in DMSO) sample preparation: mix 0.5 mL of acid **2j** in DMSO solution (0.001 M) + 0.5 mL of B_2pin_2 in DMSO solution (0.001 M) + 0.5 mL [1.1.1]propellane in DMSO solution (0.001 M) in a reaction vial for UV measurement
HAT cycle (0.01 M in DMSO) sample preparation: mix 0.5 mL of acid **2j** in DMSO solution (0.01 M) + 0.5 mL of B_2pin_2 in DMSO solution (0.01 M) + 0.5 mL [1.1.1]propellane in DMSO solution (0.01 M) in a reaction vial for UV measurement
HAT cycle (0. 1 M in DMSO) sample preparation: mix 0.5 mL of acid **2j** in DMSO solution (0.1 M) + 0.5 mL of B_2pin_2 in DMSO solution (0.1 M) + 0.5 mL [1.1.1]propellane in DMSO solution (0.1 M) in a reaction vial for UV measurement
Standard HAT condition sample preparation: mix acid **2j** (0.2 mmol, 2.0 equiv.) + B_2pin_2 (0.3 mmol, 0.3 equiv.) + [1.1.1]propellane (0.1 mmol, 1.0 equiv.) in DMSO solution (0.1 M) in a reaction vial for UV measurement

DMSO replacement study:

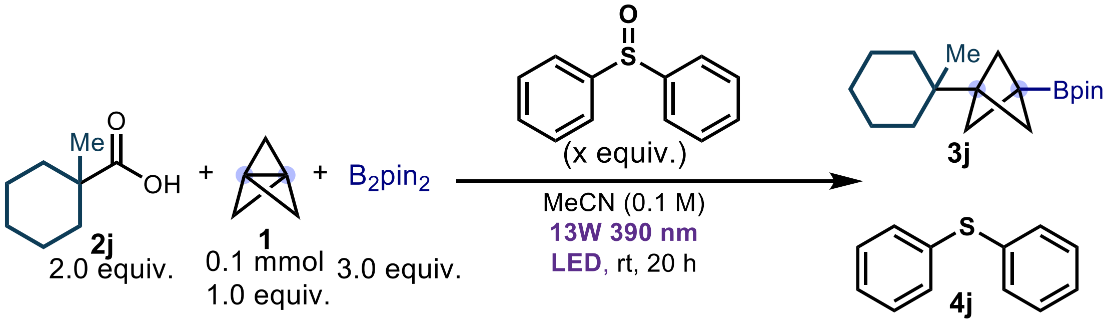

Reactions were carried out following the general procedure with 0.1 mmol scale in the absence of iron and base when DMSO was replaced with different equivalents of solid diphenyl sulfoxide.

Entry	Equivalence of phenyl sulfoxide	solvent	Yield of **3j**	Amount of **4j** formed
1	0	MeCN	0	0 mmol
2	5.0	MeCN	32%	0.053 mmol
3	5.0	DMA	11%	0.033 mmol
4	5.0	DMF	7%	0.019 mmol
5	10.0	MeCN	41%	0.067 mmol
6	15.0	MeCN	46%	0.087 mmol

NMR yield in each entry was obtained by adding 16 μL DCE (0.2 mmol) as internal standard.

Entry 1: no desired product **3j** formed in the absence of sulfoxide

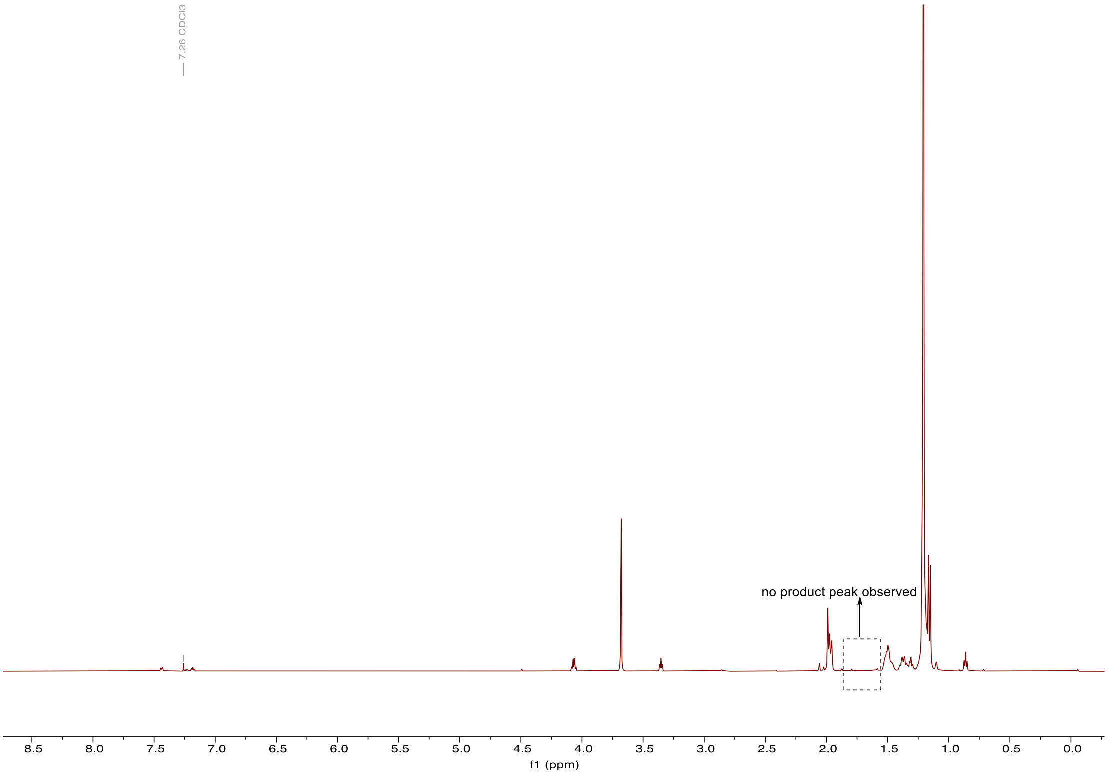

TLC (PMA stain) shows no desired product **3j** observed without addition of phenyl sulfoxide.

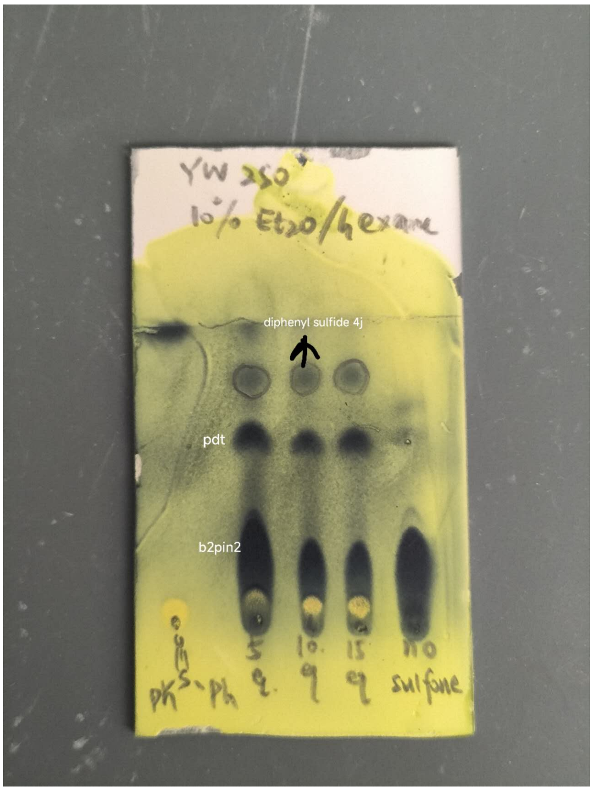

Entry 2: 4.14 (DCE, 4H):1.00 (**3j** BCP, 6H), (4.14/4) : (1/6) =1.035:0.167
0.2 mmol/(1.035/0.167) = 0.032 mmol, yield of **3j**: 0.032 mmol/0.1 mmol = 32%
Amount of **4j** formed:(4.14/4) : (2.75/10) = 1.035:0.275, 0.2 mmol/(1.035/0.275) = 0.053 mmol

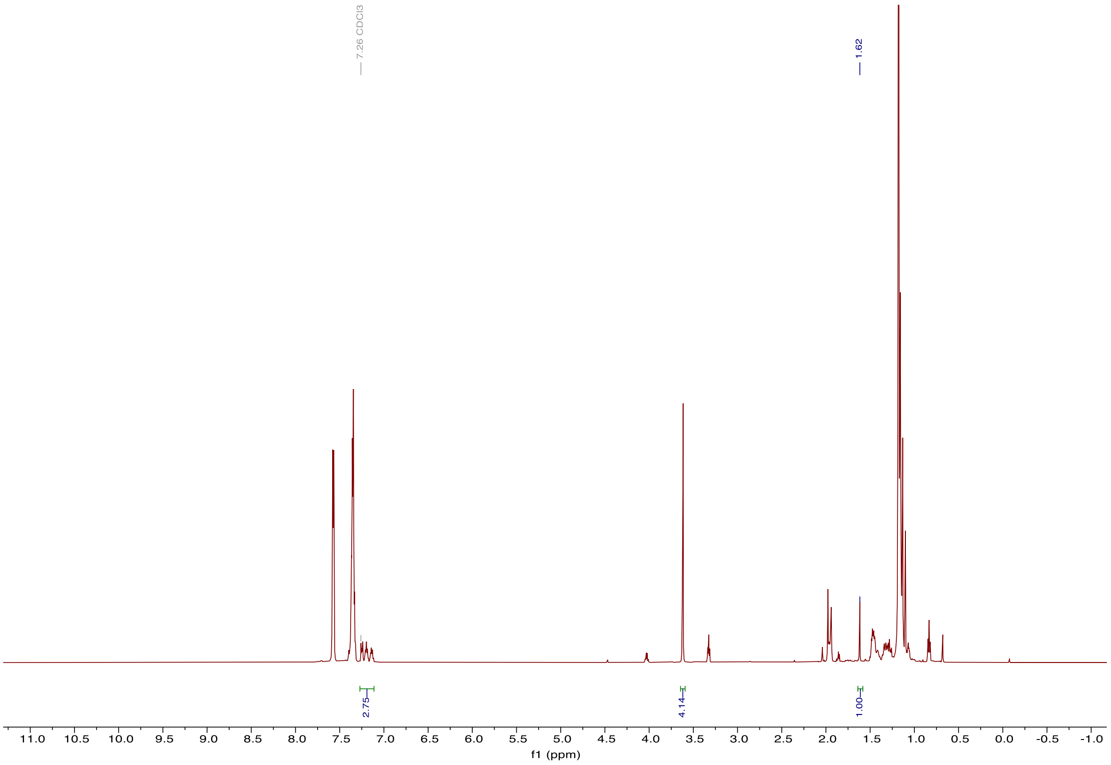

Entry 3: 12.01 (DCE, 4H):1.00 (**3j** BCP, 6H), (12.01/4) : (1/6) =3.0025:0.167
0.2 mmol/(3.0025/0.167) = 0.011 mmol, yield of **3j**: 0.011 mmol/0.1 mmol = 11%
Amount of **4j** formed:(12.01/4) : (5.04/10) = 3.0025:0.504, 0.2 mmol/(3.0025/0.504) = 0.033 mmol

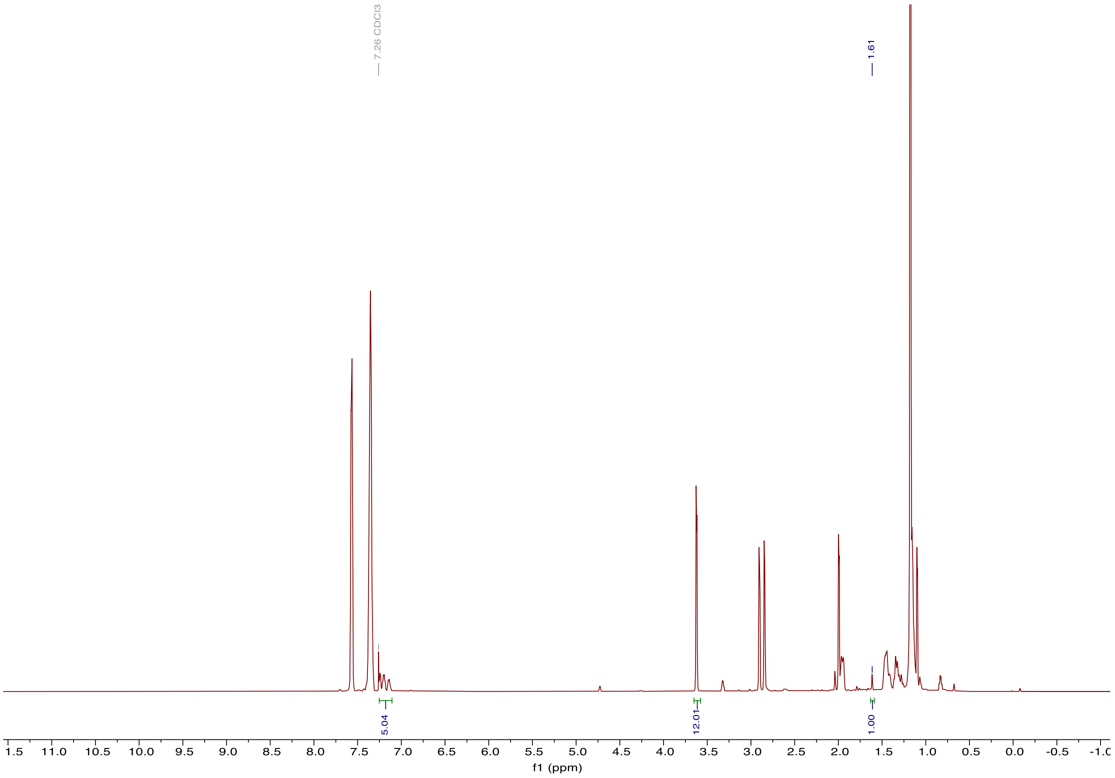

Entry 4: 19.79 (DCE, 4H):1.00 (**3j** BCP, 6H), (19.79/4) : (1/6) =4.9475:0.167
0.2 mmol/(4.9475/0.167) = 0.0068 mmol, yield of **3j**: 0.0068 mmol/0.1 mmol = 7%
Amount of **4j** formed:(19.79/4) : (4.80/10) = 4.9475:0.480, 0.2 mmol/(4.9475/0.480) = 0.019 mmol

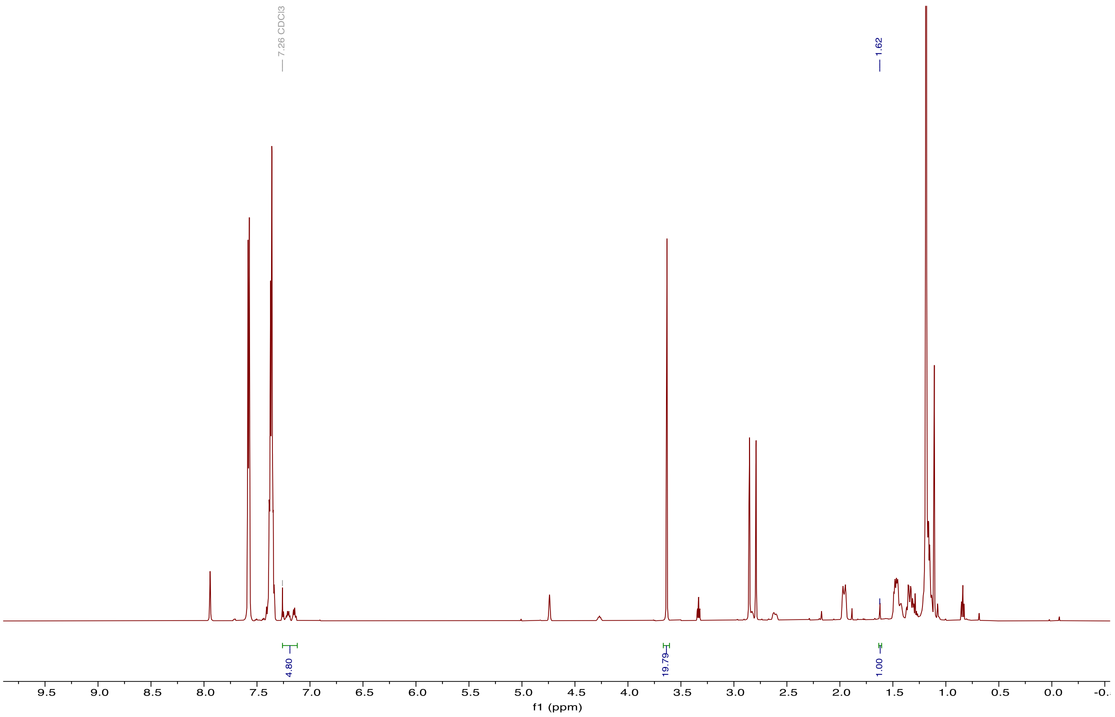

Entry 5: 3.22 (DCE, 4H):1.00 (**3j** BCP, 6H), (3.22/4) : (1/6) =0.805:0.167
0.2 mmol/(0.805/0.167) = 0.041 mmol, yield of **3j**: 0.041 mmol/0.1 mmol = 41%
Amount of **4j** formed:(3.22/4) : (2.68/10) = 0.805:0.268, 0.2 mmol/(0.805/0.268) = 0.067 mmol

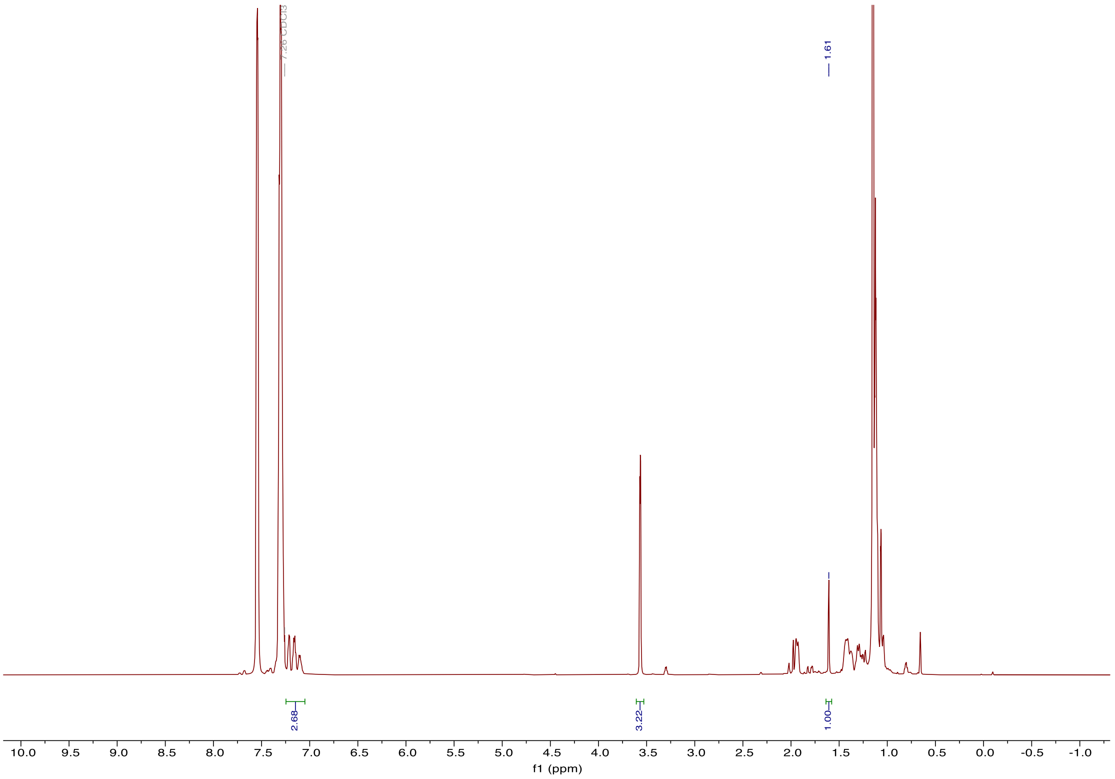

Entry 6: 2.89 (DCE, 4H):1.00 (**3j** BCP, 6H), (2.89/4) : (1/6) =0.7225:0.167
0.2 mmol/(0.7225/0.167) = 0.046 mmol, yield of **3j**: 0.046 mmol/0.1 mmol = 46%
Amount of **4j** formed:(2.89/4) : (3.13/10) = 0.7225:0.313, 0.2 mmol/(0.7225/0.313) = 0.087 mmol

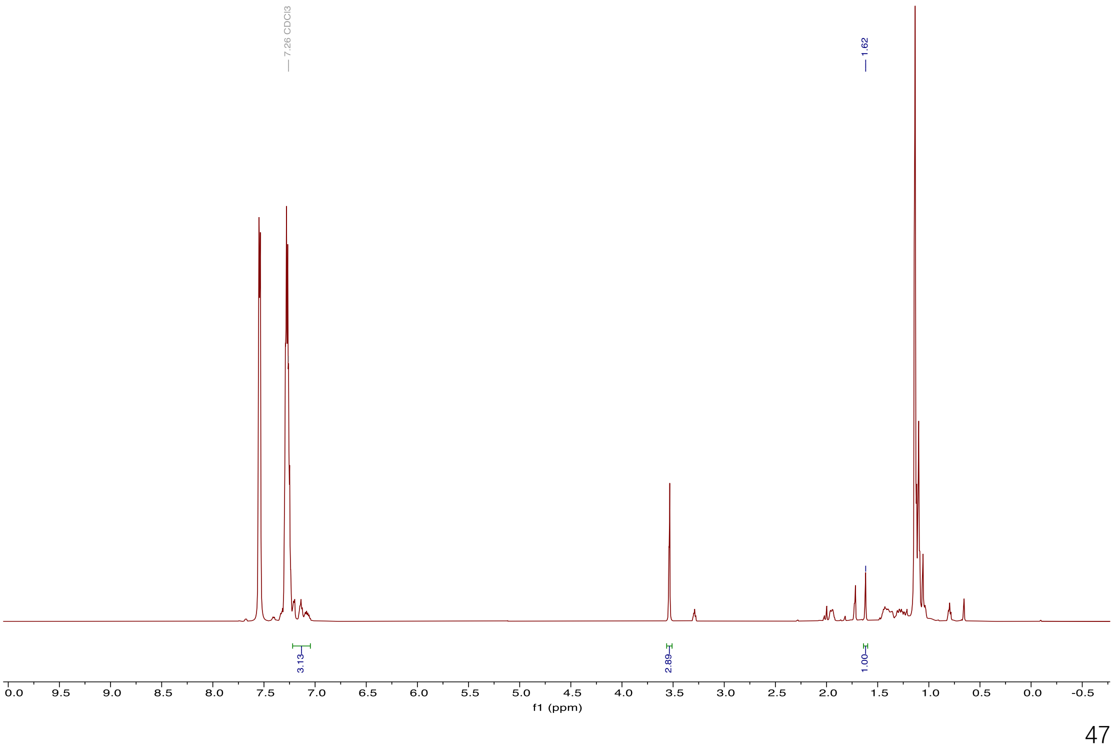

The DMSO replacement experiments demonstrate that sulfoxide (DMSO and phenyl sulfoxide) is a key factor for the successful reactions under both iron and base-free conditions. Based on the above experiments, we hypothesize that B_2pin_2 may have coordinated with DMSO and released OBpin radical. To further verify our hypothesis, EPR (electron paramagnetic resonance) experiments were performed.

EPR study:

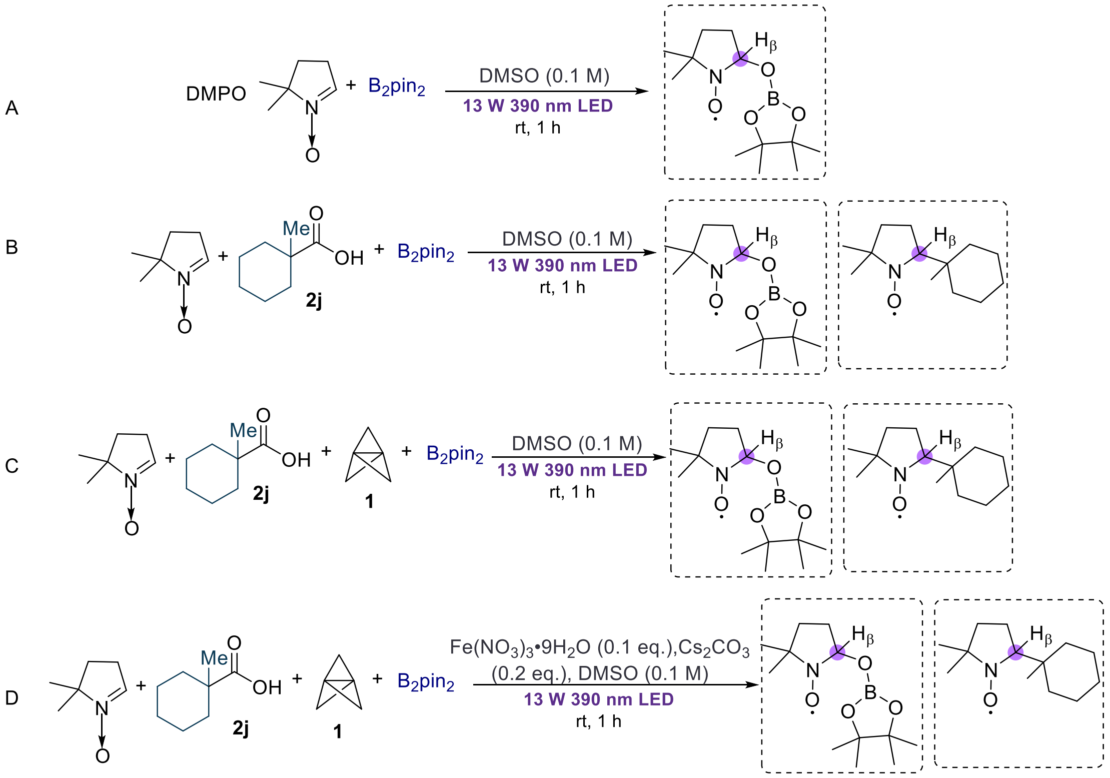

Spin-trap EPR experiment Photo spin-trapping reactions were set up as follows: in 1 ml DMSO which contained 5,5-dimethyl-1-pyrroline *N*-oxide (DMPO) (113.2 mg, 1.0 mmol, 10.0 equiv.), added (A) B_2pin_2 (76.2 mg, 0.3 mmol, 3.0 equiv.), (B) B_2pin_2 (76.2 mg, 0.3 mmol, 3.0 equiv.) plus acid **2j** (28.4 mg, 0.2 mmol, 2.0 equiv.), (C) B_2pin_2 (76.2 mg, 0.3 mmol, 3.0 equiv.) plus acid **2j** (28.4 mg, 0.2 mmol, 2.0 equiv.) and [1.1.1]propellane (0.1 mmol, 1.0 equiv.), and (D) B_2pin_2 (76.2 mg, 0.3 mmol, 3.0 equiv.) plus acid **2j** (28.4 mg, 0.2 mmol, 2.0 equiv.) and [1.1.1]propellane (0.1 mmol, 1.0 equiv.) and $Fe(NO_3)_3 \cdot 9\,H_2O$ (4.0 mg, 0.01 mmol, 0.1 equiv.) and Cs_2CO_3 (6.5 mg, 0.02 mmol, 0.2 equiv.). The reaction mixtures were placed in 390 nm Kessil® light (25% intensity, 13 W) with a cooling fan stirring for 1 h before sampling with glass capillary tubes, which were then sealed with Critoseal and transferred to EPR cavity for measurements.

X-band EPR spectra of DMPO-radical adducts were recorded on a Bruker EMX spectrometer. EPR parameters were: frequency, 9.3 GHz; microwave power, 10 mW; scan range, 80 G; modulation frequency, 100 kHz; modulation amplitude, 1.0 G, time constant, 0.16 s, and receiver gain, 1×10^5 or 7.1×10^4. One or four scans were acquired for each sample. The spectra were analyzed and simulated using WinEPR and SimFonia, respectively.

Results: significant amounts of radicals were captured using DMPO in the photoreactions of B_2pin_2 with DMSO solvent, both in the absence and presence of acid and iron catalysts (Fig. 1). On the other hand, no radical EPR signal was observed in the photoreaction in the absence of a spin-trapping reagent (Fig. 2), indicating that any radical intermediate produced in the photoreactions is transient. All the DMPO-radical adducts show a g value of 2.006. In the absence of acid or iron, the radical produced in the photoreaction with B_2pin_2 only is mainly an oxygen-centered radical as indicated by the hyperfine splitting constants, a_N = 13.7 G and $a_{\beta\text{-}H}$ = 11.7 G, that are typical of DMPO adduct with an oxygen-centered radical (Figure 1A). The oxygen-centered radical most likely resides on the •OBpin moiety generated from the B_2pin_2/DMSO complex. Moreover, the $a_N/a_{\beta\text{-}H}$ ratio of the DMPO-OB(OR)$_2$ adduct is 1.2, which is similar to those observed in DMPO-OOR adducts but noticeably larger than those of DMPO-OR adducts. The boron atom therefore affects the hyperfine splittings in the DMPO-OB(OR)$_2$ adduct in a similar pattern to the second oxygen atom in DMPO-OOR adducts.

EPR data of the acid-catalyzed photoreaction was more complicated (Figure 1B) compared to spectrum A. The extra EPR features suggest that DMPO captured both radicals- the oxygen-center O-Bpin radical (compared to spectrum A) and a carbon-center radical (compared to spectrum D, *vide infra*). Moreover, more EPR features were observed which may be due to different conformers of DMPO adduct(s) in the presence of acid. The radical captured in reactions C and D, catalyzed by both acid and **1** (C) plus iron and cesium carbonate (D), was mainly a carbon-centered radical, as indicated by the hyperfine splitting constants a_N = 14.6 G and $a_{\beta\text{-}H}$ = 21.3 G and $a_N/a_{\beta\text{-}H}$ = 0.69 that are typical of DMPO adducts with carbon-centered radicals, DMPO-CR (Figure 1D). We proposed that the oxygen centered radical observed in reaction A was generated from homolytic cleavage of B_2pin_2-DMSO complex and the carbon-centered radical observed in reactions B – D was produced by decarboxylation of **2j**. It appears that the decarboxylation reaction was enhanced by addition of iron salt and base, as indicated by the significantly larger size of the EPR signal in spectrum D compared to the same signals in spectra B and C. Thus, we hypothesized that iron-mediated LMCT and •OBpin mediated HAT synergistically accelerated the decarboxylation process.

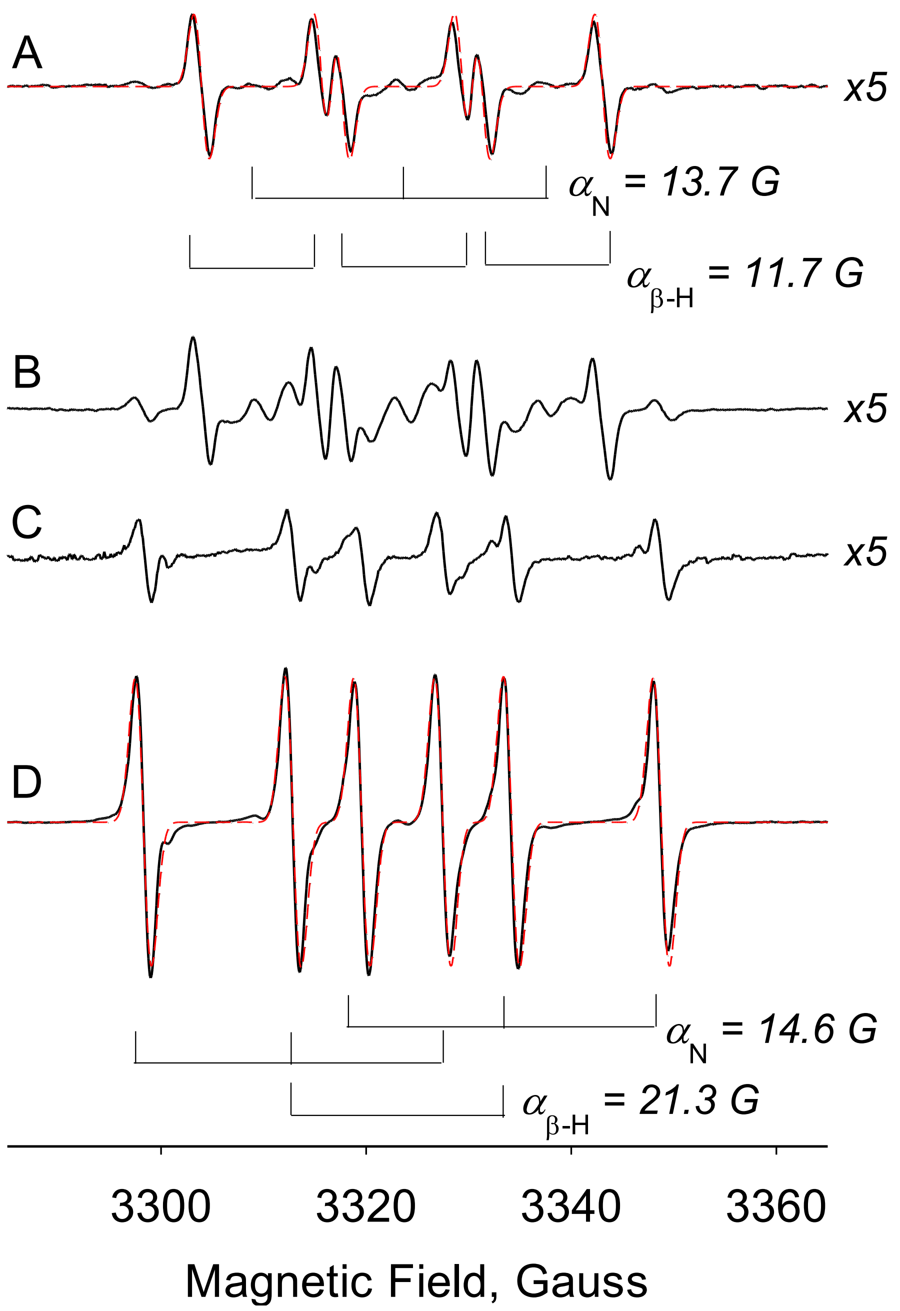

Fig.1. EPR spectra of the trapped radical species in the photoreactions. The EPR spectra of DMPO-trapped radical(s) in the photoreactions of (A) B_2pin_2 only, (B) B_2pin_2 in the presence of acid, (C) B_2pin_2 in the presence of acid **2j** and **1**, and (D) B_2pin_2 in the presence of acid **2j**, **1**, iron and cesium salts. Red dash lines: simulations of the major DMPO-radical adducts using the marked hyperfine splitting constants. The EPR data is normalized for receiver gain and number of scans for direct comparison. Spectra (A) – (C) are scaled by a factor of 5, marked by "x5" on the right, for easy visualization. The hyperfine splitting constants of nitrogen and *β*-proton of DMPO-radical adducts, a_N and $a_{\beta\text{-}H}$, are labeled for spectra (A) and (D).

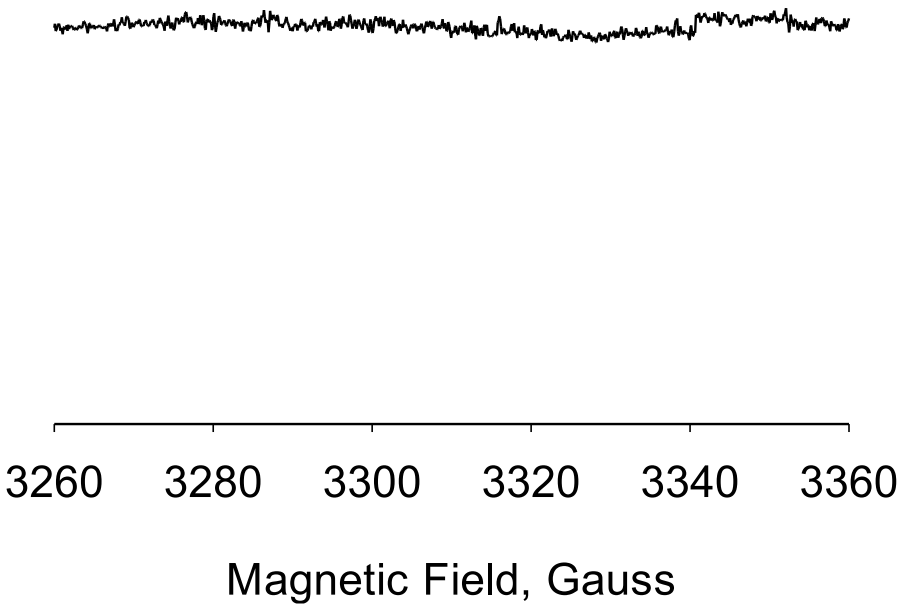

Fig. 2. EPR spectrum of the photoreaction mixture in the absence of DMPO. The same reaction of (D) in the absence of DMPO. No EPR signal is observed.

4. NMR spectra of novel compounds.

2-(3-isopentylbicyclo[1.1.1]pentan-1-yl)-4,4,5,5-tetramethyl-1,3,2-dioxaborolane (3a)

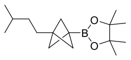

^{1}H NMR (600 MHz, $CDCl_3$)

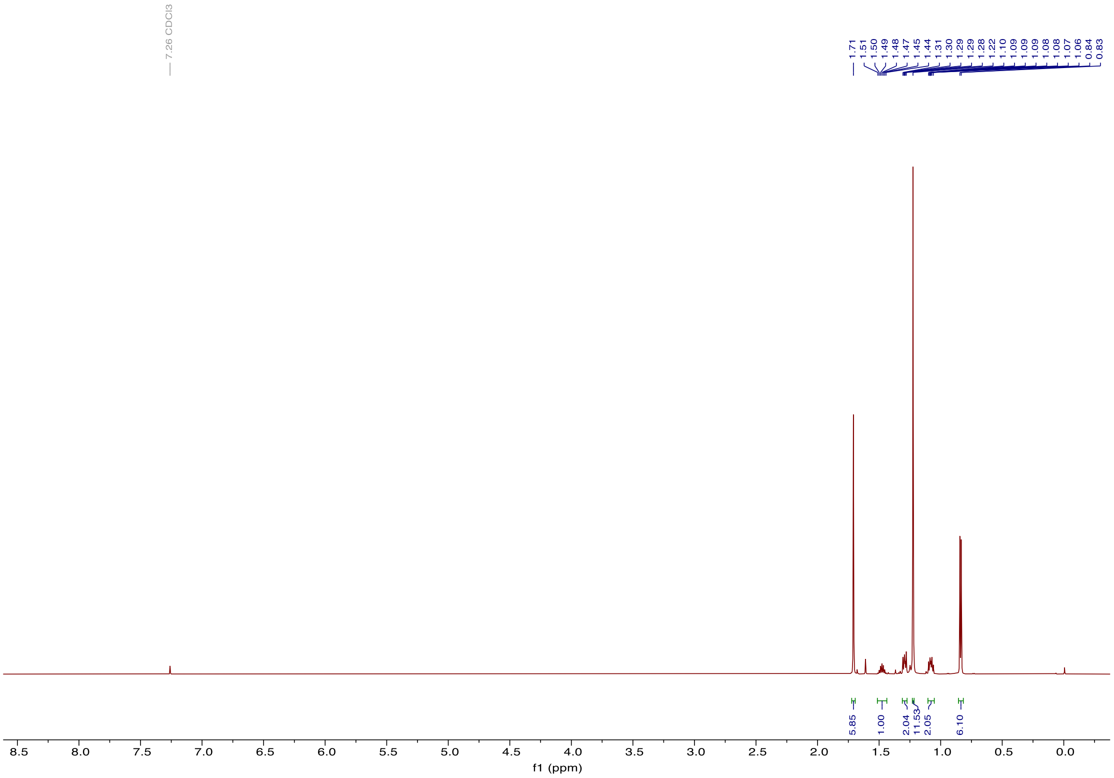

^{13}C NMR (151 MHz, $CDCl_3$)

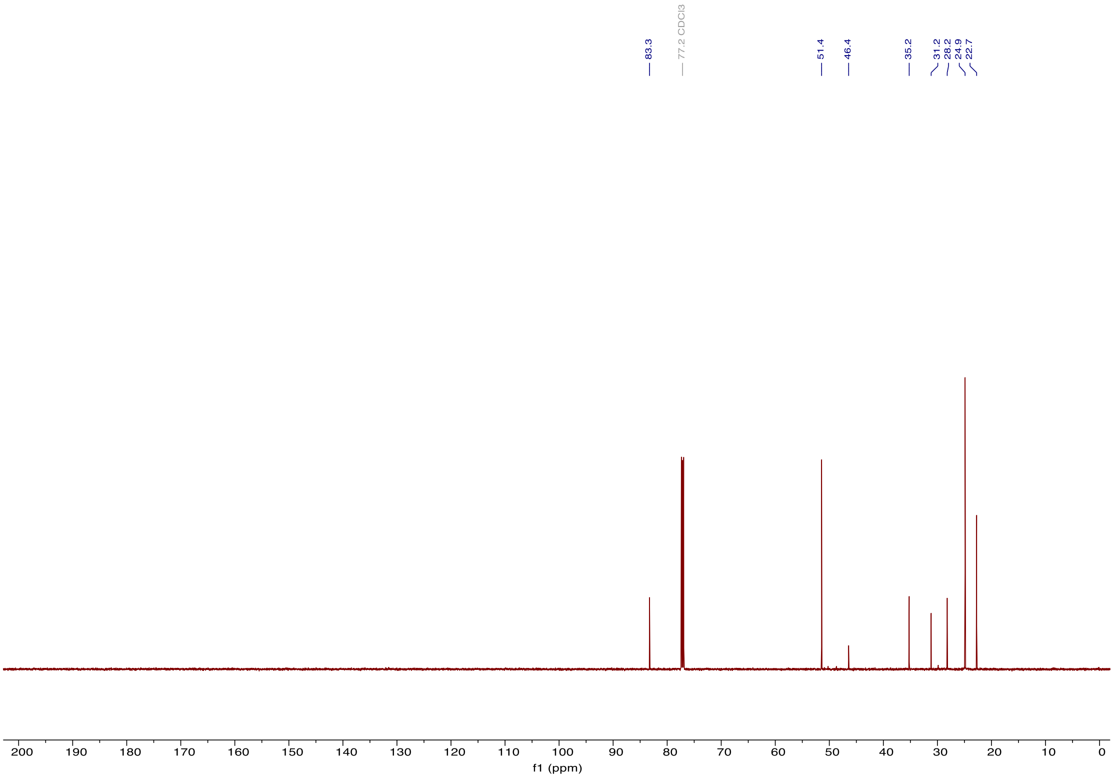

^{11}B NMR (192 MHz, $CDCl_3$)

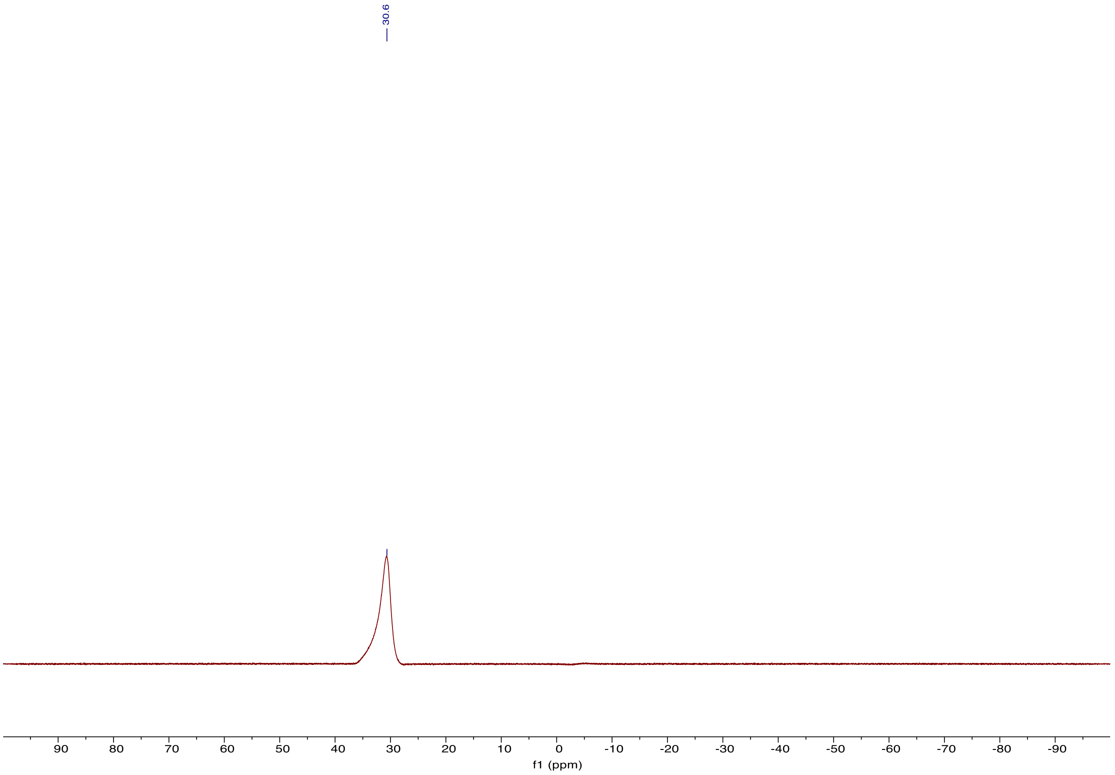

2-(3-butylbicyclo[1.1.1]pentan-1-yl)-4,4,5,5-tetramethyl-1,3,2-dioxaborolane (3b)

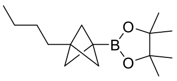

^{1}H NMR (600 MHz, $CDCl_3$)

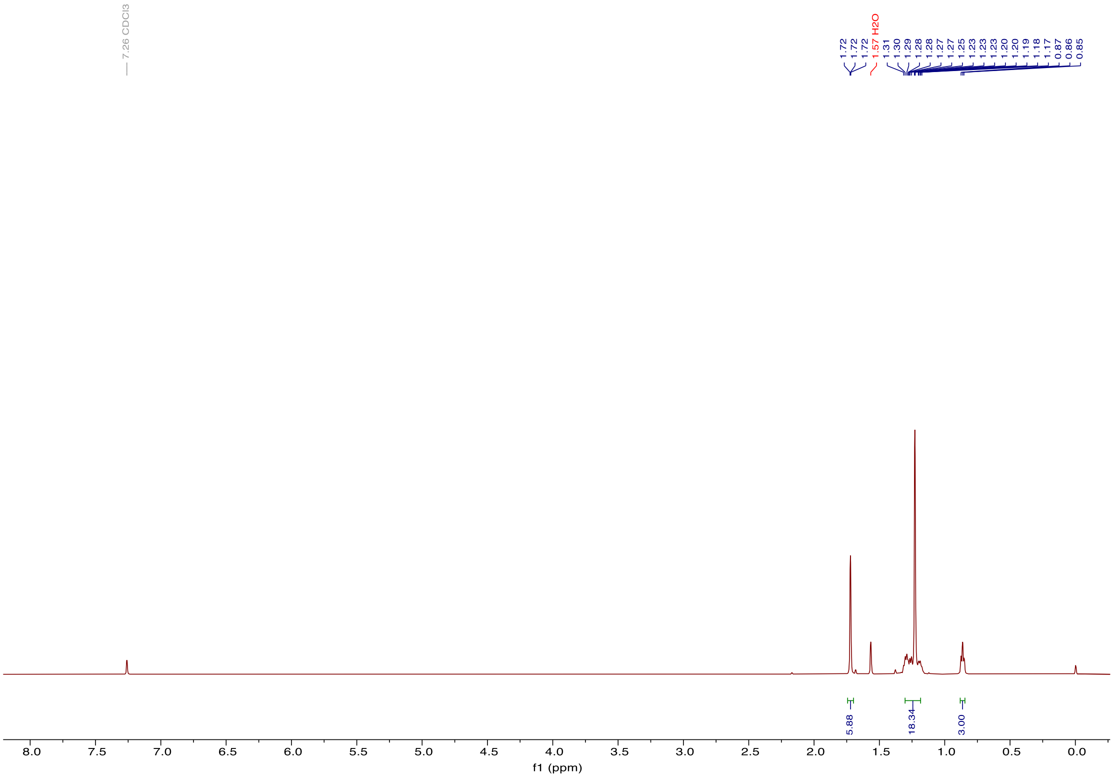

^{13}C NMR (151 MHz, CDCl$_3$)

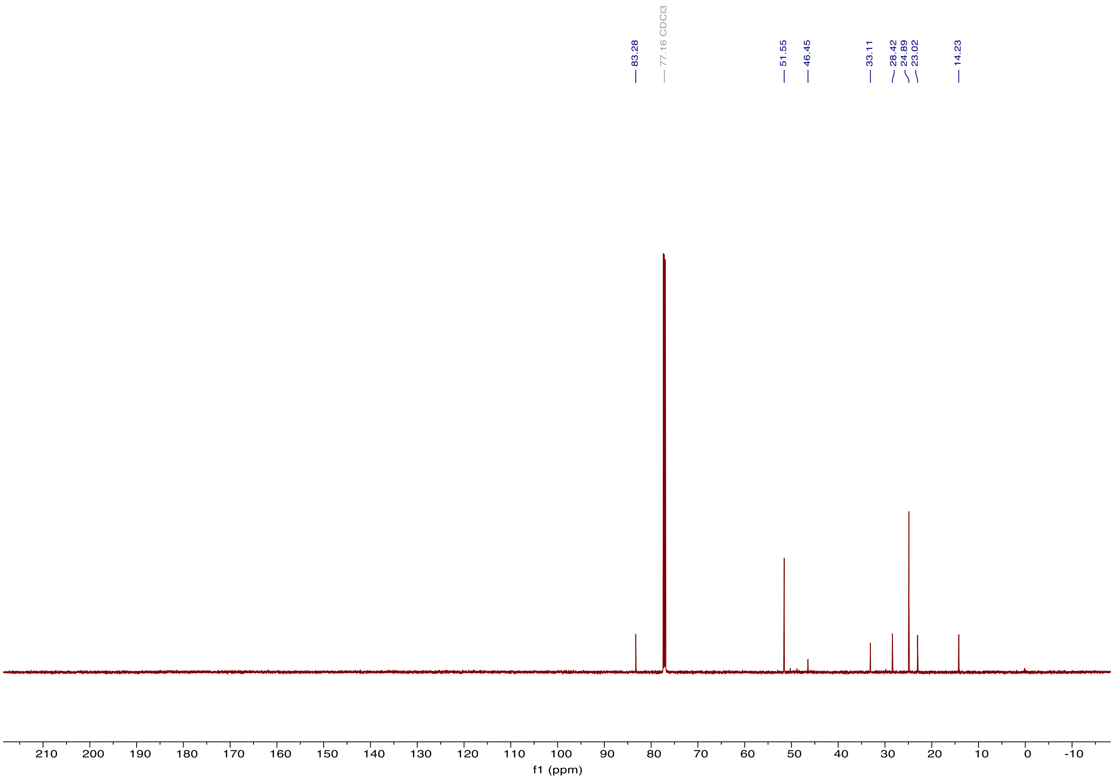

^{11}B NMR (192 MHz, CDCl$_3$)

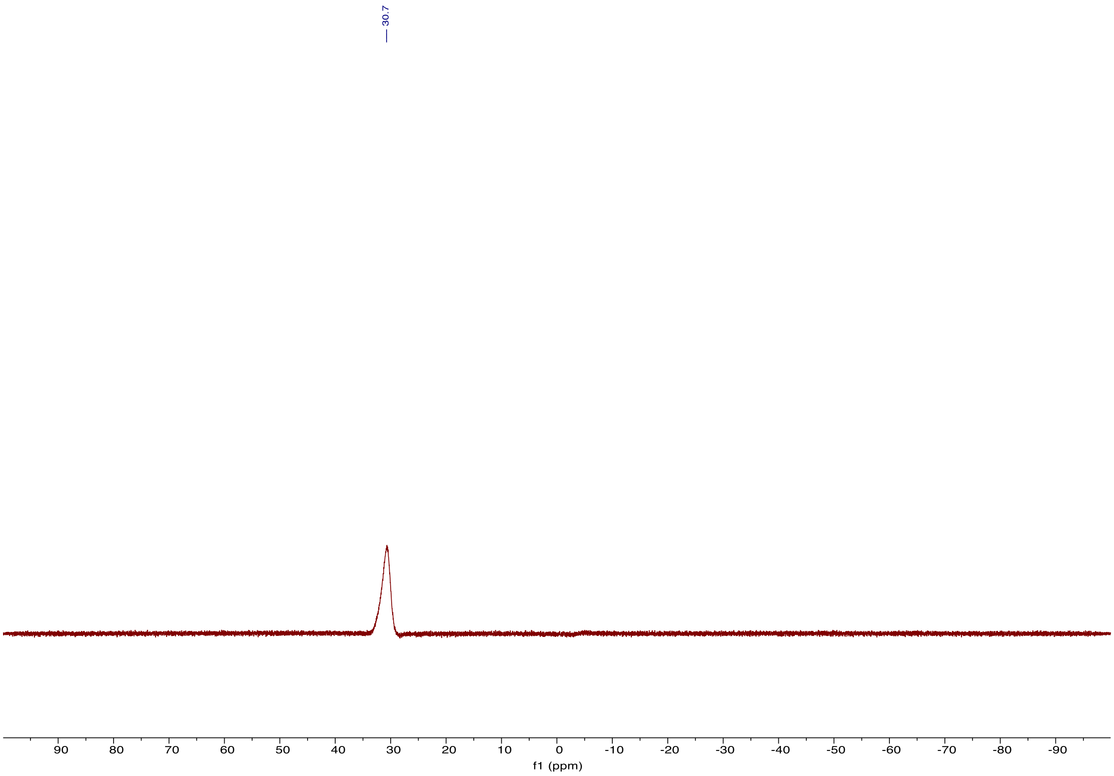

2-(3-(dec-9-yn-1-yl)bicyclo[1.1.1]pentan-1-yl)-4,4,5,5-tetramethyl-1,3,2-dioxaborolane (3c)

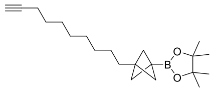

^{1}H NMR (600 MHz, CDCl$_3$)

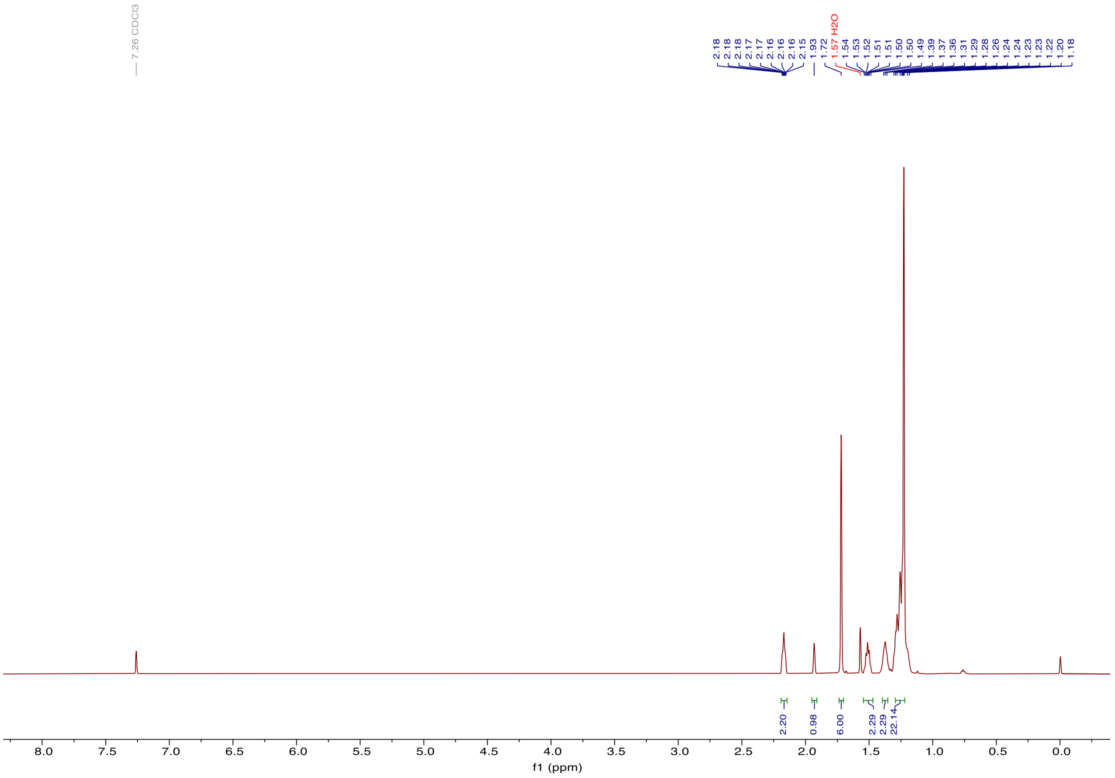

^{13}C NMR (151 MHz, CDCl$_3$)

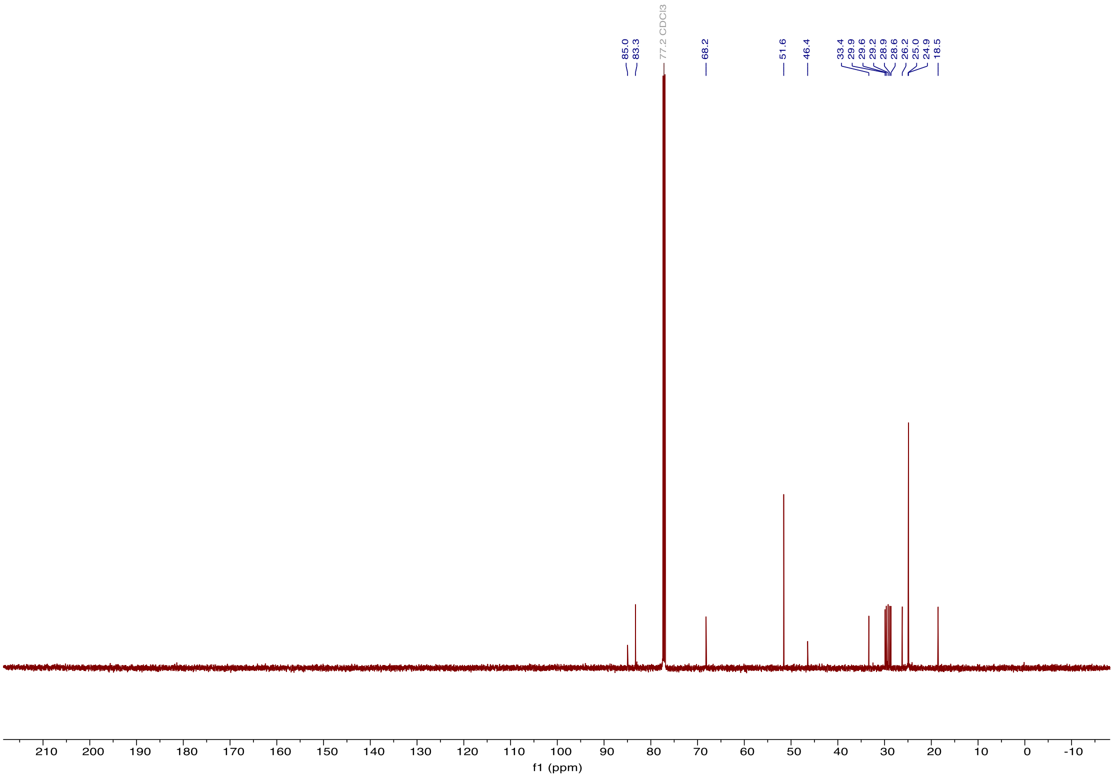

^{11}B NMR (192 MHz, $CDCl_3$)

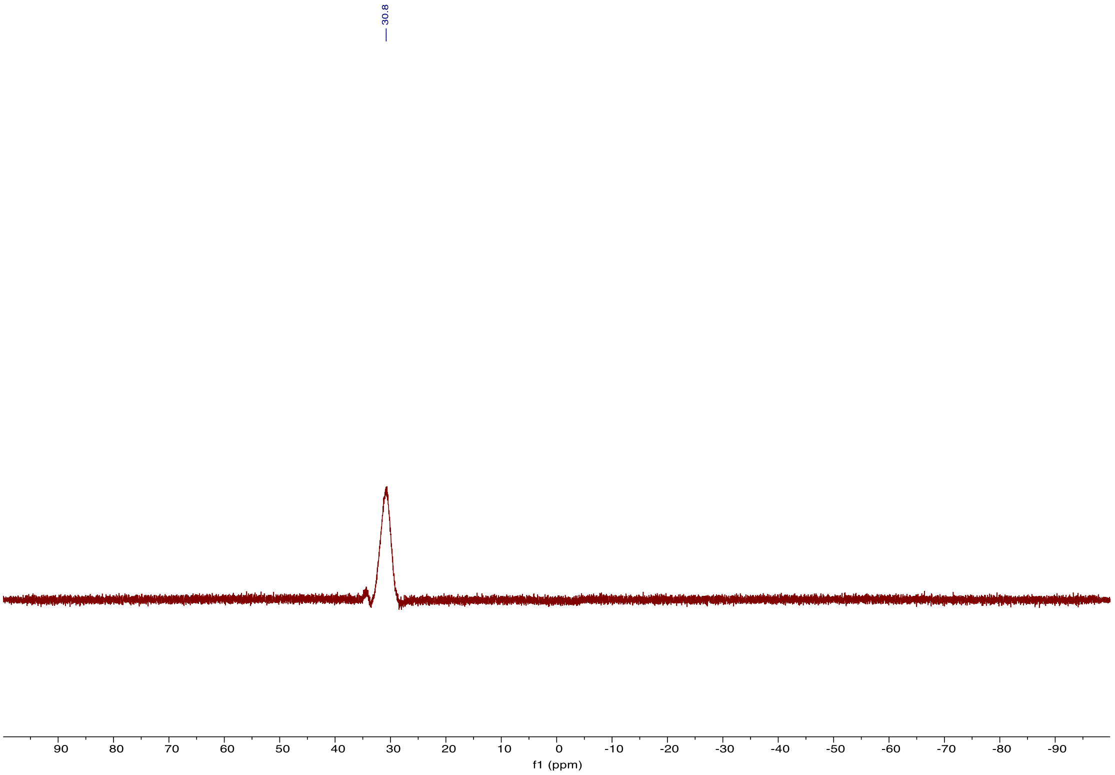

2-(3-(but-3-en-1-yl)bicyclo[1.1.1]pentan-1-yl)-4,4,5,5-tetramethyl-1,3,2-dioxaborolane (3d)

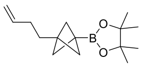

^{1}H NMR (600 MHz, $CDCl_3$)

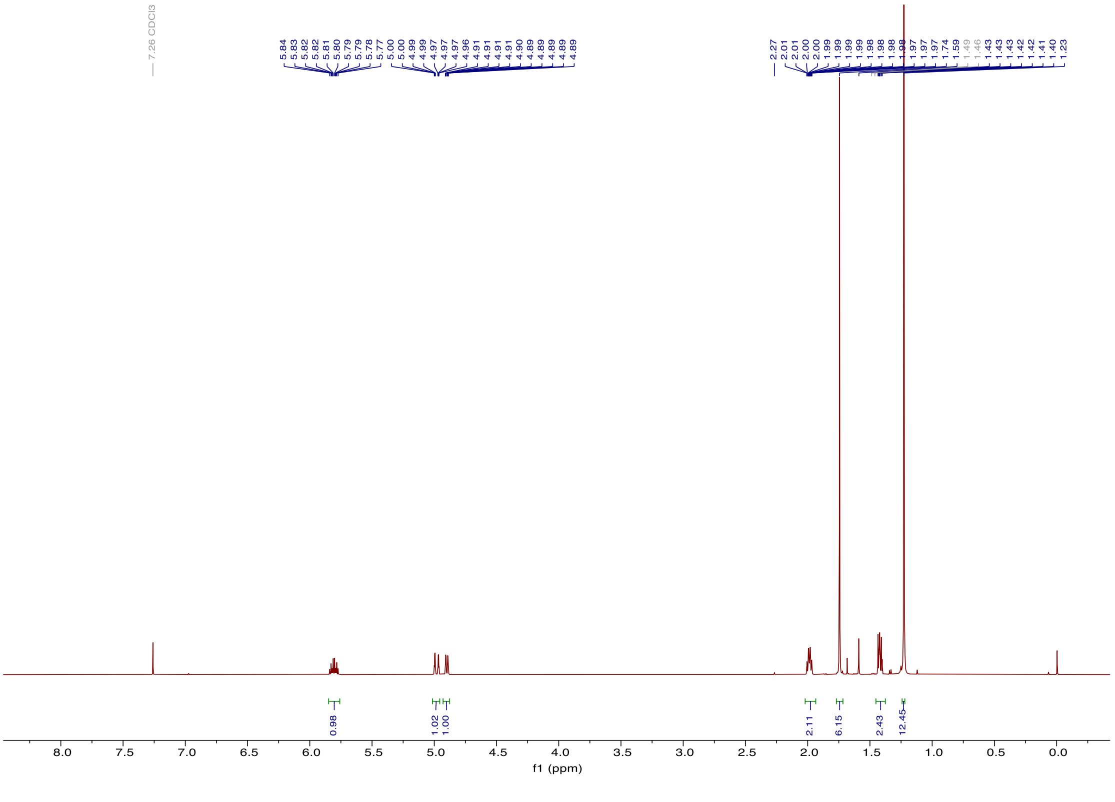

^{13}C NMR (151 MHz, $CDCl_3$)

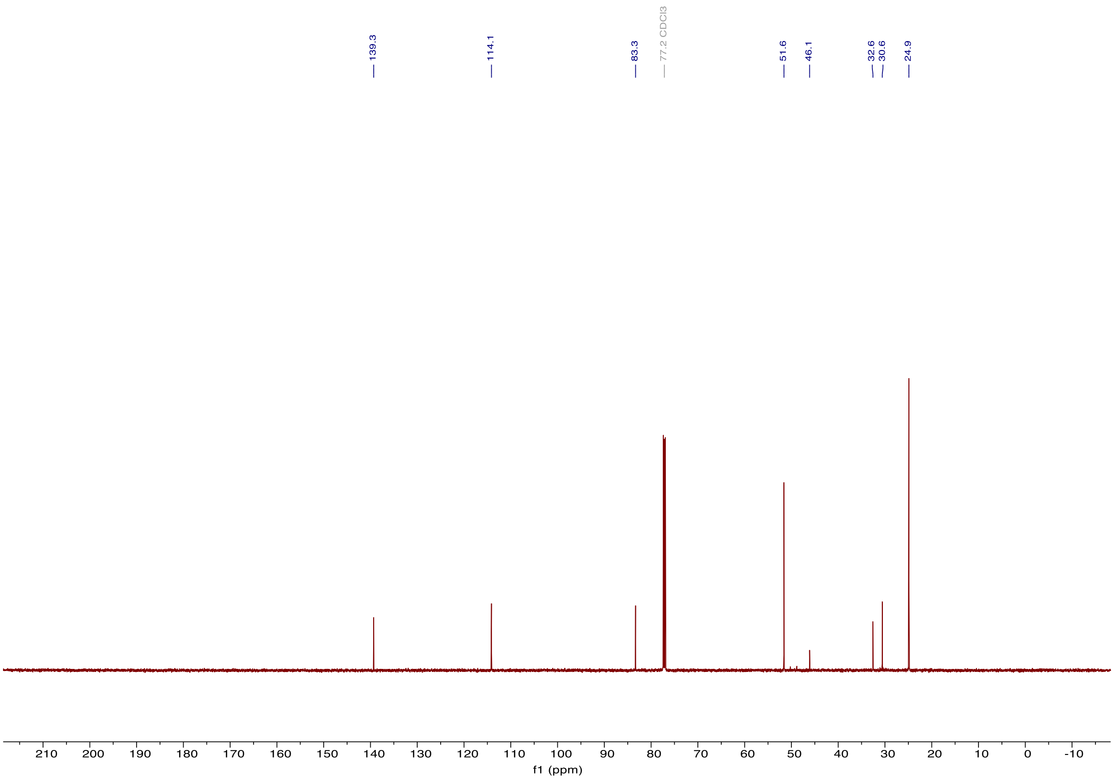

^{11}B NMR (192 MHz, $CDCl_3$)

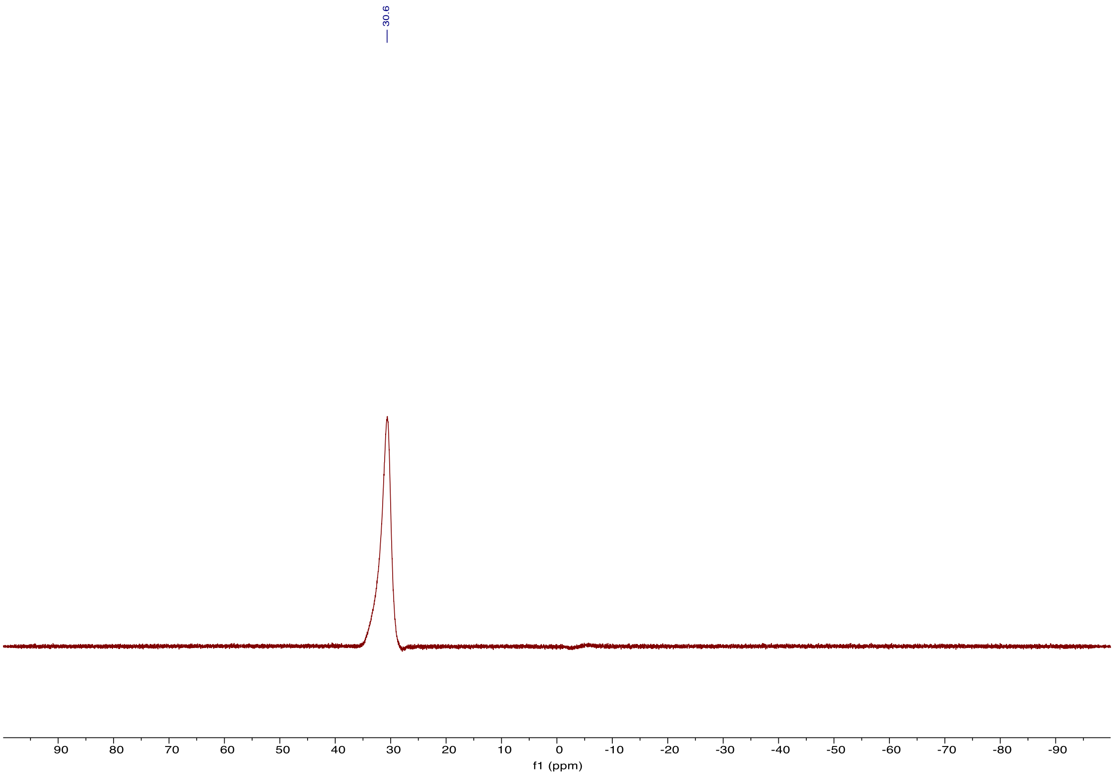

4,4,5,5-tetramethyl-2-(3-(phenoxymethyl)bicyclo[1.1.1]pentan-1-yl)-1,3,2-dioxaborolane (3e)

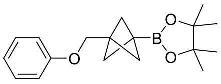

^{1}H NMR (600 MHz, $CDCl_3$)

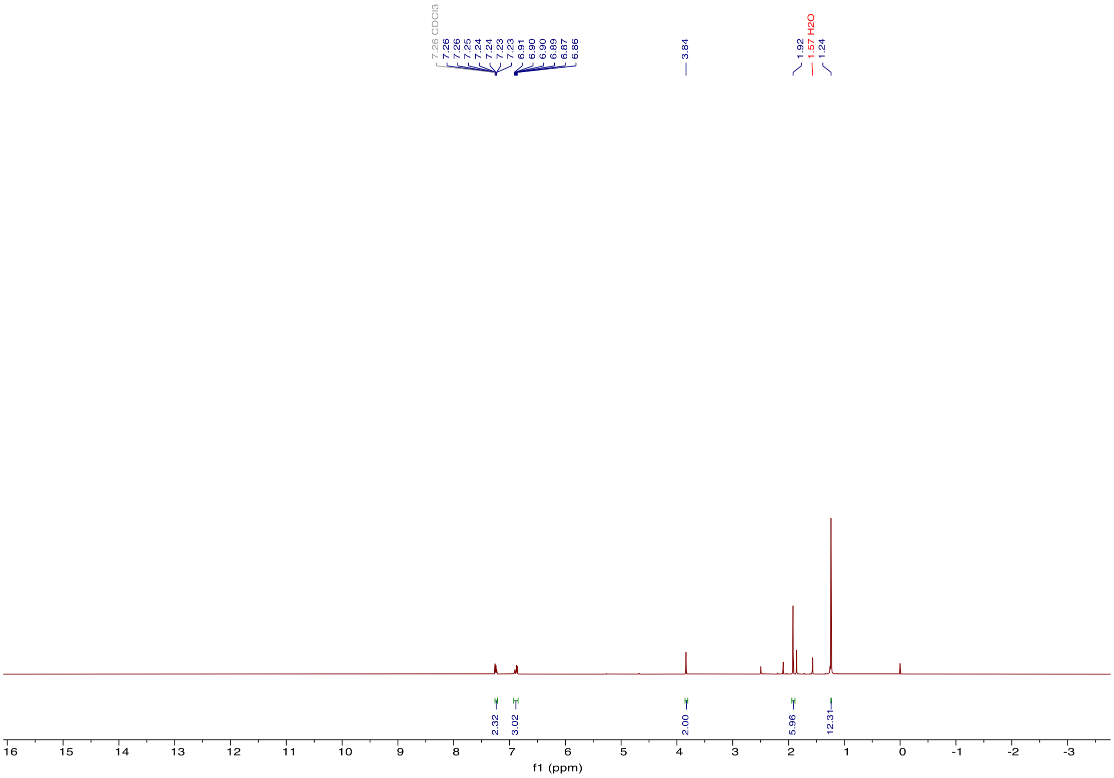

^{13}C NMR (151 MHz, $CDCl_3$)

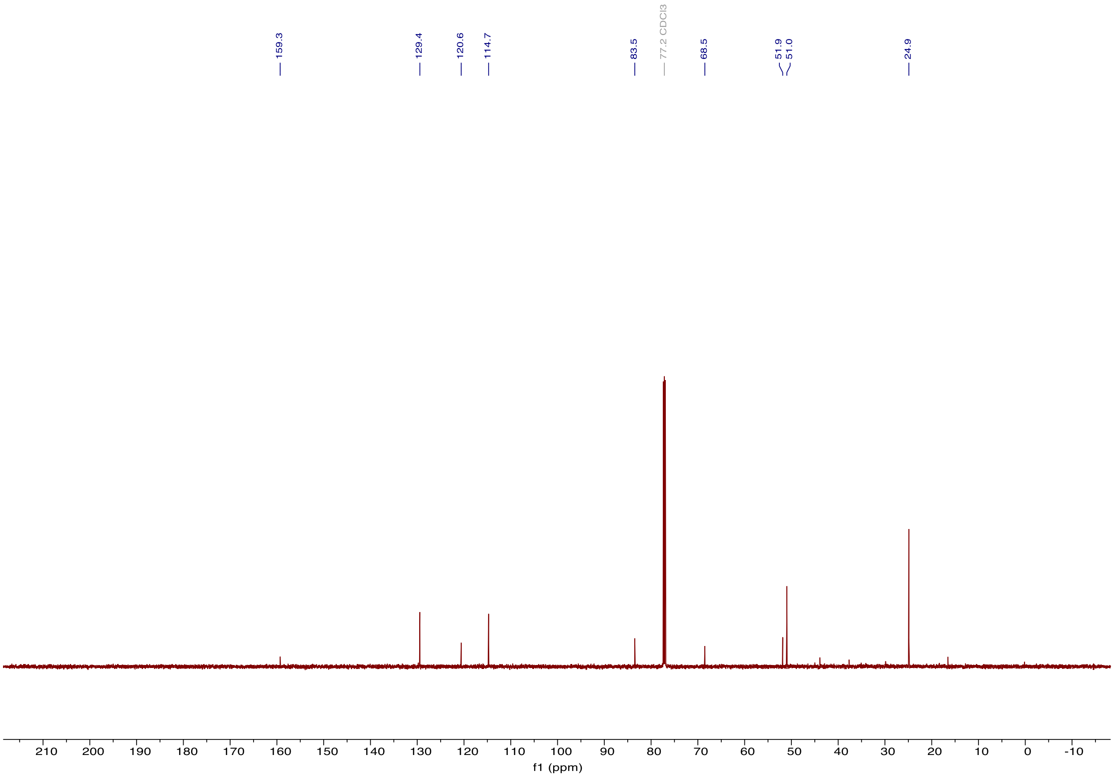

^{11}B NMR (192 MHz, $CDCl_3$)

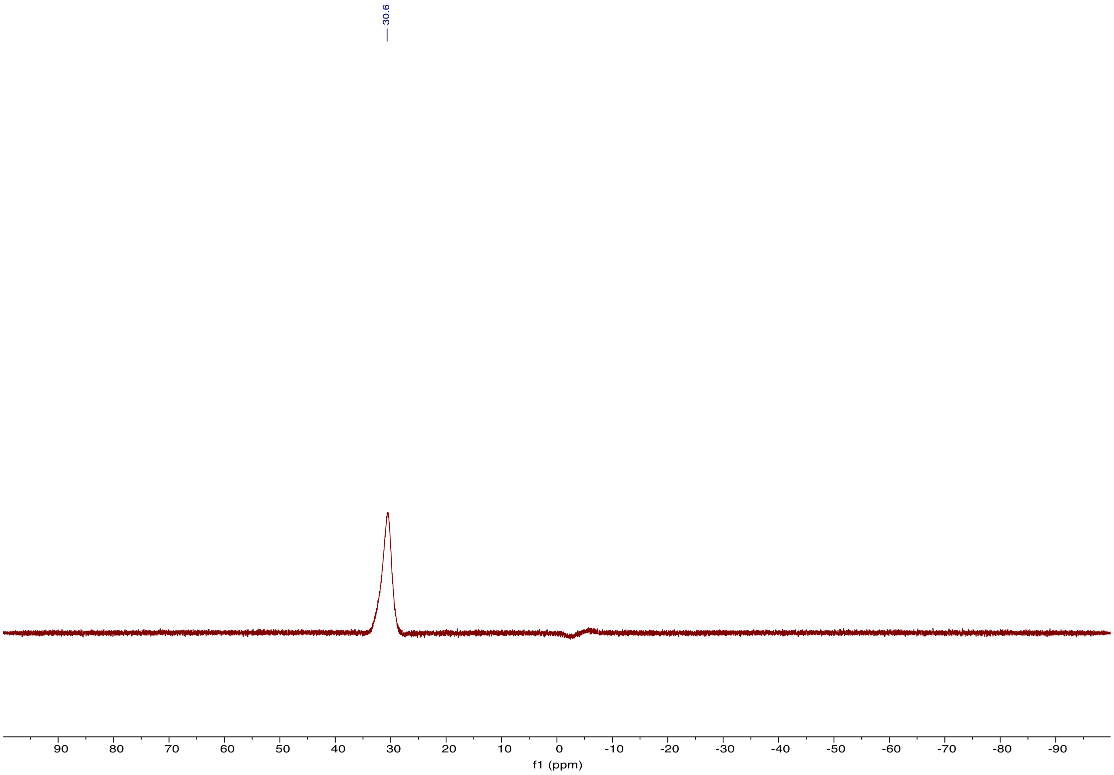

2-(3-(1-(4-fluorophenoxy)ethyl)bicyclo[1.1.1]pentan-1-yl)-4,4,5,5-tetramethyl-1,3,2-dioxaborolane (3f)

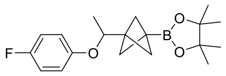

^{1}H NMR (600 MHz, $CDCl_3$)

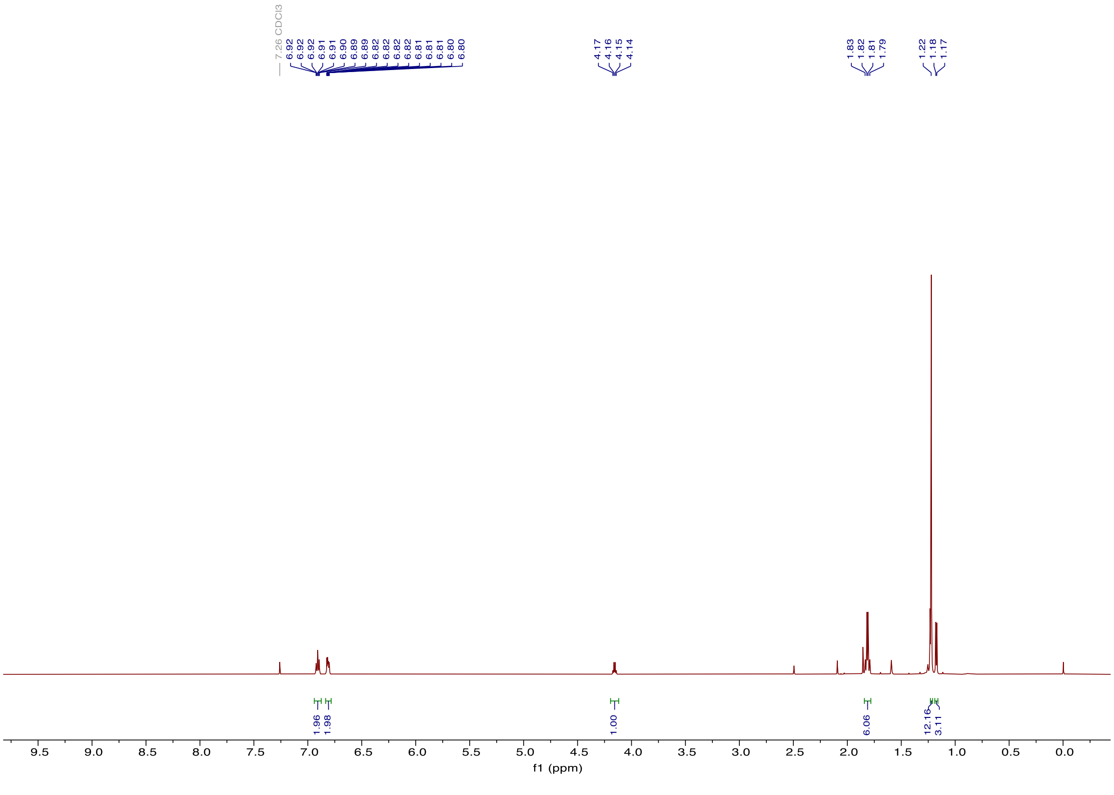

^{13}C NMR (151 MHz, $CDCl_3$)

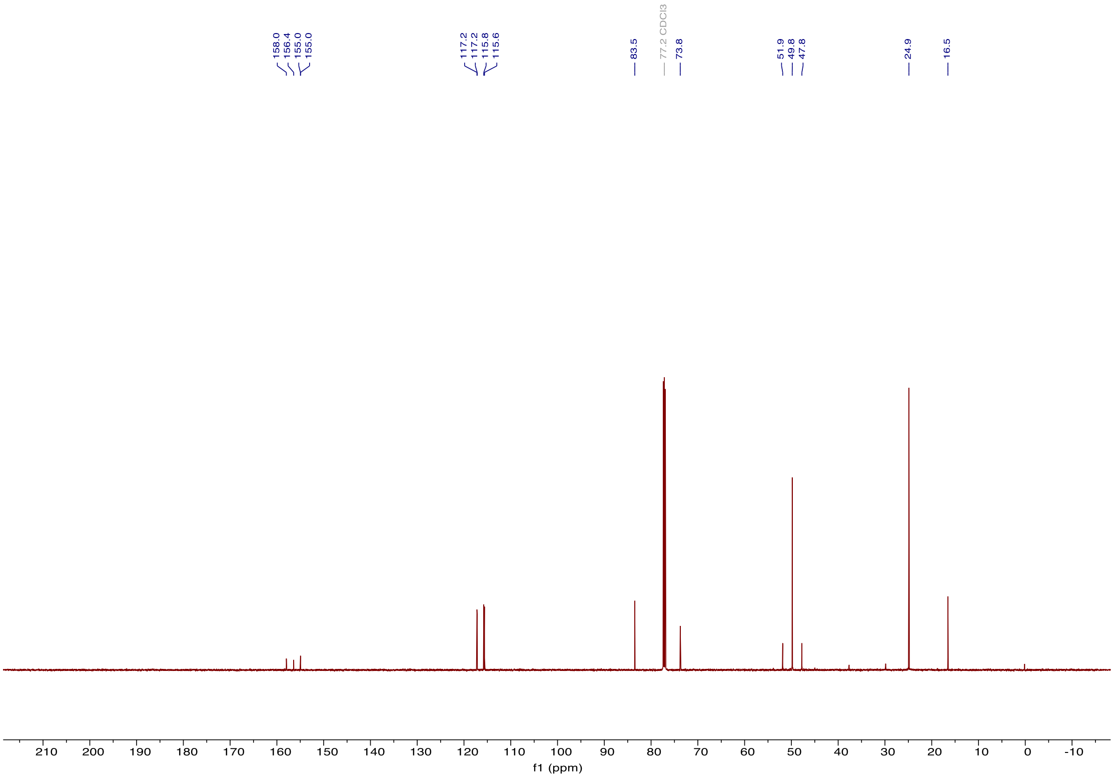

^{11}B NMR (192 MHz, $CDCl_3$)

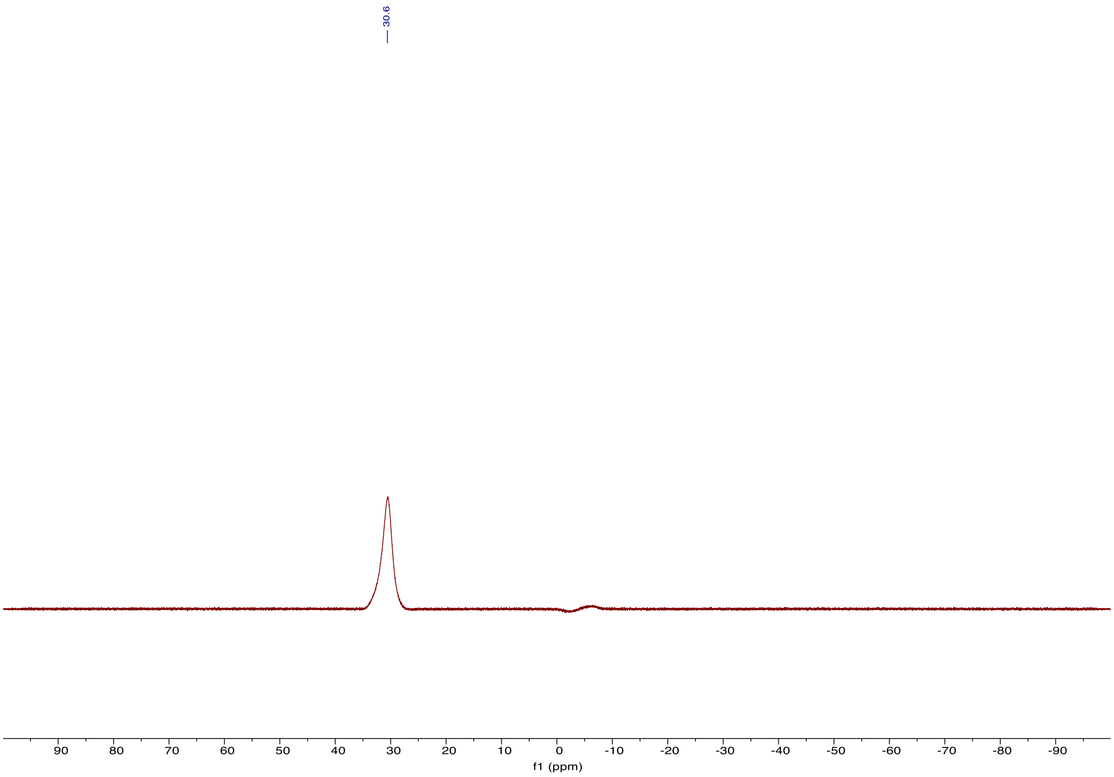

^{19}F NMR (564 MHz, $CDCl_3$)

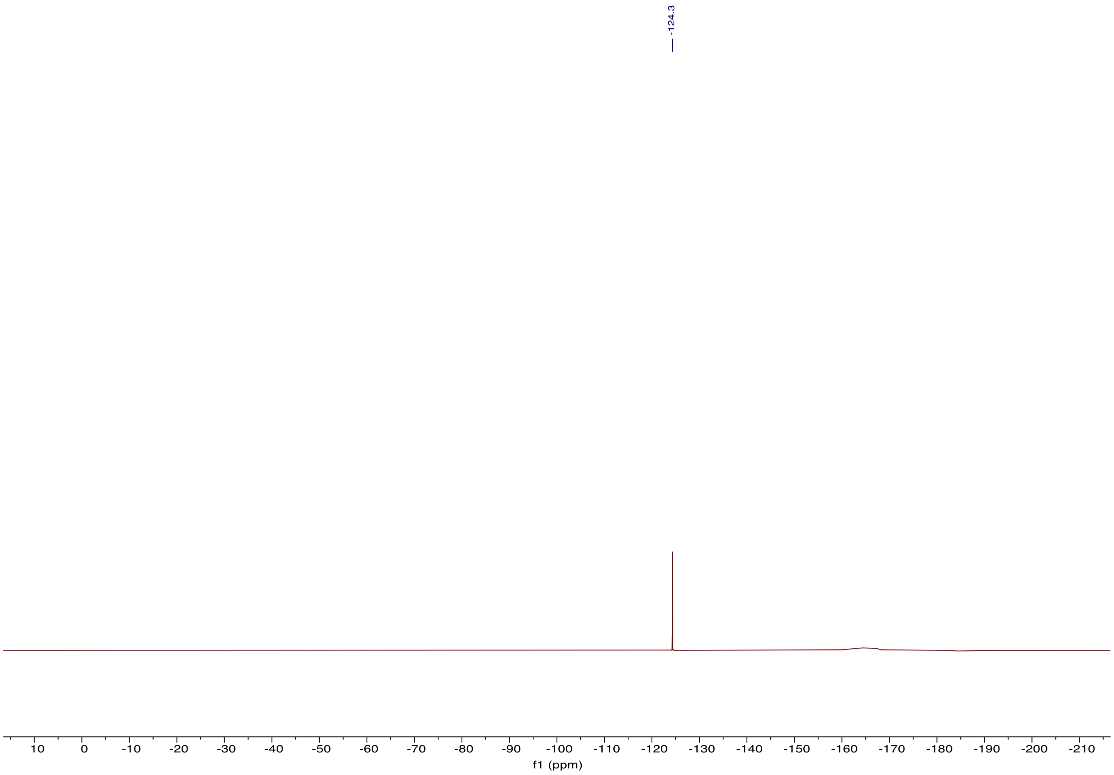

2-(3-(1-(2,4-dichlorophenoxy)ethyl)bicyclo[1.1.1]pentan-1-yl)-4,4,5,5-tetramethyl-1,3,2-dioxaborolane (3g)

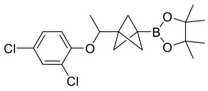

^{1}H NMR (600 MHz, $CDCl_3$)

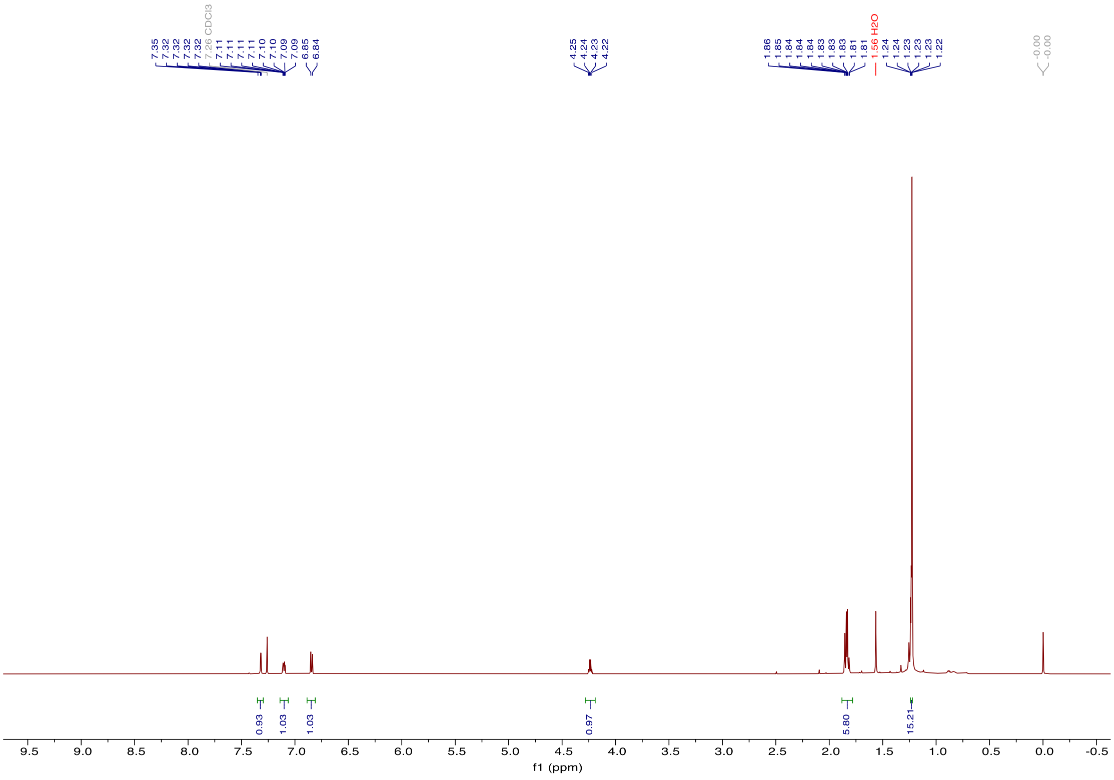

^{13}C NMR (151 MHz, $CDCl_3$)

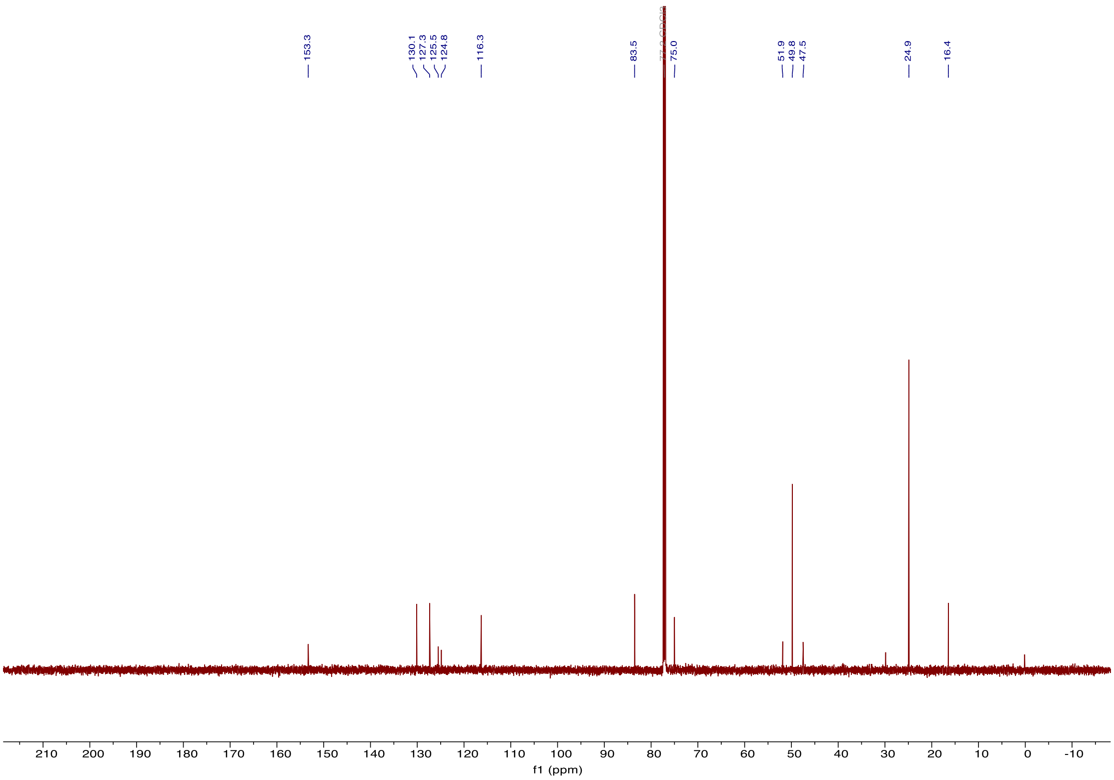

^{11}B NMR (192 MHz, $CDCl_3$)

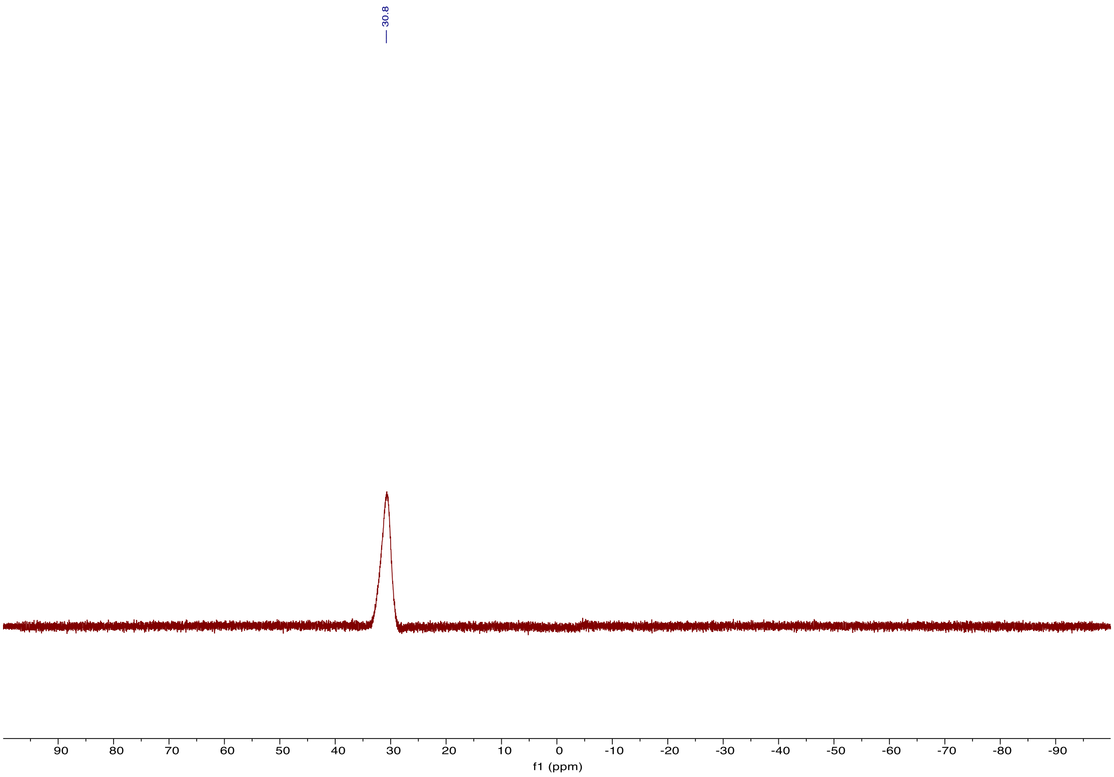

2-(3-(1-methoxyethyl)bicyclo[1.1.1]pentan-1-yl)-4,4,5,5-tetramethyl-1,3,2-dioxaborolane (3h)

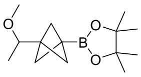

^{1}H NMR (600 MHz, $CDCl_3$)

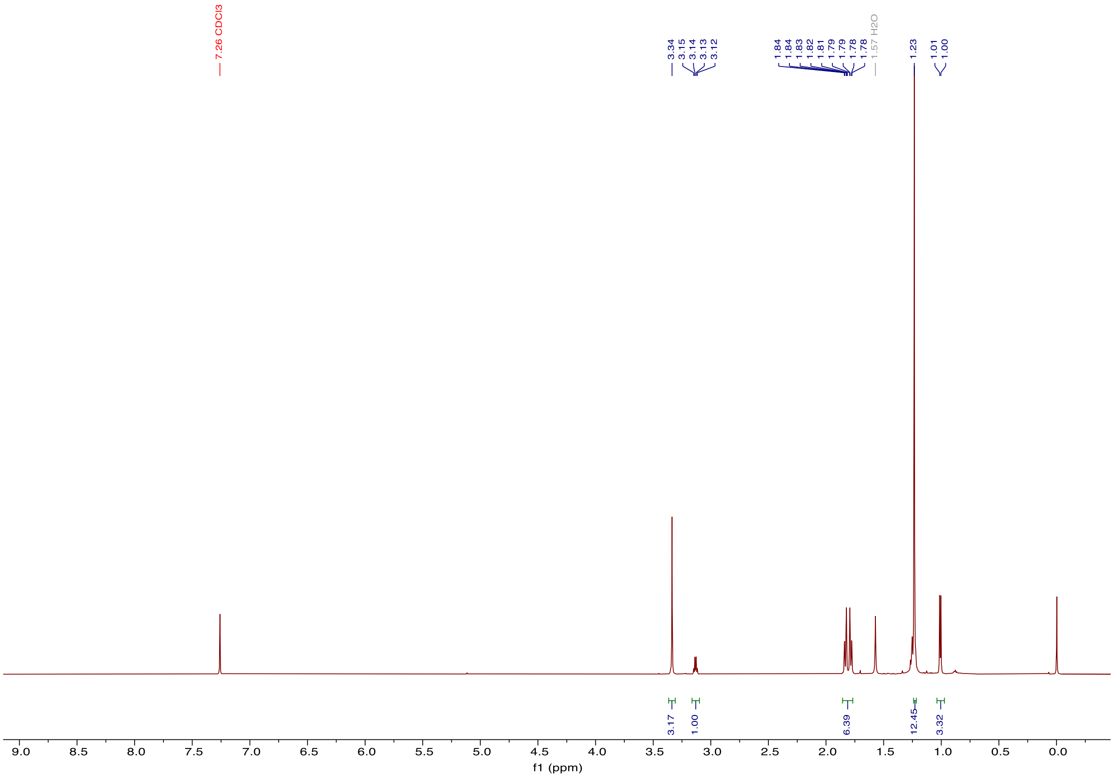

^{13}C NMR (151 MHz, $CDCl_3$)

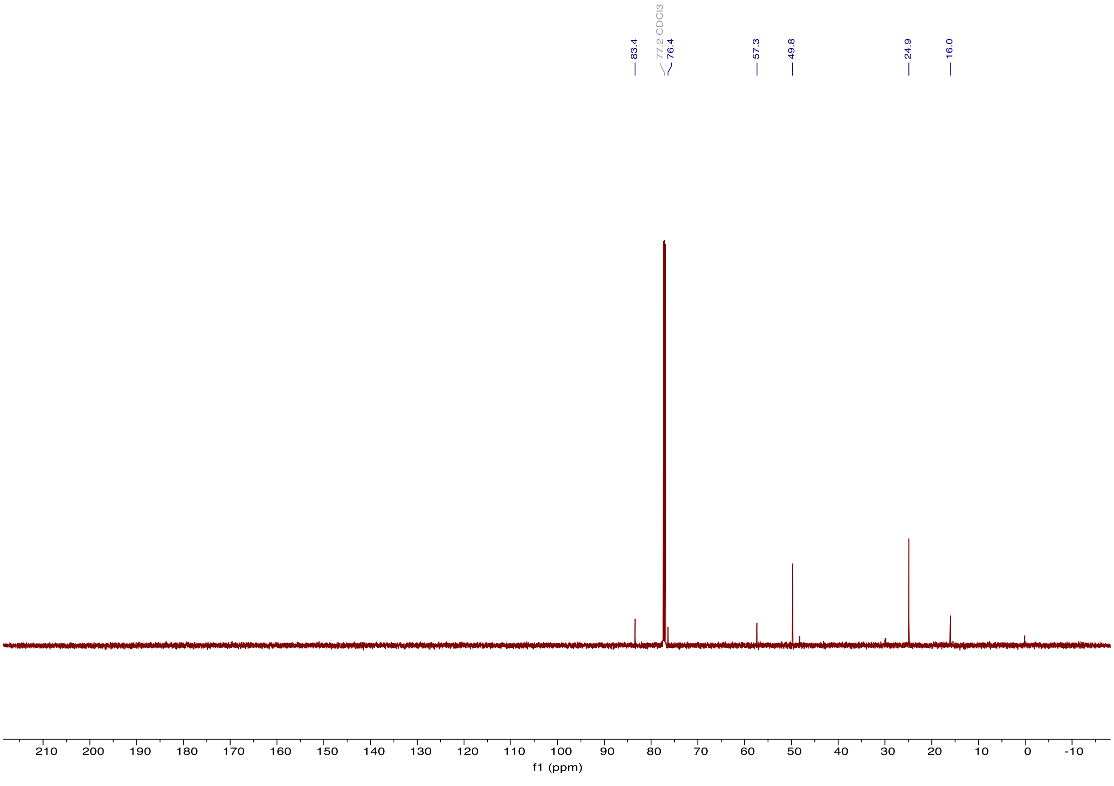

^{11}B NMR (192 MHz, $CDCl_3$)

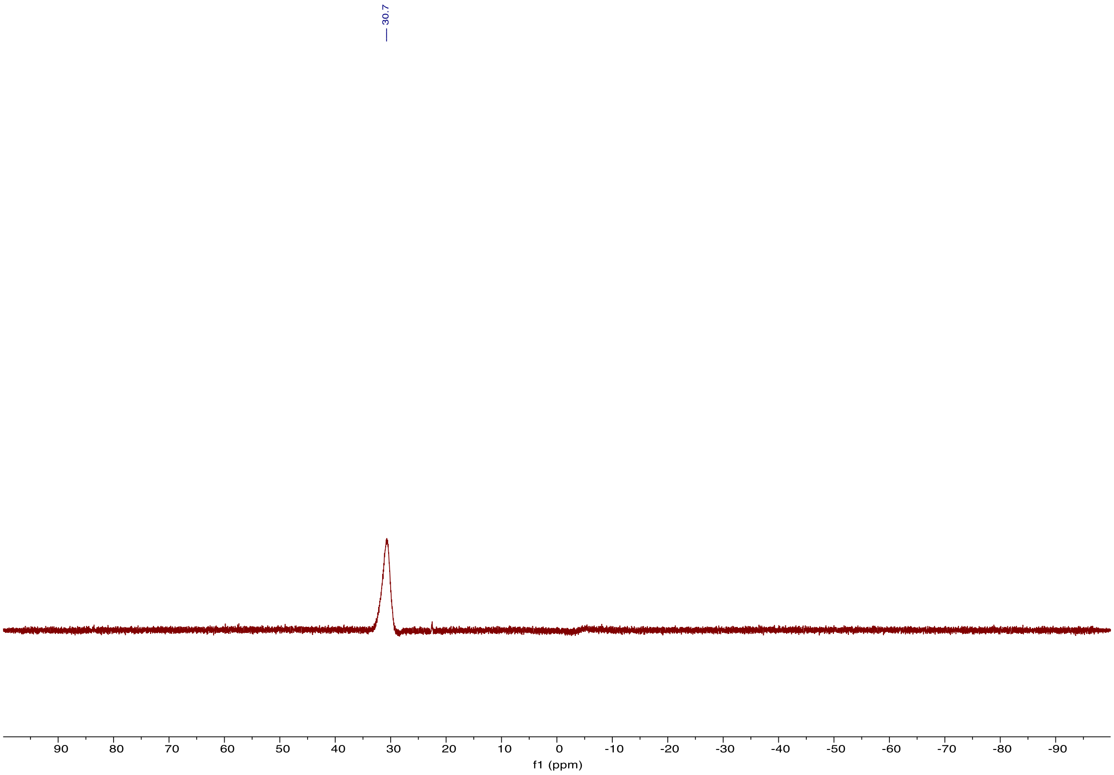

2-(3-(1-methoxy-3-methylbutyl)bicyclo[1.1.1]pentan-1-yl)-4,4,5,5-tetramethyl-1,3,2-dioxaborolane (3i)

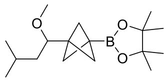

^{1}H NMR (600 MHz, $CDCl_3$)

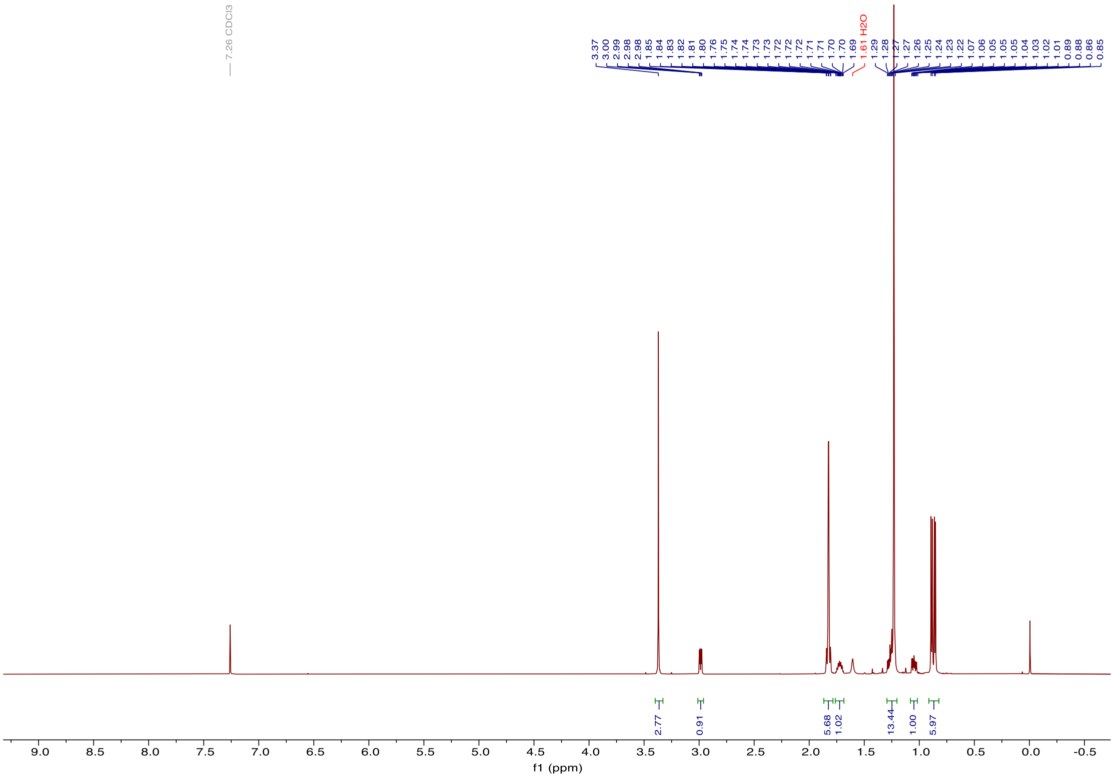

^{13}C NMR (151 MHz, $CDCl_3$)

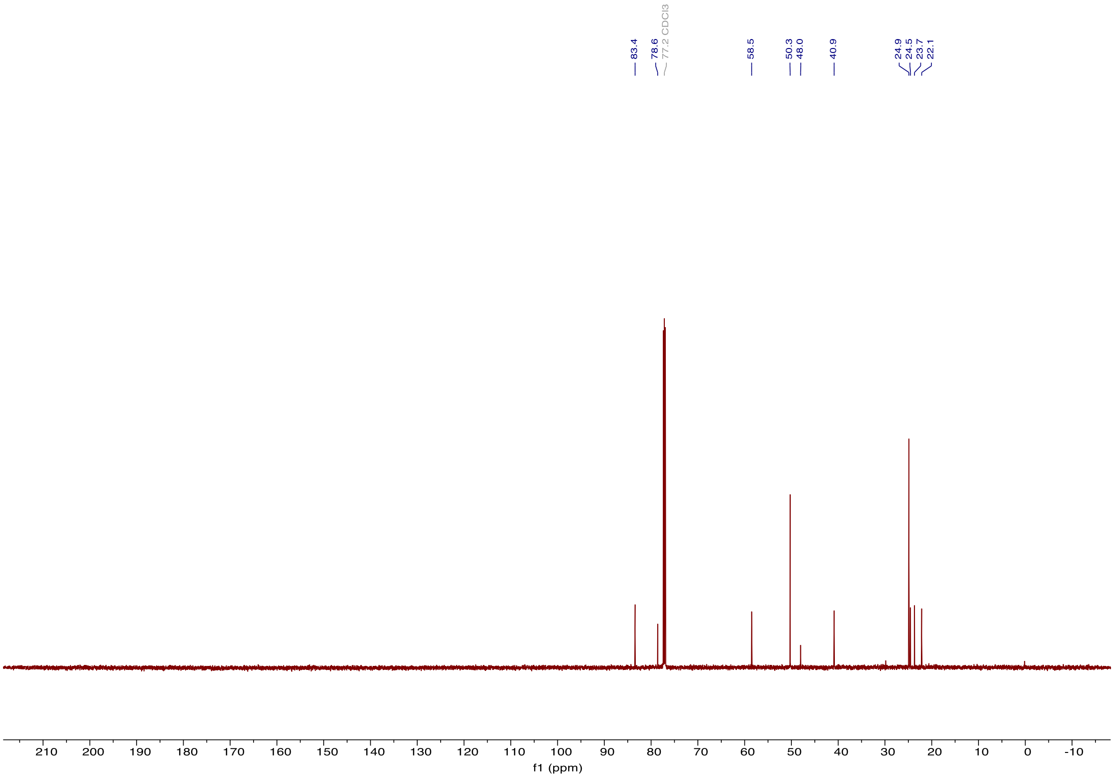

^{11}B NMR (192 MHz, $CDCl_3$)

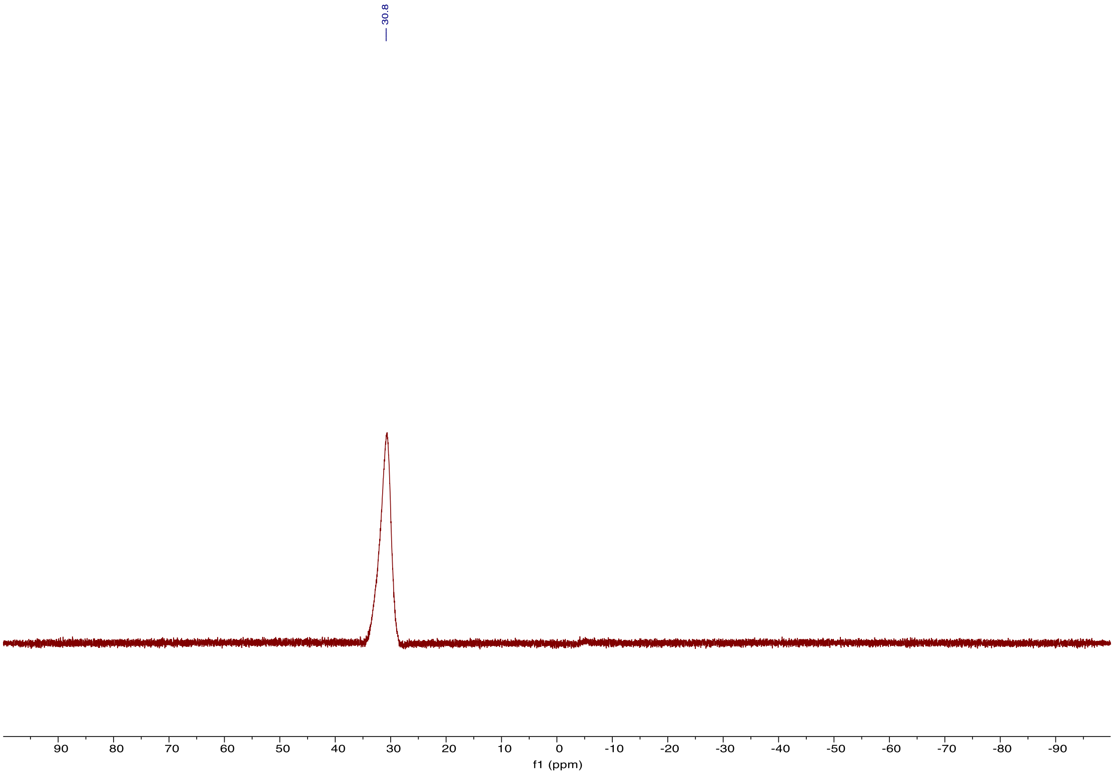

4,4,5,5-tetramethyl-2-(3-(1-methylcyclohexyl)bicyclo[1.1.1]pentan-1-yl)-1,3,2-dioxaborolane (3j)

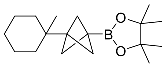

^{1}H NMR (600 MHz, CDCl$_3$)

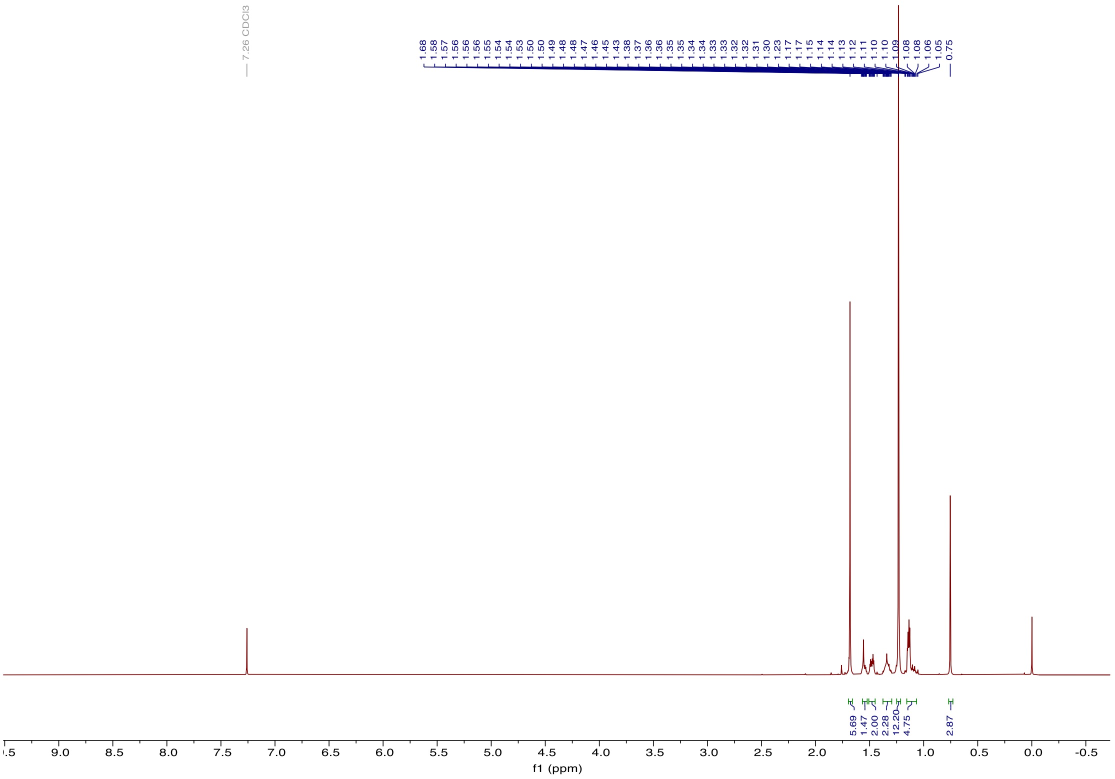

^{13}C NMR (151 MHz, CDCl$_3$)

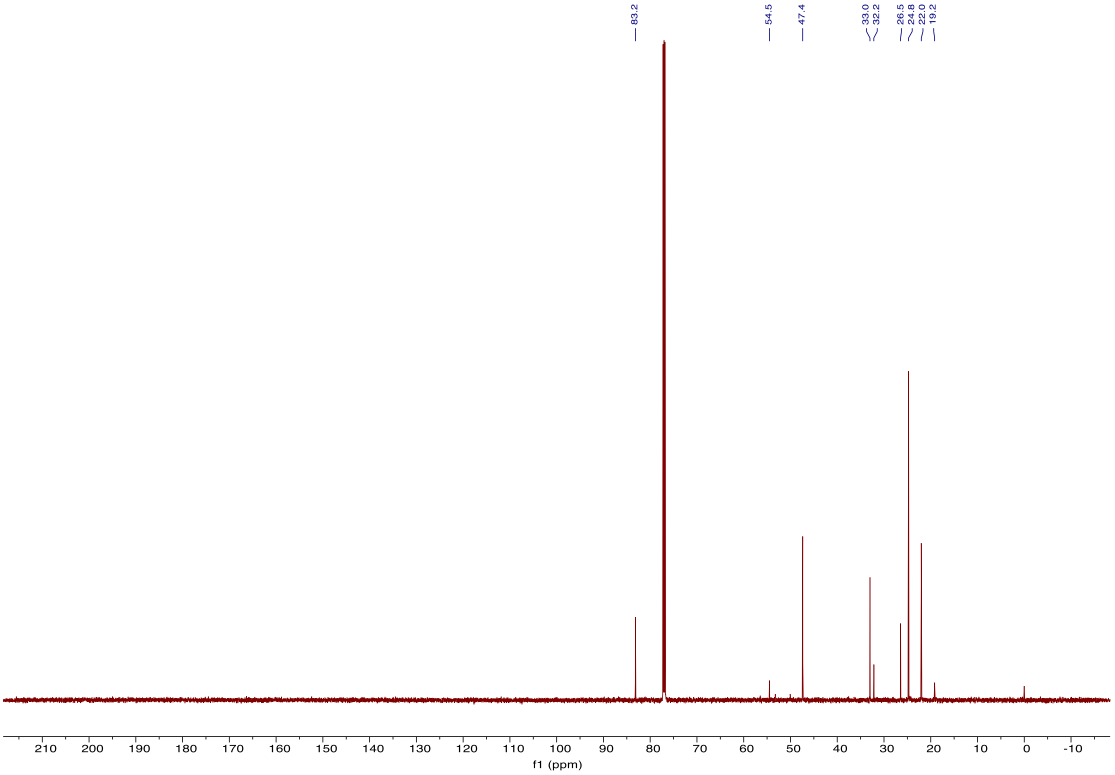

^{11}B NMR (192 MHz, $CDCl_3$)

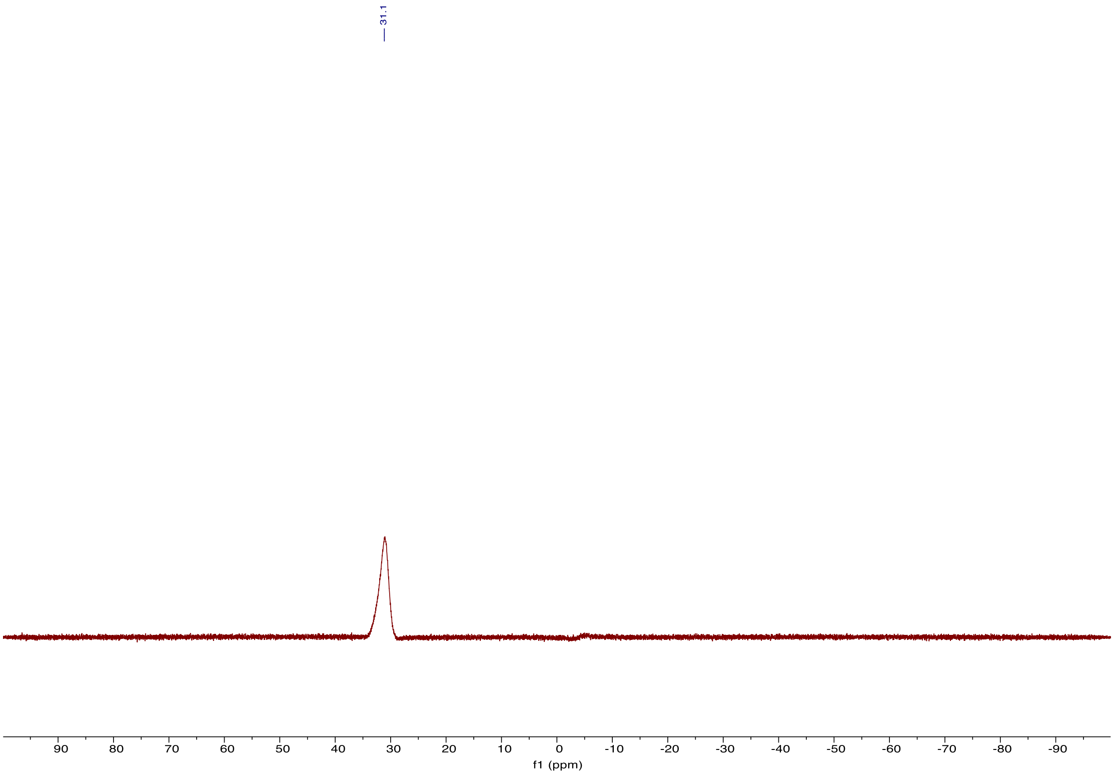

2-(3-(tert-butyl)bicyclo[1.1.1]pentan-1-yl)-4,4,5,5-tetramethyl-1,3,2-dioxaborolane (3k)

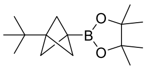

^{1}H NMR (600 MHz, $CDCl_3$)

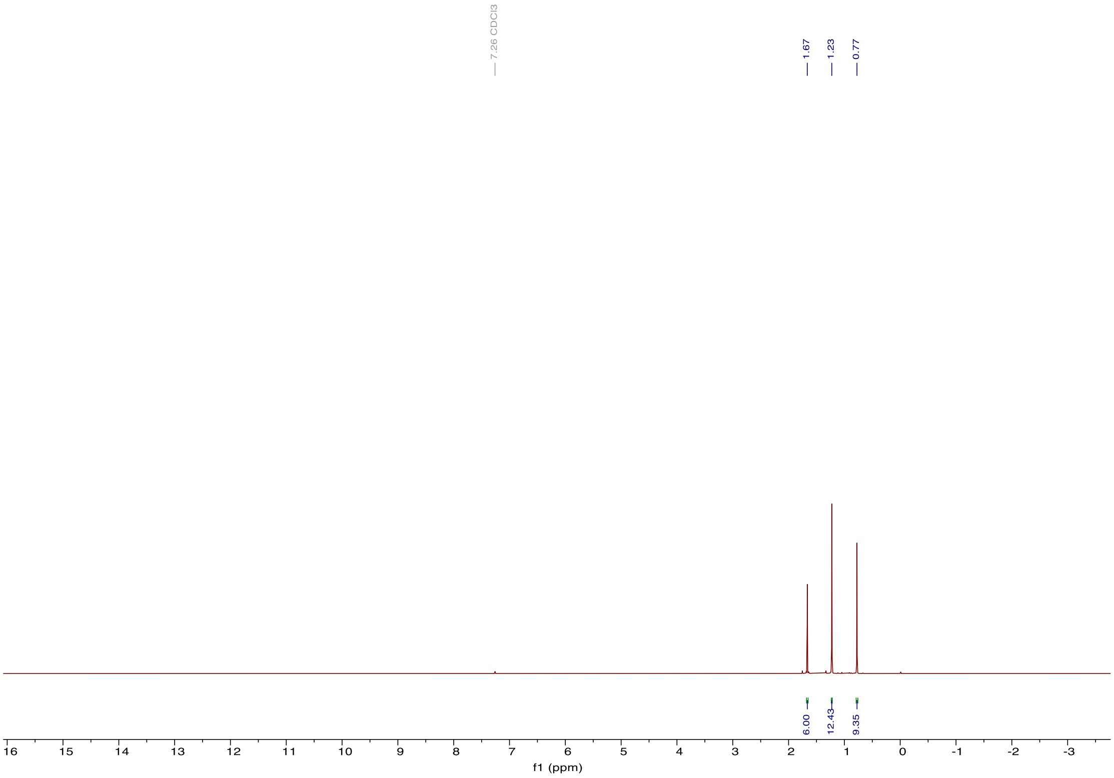

^{13}C NMR (151 MHz, $CDCl_3$)

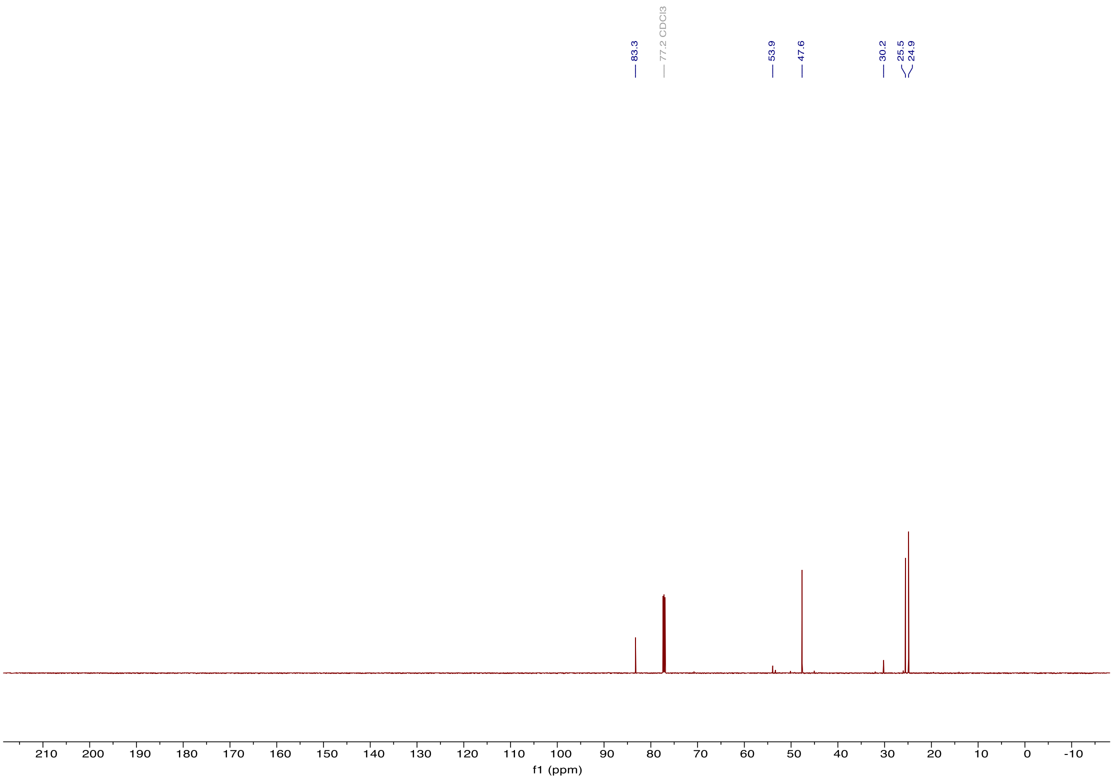

^{11}B NMR (192 MHz, $CDCl_3$)

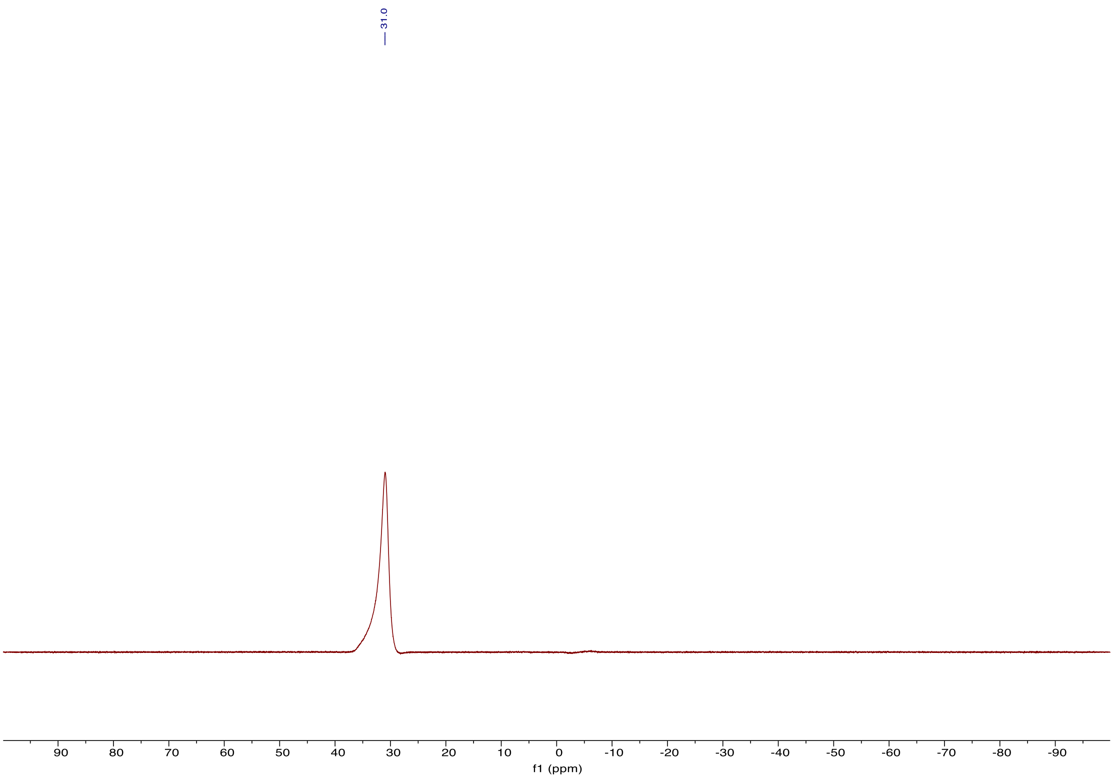

4,4,5,5-tetramethyl-2-(3-(tert-pentyl)bicyclo[1.1.1]pentan-1-yl)-1,3,2-dioxaborolane (3l)

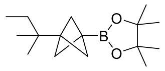

^{1}H NMR (600 MHz, $CDCl_3$)

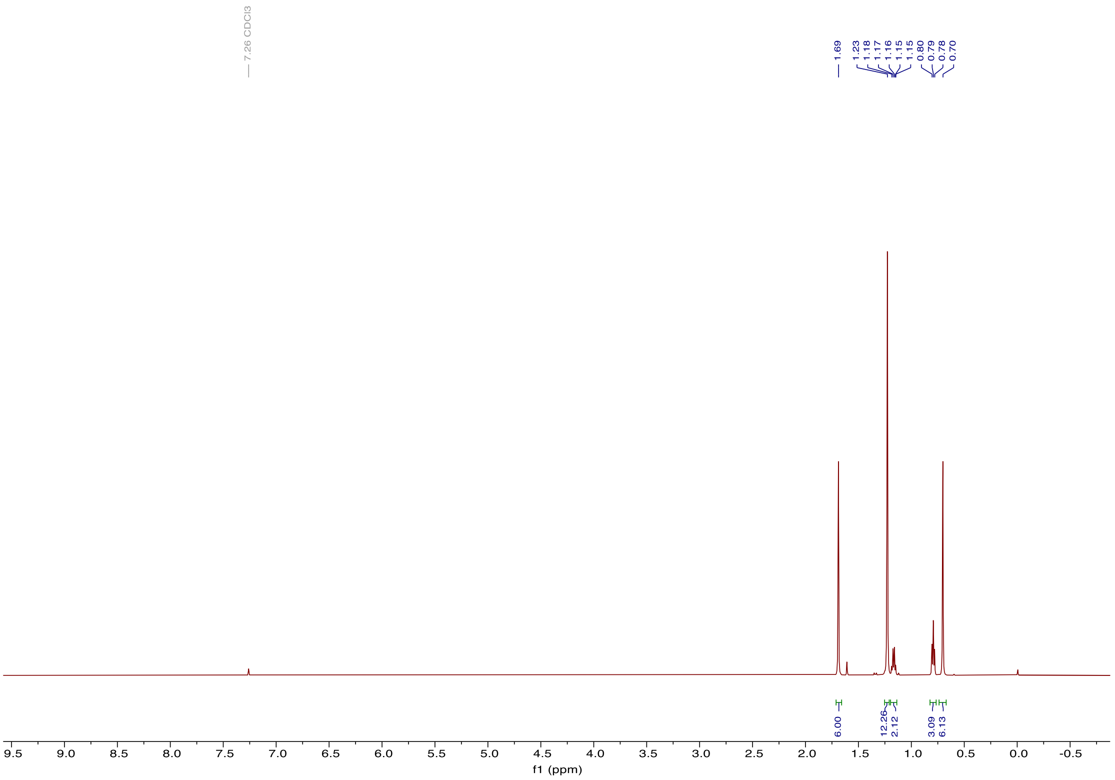

^{13}C NMR (151 MHz, $CDCl_3$)

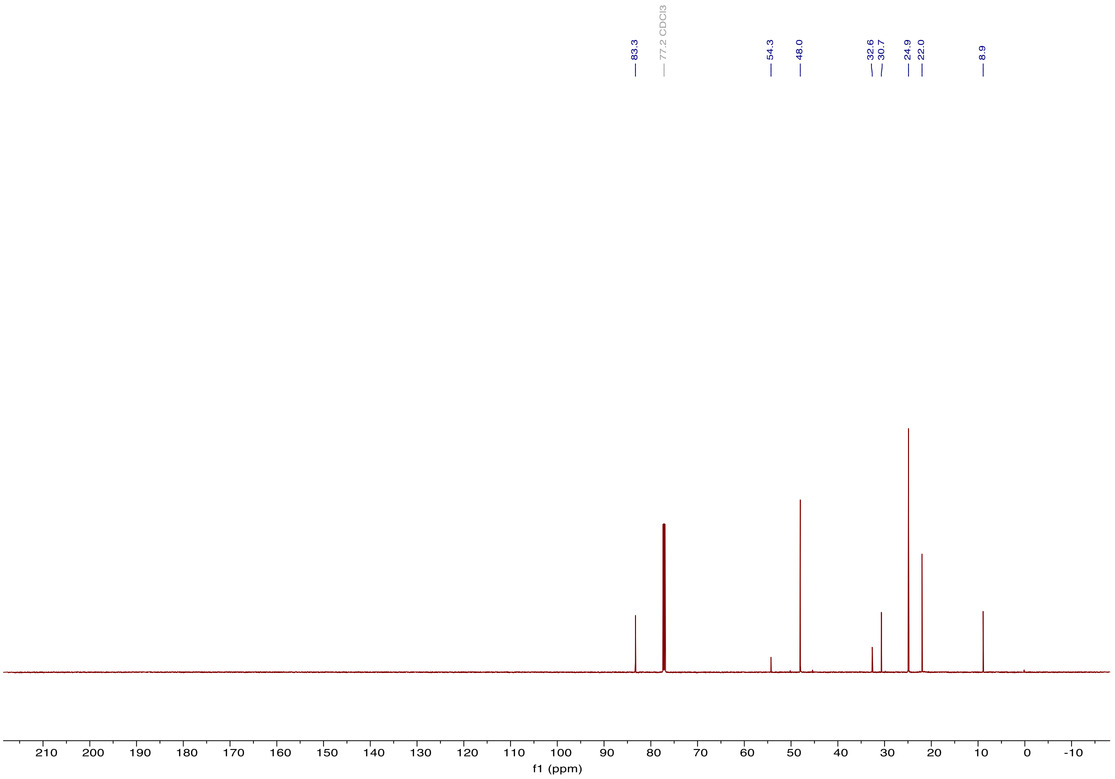

^{11}B NMR (192 MHz, $CDCl_3$)

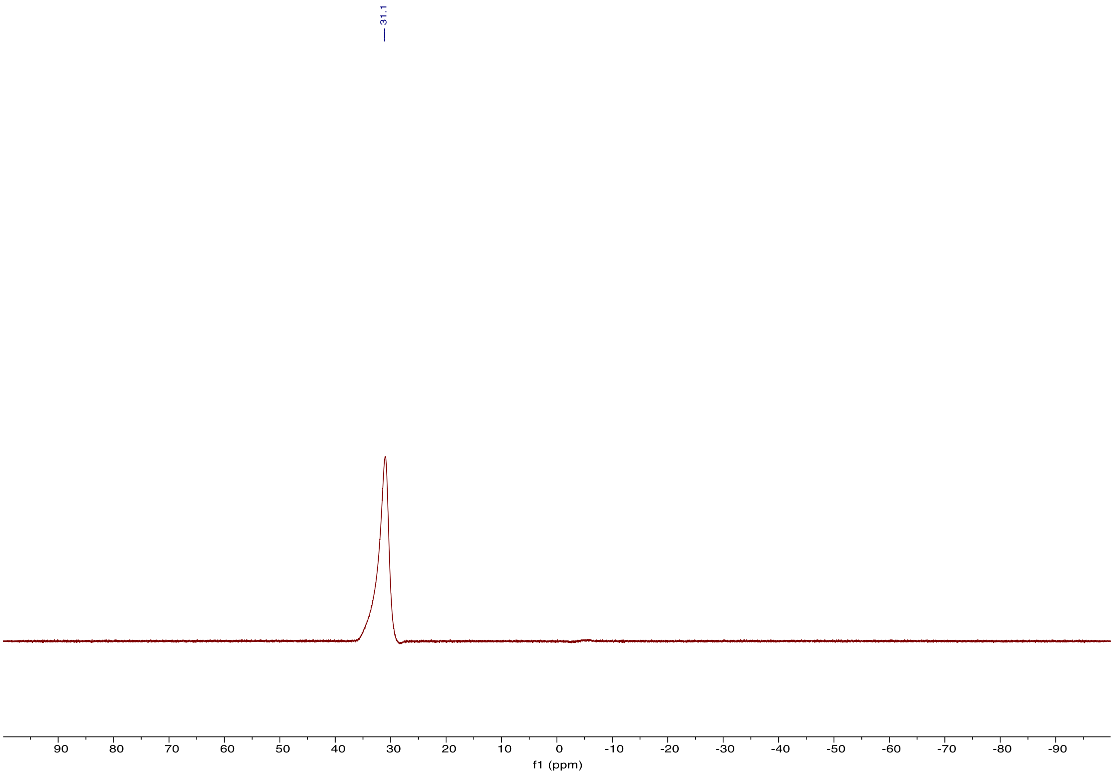

2-(3-(1-fluoro-2-methylpropan-2-yl)bicyclo[1.1.1]pentan-1-yl)-4,4,5,5-tetramethyl-1,3,2-dioxaborolane (3m)

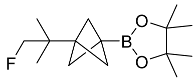

^{1}H NMR (600 MHz, $CDCl_3$)

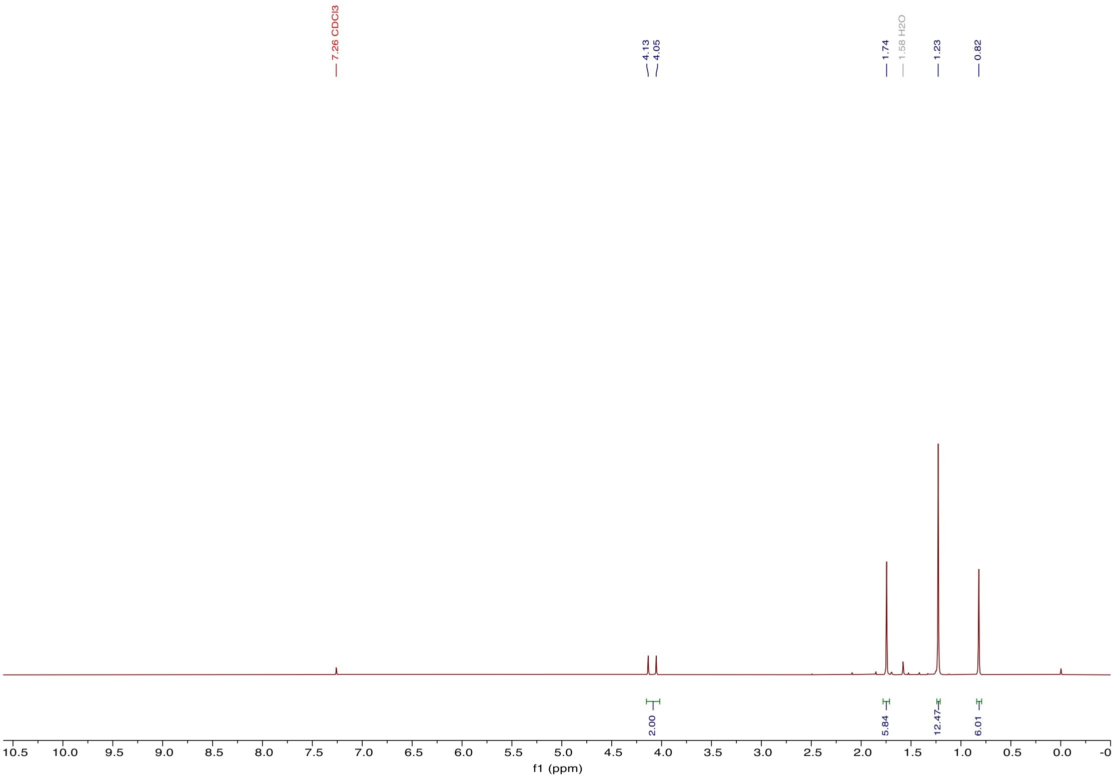

^{13}C NMR (151 MHz, $CDCl_3$)

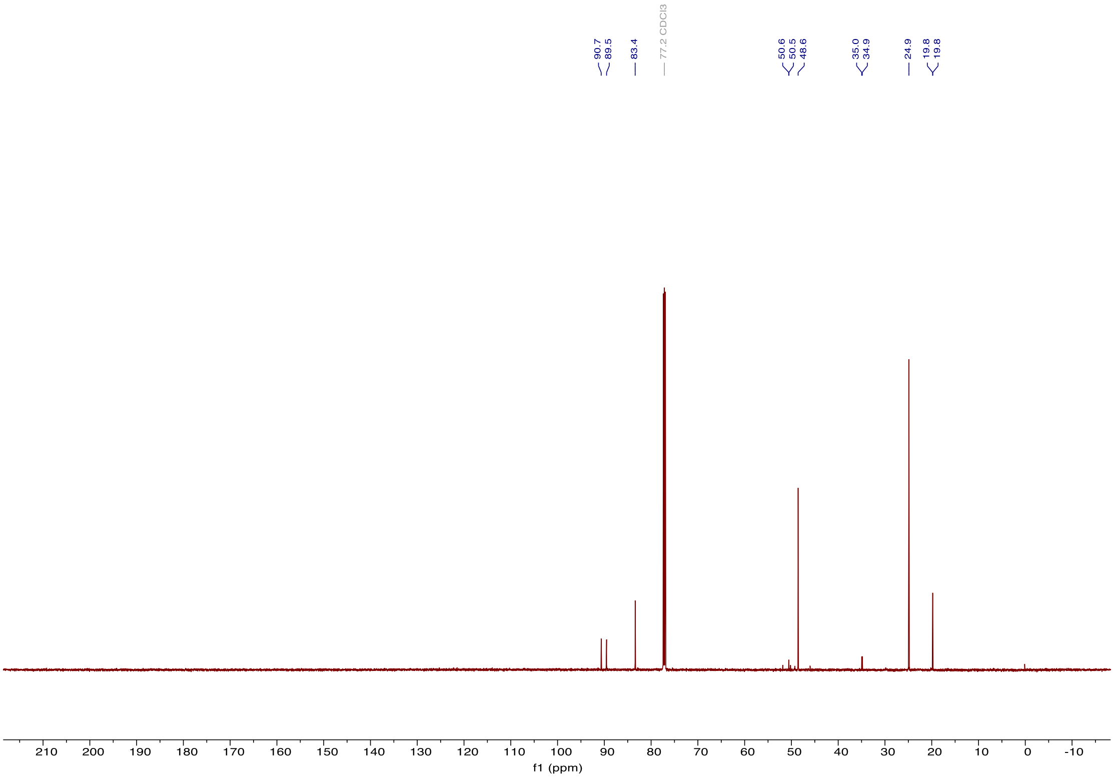

^{11}B NMR (192 MHz, $CDCl_3$)

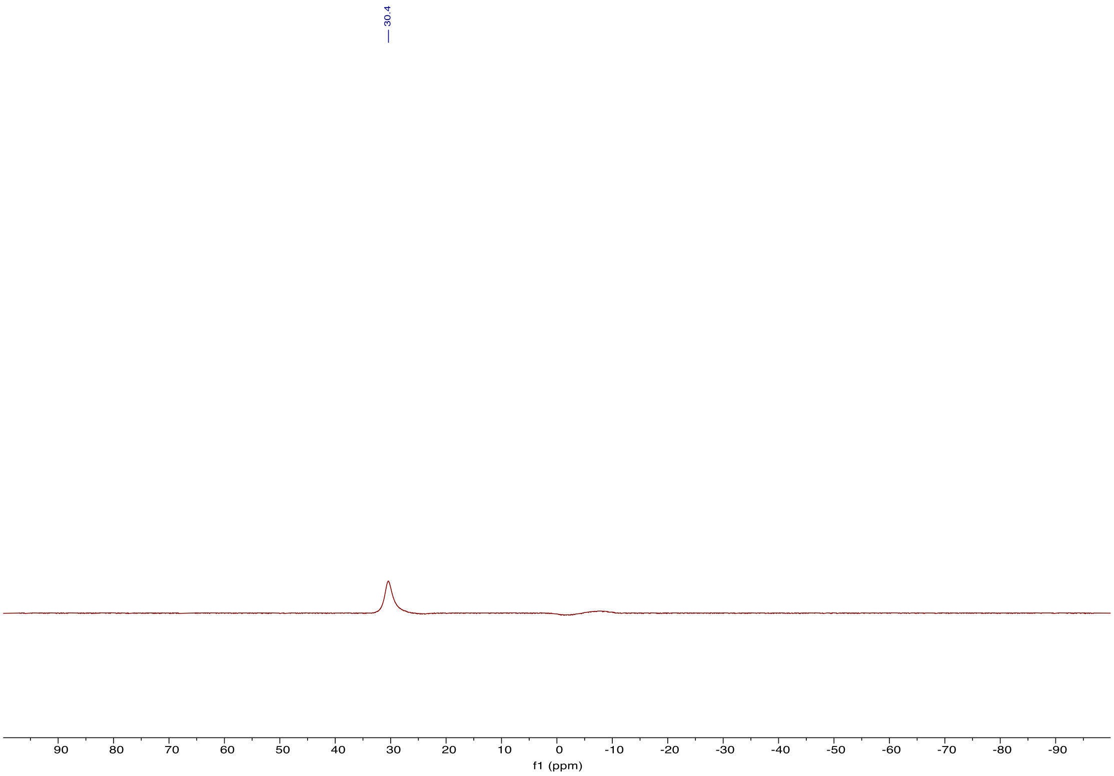

^{19}F NMR (564 MHz, $CDCl_3$)

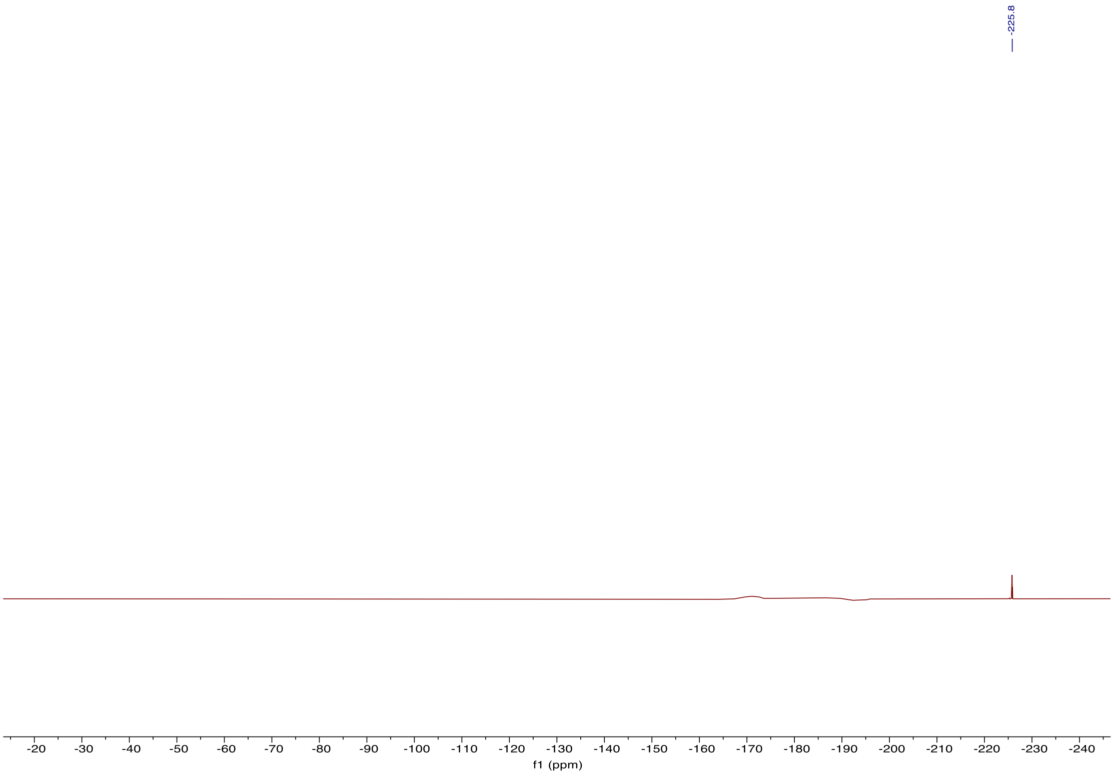

4,4,5,5-tetramethyl-2-(3-(1-methylcyclopropyl)bicyclo[1.1.1]pentan-1-yl)-1,3,2-dioxaborolane (3n)

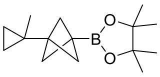

^{1}H NMR (600 MHz, $CDCl_3$)

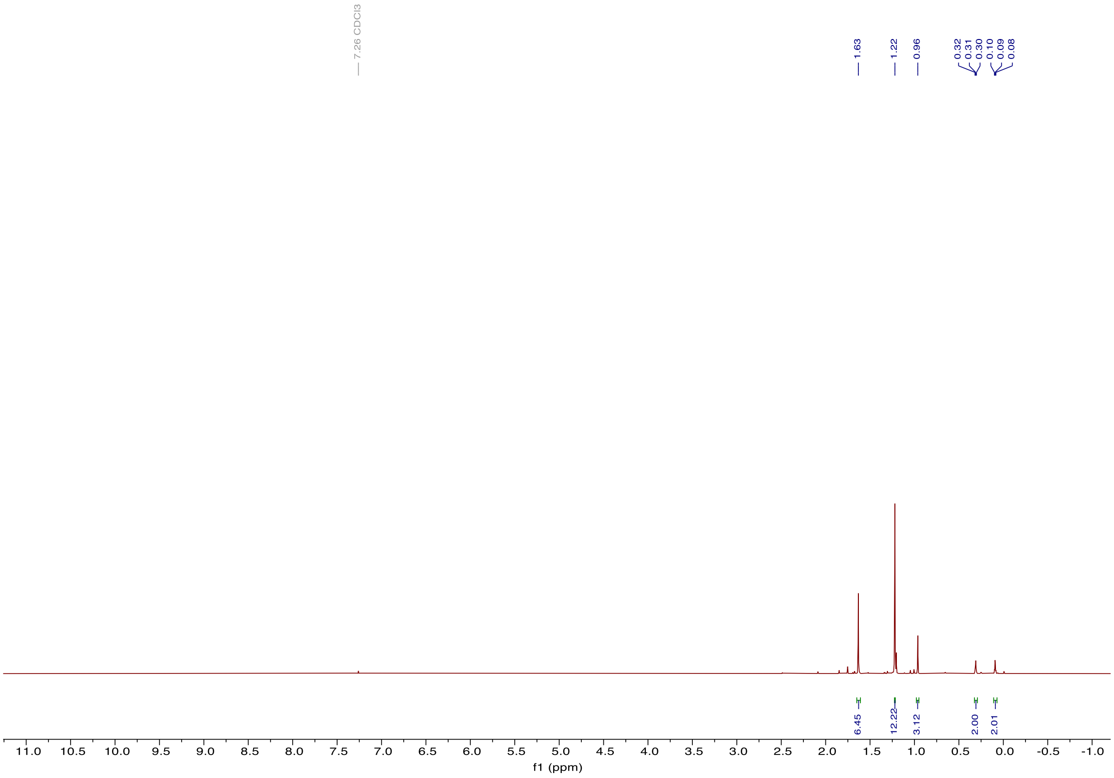

^{13}C NMR (151 MHz, $CDCl_3$)

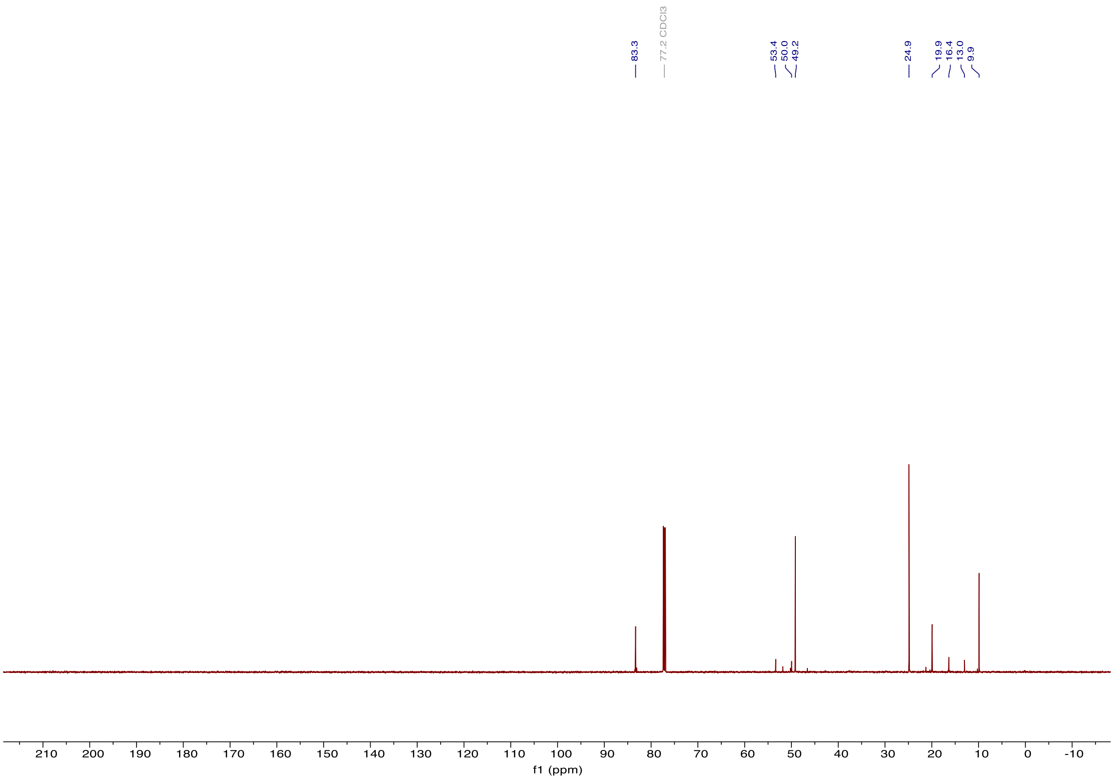

^{11}B NMR (192 MHz, $CDCl_3$)

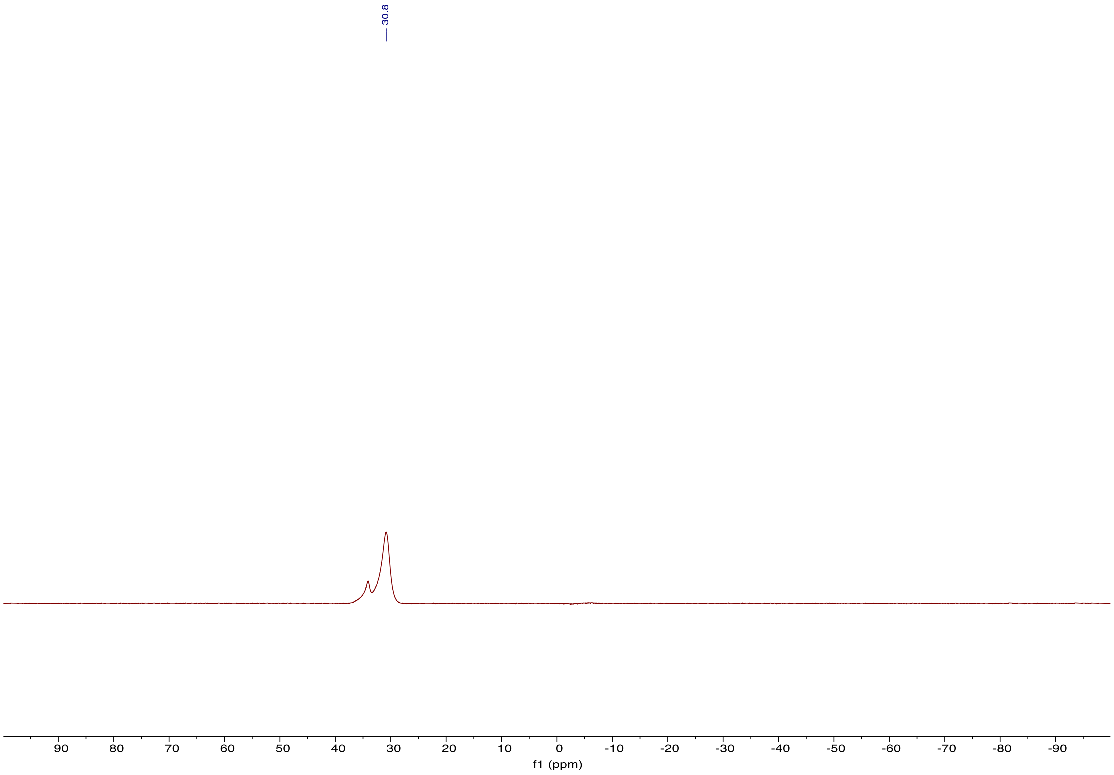

4,4,5,5-tetramethyl-2-(3-(4-methyltetrahydro-2H-pyran-4-yl)bicyclo[1.1.1]pentan-1-yl)-1,3,2-dioxaborolane (3o)

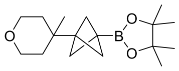

^{1}H NMR (600 MHz, $CDCl_3$)

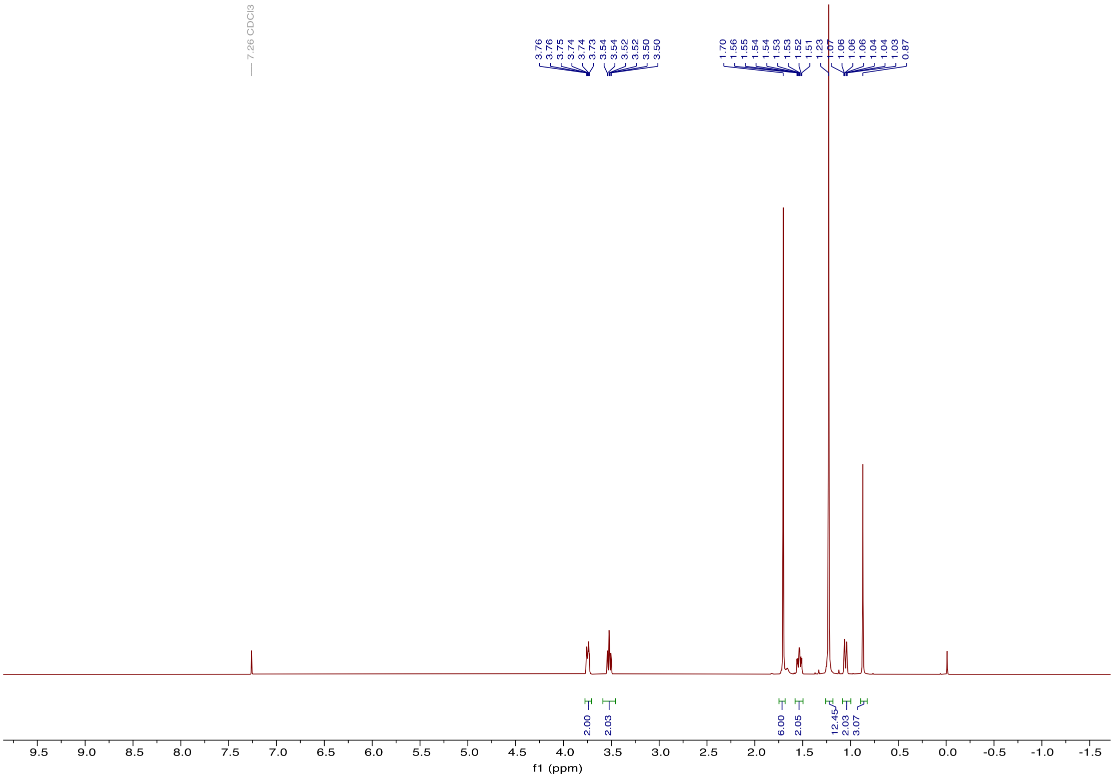

^{13}C NMR (151 MHz, $CDCl_3$)

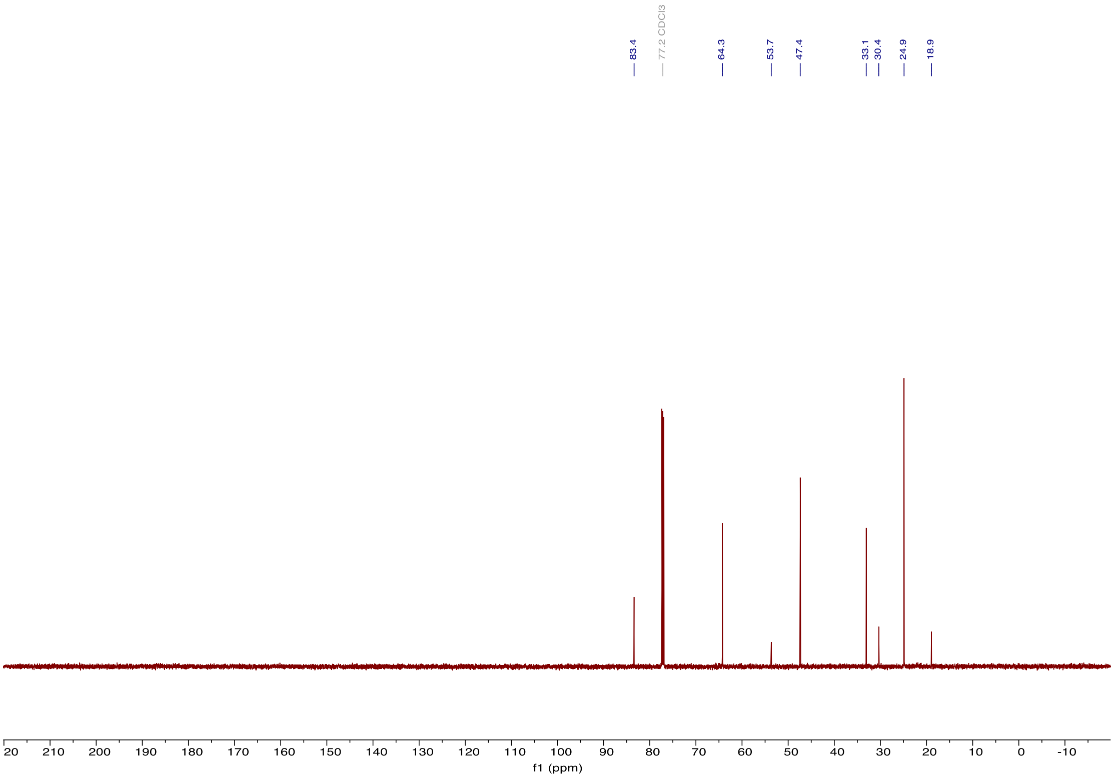

^{11}B NMR (192 MHz, CDCl$_3$)

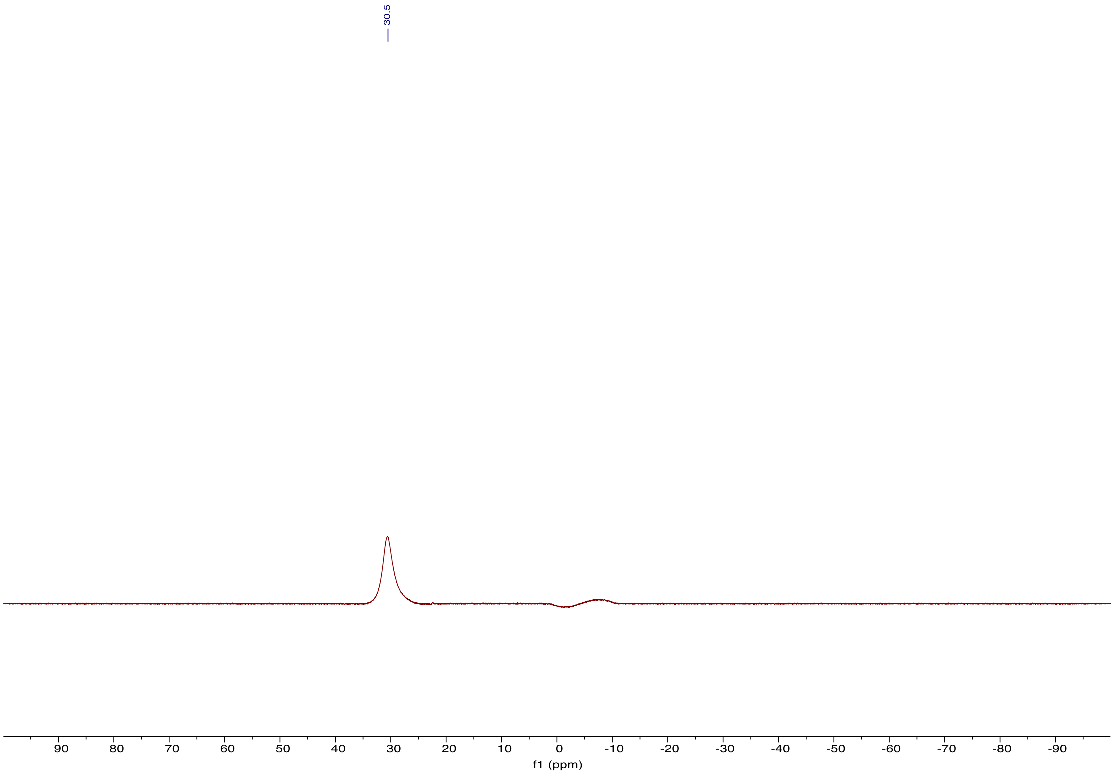

4,4,5,5-tetramethyl-2-(3-(3-methyloxetan-3-yl)bicyclo[1.1.1]pentan-1-yl)-1,3,2-dioxaborolane (3p)

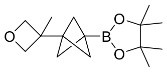

^{1}H NMR (600 MHz, CDCl$_3$)

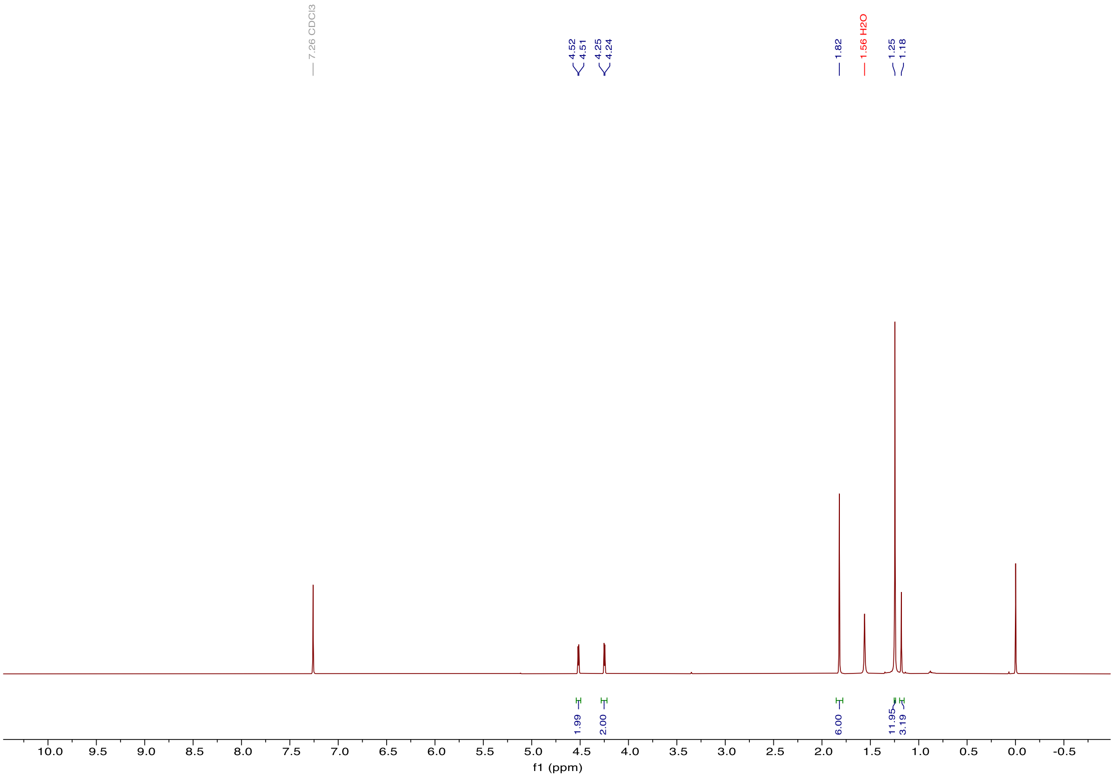

^{13}C NMR (151 MHz, $CDCl_3$)

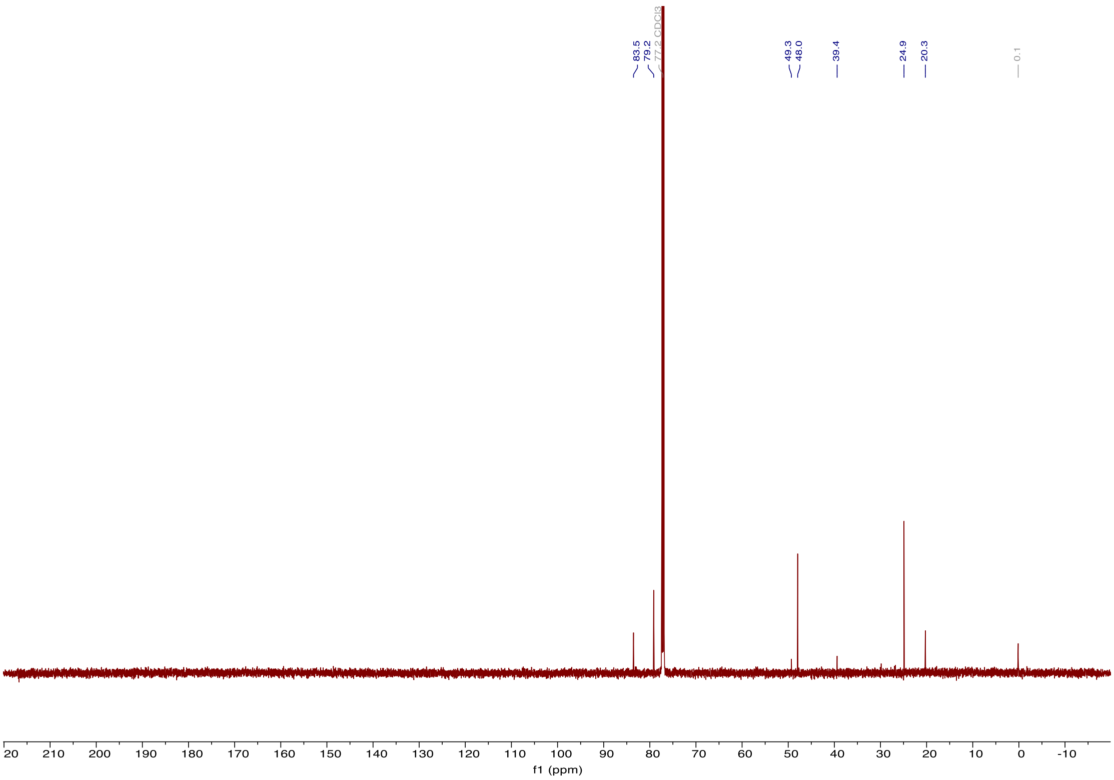

^{11}B NMR (192 MHz, $CDCl_3$)

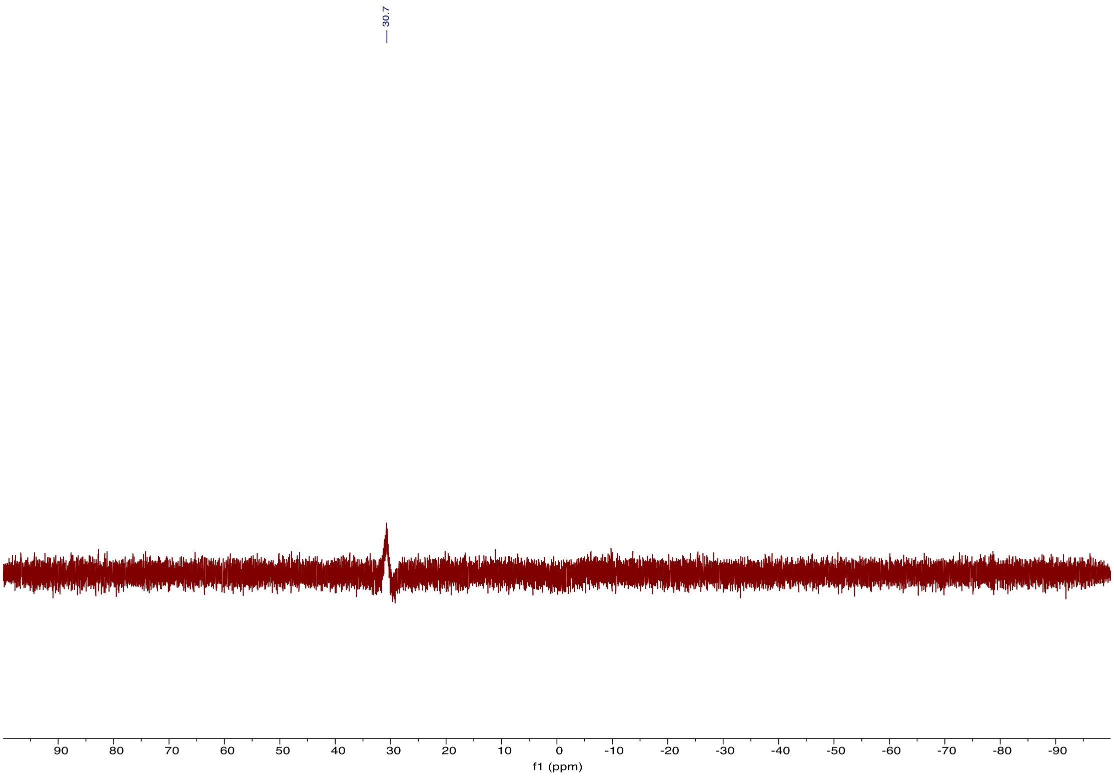

tert-butyl 4-methyl-4-(3-(4,4,5,5-tetramethyl-1,3,2-dioxaborolan-2-yl)bicyclo[1.1.1]pentan-1-yl)piperidine-1-carboxylate (3q)

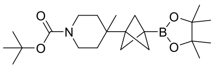

^{1}H NMR (600 MHz, CDCl$_3$)

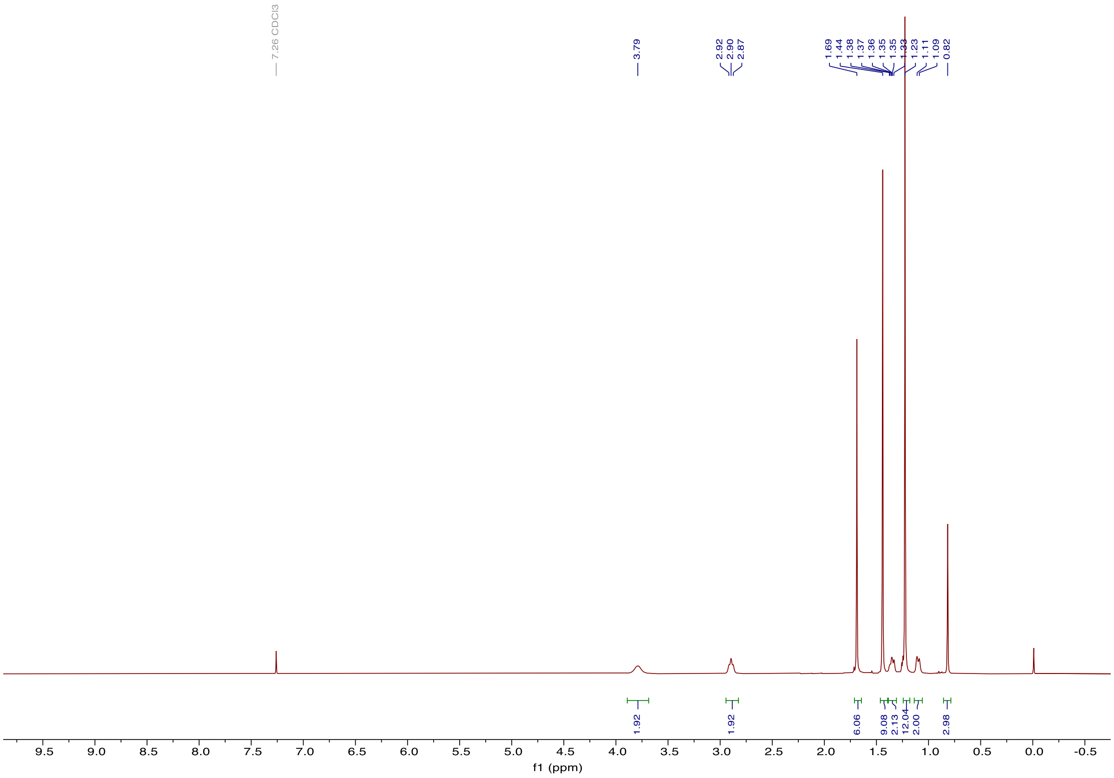

^{13}C NMR (151 MHz, CDCl$_3$)

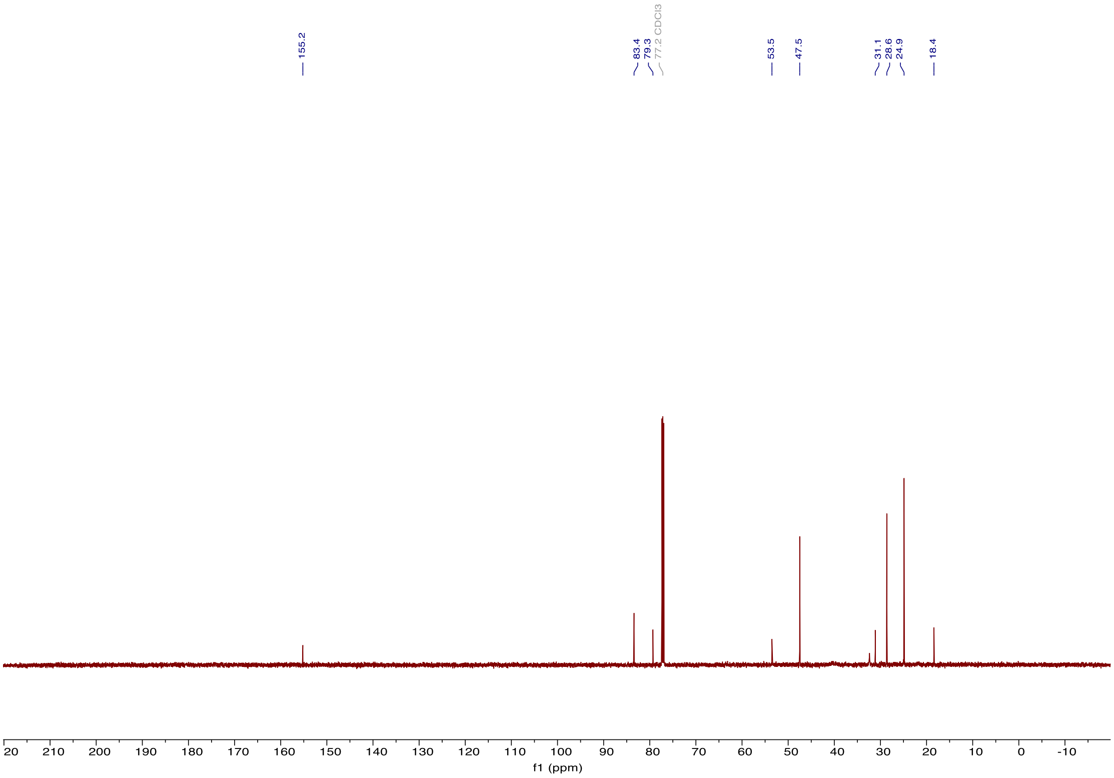

^{11}B NMR (192 MHz, $CDCl_3$)

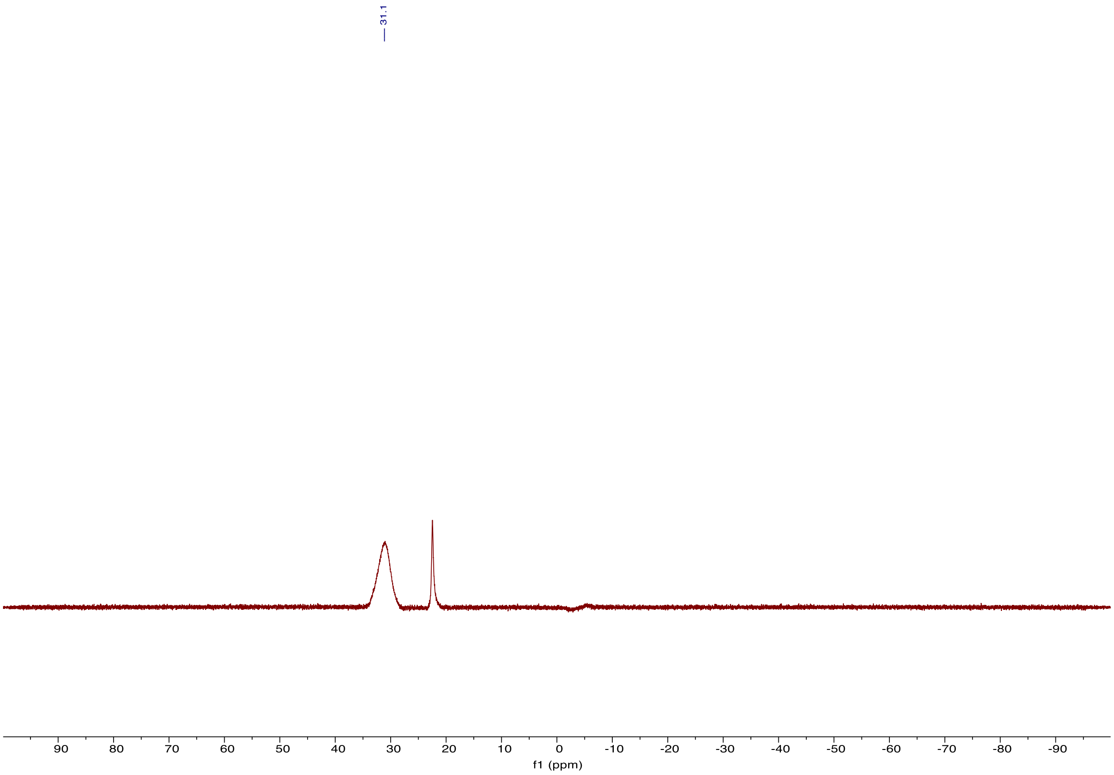

2-(3'-fluoro-[1,1'-bi(bicyclo[1.1.1]pentan)]-3-yl)-4,4,5,5-tetramethyl-1,3,2-dioxaborolane (3r)

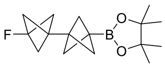

^{1}H NMR (600 MHz, $CDCl_3$)

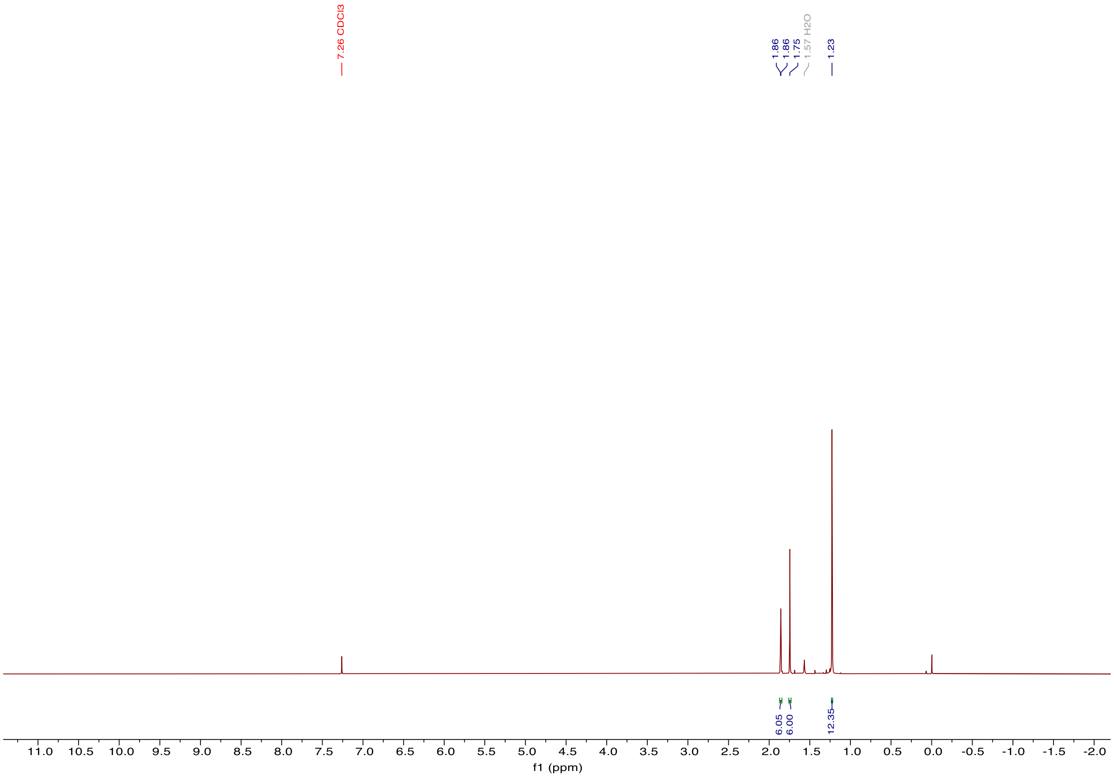

^{13}C NMR (151 MHz, $CDCl_3$)

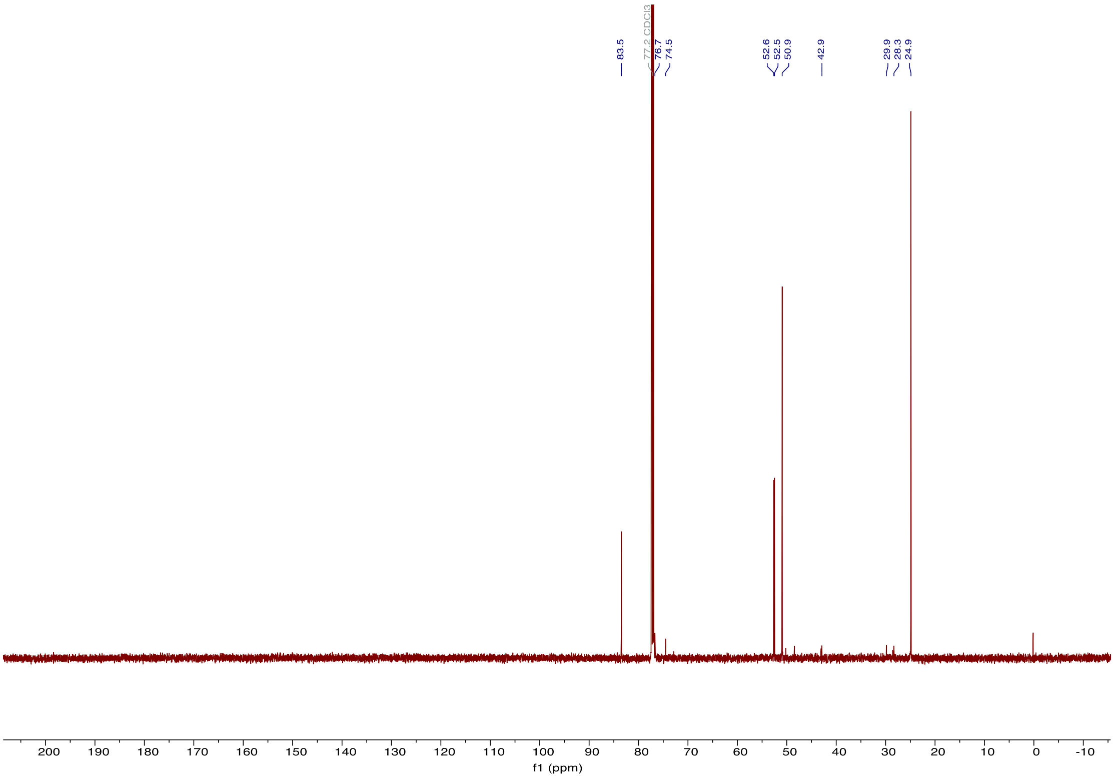

^{11}B NMR (192 MHz, $CDCl_3$)

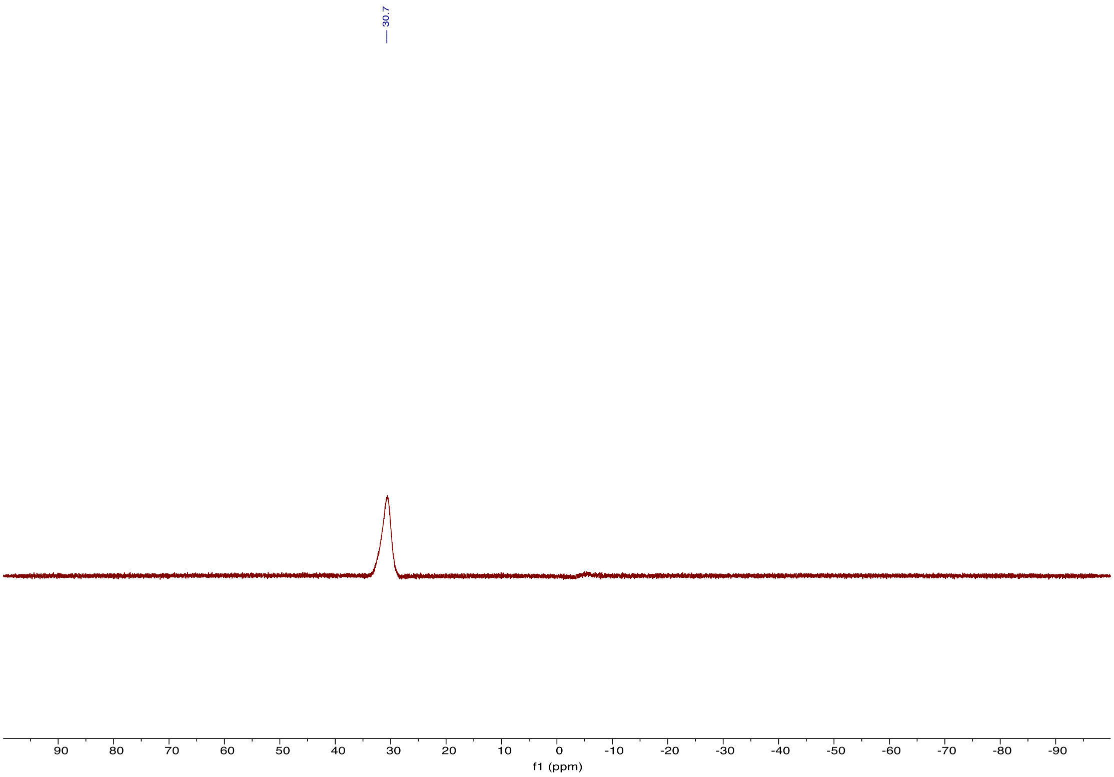

^{19}F NMR (564 MHz, $CDCl_3$)

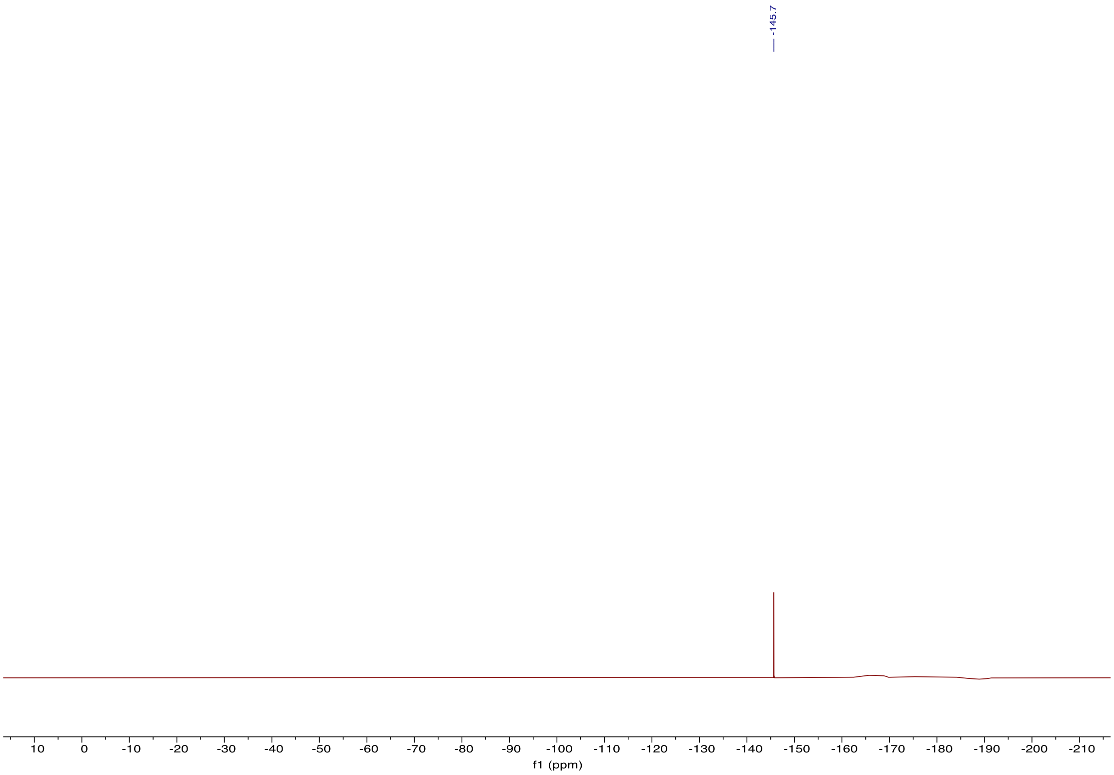

2-(3-((2R,3as,5S,6as)-hexahydro-2,5-methanopentalen-3a(1H)-yl)bicyclo[1.1.1]pentan-1-yl)-4,4,5,5-tetramethyl-1,3,2-dioxaborolane (3s)

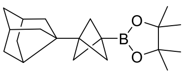

^{1}H NMR (600 MHz, $CDCl_3$)

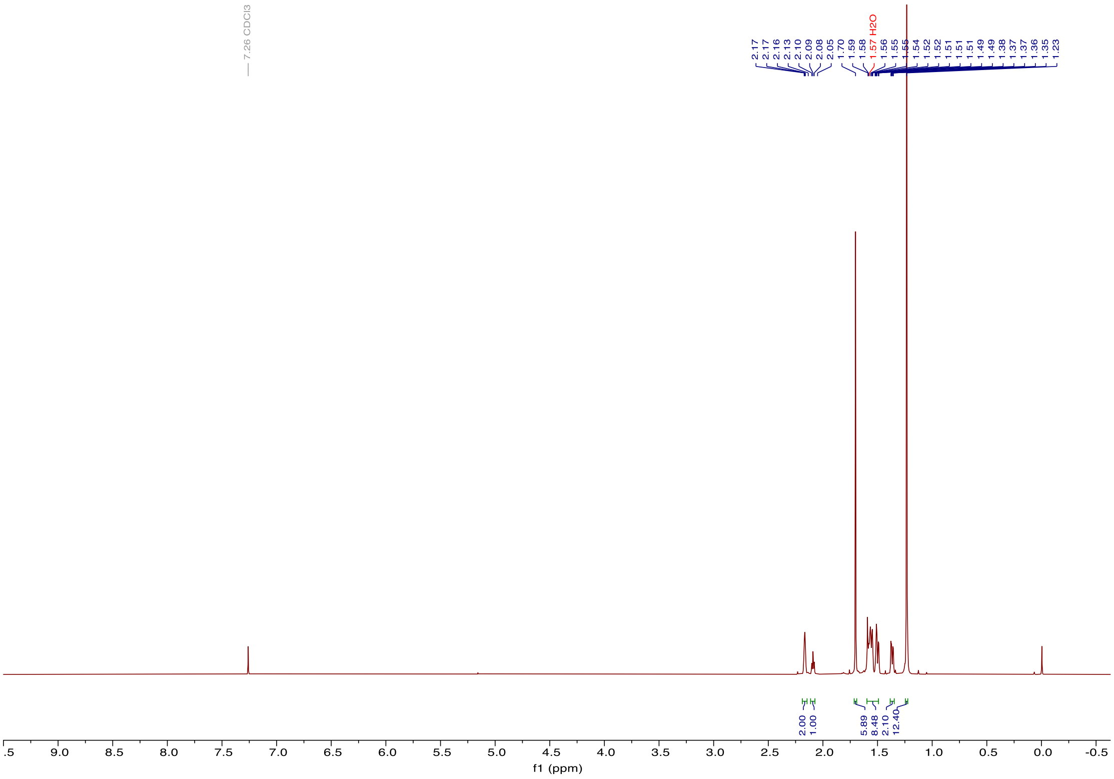

^{13}C NMR (151 MHz, $CDCl_3$)

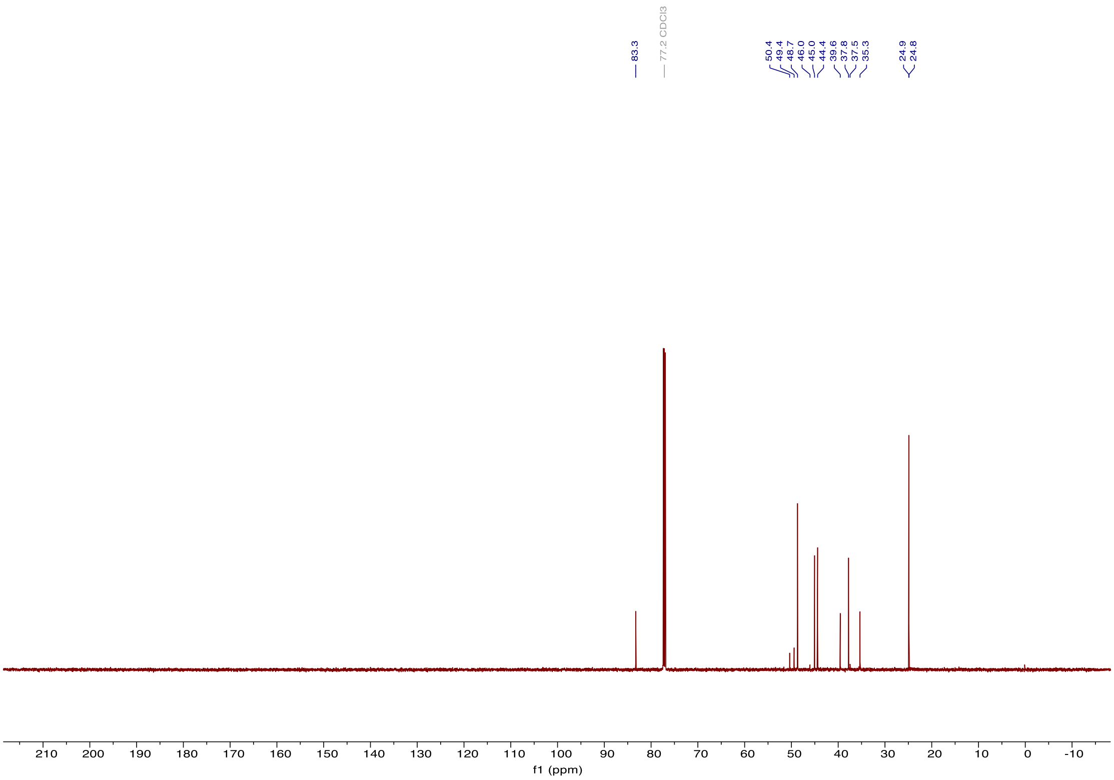

^{11}B NMR (192 MHz, $CDCl_3$)

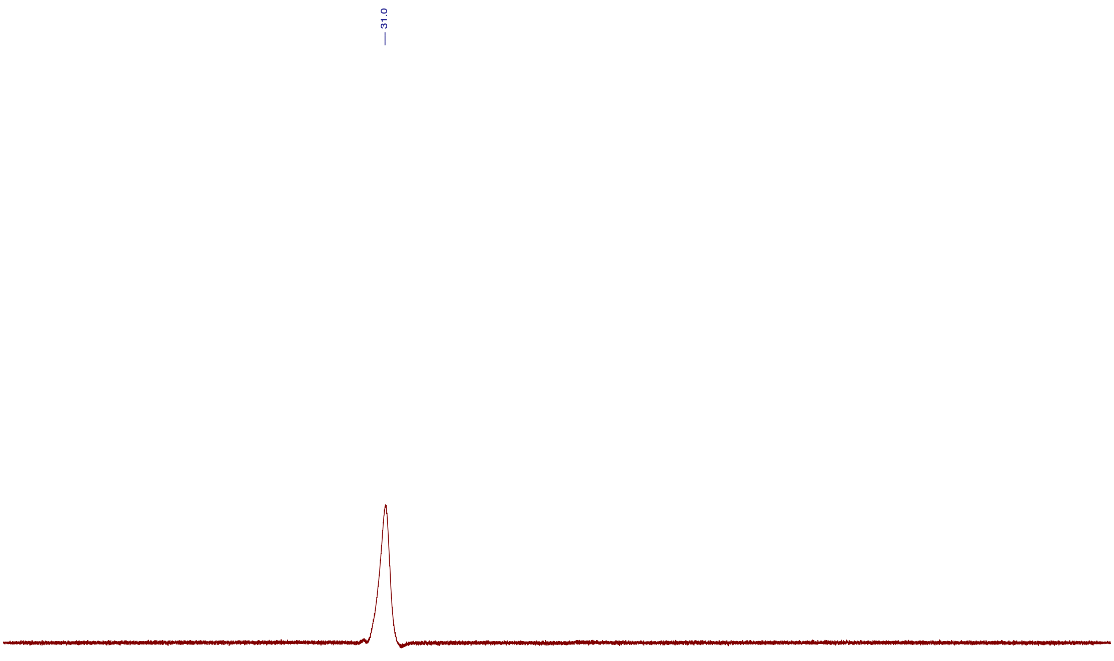

2-(3-((1r,3R,5S,7r)-3,5-dimethyladamantan-1-yl)bicyclo[1.1.1]pentan-1-yl)-4,4,5,5-tetramethyl-1,3,2-dioxaborolane (3t)

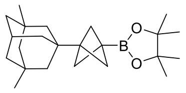

^{1}H NMR (600 MHz, $CDCl_3$)

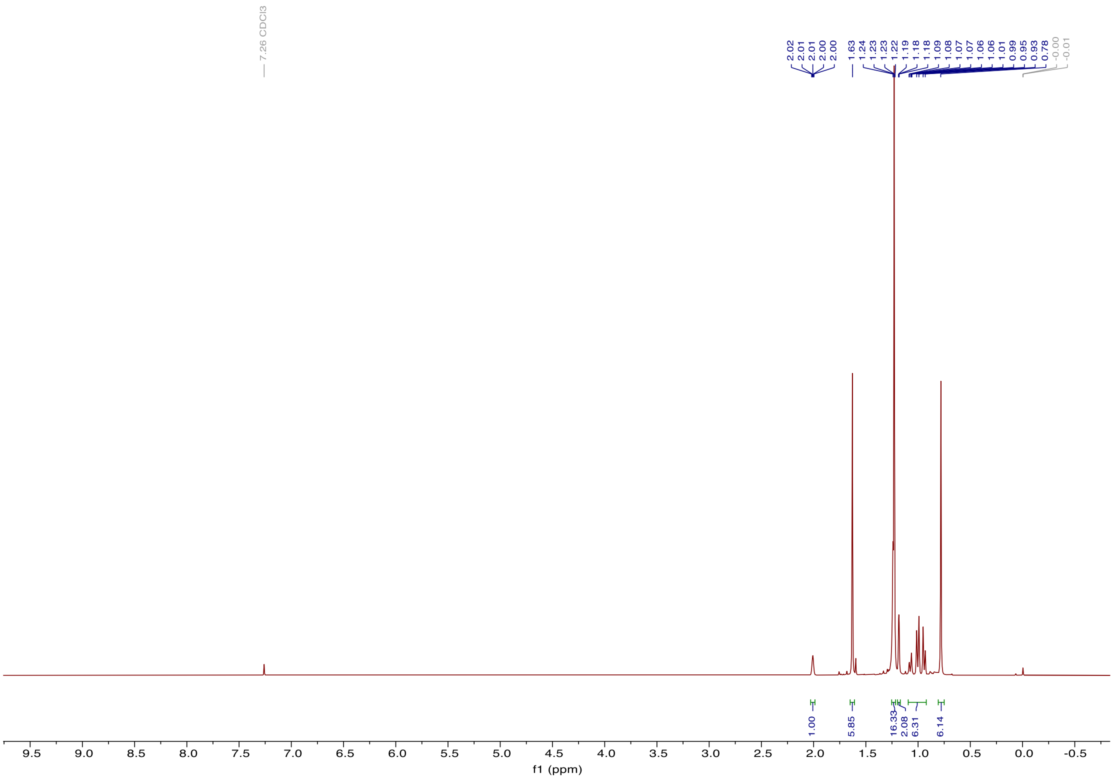

^{13}C NMR (151 MHz, $CDCl_3$)

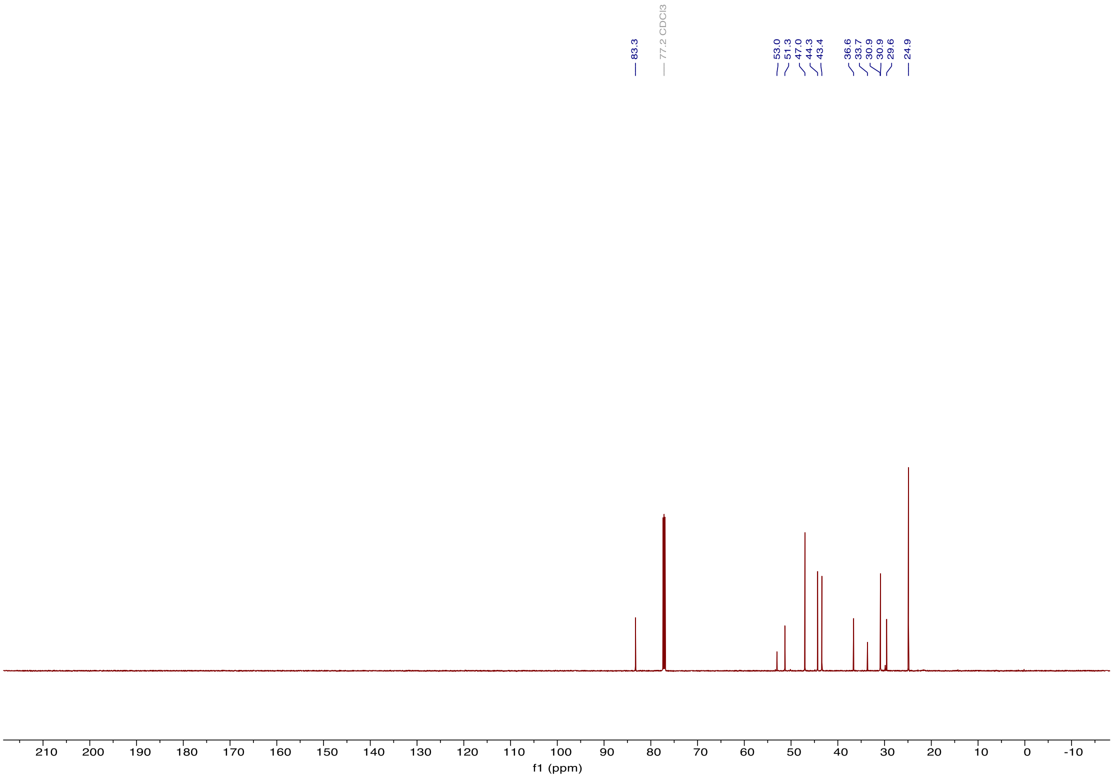

^{11}B NMR (192 MHz, $CDCl_3$)

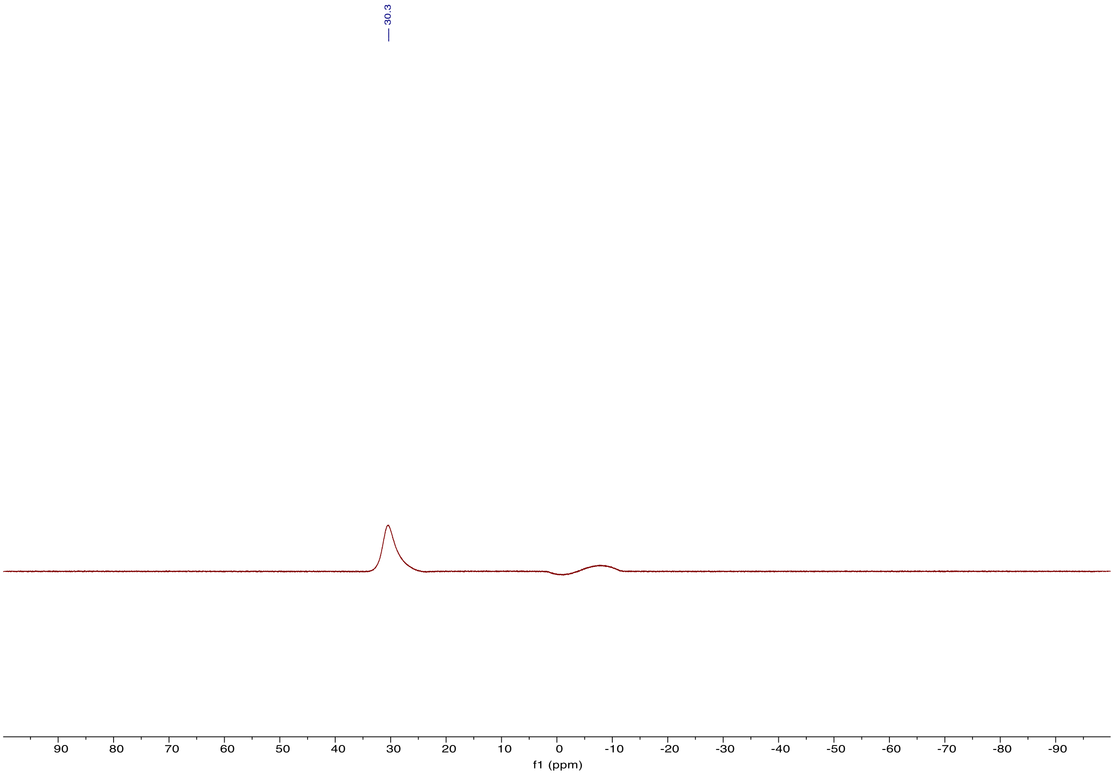

4,4,5,5-tetramethyl-2-(3-(2-phenoxypropan-2-yl)bicyclo[1.1.1]pentan-1-yl)-1,3,2-dioxaborolane (3u)

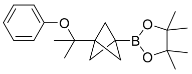

^{1}H NMR (600 MHz, $CDCl_3$)

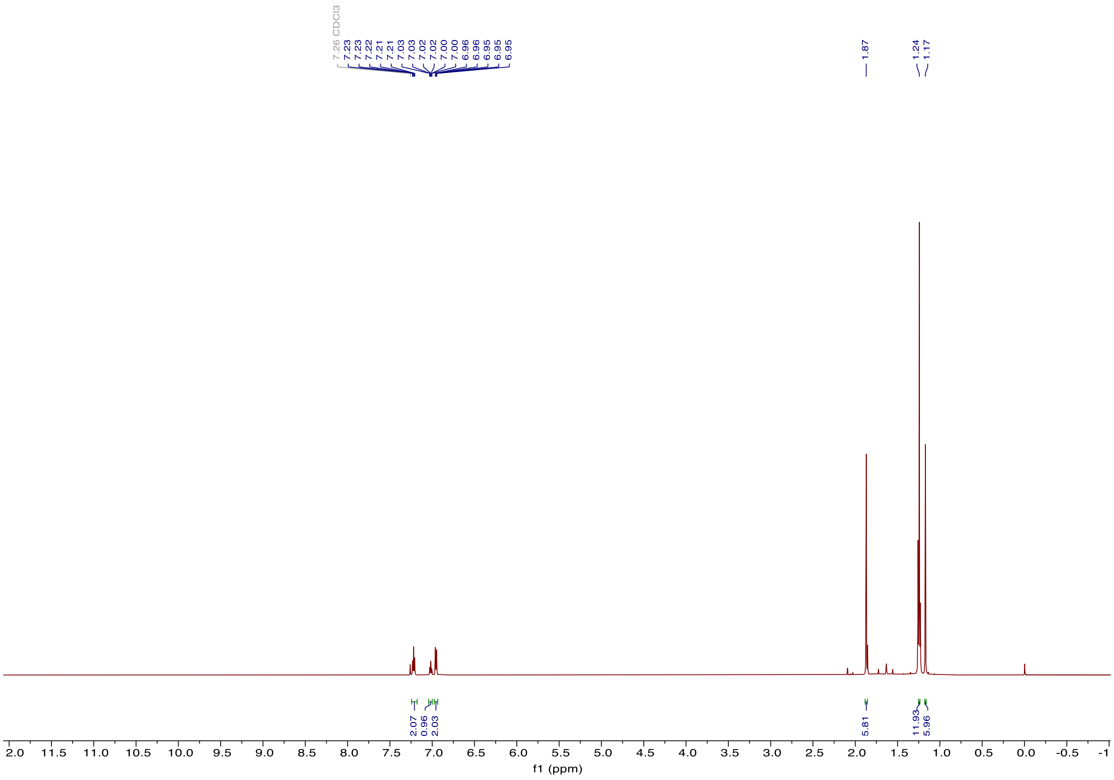

^{13}C NMR (151 MHz, $CDCl_3$)

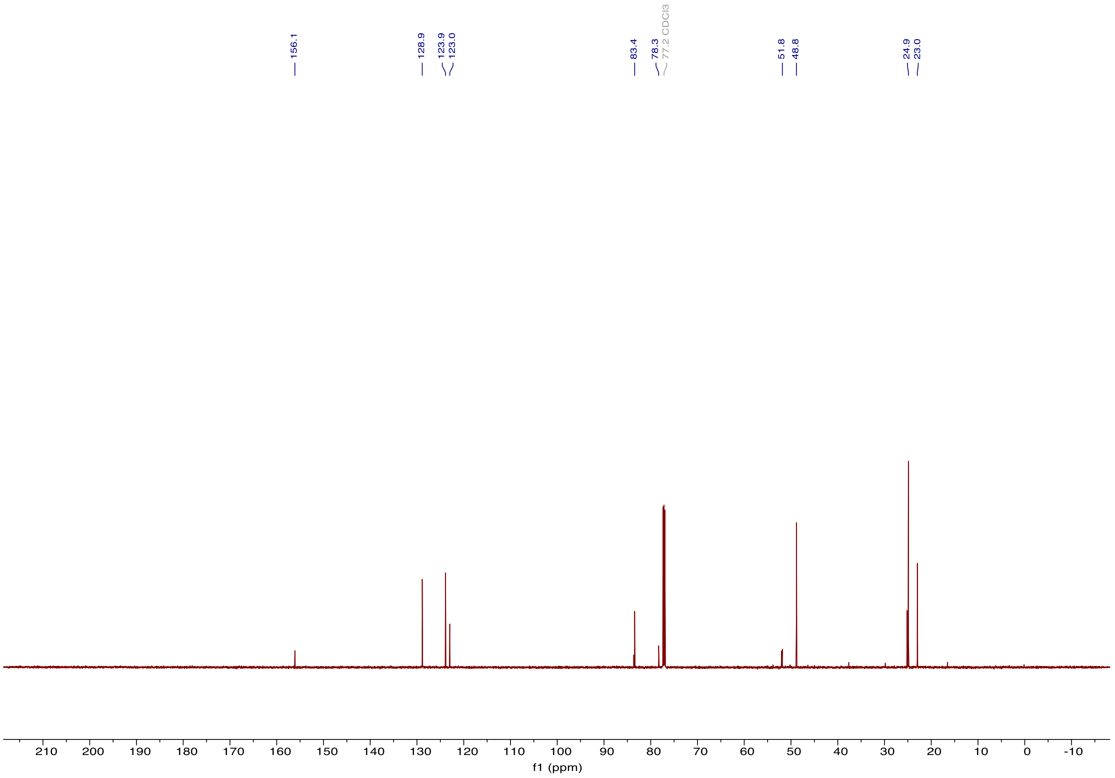

^{11}B NMR (192 MHz, $CDCl_3$)

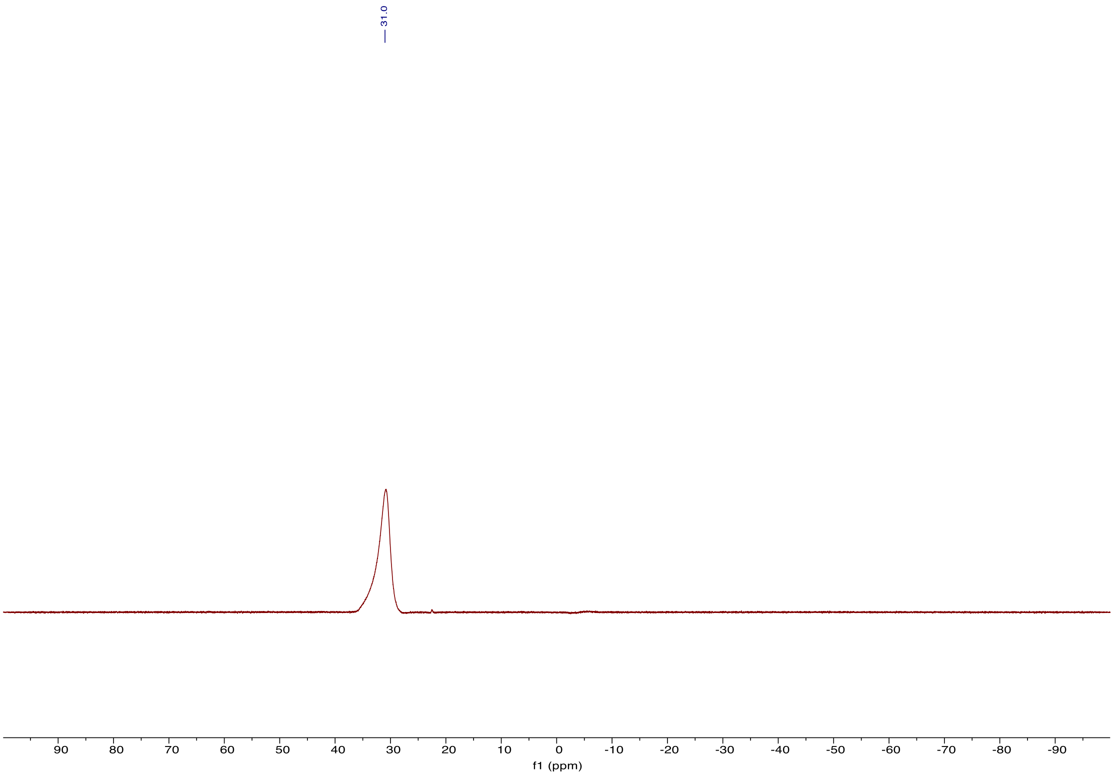

2-(3-(2-(4-chlorophenoxy)propan-2-yl)bicyclo[1.1.1]pentan-1-yl)-4,4,5,5-tetramethyl-1,3,2-dioxaborolane (3v)

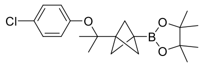

^{1}H NMR (600 MHz, $CDCl_3$)

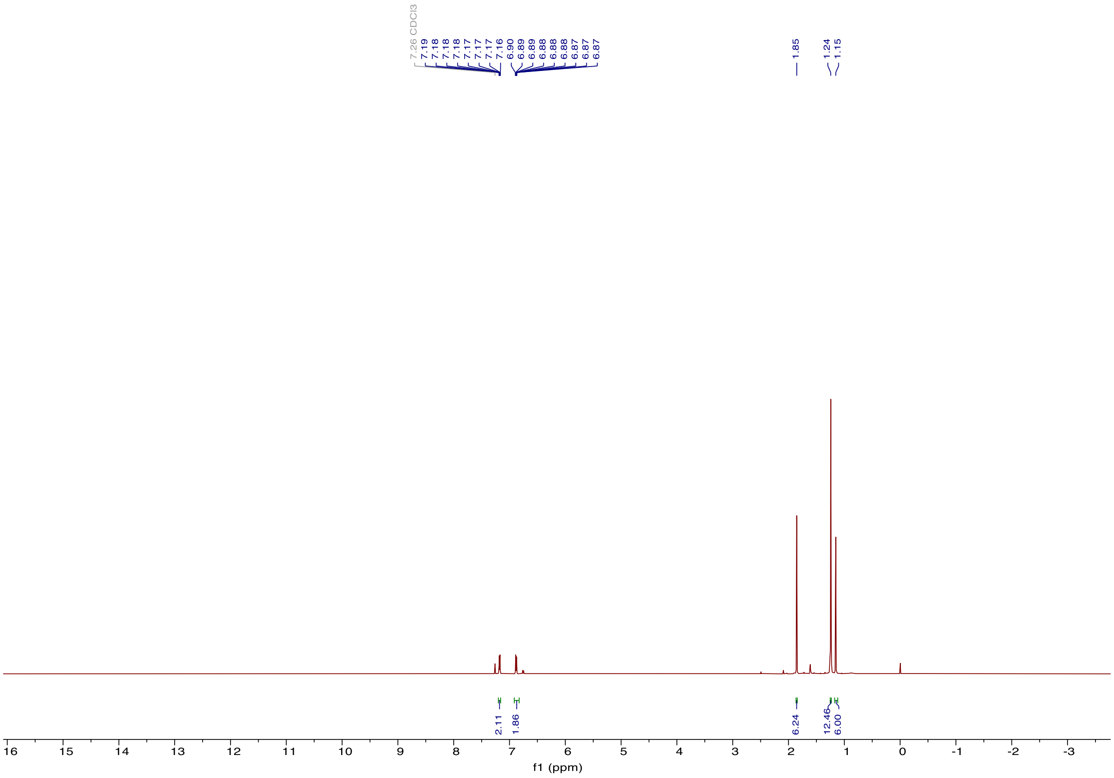

^{13}C NMR (151 MHz, $CDCl_3$)

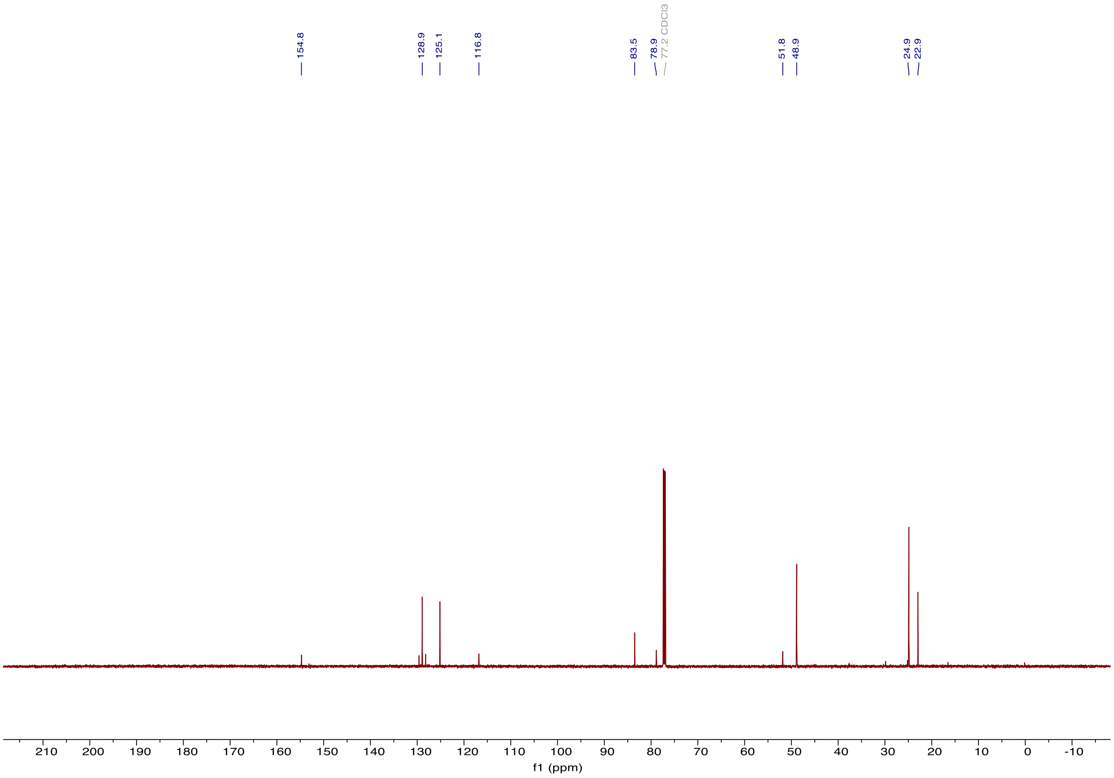

^{11}B NMR (192 MHz, $CDCl_3$)

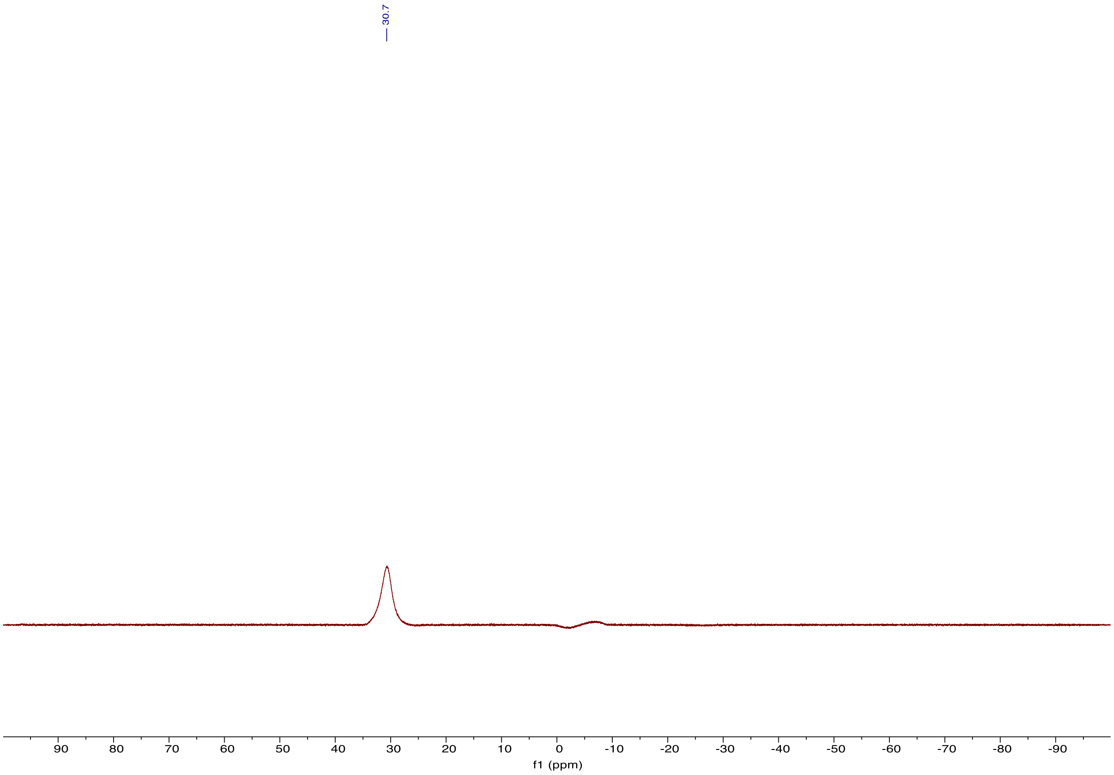

4,4,5,5-tetramethyl-2-(3-(4-(methylthio)benzyl)bicyclo[1.1.1]pentan-1-yl)-1,3,2-dioxaborolane (3w)

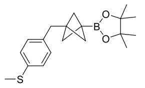

^{1}H NMR (600 MHz, $CDCl_3$)

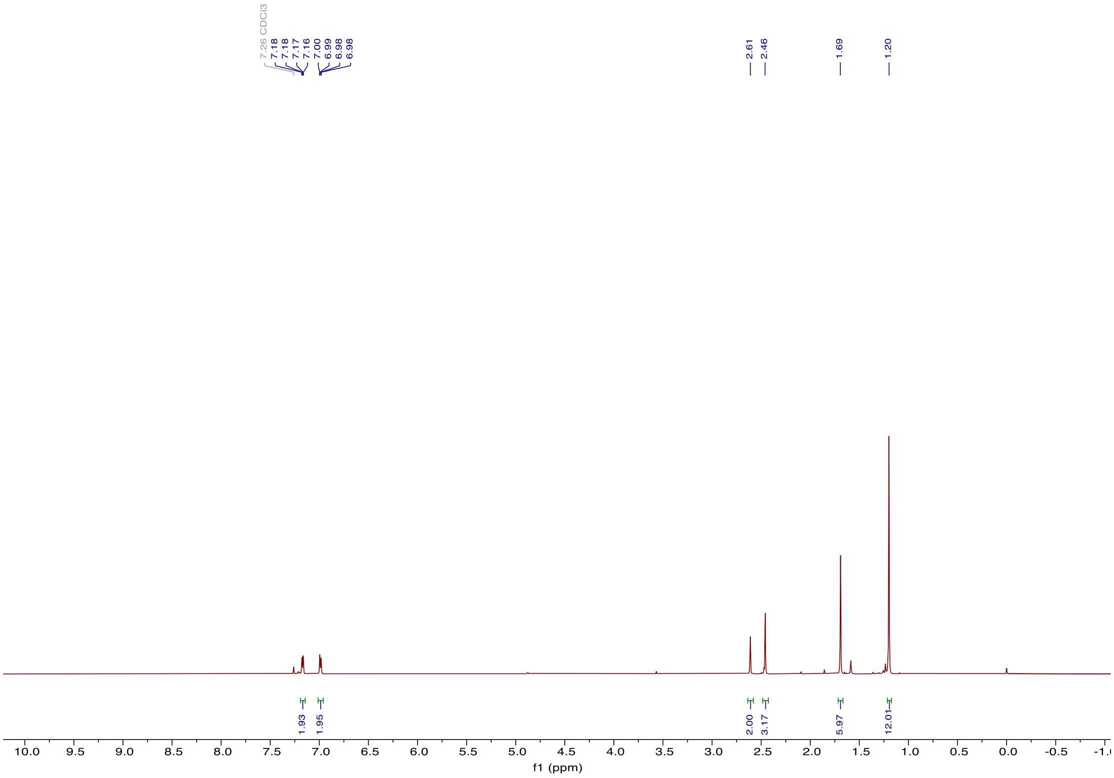

^{13}C NMR (151 MHz, $CDCl_3$)

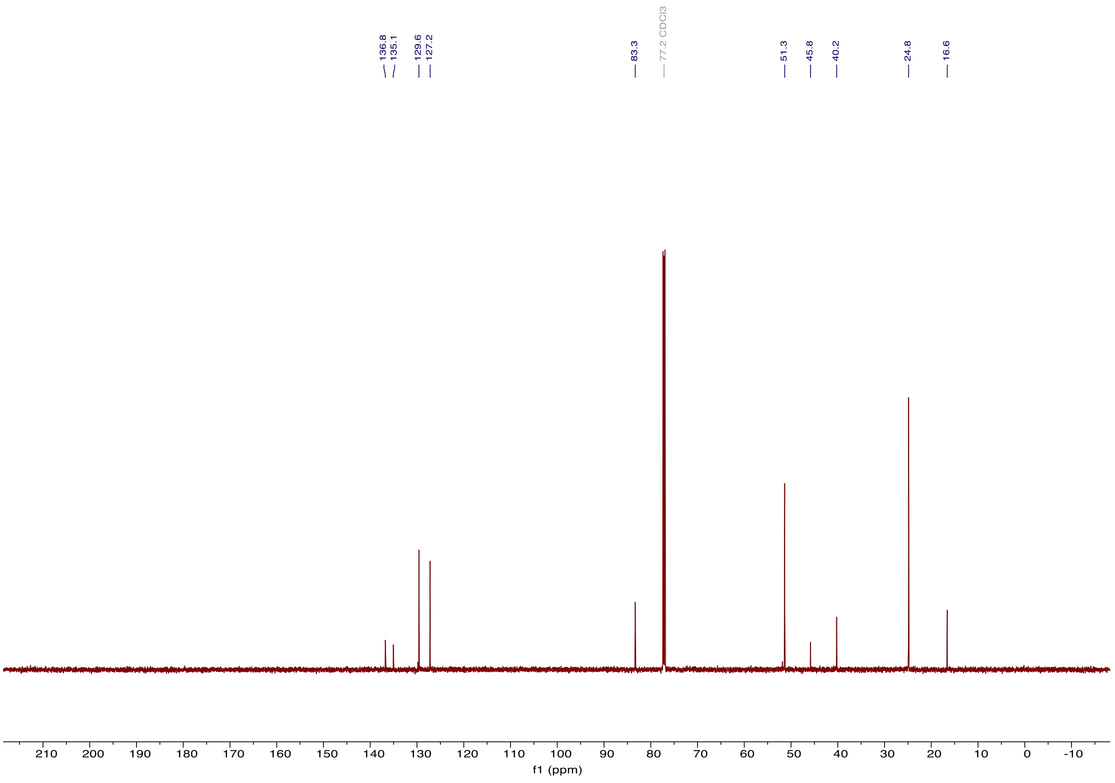

^{11}B NMR (192 MHz, $CDCl_3$)

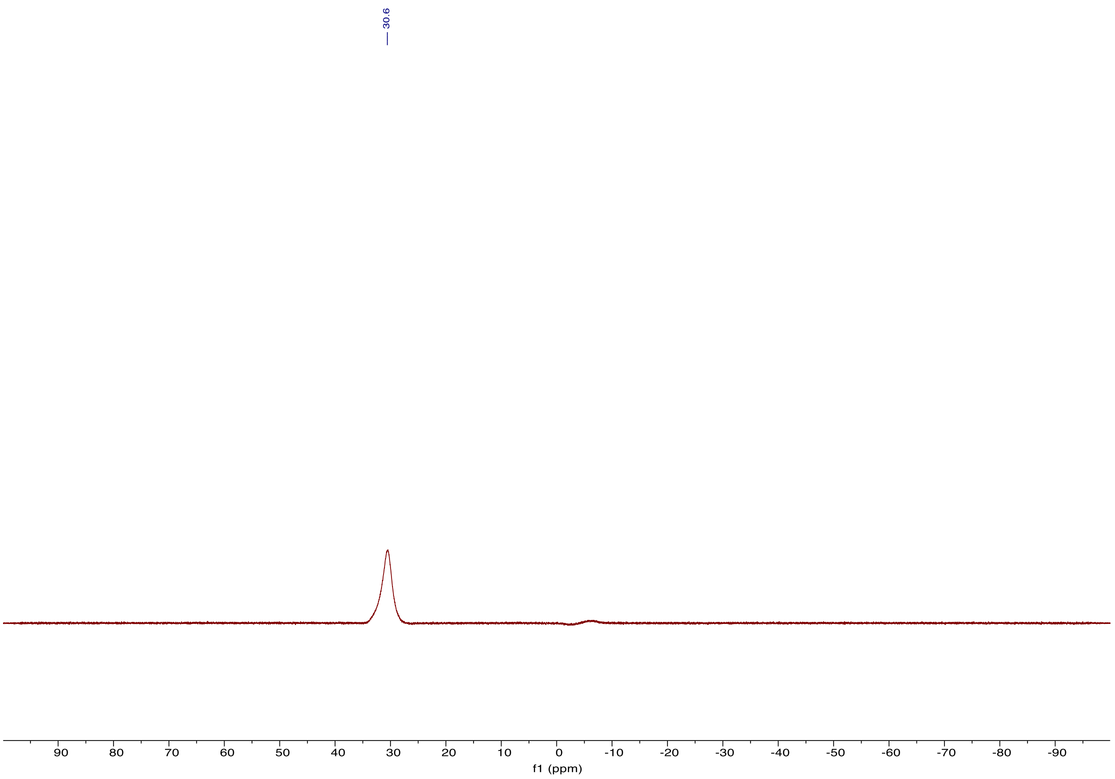

4,4,5,5-tetramethyl-2-(3-(1-phenylethyl)bicyclo[1.1.1]pentan-1-yl)-1,3,2-dioxaborolane (3x)

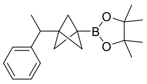

^{1}H NMR (600 MHz, $CDCl_3$)

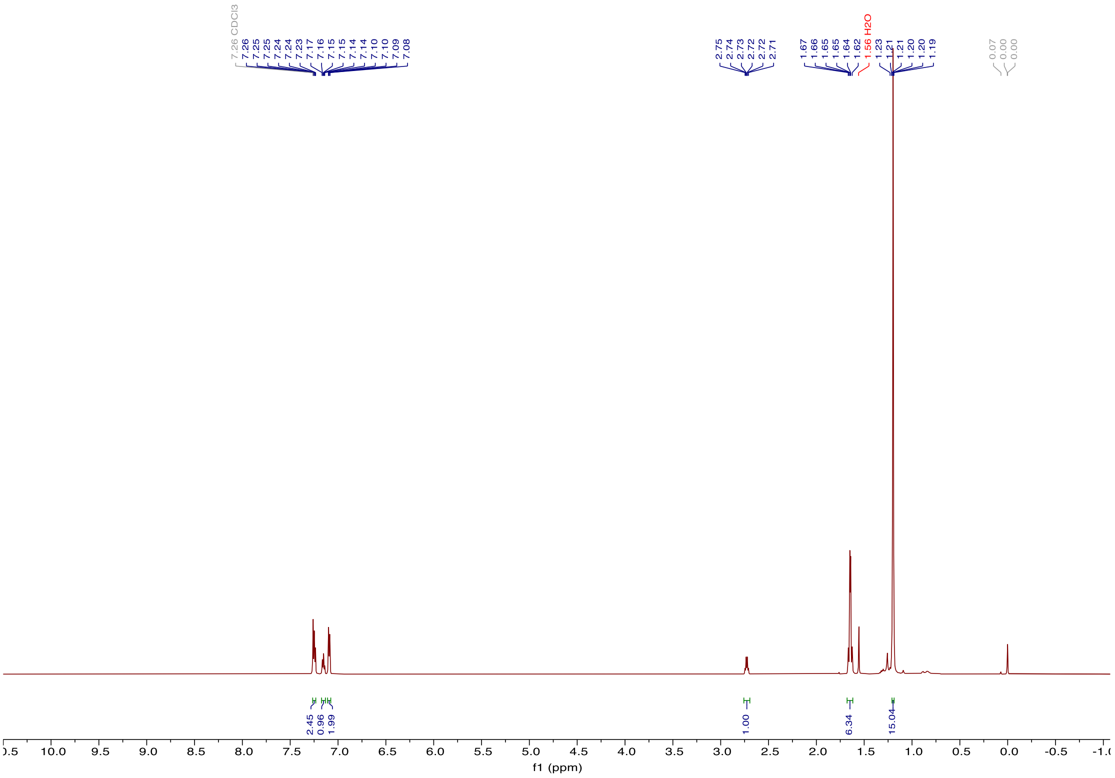

^{13}C NMR (151 MHz, $CDCl_3$)

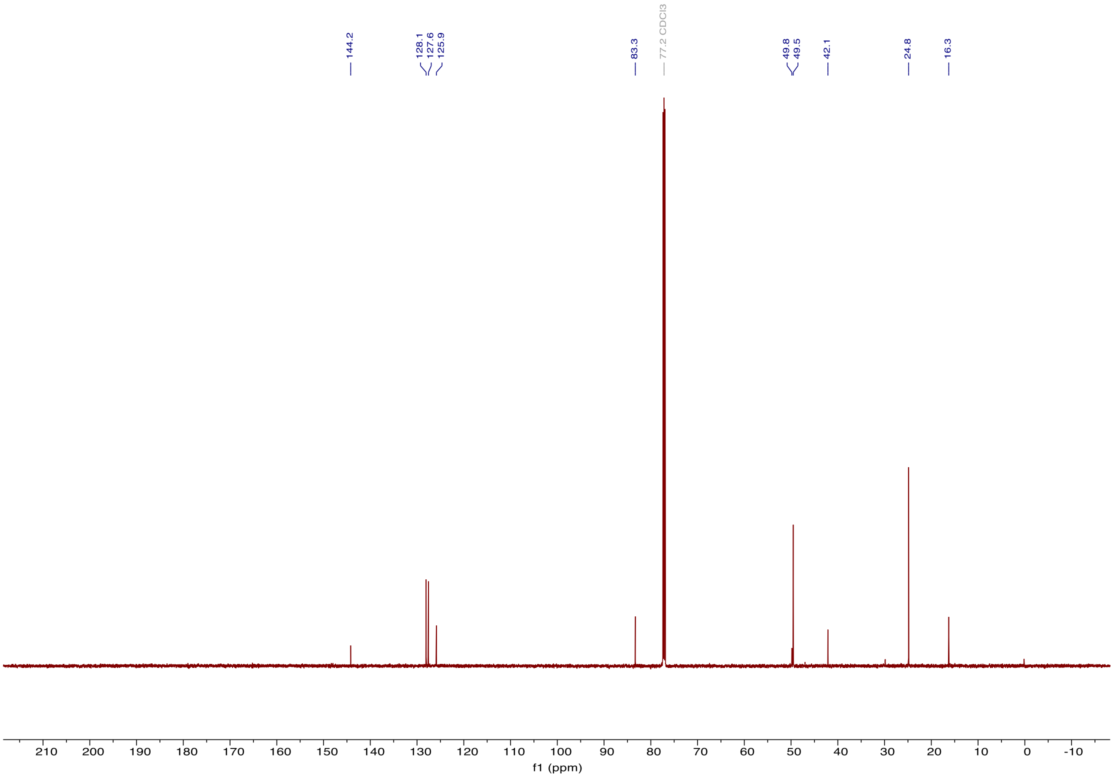

^{11}B NMR (192 MHz, $CDCl_3$)

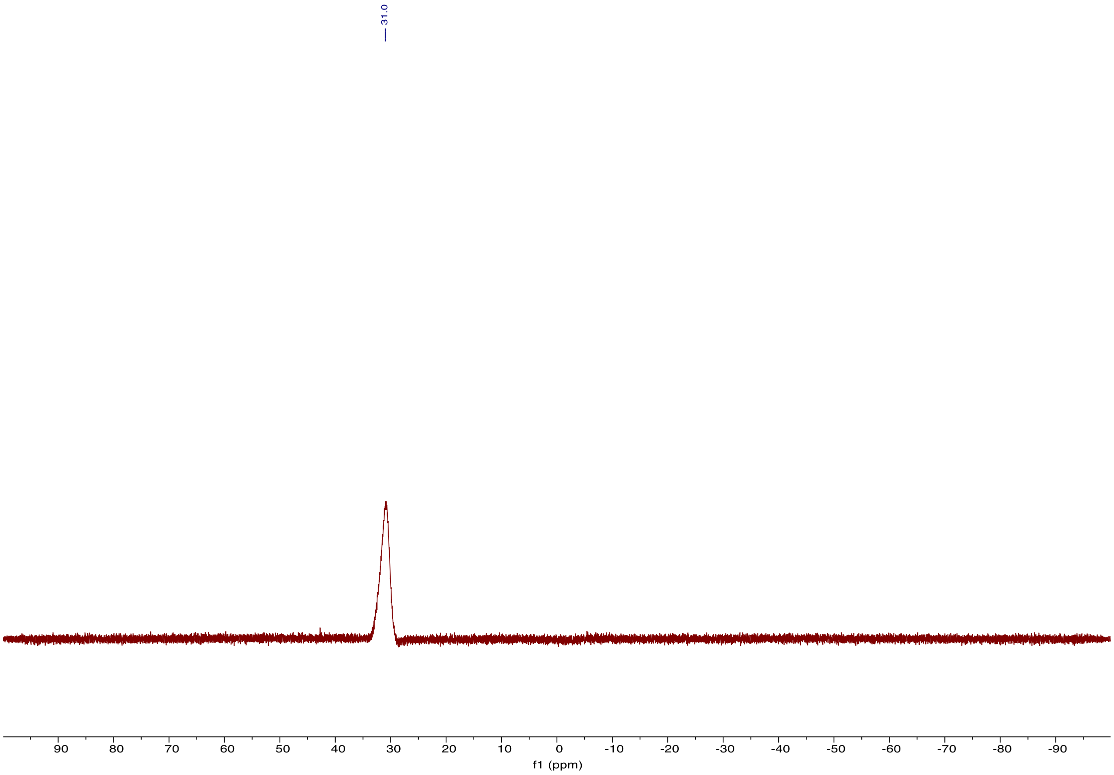

2-(3-(1-(4-fluorophenyl)ethyl)bicyclo[1.1.1]pentan-1-yl)-4,4,5,5-tetramethyl-1,3,2-dioxaborolane (3y)

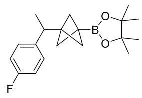

^{1}H NMR (600 MHz, $CDCl_3$)

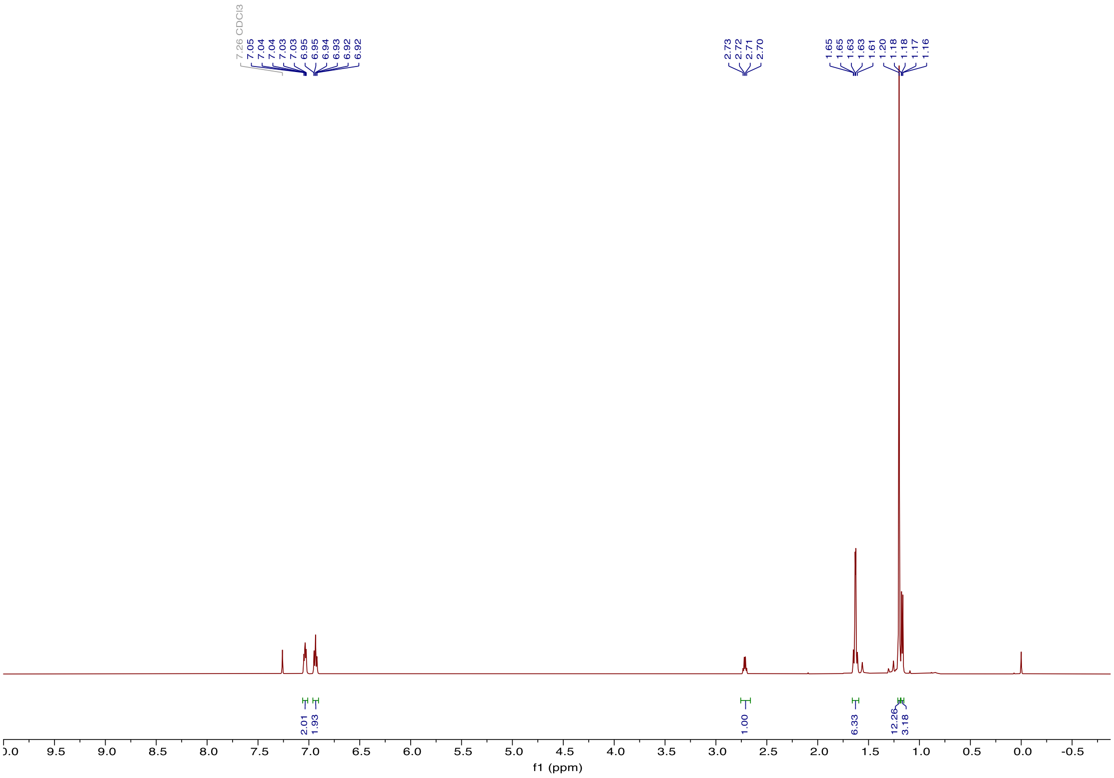

^{13}C NMR (151 MHz, $CDCl_3$)

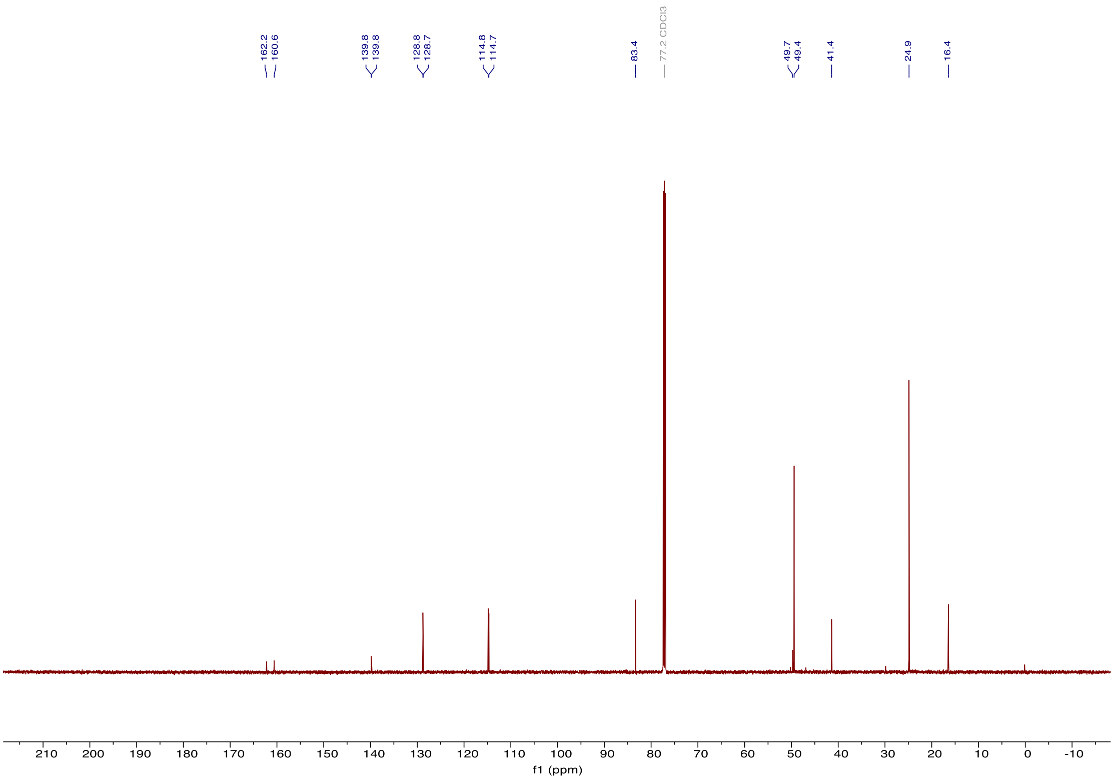

^{11}B NMR (192 MHz, $CDCl_3$)

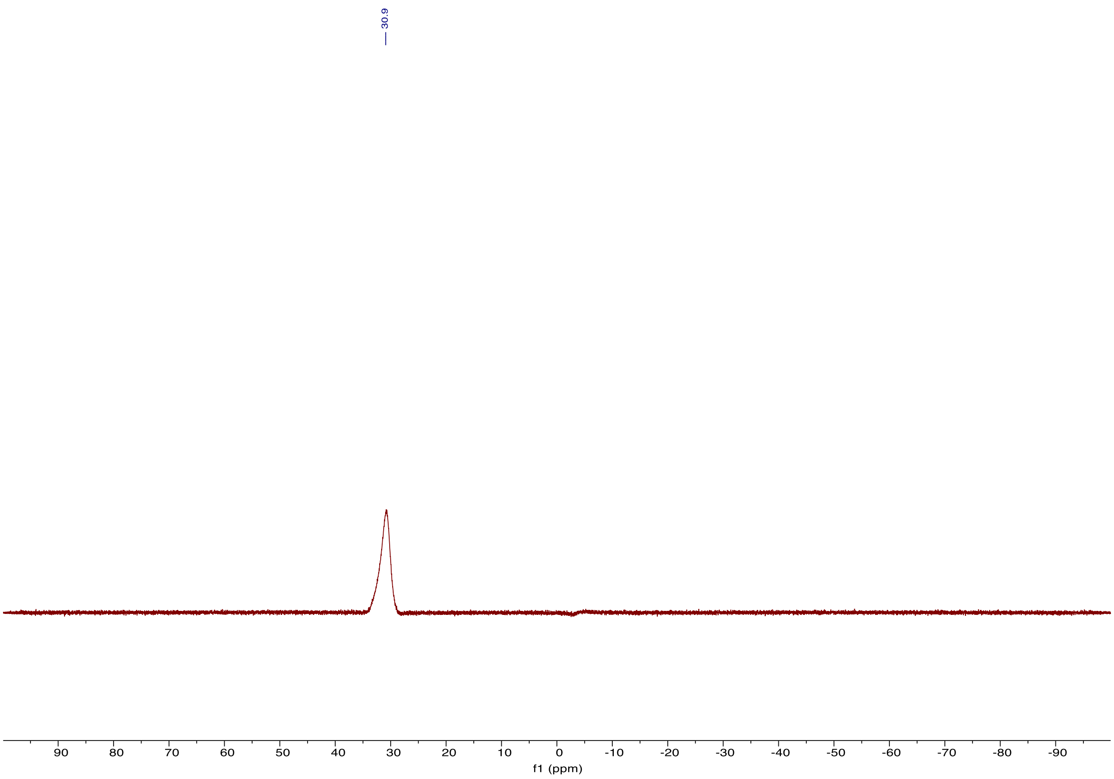

^{19}F NMR (564 MHz, $CDCl_3$)

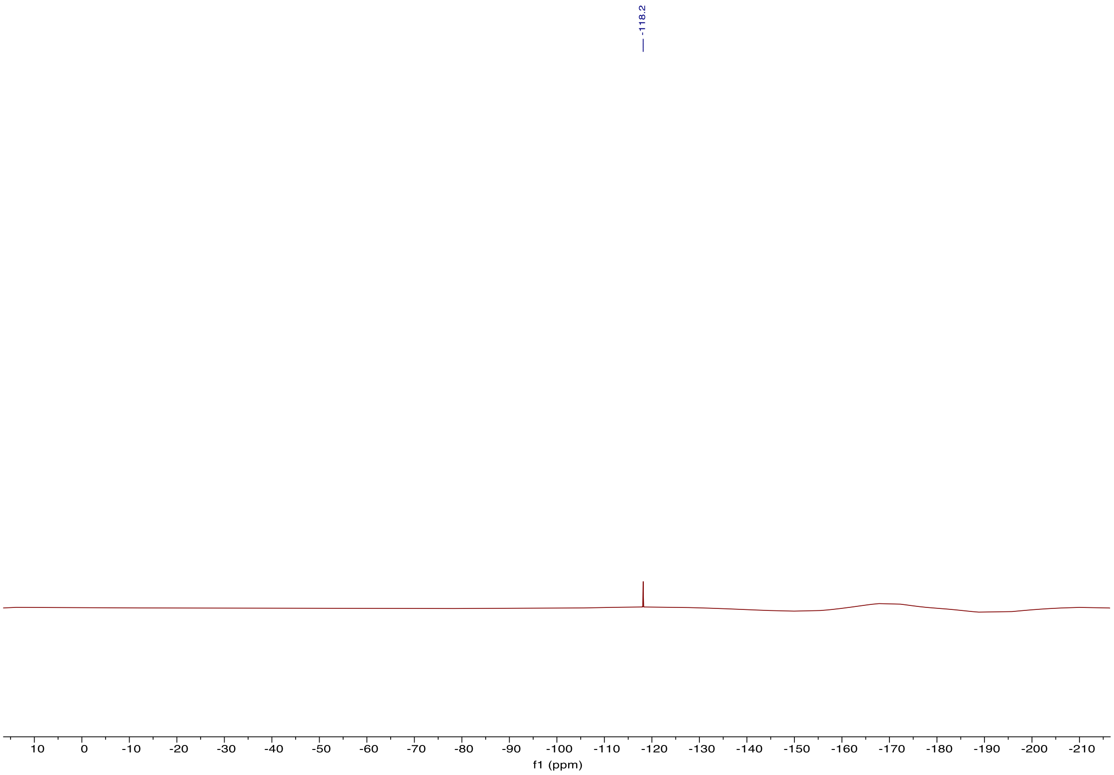

2-(3-(1-(3-bromophenyl)ethyl)bicyclo[1.1.1]pentan-1-yl)-4,4,5,5-tetramethyl-1,3,2-dioxaborolane (3z)

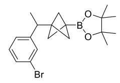

^{1}H NMR (600 MHz, $CDCl_3$)

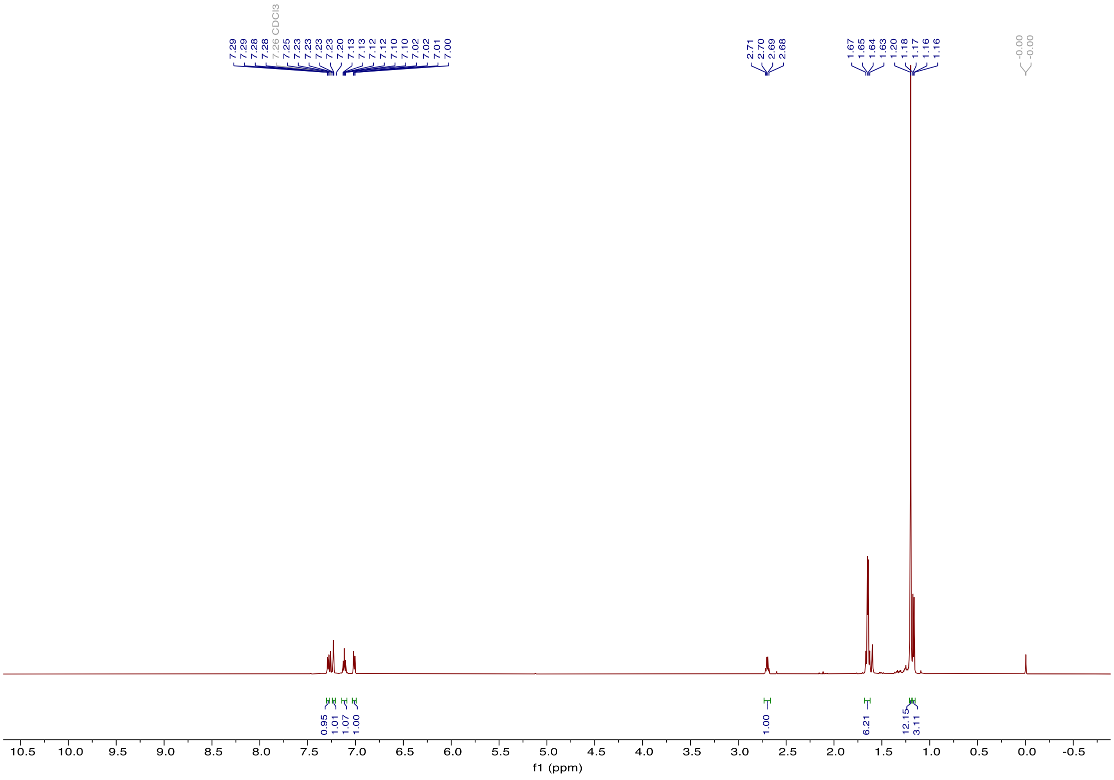

^{13}C NMR (151 MHz, $CDCl_3$)

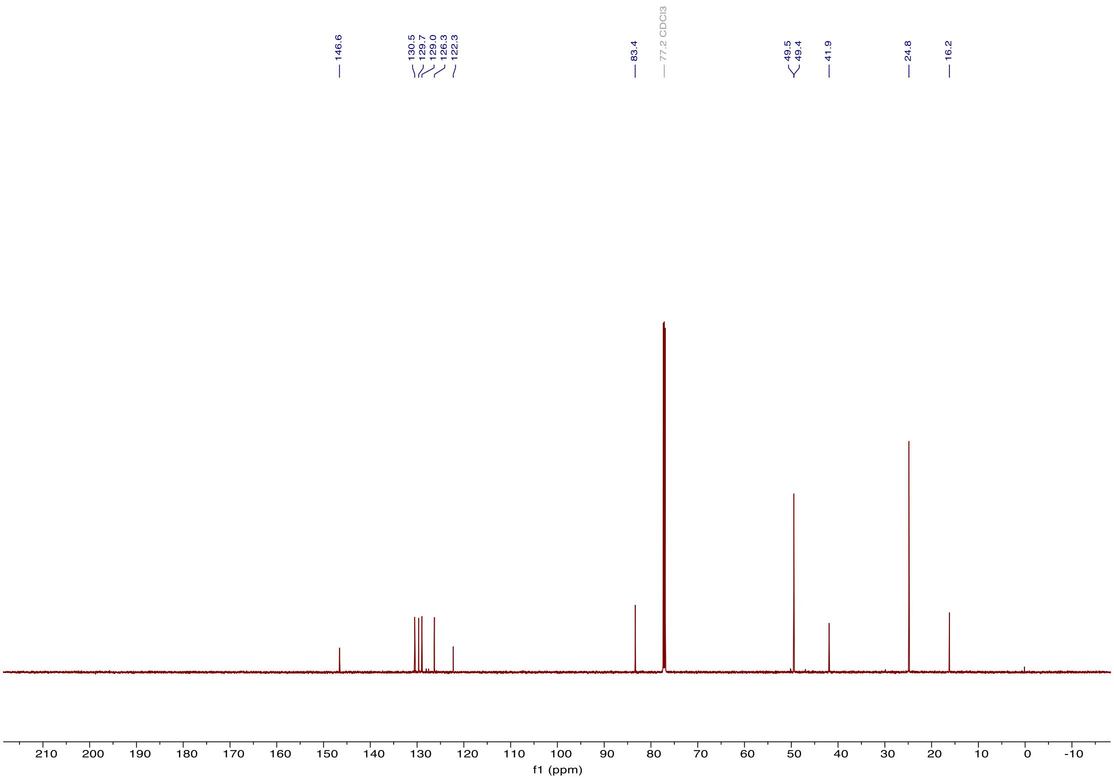

^{11}B NMR (192 MHz, $CDCl_3$)

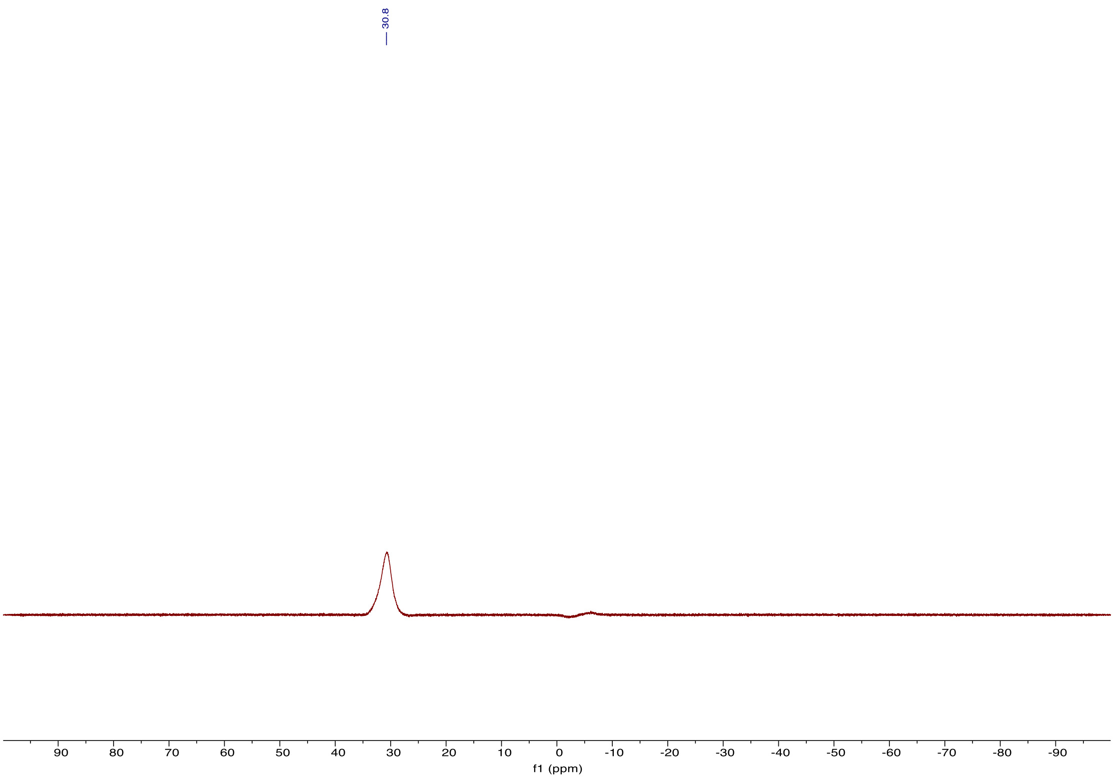

2-(3-(1-(4-chlorophenyl)-2-methylpropyl)bicyclo[1.1.1]pentan-1-yl)-4,4,5,5-tetramethyl-1,3,2-dioxaborolane (3aa)

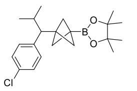

^{1}H NMR (600 MHz, $CDCl_3$)

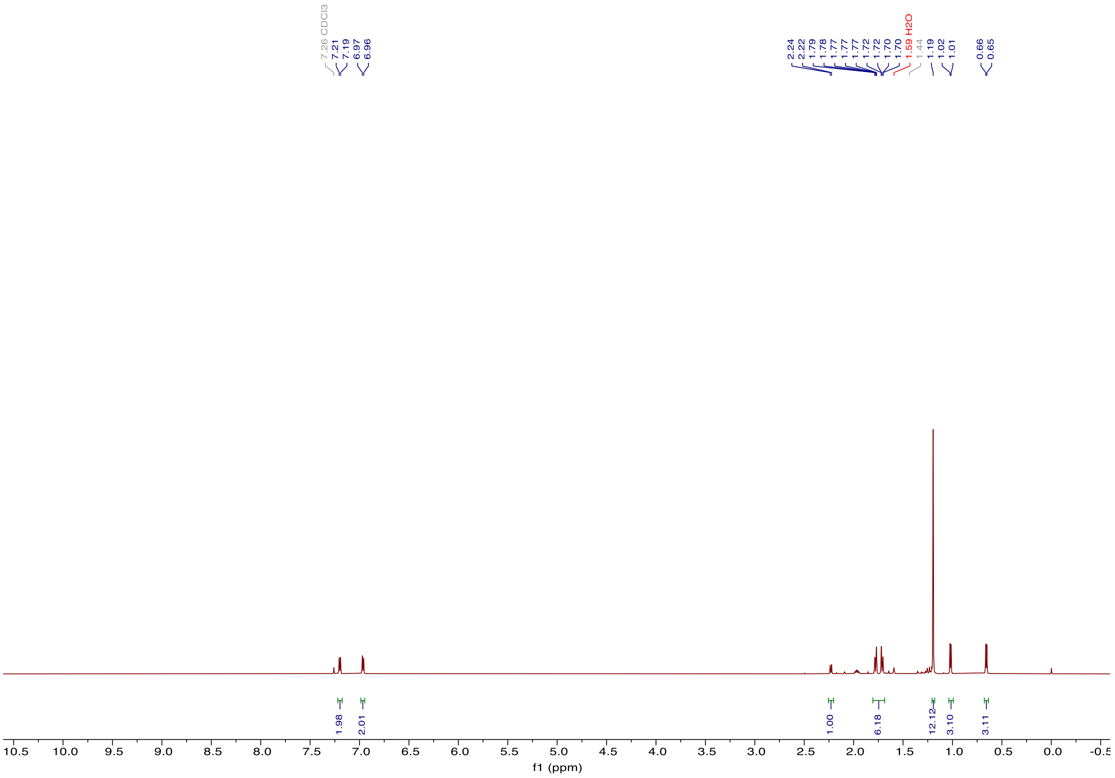

^{13}C NMR (151 MHz, $CDCl_3$)

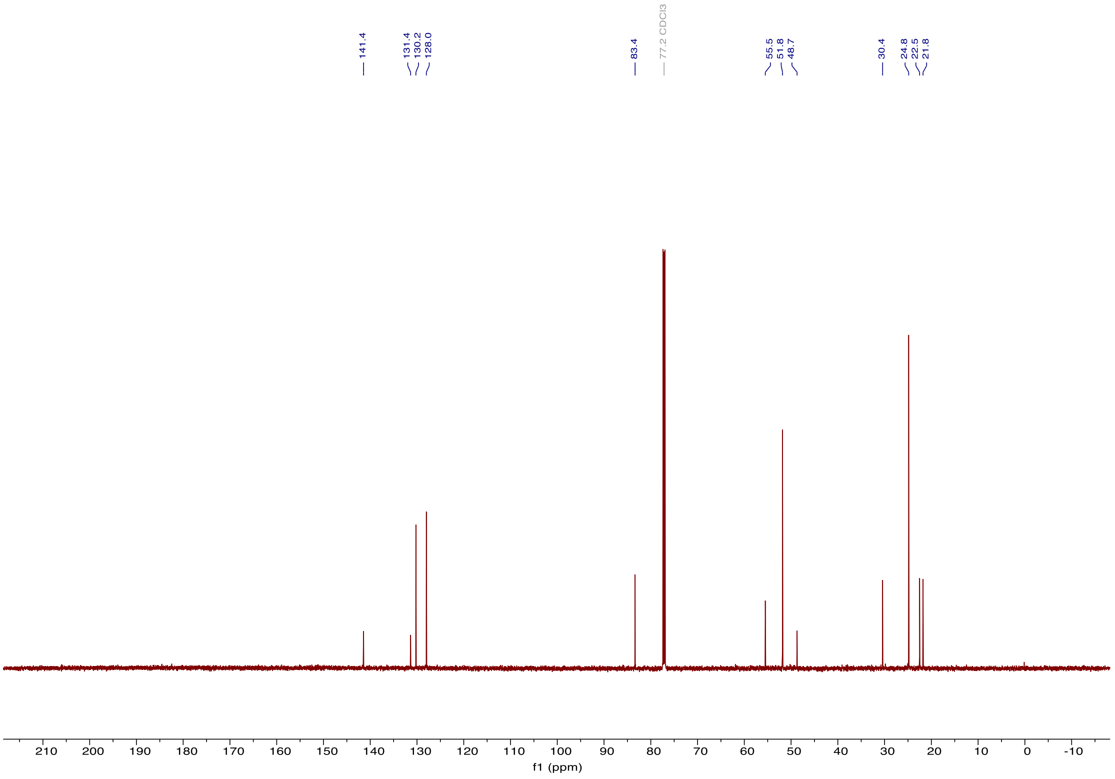

^{11}B NMR (192 MHz, $CDCl_3$)

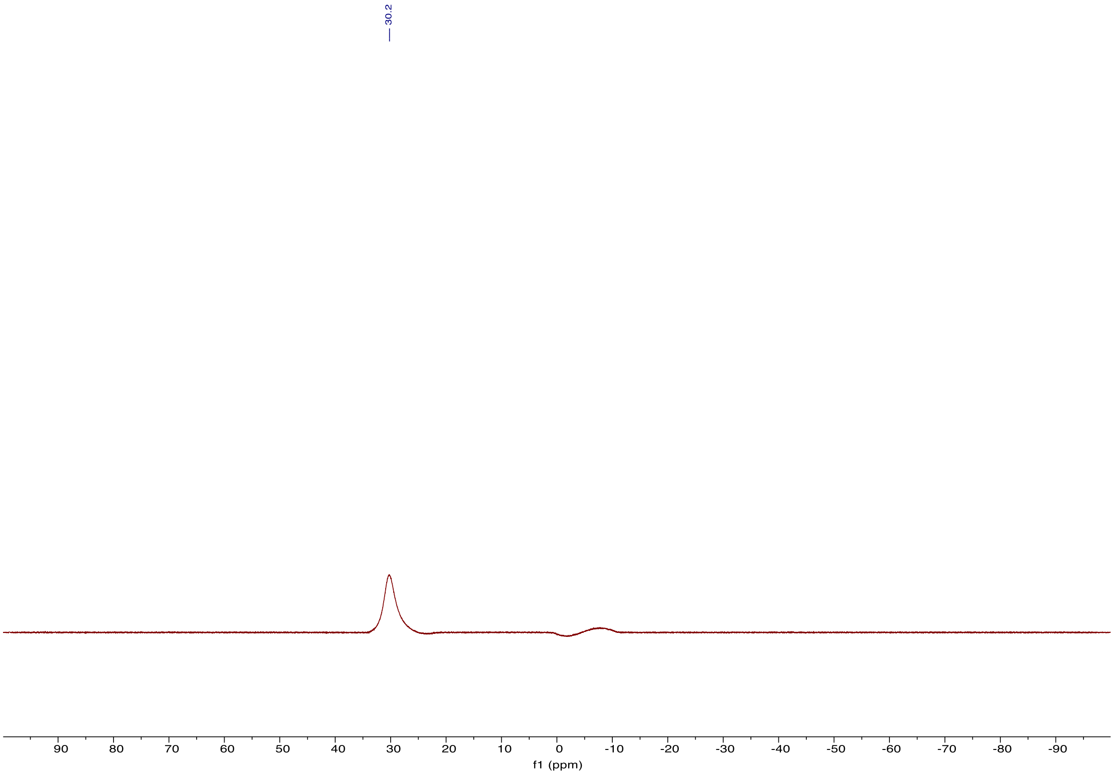

4,4,5,5-tetramethyl-2-(3-(1-(4-phenoxyphenyl)ethyl)bicyclo[1.1.1]pentan-1-yl)-1,3,2-dioxaborolane (3ab)

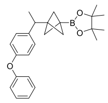

^{1}H NMR (600 MHz, $CDCl_3$)

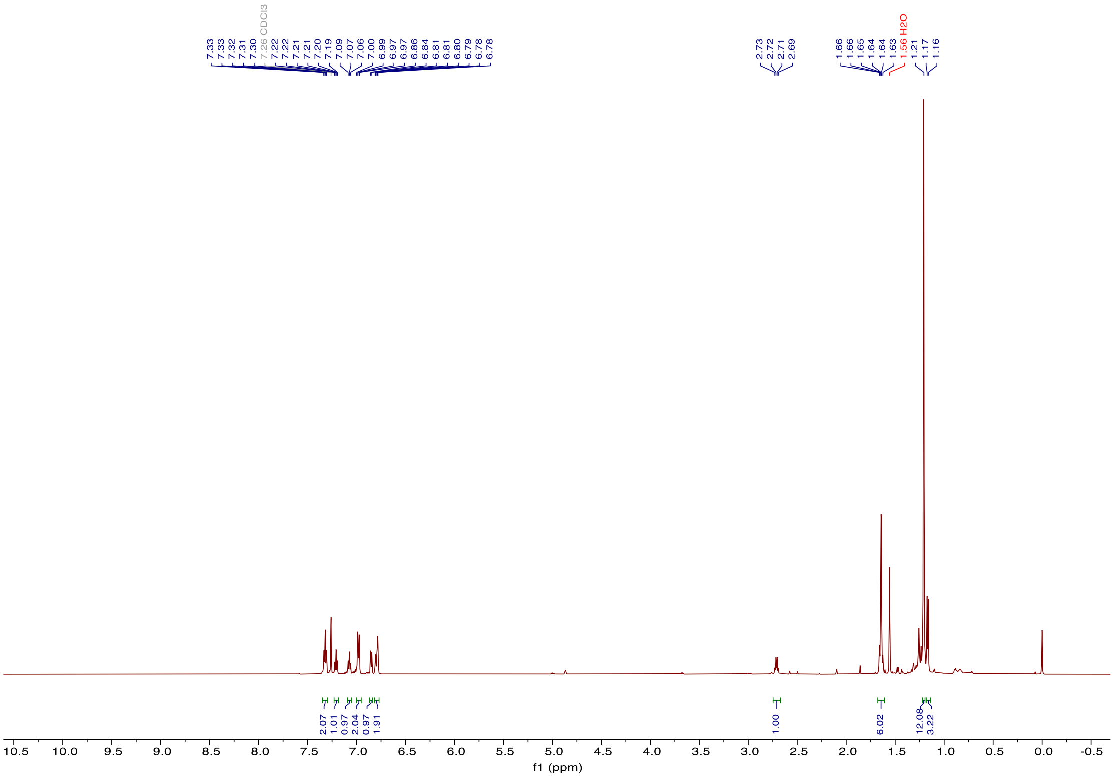

^{13}C NMR (151 MHz, CDCl$_3$)

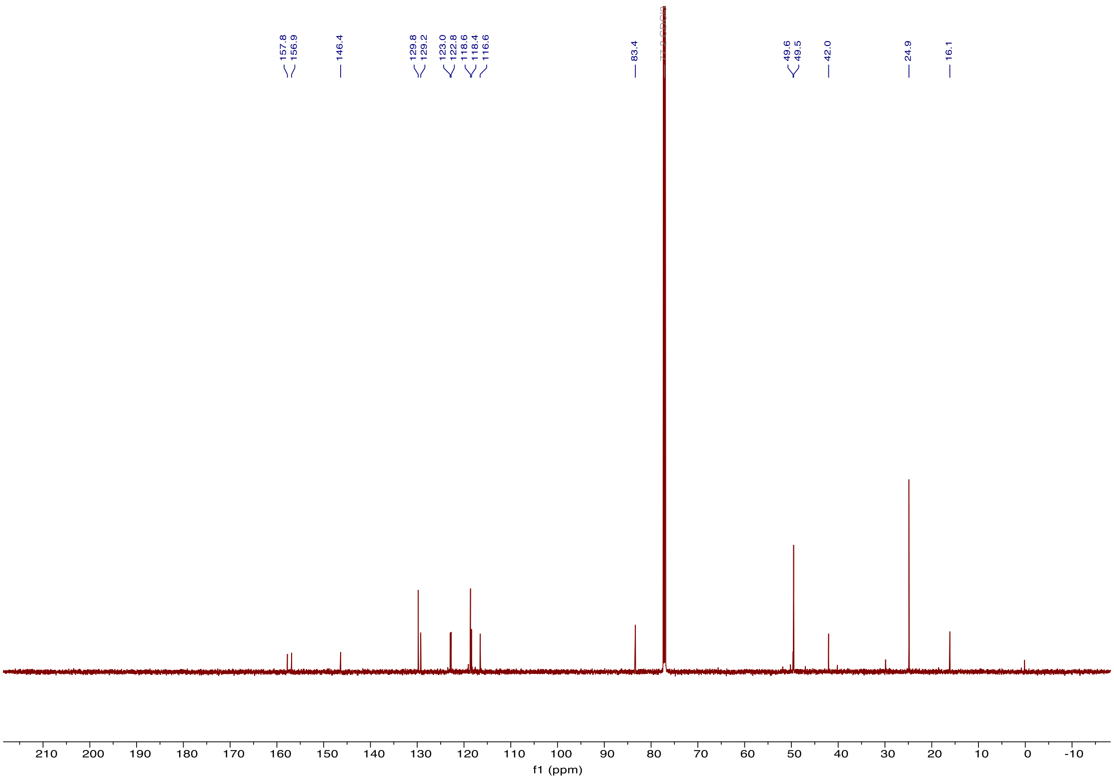

^{11}B NMR (192 MHz, CDCl$_3$)

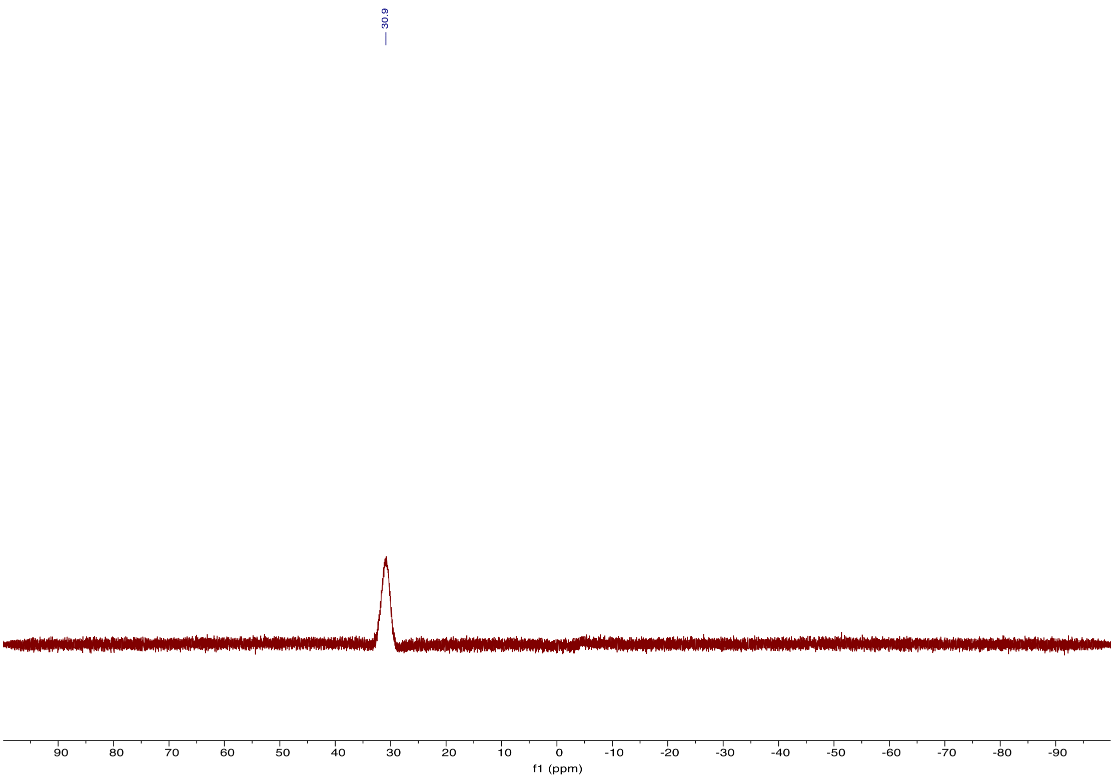

2-(3-(1-(4-isobutylphenyl)ethyl)bicyclo[1.1.1]pentan-1-yl)-4,4,5,5-tetramethyl-1,3,2-dioxaborolane (3ac)

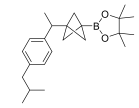

^{1}H NMR (600 MHz, $CDCl_3$)

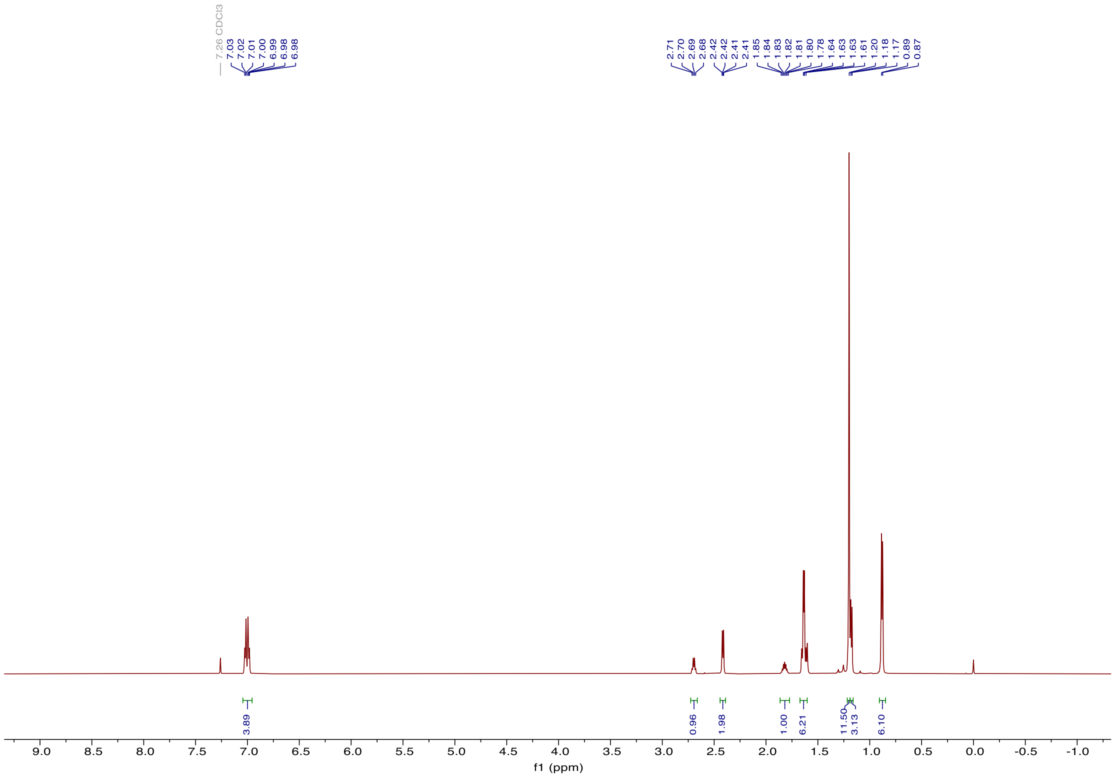

^{13}C NMR (151 MHz, $CDCl_3$)

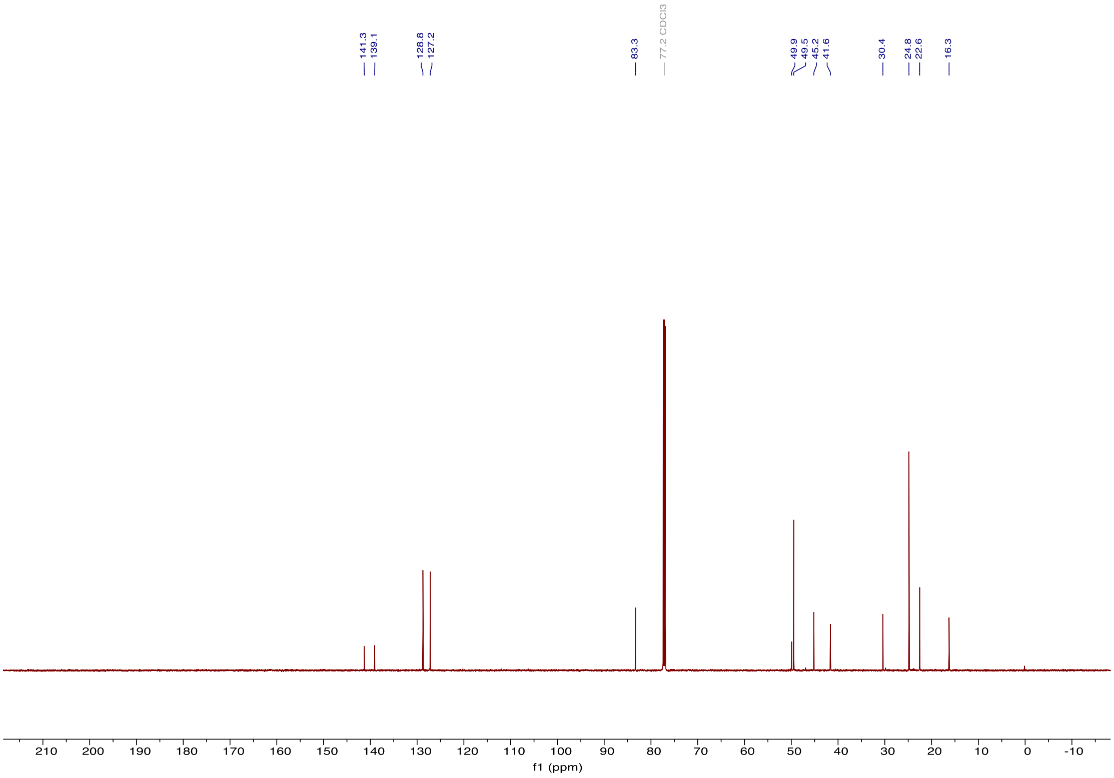

^{11}B NMR (192 MHz, $CDCl_3$)

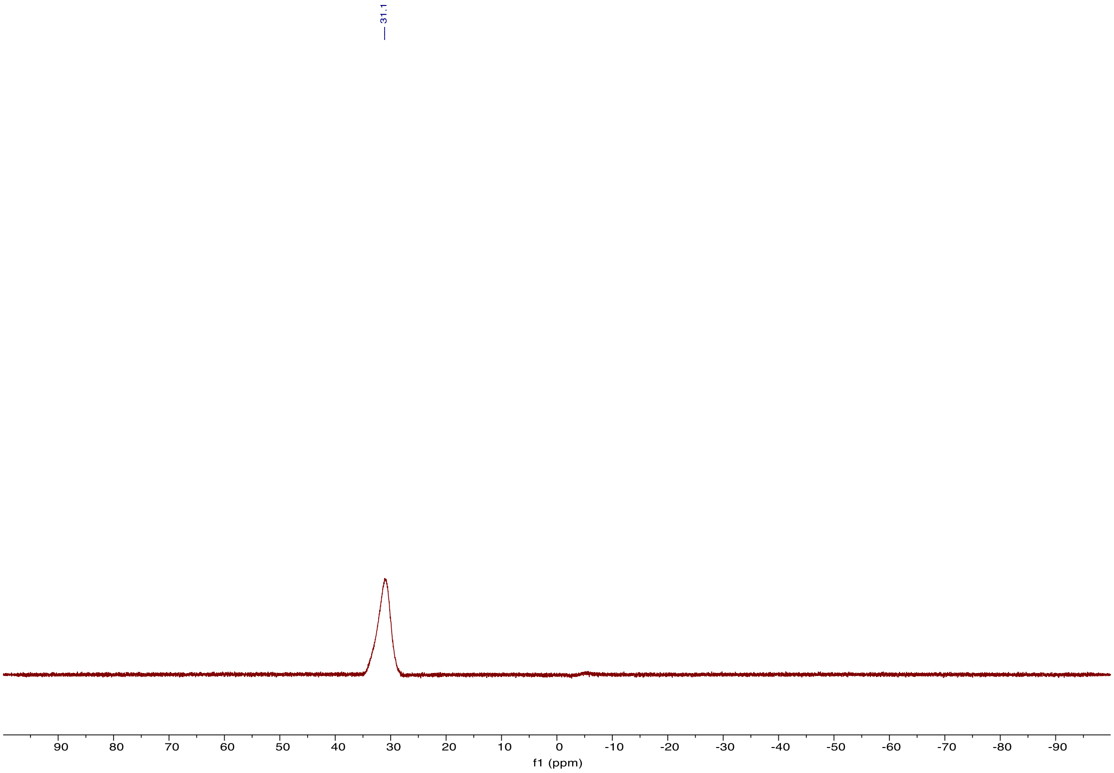

2-(3-(bicyclo[4.2.0]octa-1(6),2,4-trien-7-yl)bicyclo[1.1.1]pentan-1-yl)-4,4,5,5-tetramethyl-1,3,2-dioxaborolane (3ad)

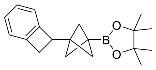

^{1}H NMR (600 MHz, $CDCl_3$)

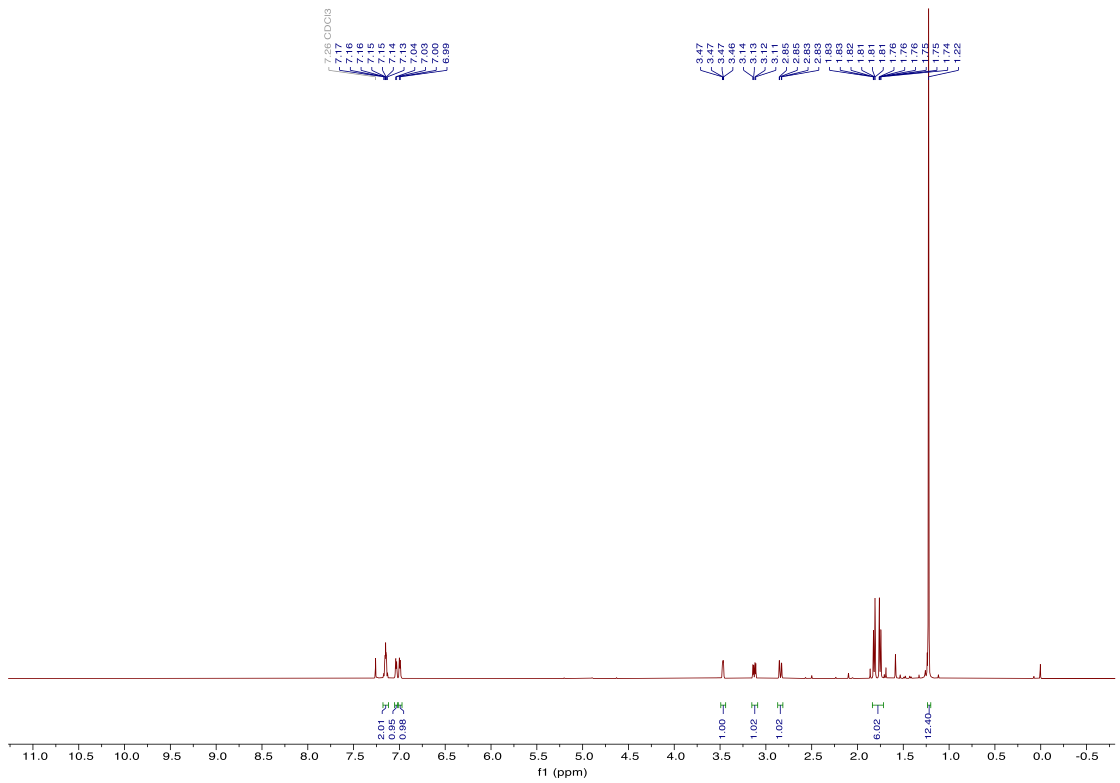

^{13}C NMR (151 MHz, $CDCl_3$)

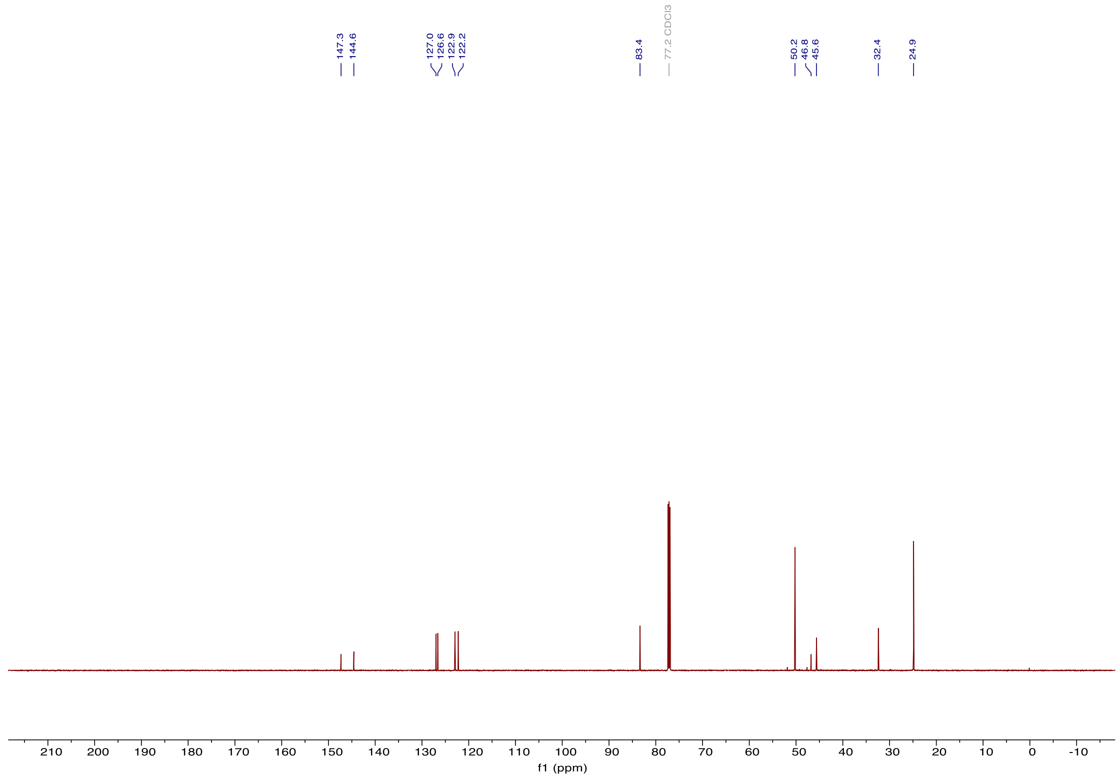

^{11}B NMR (192 MHz, $CDCl_3$)

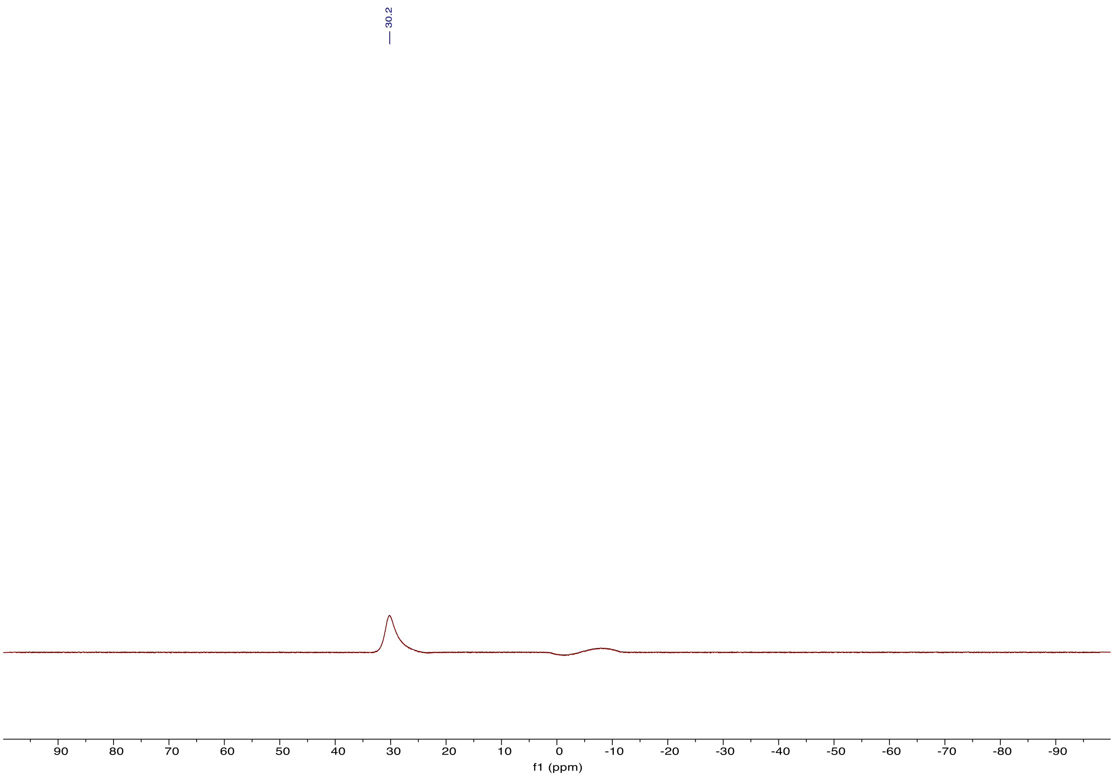

4,4,5,5-tetramethyl-2-(3-(1,2,3,4-tetrahydronaphthalen-1-yl)bicyclo[1.1.1]pentan-1-yl)-1,3,2-dioxaborolane (3ae)

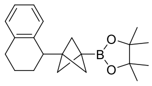

^{1}H NMR (600 MHz, $CDCl_3$)

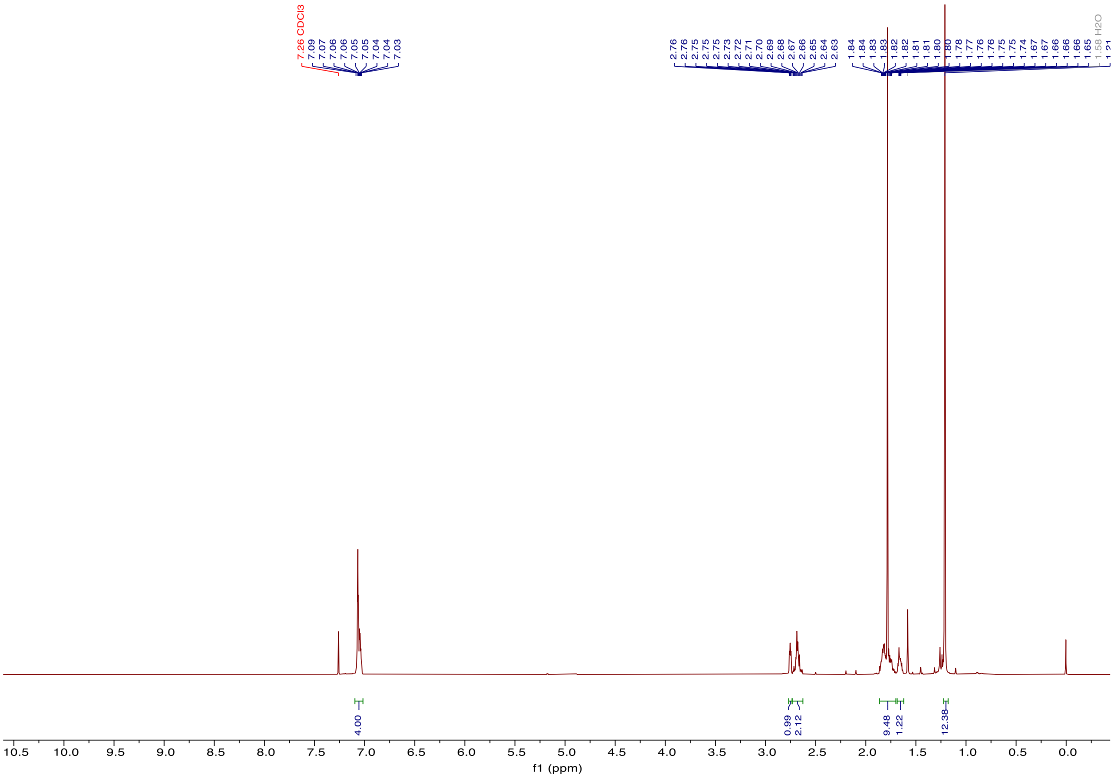

^{13}C NMR (151 MHz, $CDCl_3$)

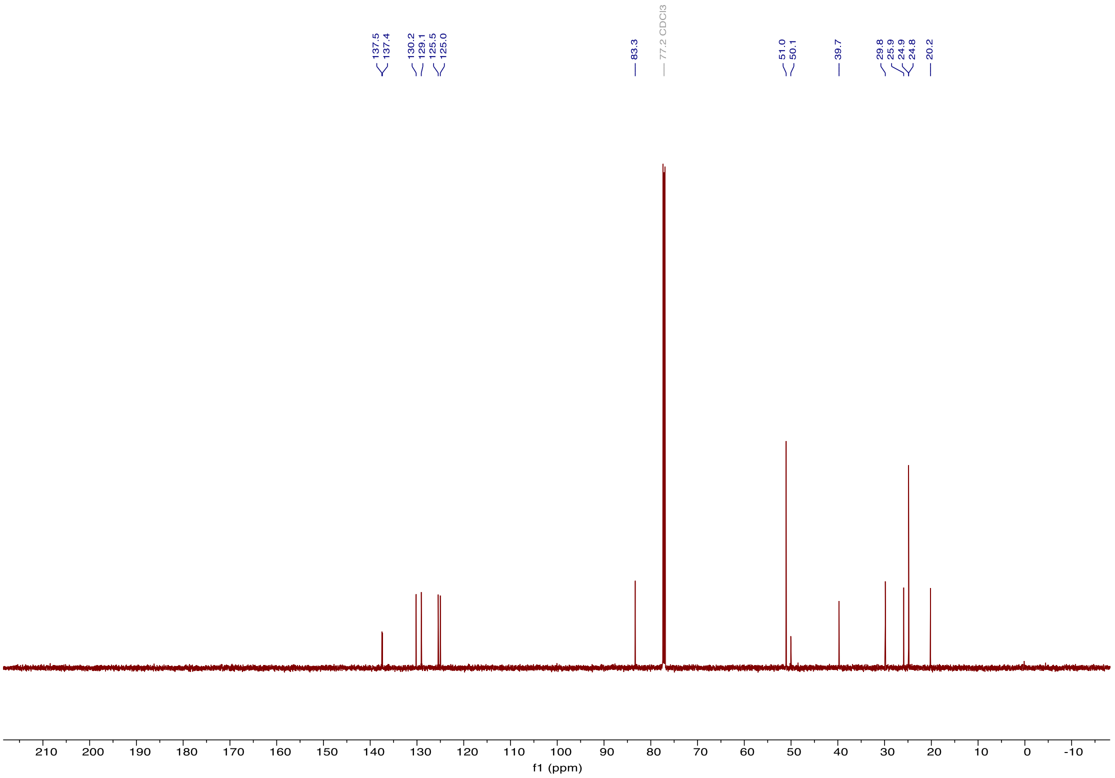

^{11}B NMR (192 MHz, $CDCl_3$)

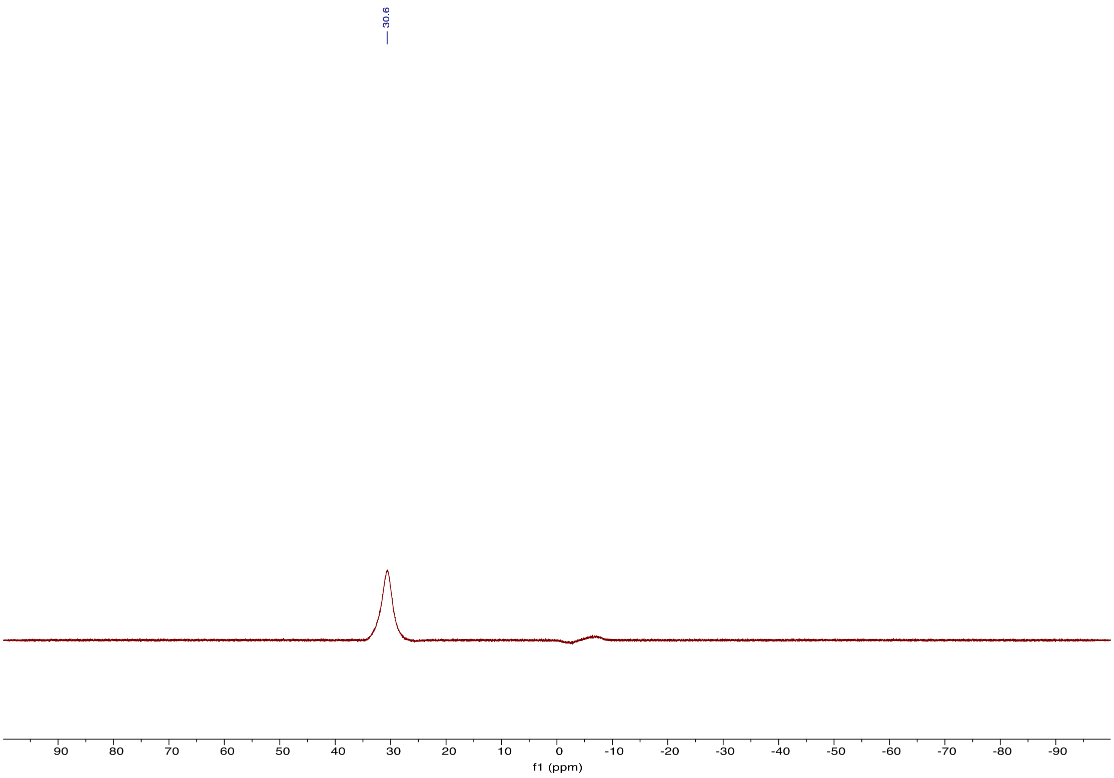

2-(3-(2-(4-ethyl-3-iodophenyl)propan-2-yl)bicyclo[1.1.1]pentan-1-yl)-4,4,5,5-tetramethyl-1,3,2-dioxaborolane (3af)

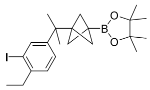

^{1}H NMR (600 MHz, $CDCl_3$)

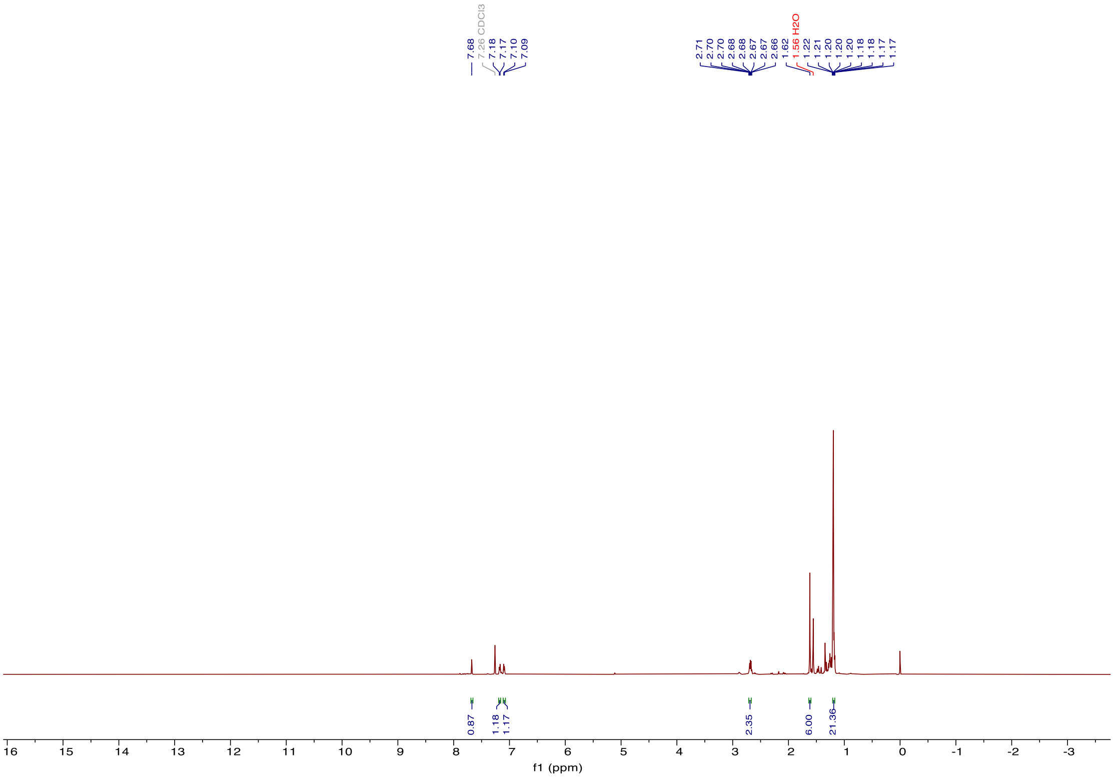

^{13}C NMR (151 MHz, $CDCl_3$)

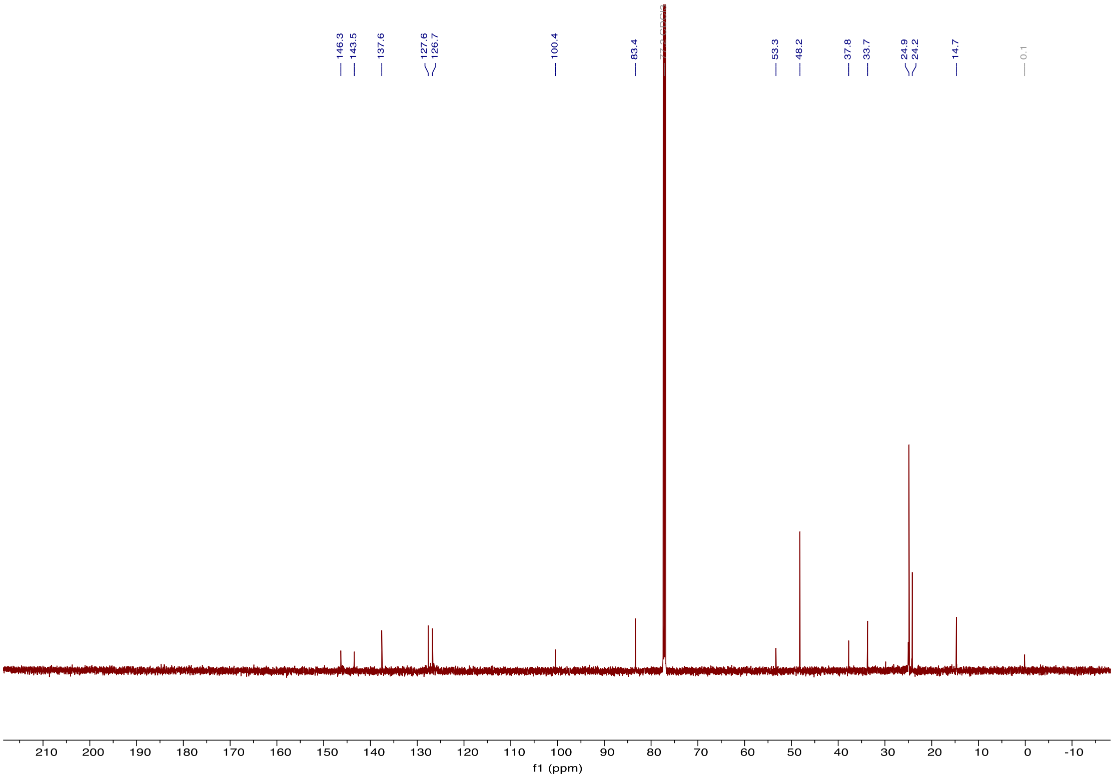

^{11}B NMR (192 MHz, $CDCl_3$)

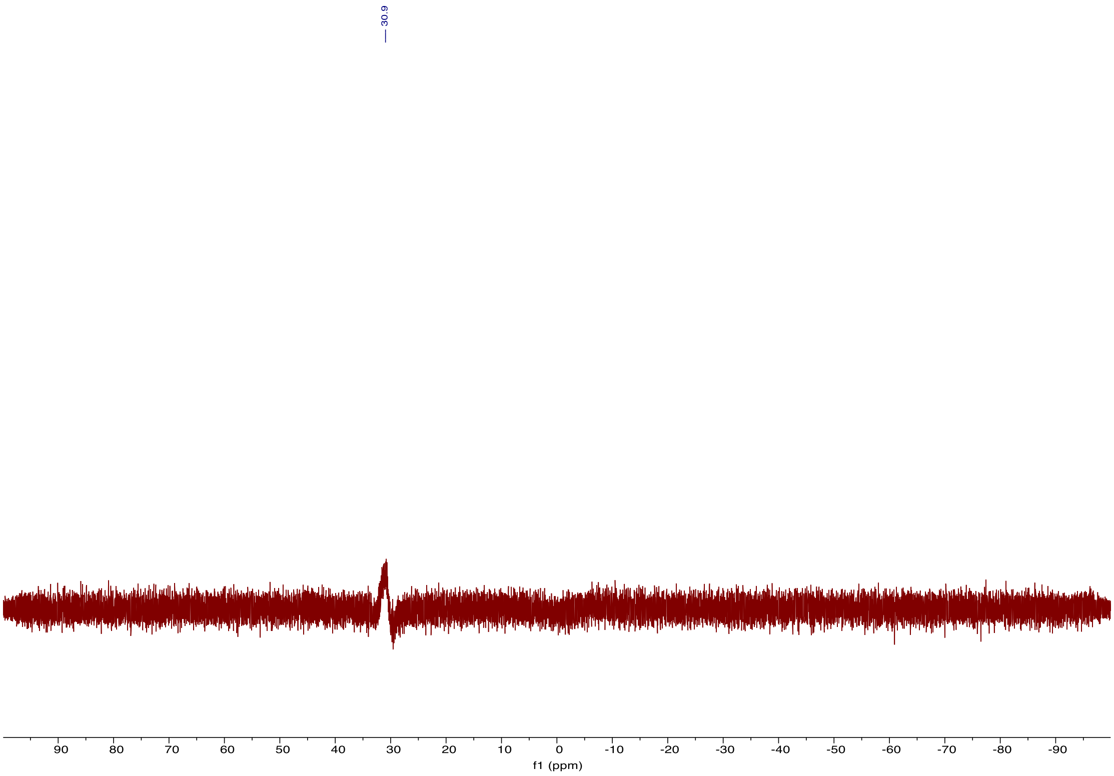

2-(3-(2-(4-bromophenyl)propan-2-yl)bicyclo[1.1.1]pentan-1-yl)-4,4,5,5-tetramethyl-1,3,2-dioxaborolane (3ag)

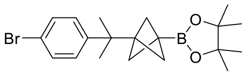

^{1}H NMR (600 MHz, $CDCl_3$)

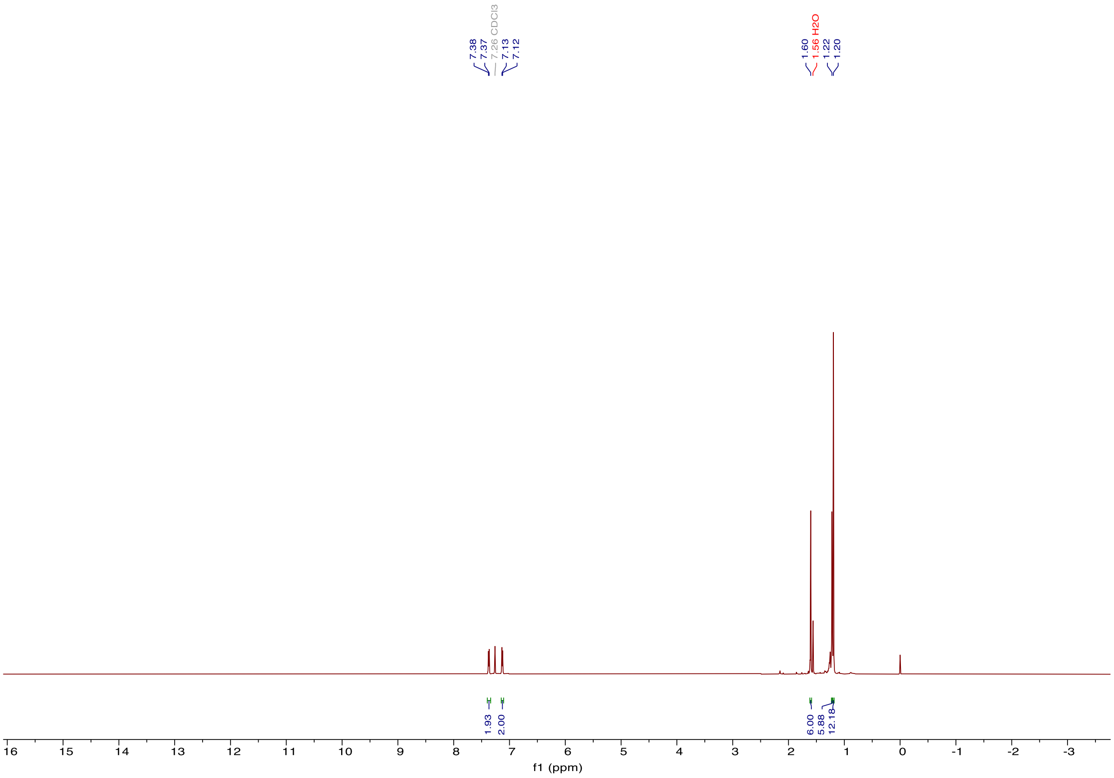

^{13}C NMR (151 MHz, $CDCl_3$)

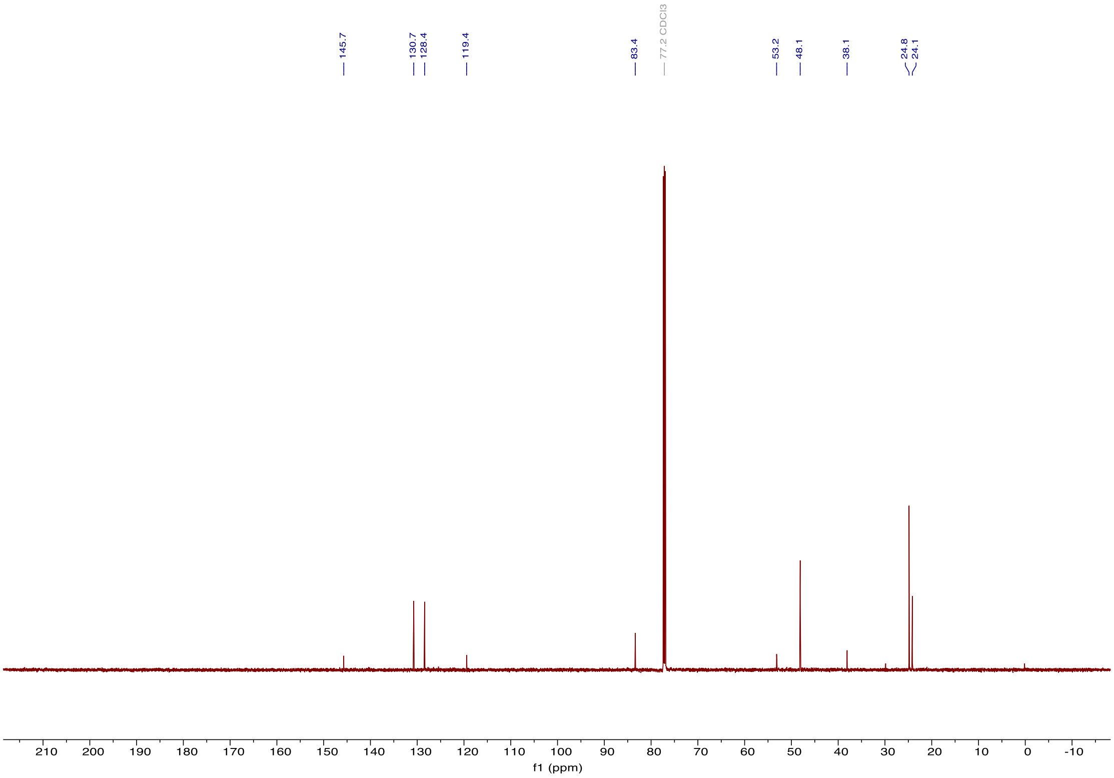

^{11}B NMR (192 MHz, $CDCl_3$)

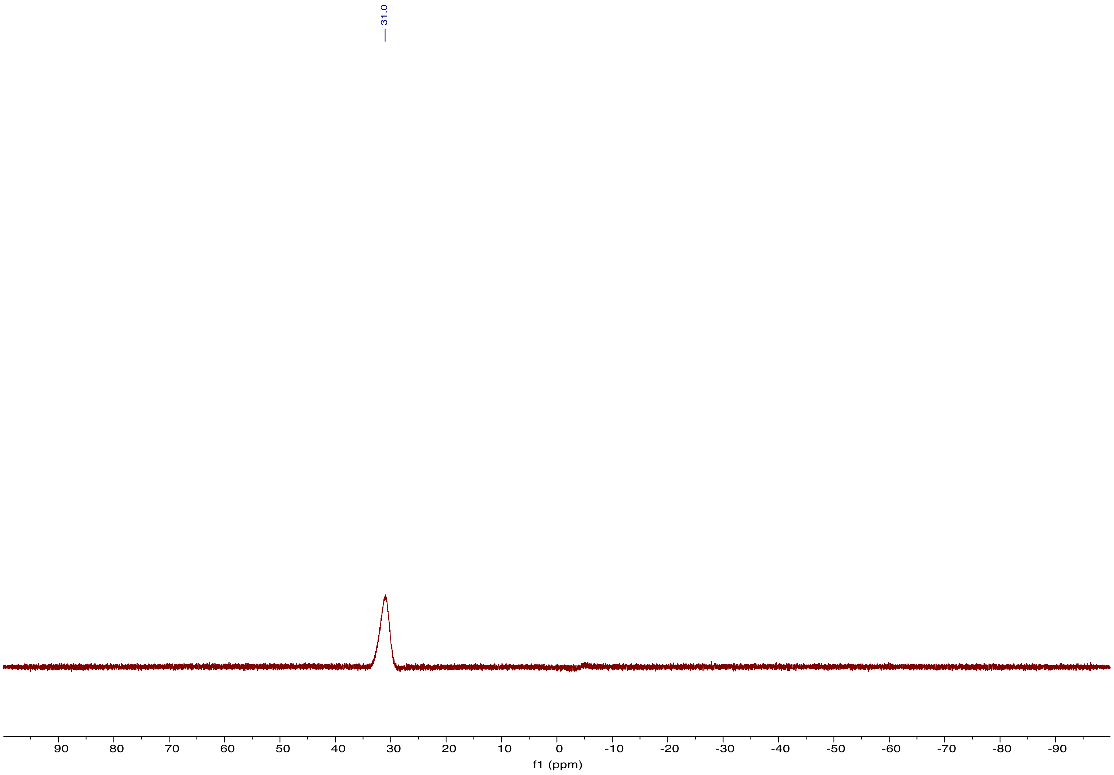

4,4,5,5-tetramethyl-2-(3-(1-phenylcyclopropyl)bicyclo[1.1.1]pentan-1-yl)-1,3,2-dioxaborolane (3ah)

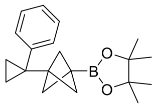

^{1}H NMR (600 MHz, $CDCl_3$)

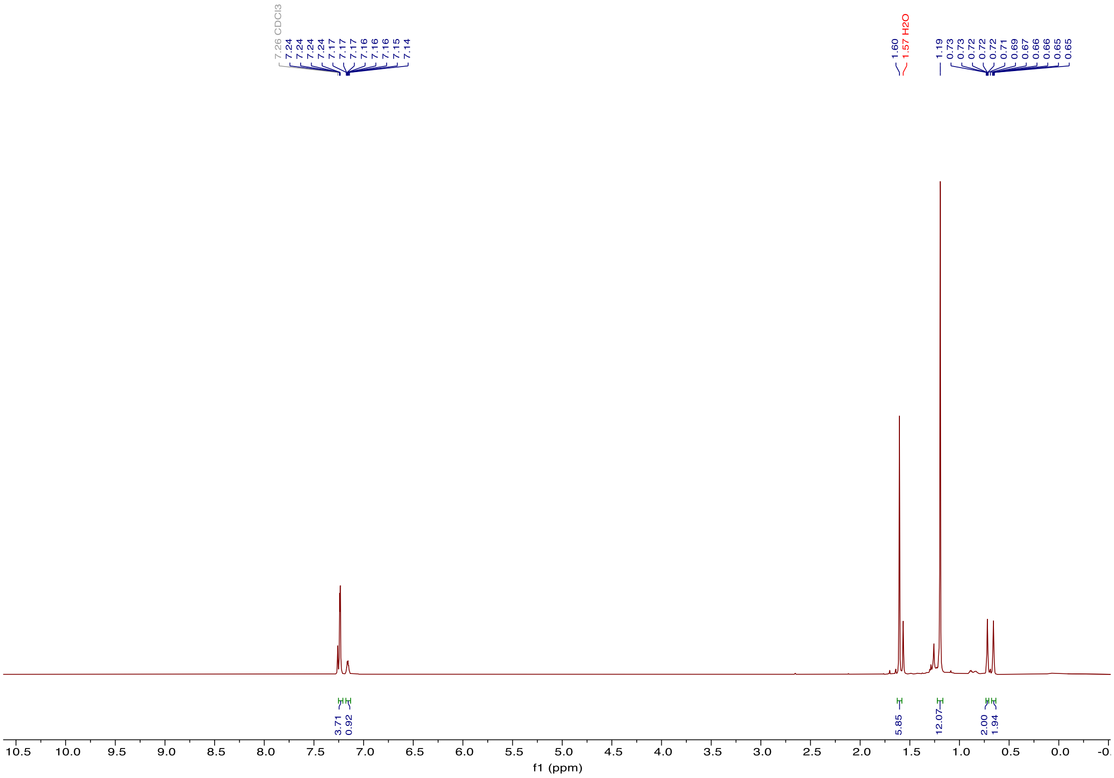

^{13}C NMR (151 MHz, $CDCl_3$)

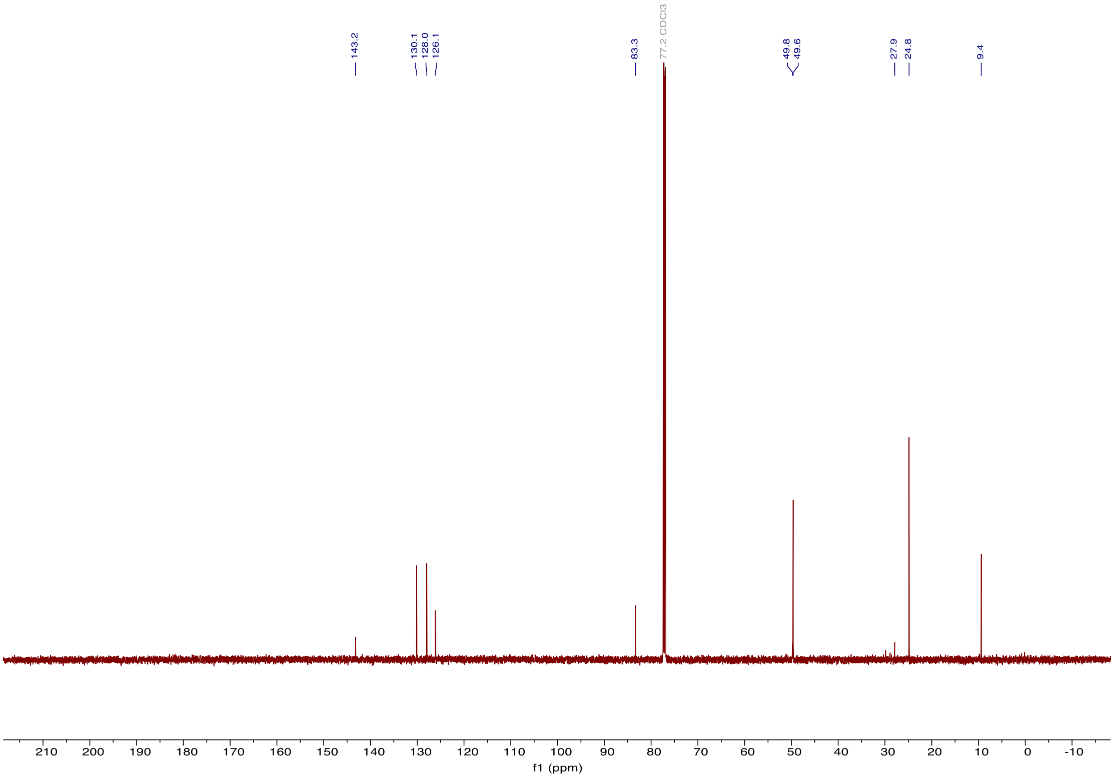

^{11}B NMR (192 MHz, $CDCl_3$)

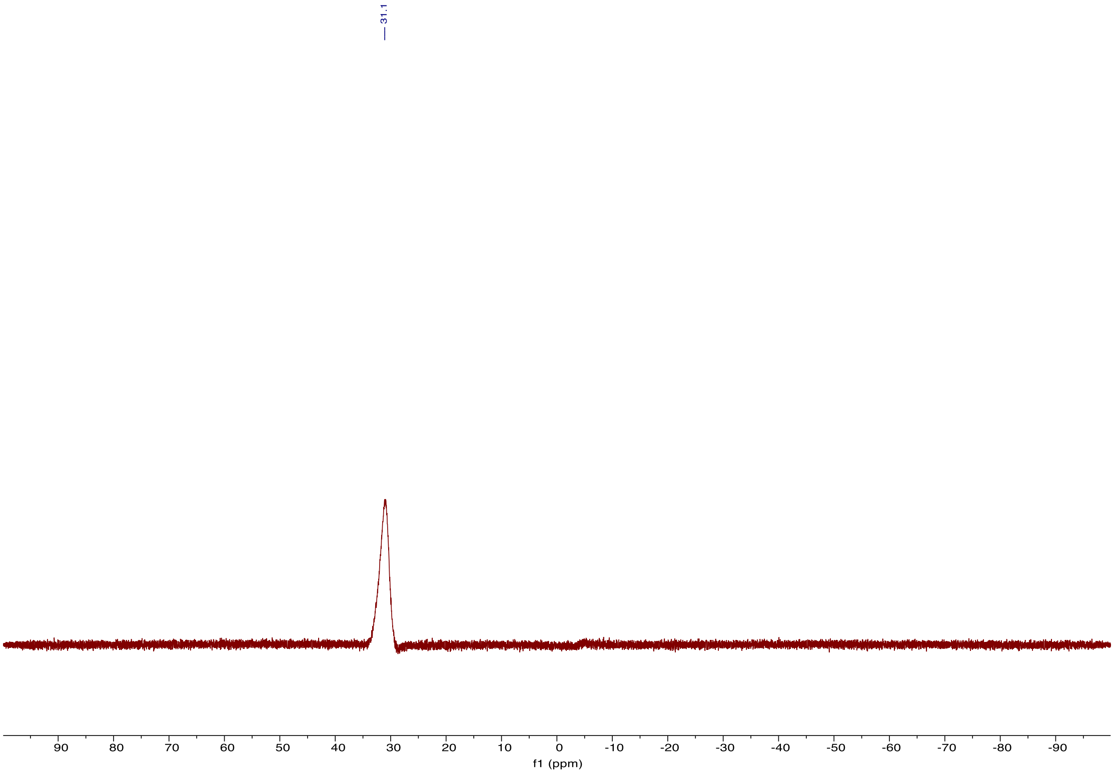

2-(3-(1-(4-bromophenyl)cyclopropyl)bicyclo[1.1.1]pentan-1-yl)-4,4,5,5-tetramethyl-1,3,2-dioxaborolane (3ai)

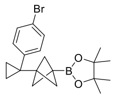

^{1}H NMR (600 MHz, CDCl$_3$)

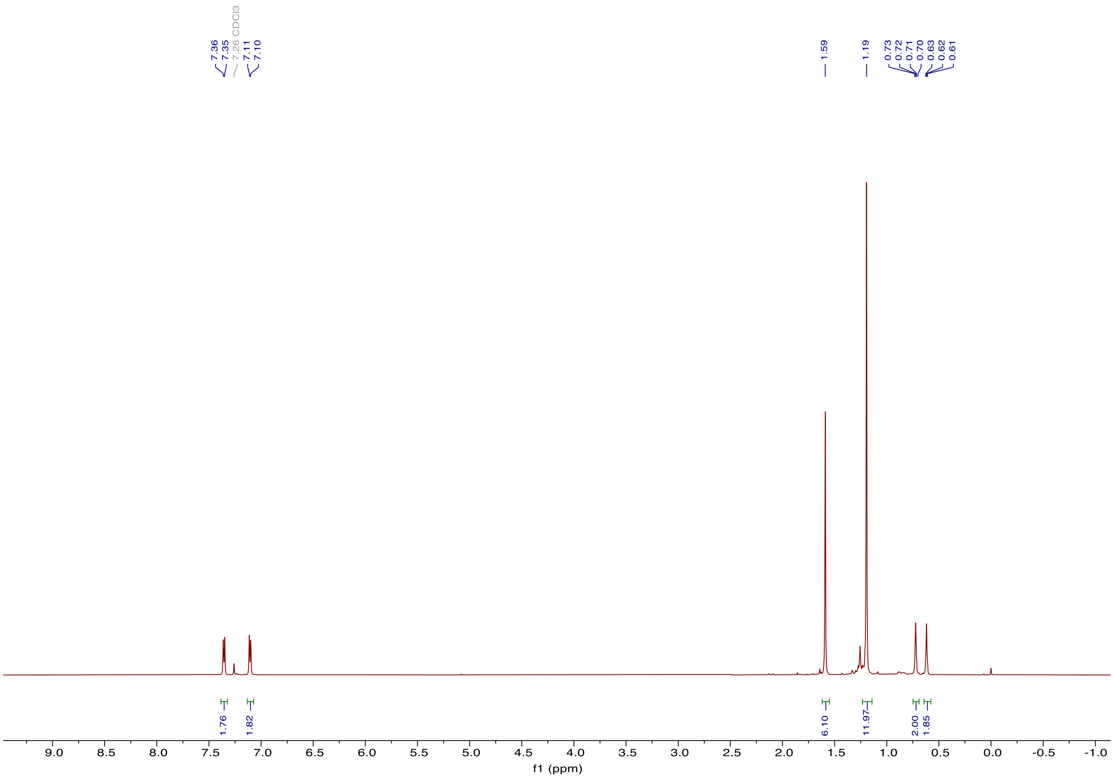

^{13}C NMR (151 MHz, CDCl$_3$)

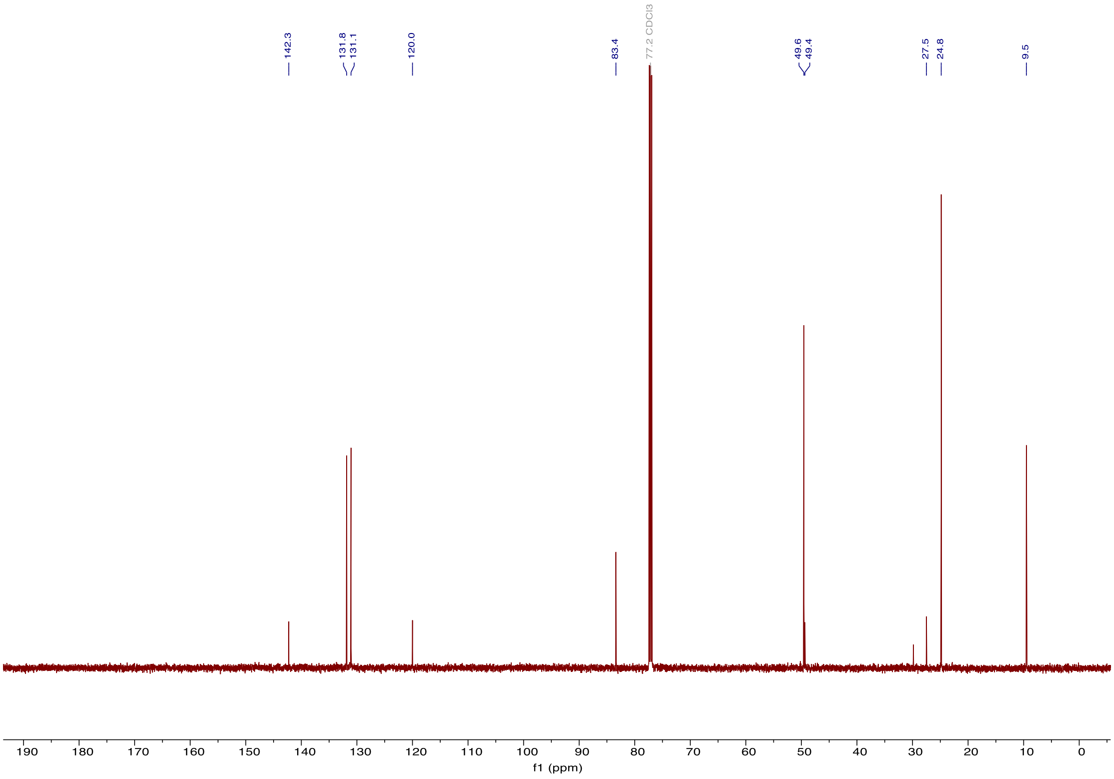

^{11}B NMR (192 MHz, $CDCl_3$)

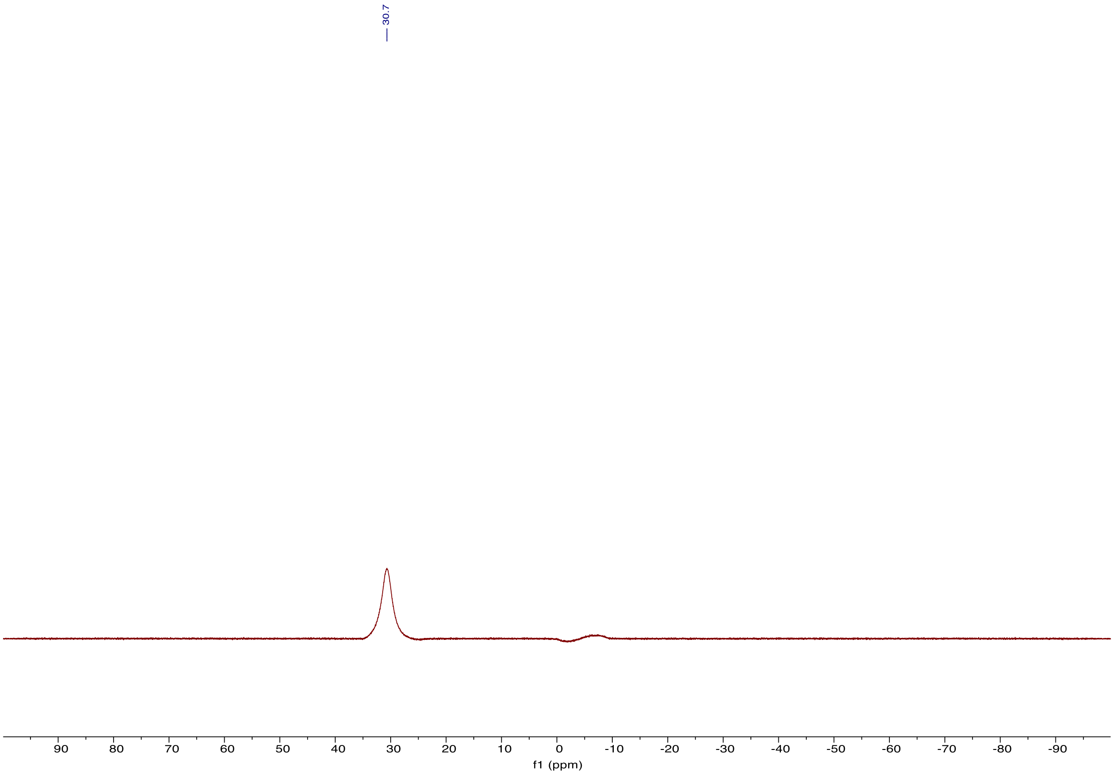

2-(3-(1-(4-methoxyphenyl)cyclobutyl)bicyclo[1.1.1]pentan-1-yl)-4,4,5,5-tetramethyl-1,3,2-dioxaborolane (3aj)

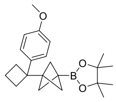

^{1}H NMR (600 MHz, $CDCl_3$)

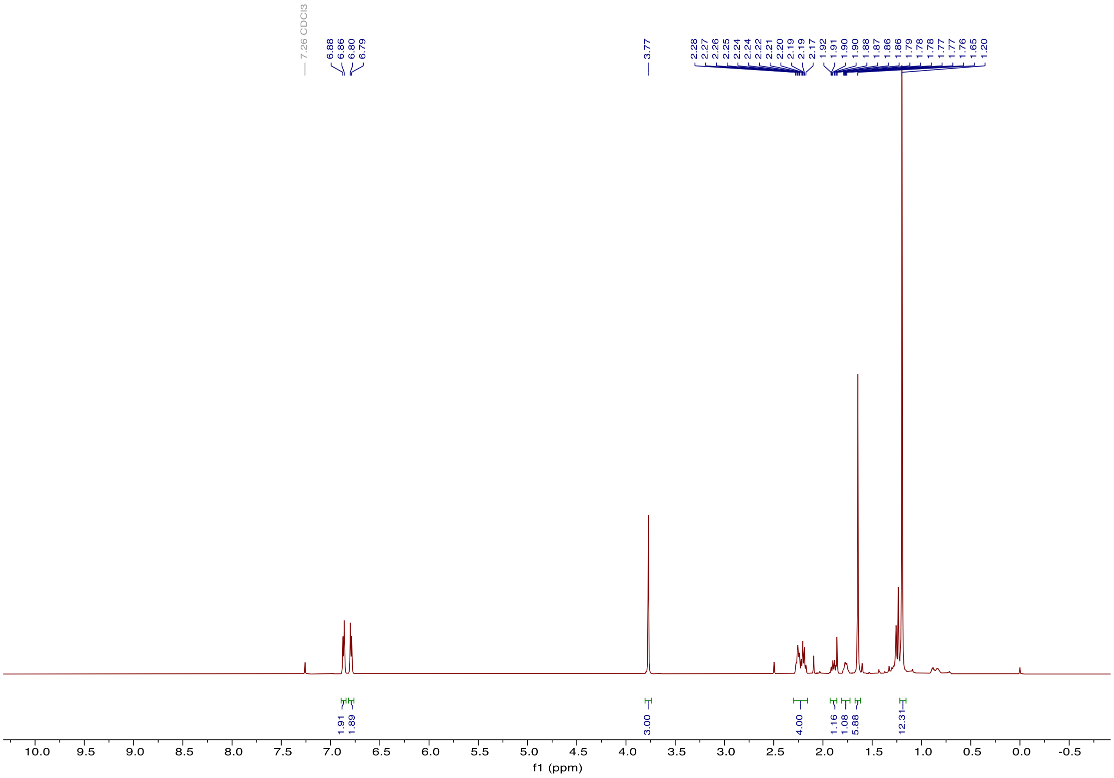

^{13}C NMR (151 MHz, CDCl$_3$)

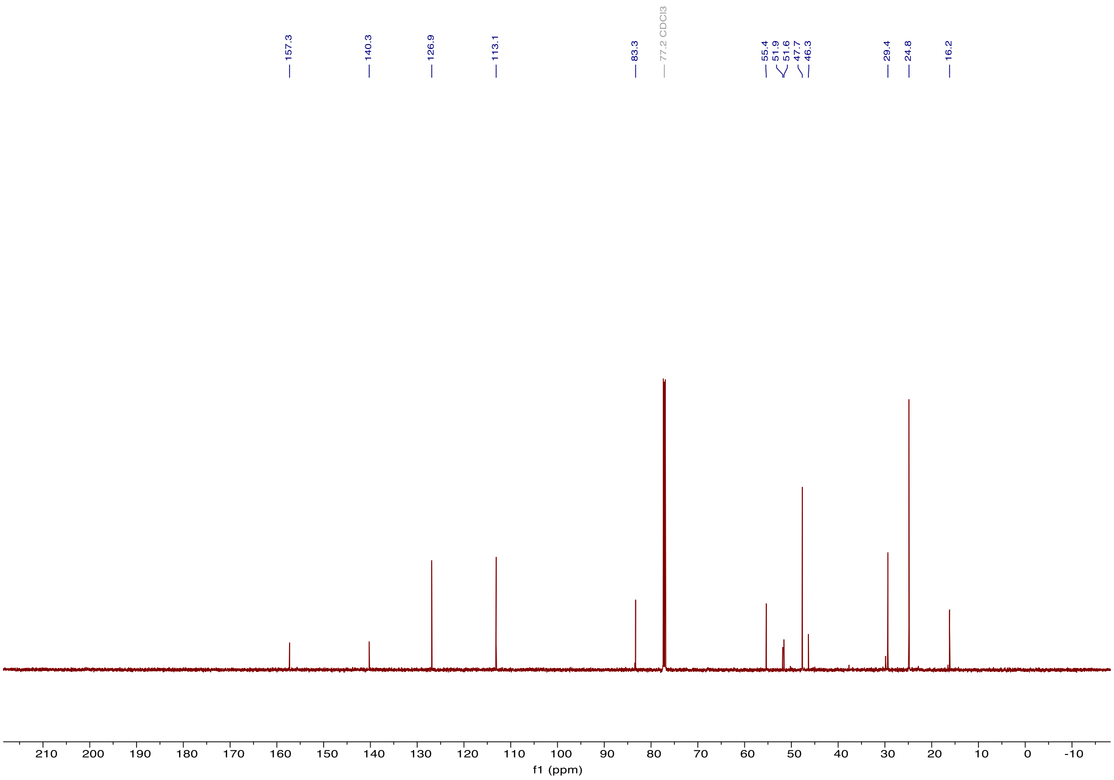

^{11}B NMR (192 MHz, CDCl$_3$)

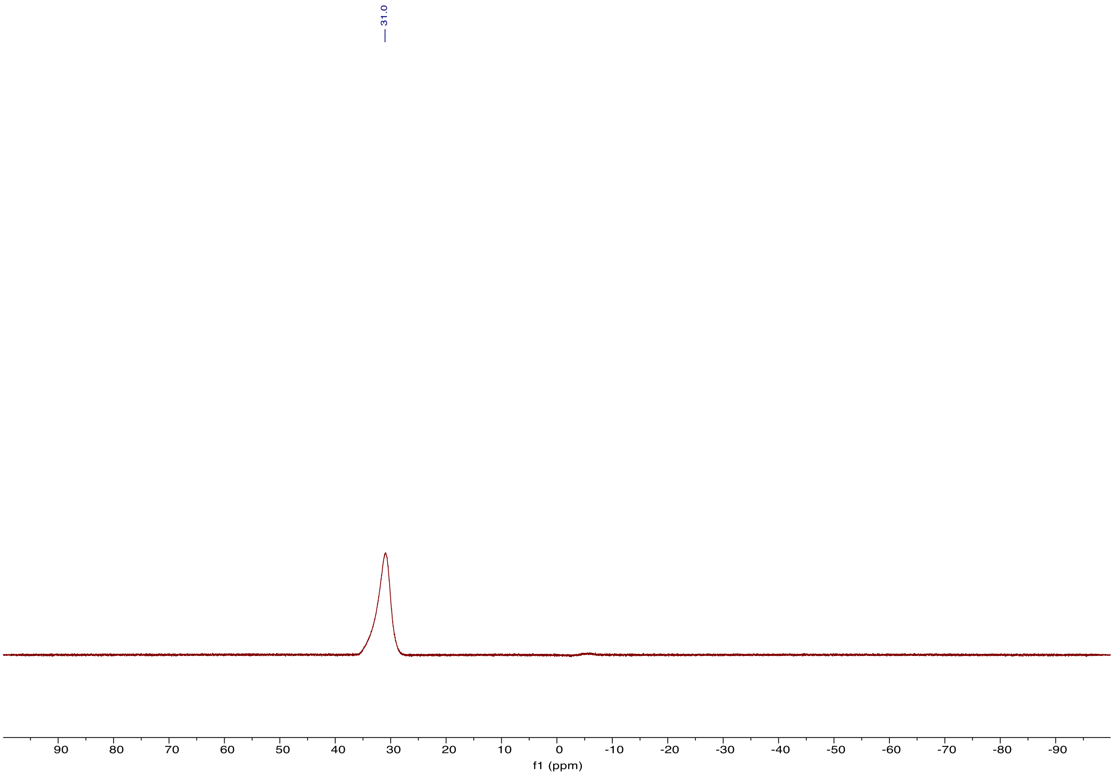

4,4,5,5-tetramethyl-2-(3-(1-phenylcyclopentyl)bicyclo[1.1.1]pentan-1-yl)-1,3,2-dioxaborolane (3ak)

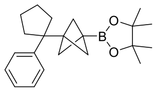

^{1}H NMR (600 MHz, $CDCl_3$)

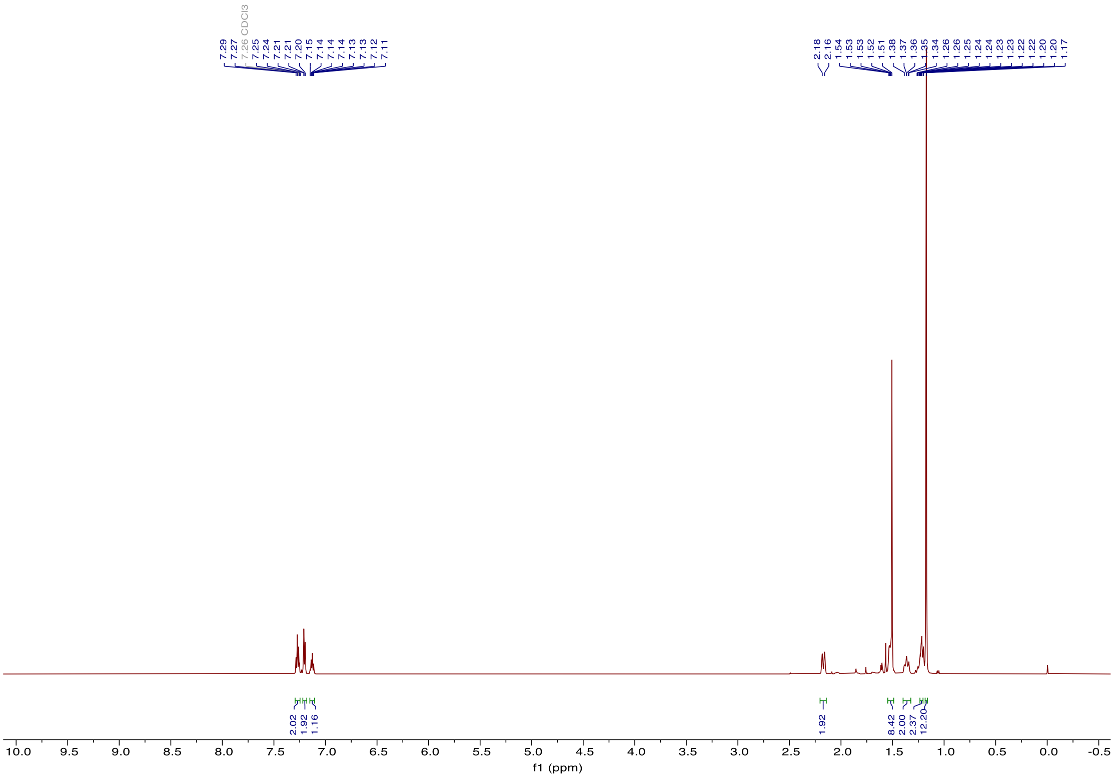

^{13}C NMR (151 MHz, $CDCl_3$)

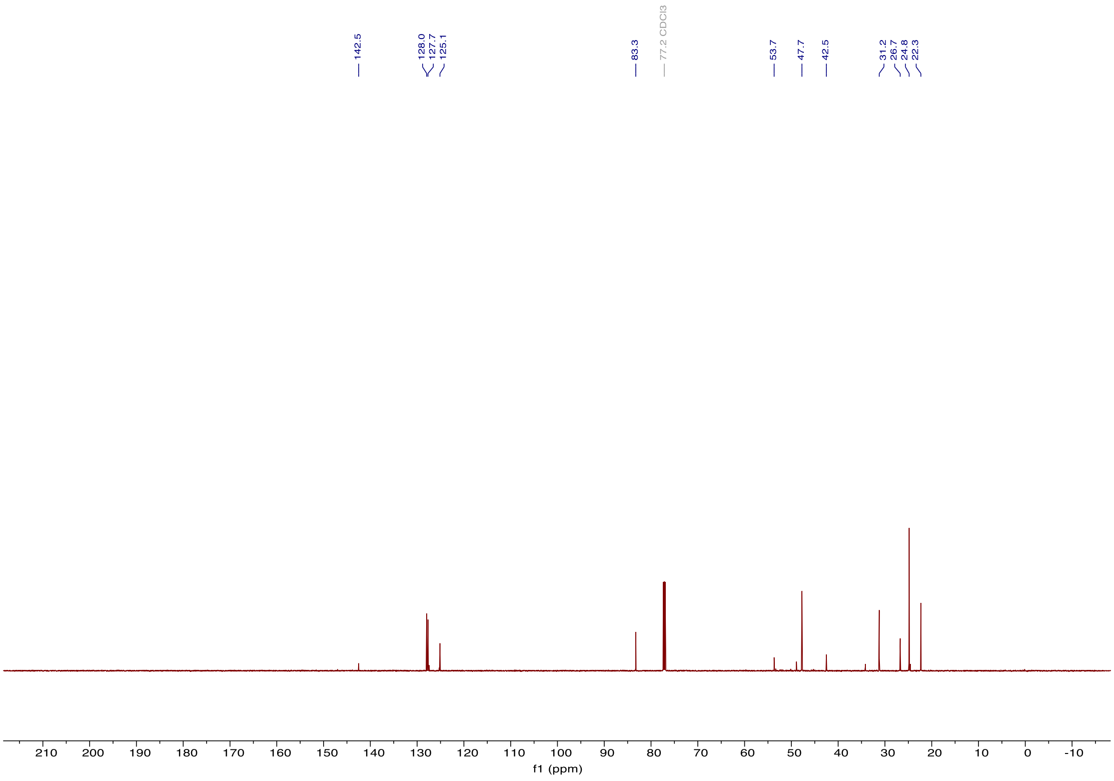

^{11}B NMR (192 MHz, $CDCl_3$)

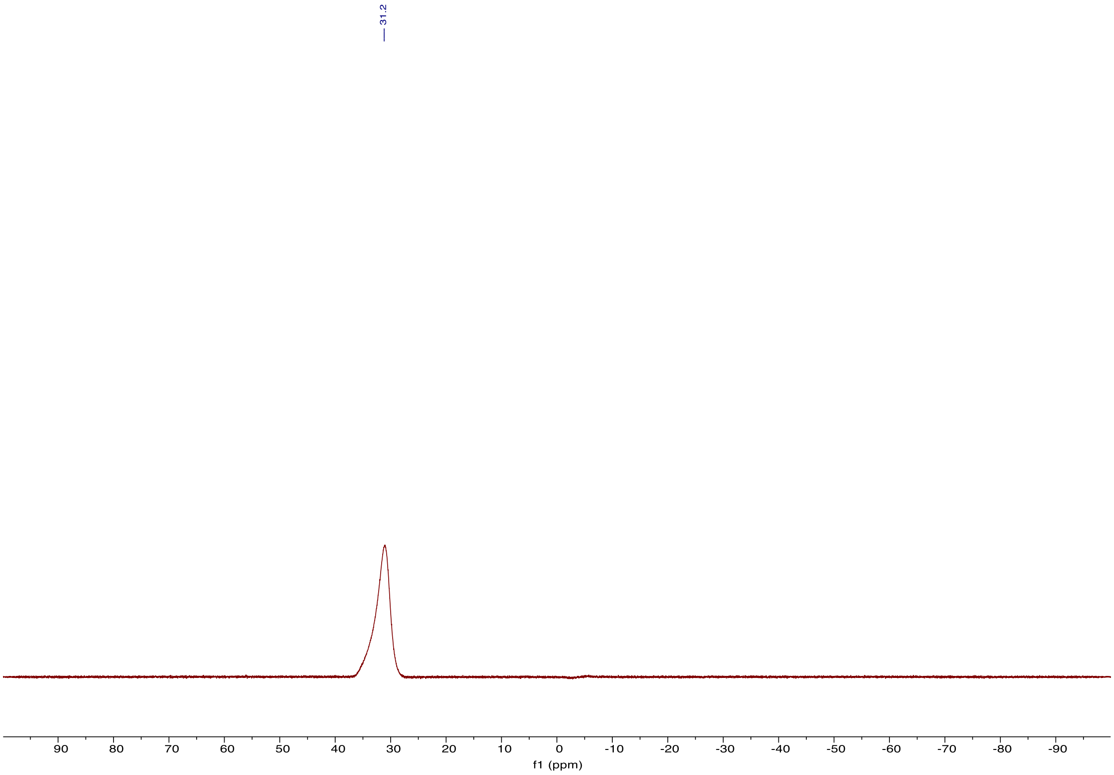

2-(3-(5-(2,5-dimethylphenoxy)-2-methylpentan-2-yl)bicyclo[1.1.1]pentan-1-yl)-4,4,5,5-tetramethyl-1,3,2-dioxaborolane (3al)

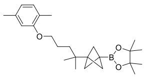

^{1}H NMR (600 MHz, $CDCl_3$)

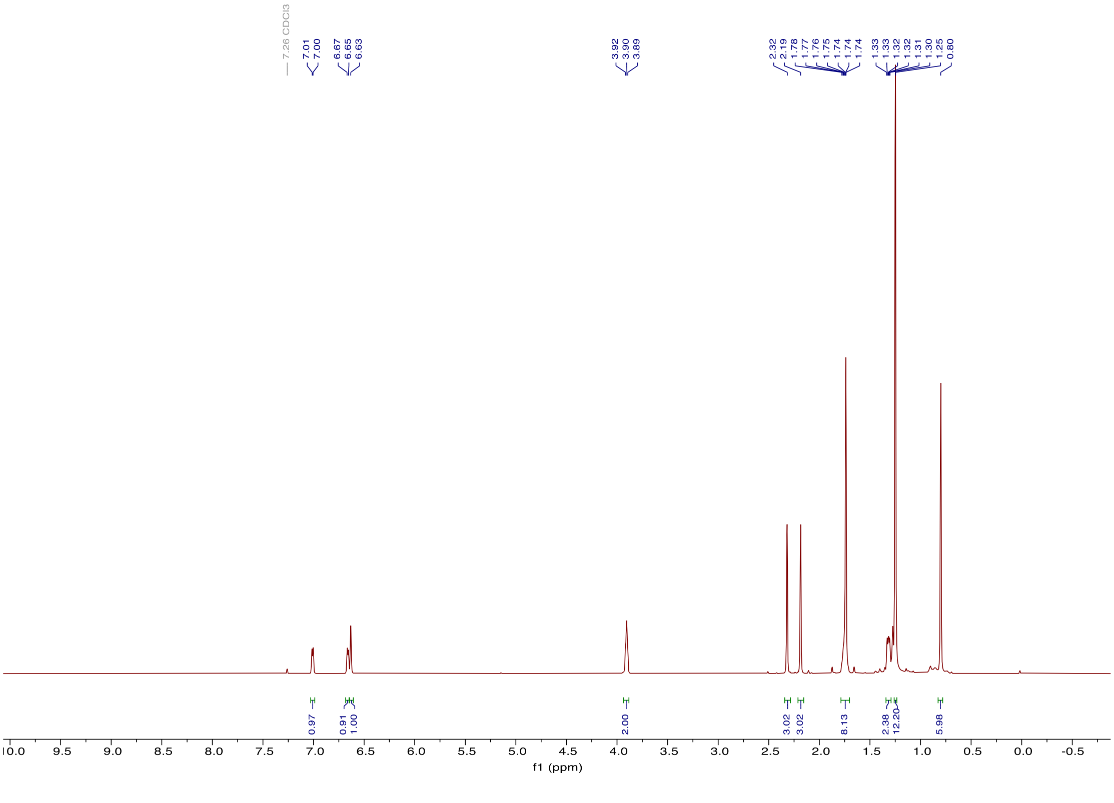

^{13}C NMR (151 MHz, $CDCl_3$)

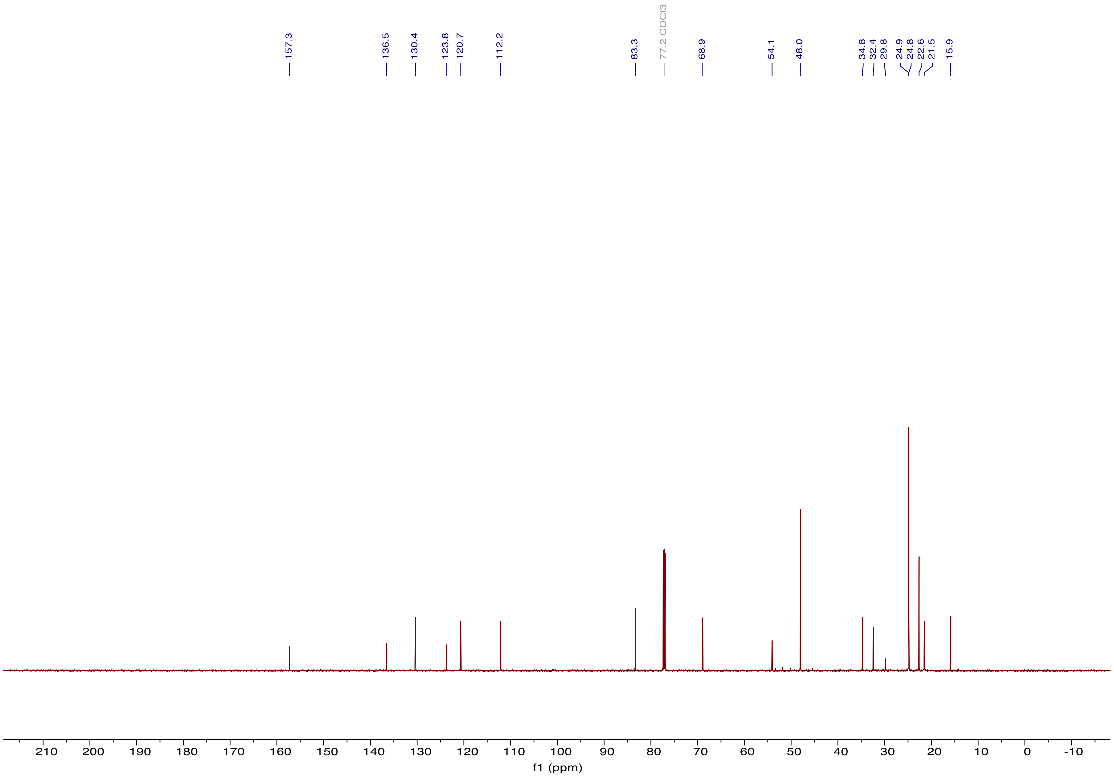

^{11}B NMR (192 MHz, $CDCl_3$)

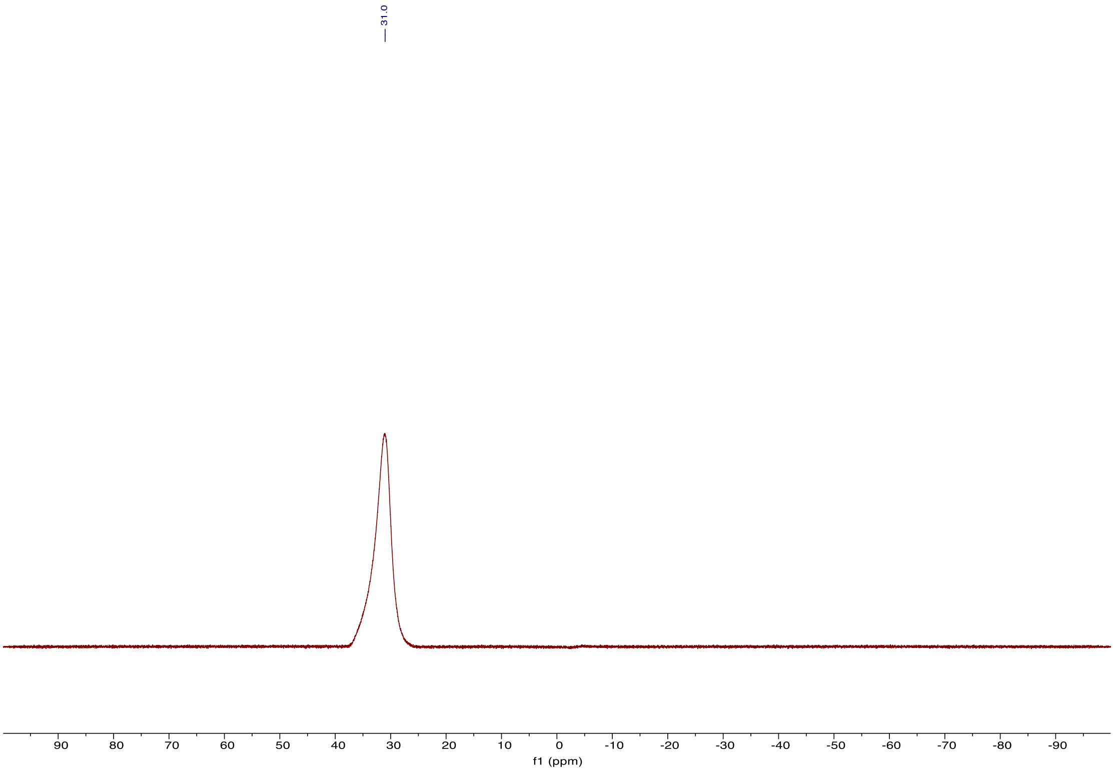

2-(3-((1*R*,4a*R*,4b*R*,10a*R*)-7-isopropyl-1,4a-dimethyl-1,2,3,4,4a,4b,5,6,10,10a-decahydrophenanthren-1-yl)bicyclo[1.1.1]pentan-1-yl)-4,4,5,5-tetramethyl-1,3,2-dioxaborolane (3am)

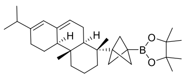

^{1}H NMR (600 MHz, $CDCl_3$)

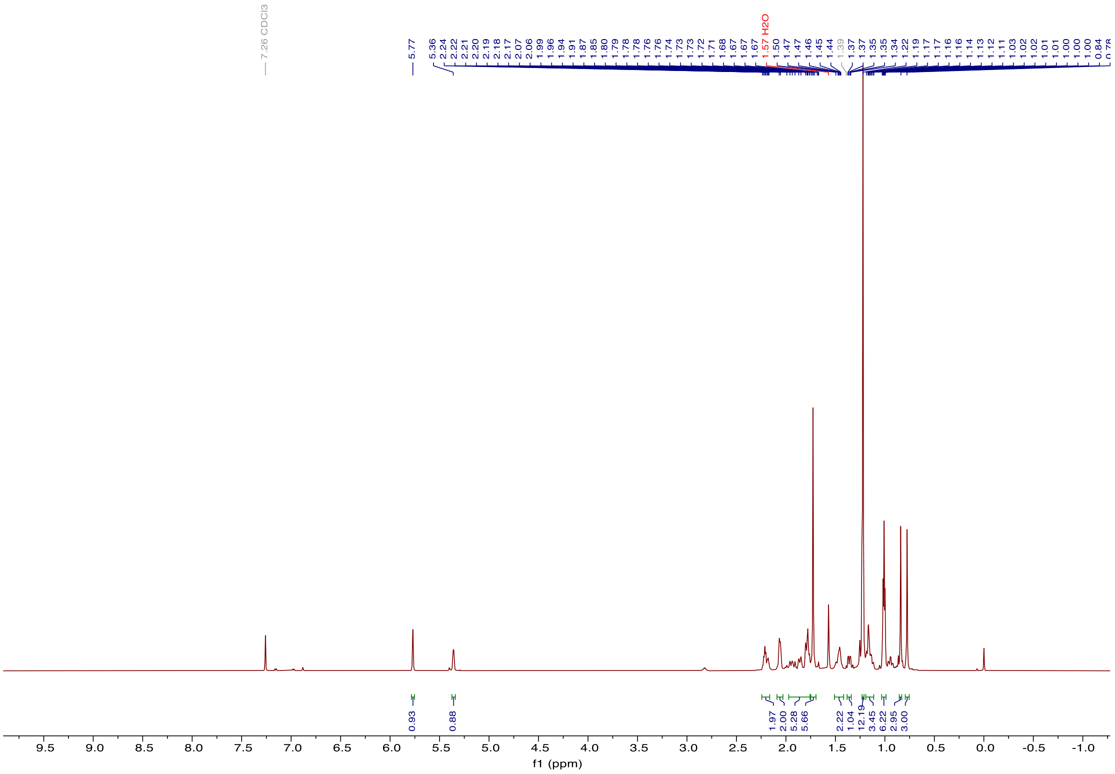

^{13}C NMR (151 MHz, $CDCl_3$)

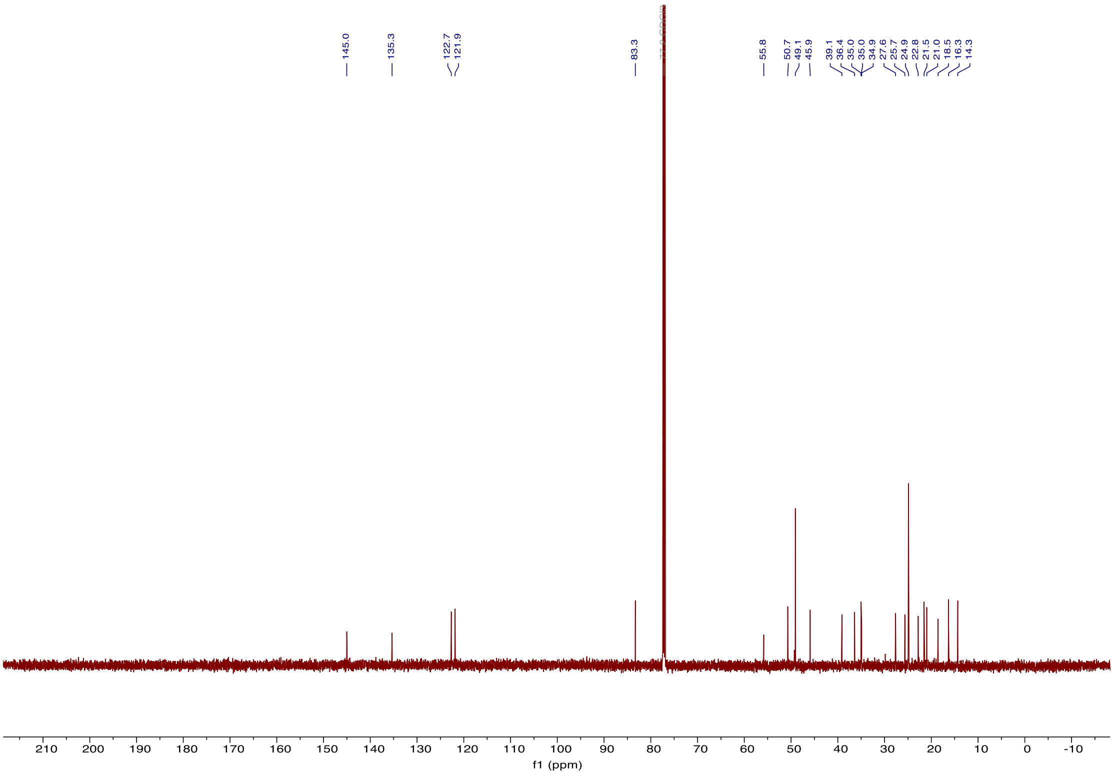

^{11}B NMR (192 MHz, CDCl$_3$)

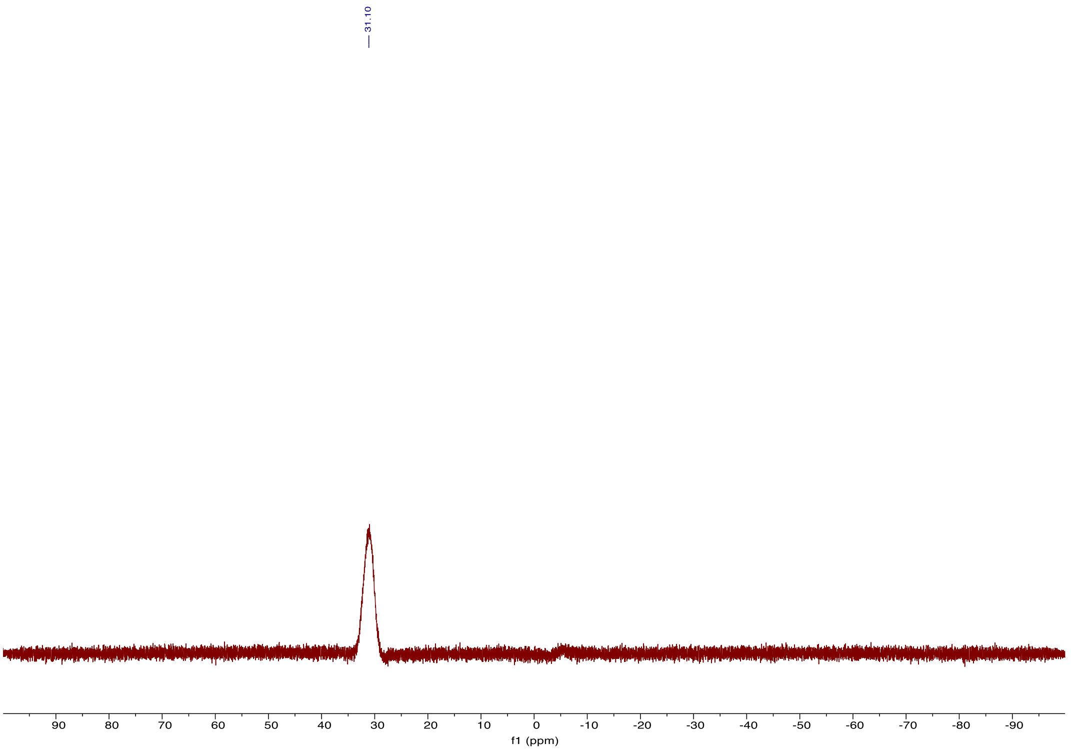

2-(3-((1*R*,4a*S*,10a*S*)-7-isopropyl-1,4a-dimethyl-1,2,3,4,4a,9,10,10a-octahydrophenanthren-1-yl)bicyclo[1.1.1]pentan-1-yl)-4,4,5,5-tetramethyl-1,3,2-dioxaborolane (3an)

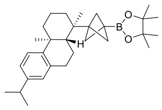

^{1}H NMR (600 MHz, CDCl$_3$)

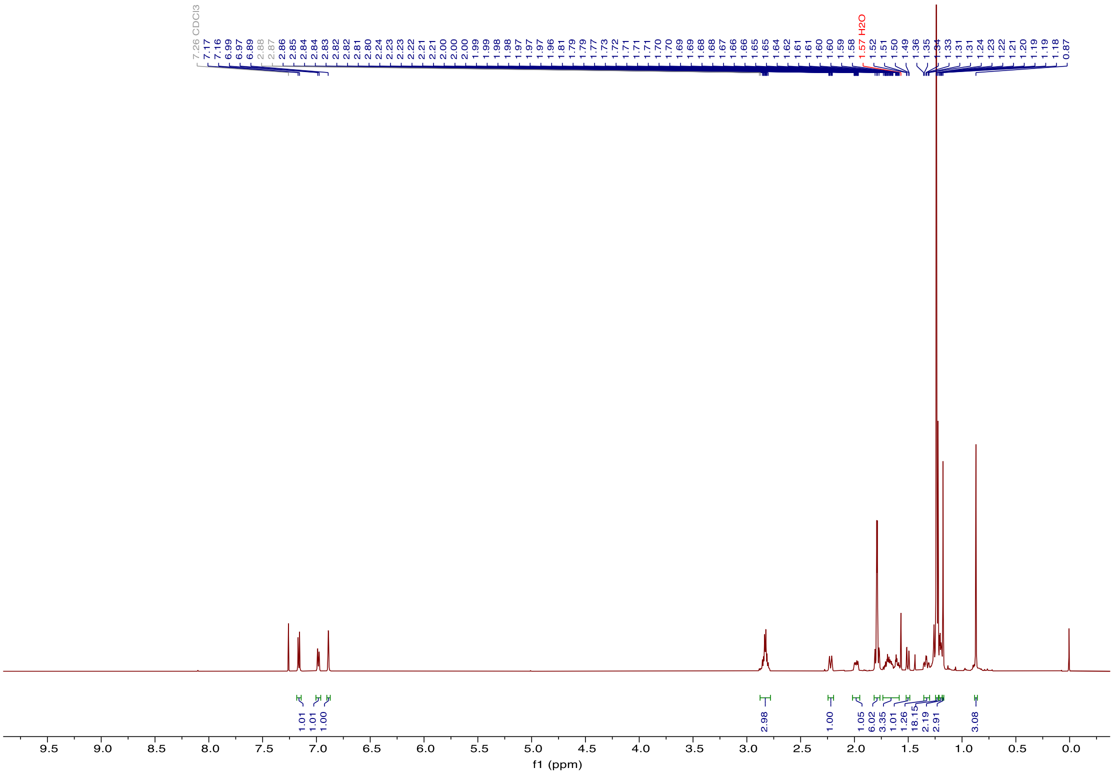

^{13}C NMR (151 MHz, $CDCl_3$)

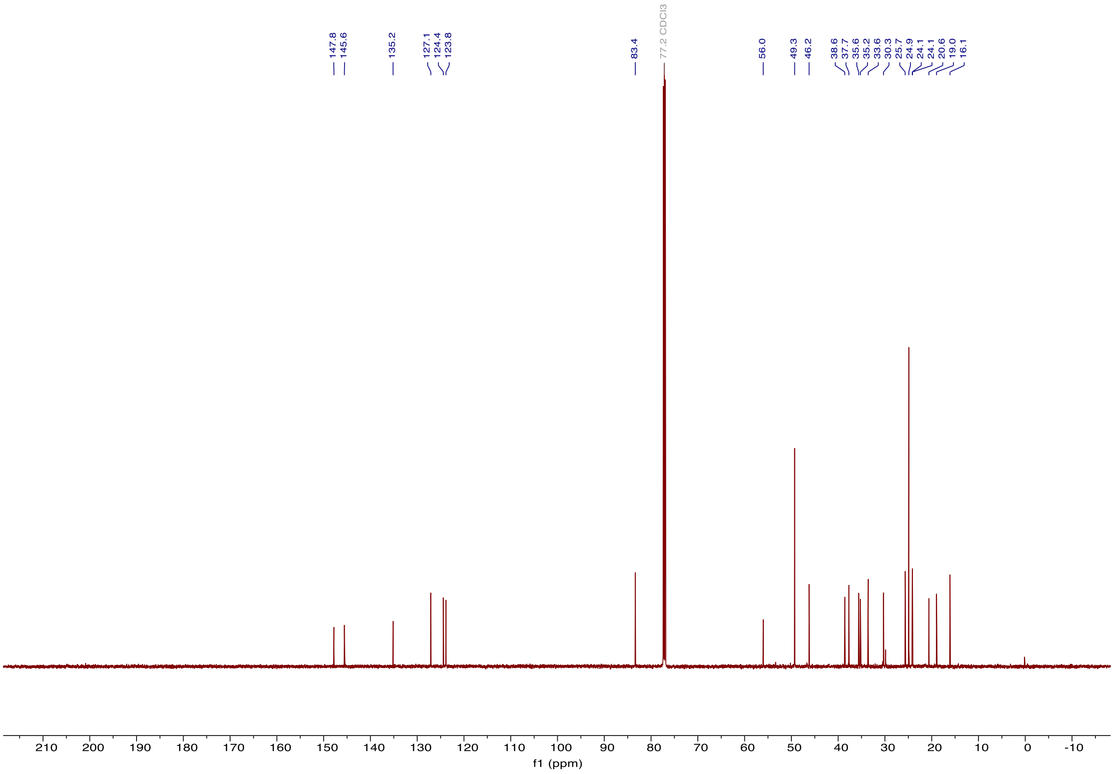

^{11}B NMR (192 MHz, $CDCl_3$)

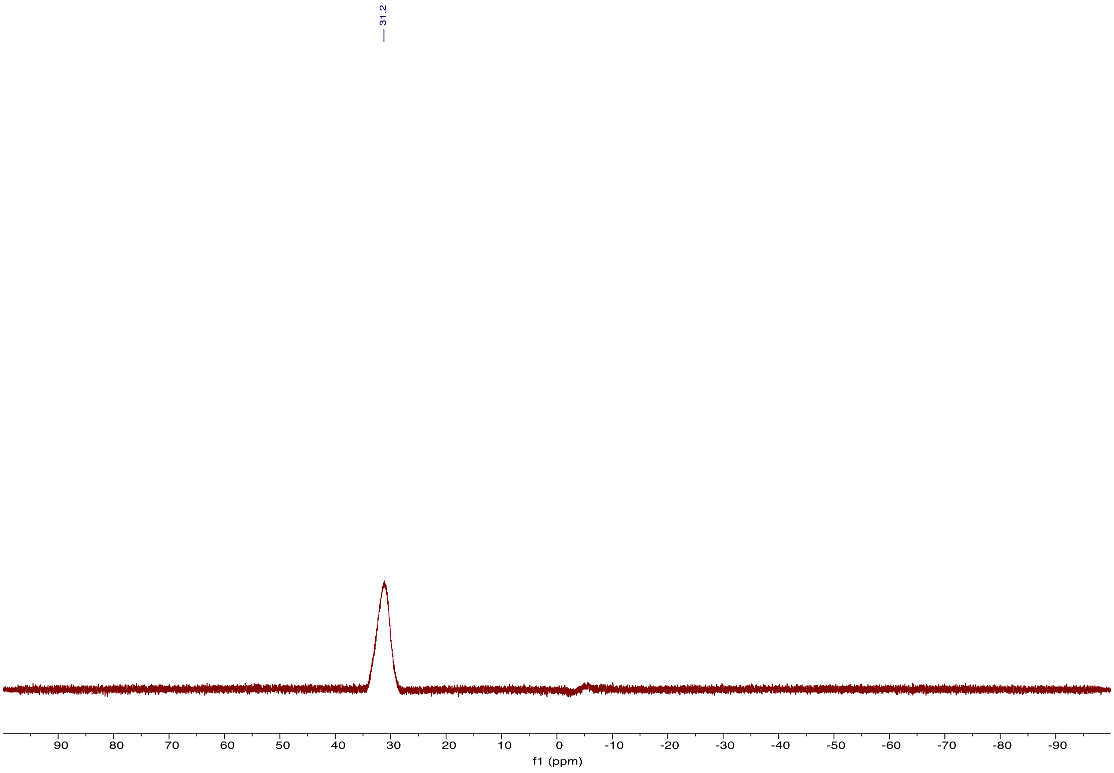

1-(3-(*tert*-butyl)bicyclo[1.1.1]pentan-1-yl)-*N*-methyl-*N*-(naphthalen-2-ylmethyl)methanamine (3ke)

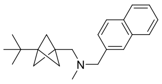

^{1}H NMR (600 MHz, CDCl3)

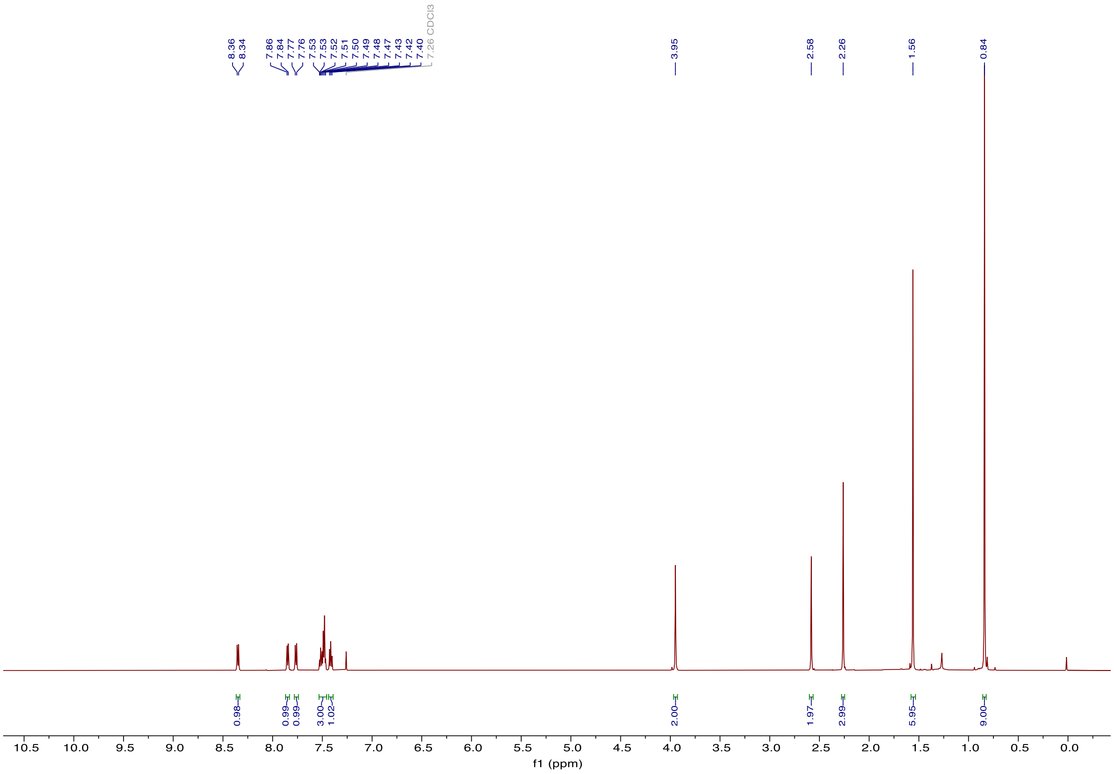

^{13}C NMR (151 MHz, CDCl3)

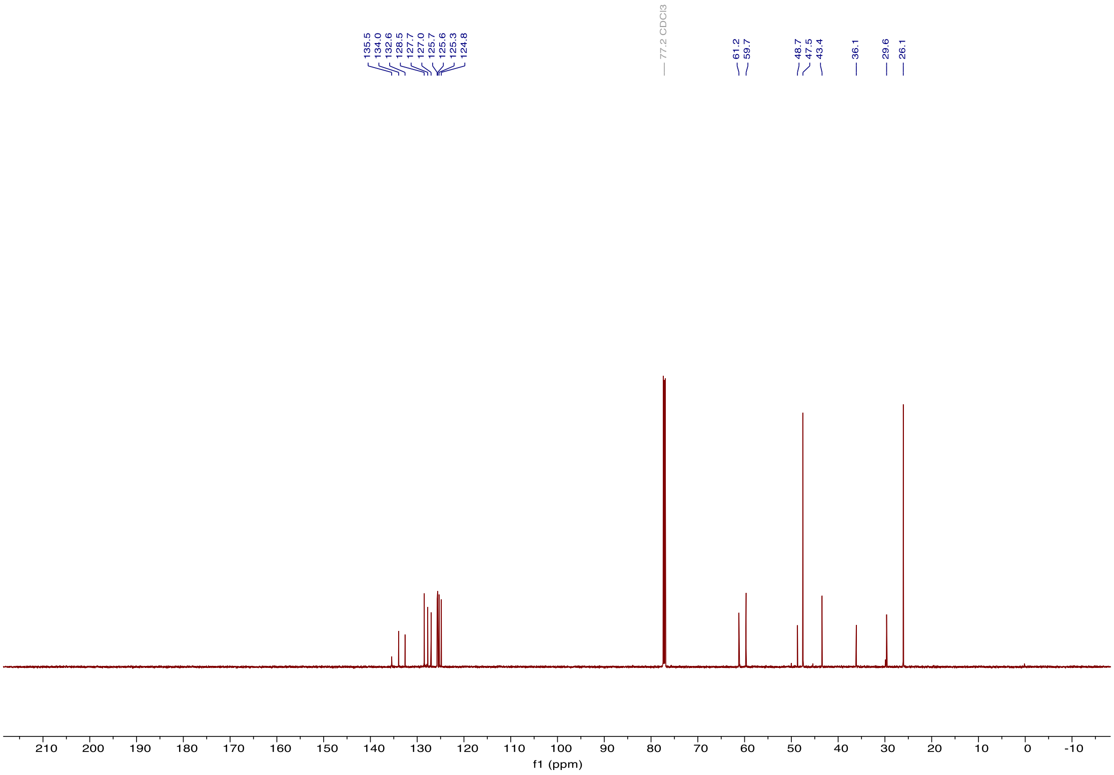

1-((3-(tert-butyl)bicyclo[1.1.1]pentan-1-yl)methyl)-4-((4-chlorophenyl)(phenyl)methyl)piperazine (3kg)

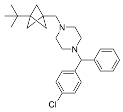

^{1}H NMR (600 MHz, $CDCl_3$)

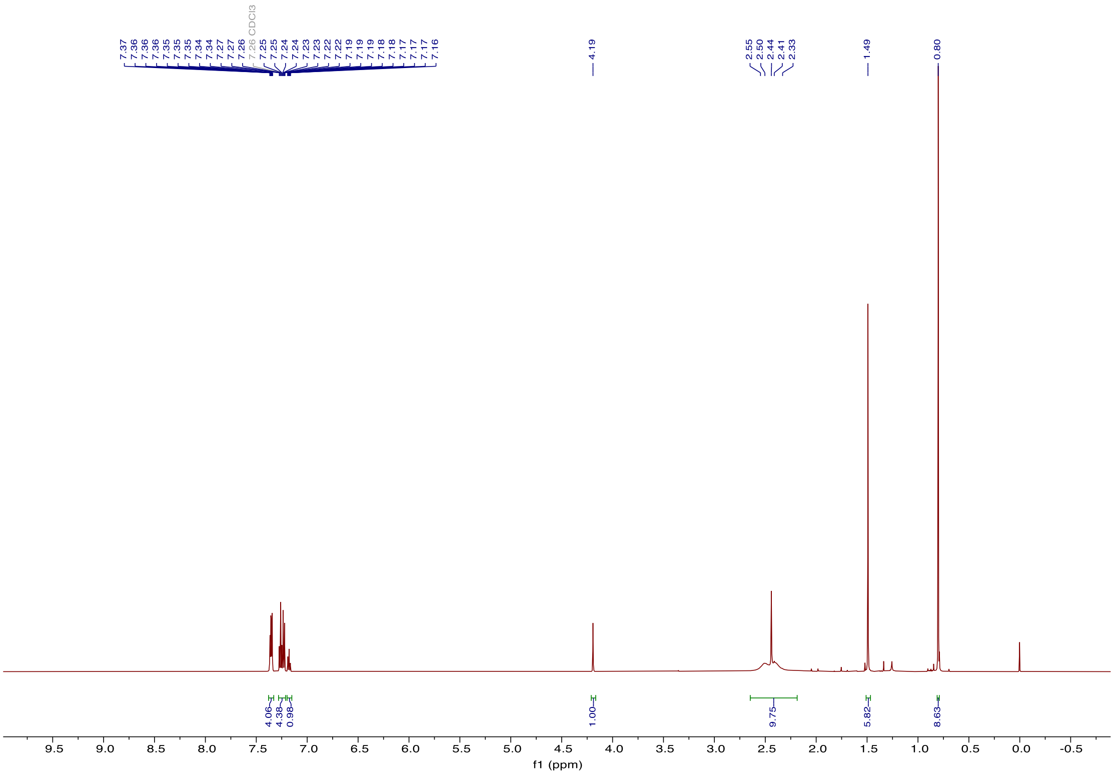

^{13}C NMR (151 MHz, $CDCl_3$)

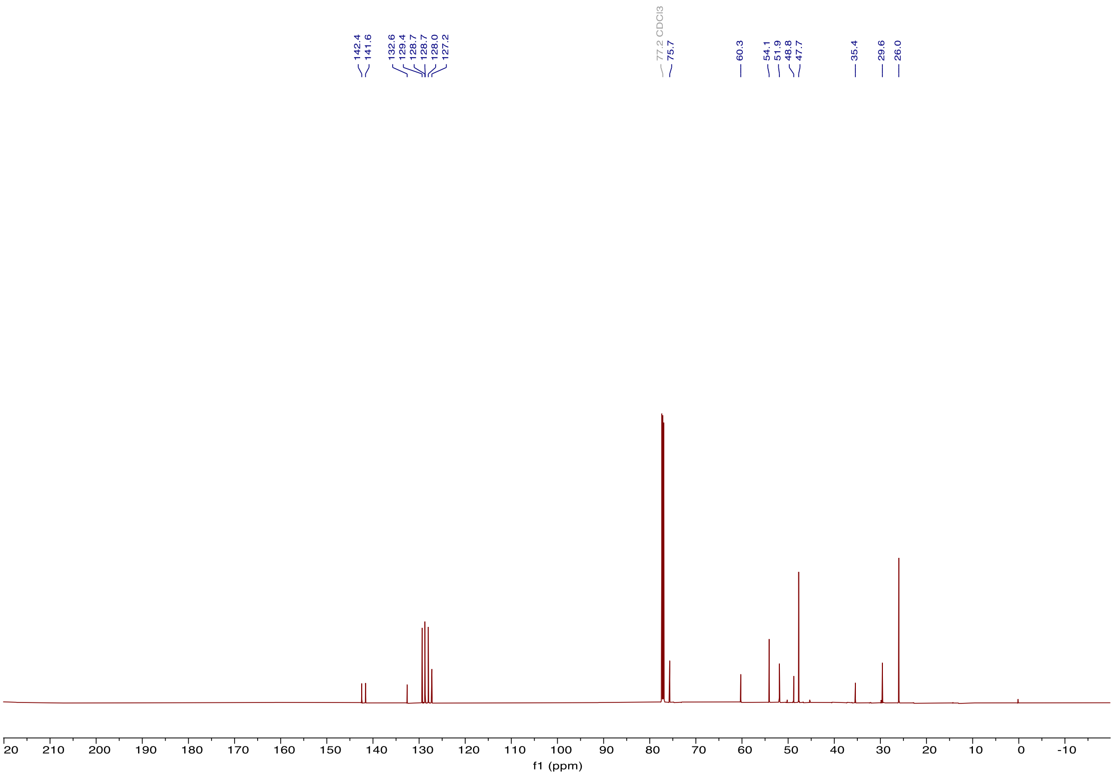

2-phenyl-2-(3-(4,4,5,5-tetramethyl-1,3,2-dioxaborolan-2-yl)bicyclo[1.1.1]pentan-1-yl)ethan-1-ol (3ao)

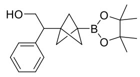

^{1}H NMR (600 MHz, $CDCl_3$)

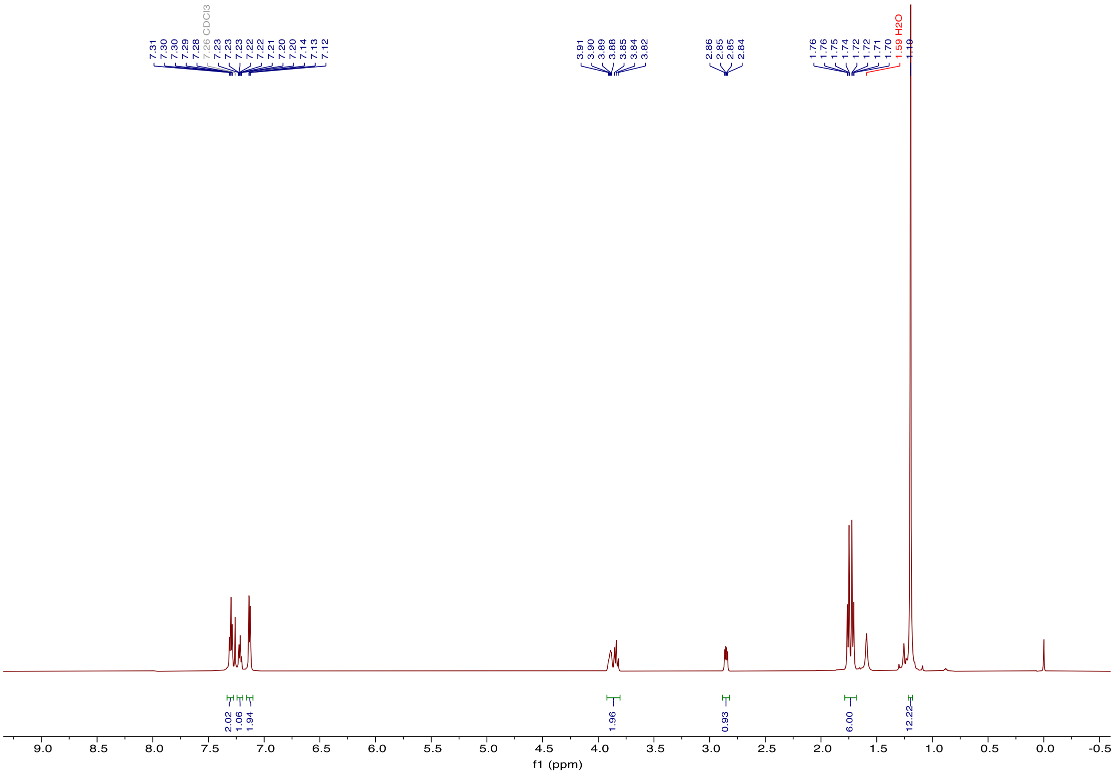

^{13}C NMR (151 MHz, $CDCl_3$)

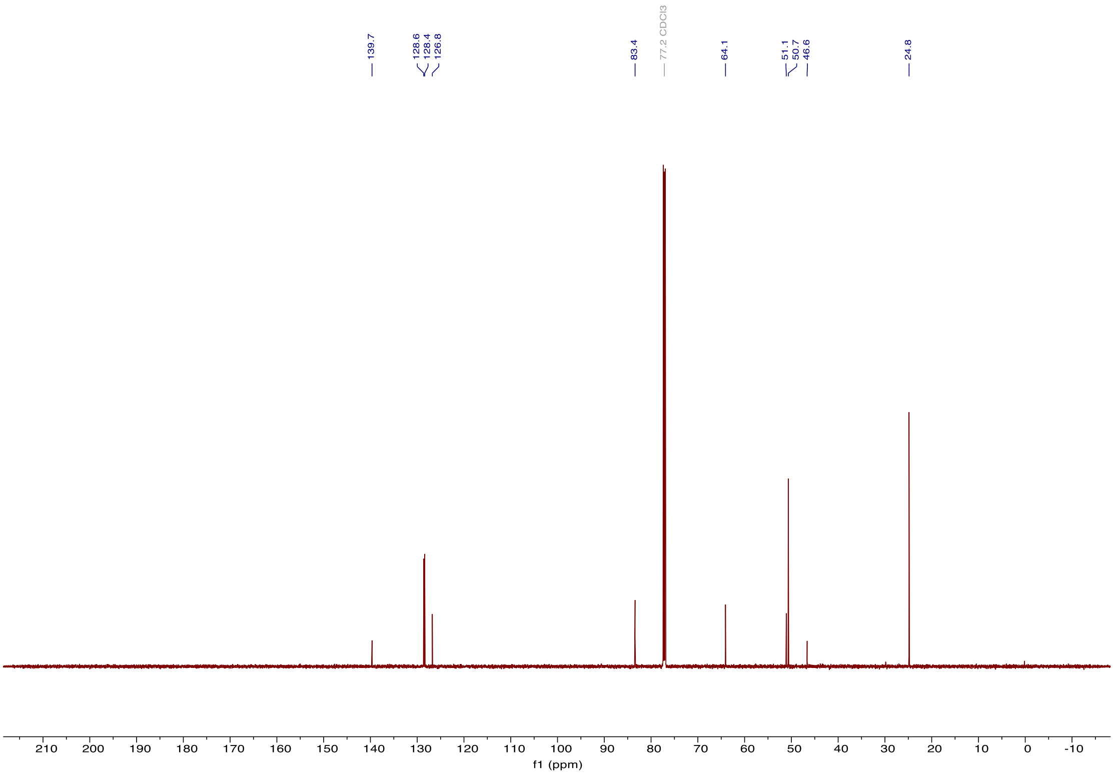

^{11}B NMR (192 MHz, CDCl$_3$)

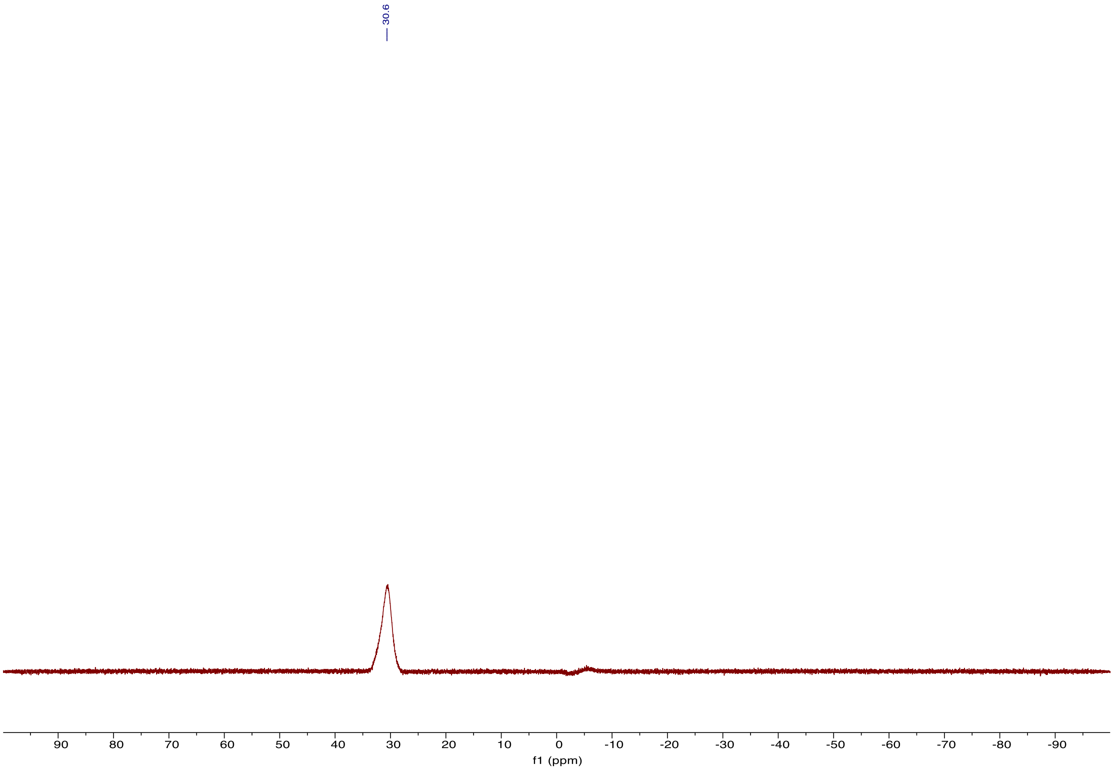

2-phenyl-2-(3-(4,4,5,5-tetramethyl-1,3,2-dioxaborolan-2-yl)bicyclo[1.1.1]pentan-1-yl)acetaldehyde (3ap)

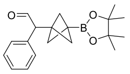

^{1}H NMR (600 MHz, CDCl$_3$)

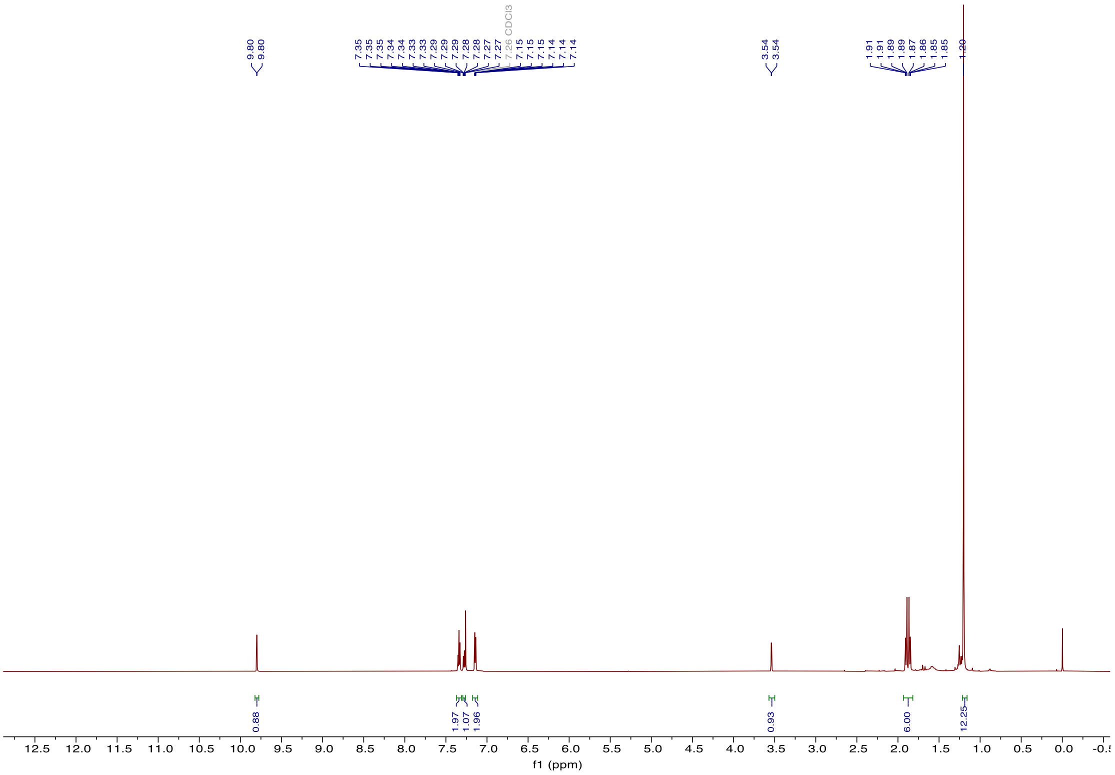

^{13}C NMR (151 MHz, $CDCl_3$)

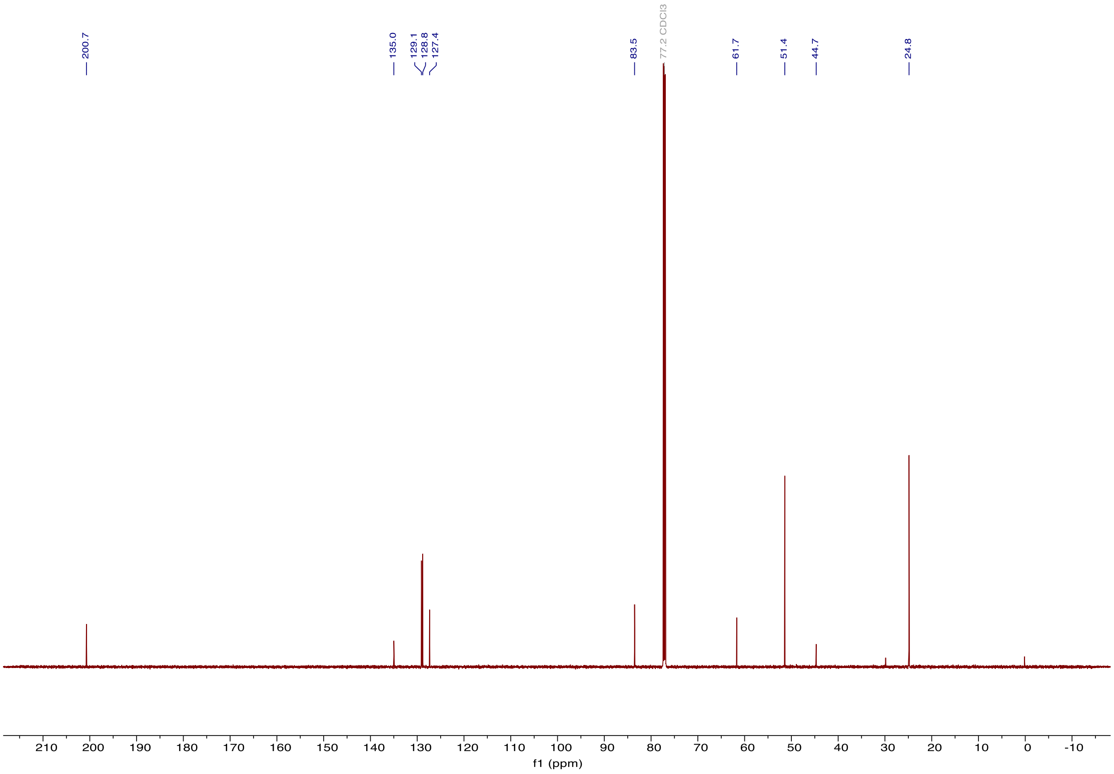

^{11}B NMR (192 MHz, $CDCl_3$)

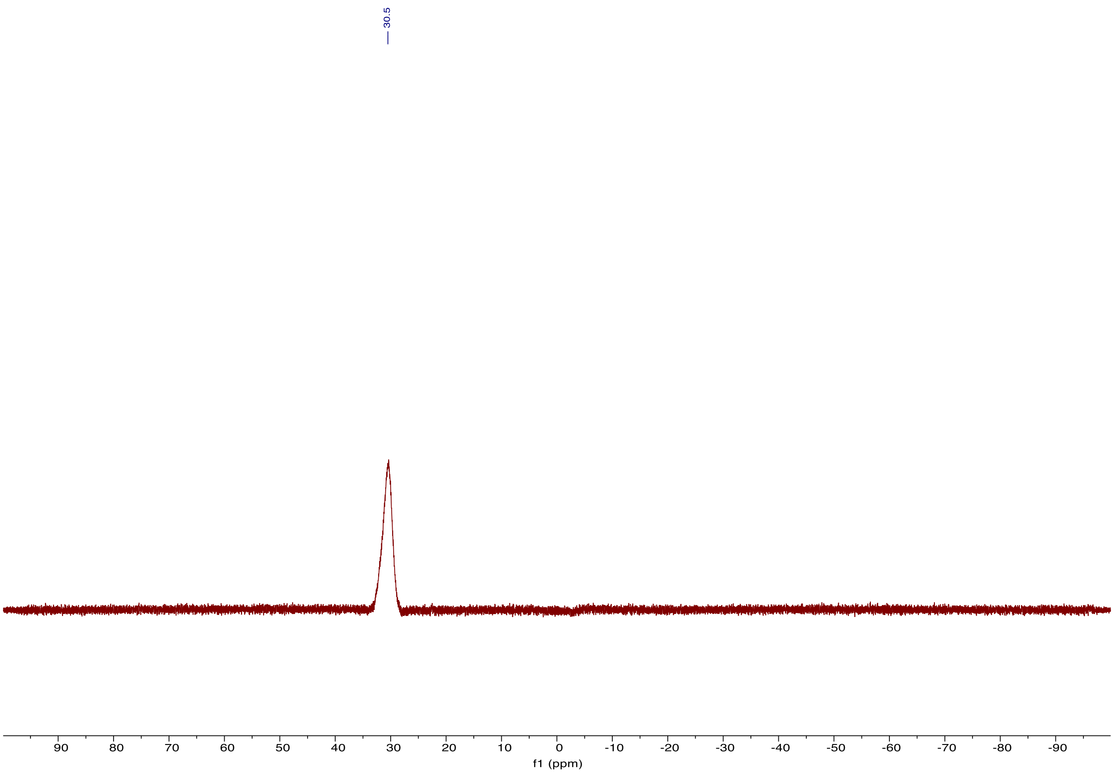

***tert*-butyl (*S*)-2-(3-(4,4,5,5-tetramethyl-1,3,2-dioxaborolan-2-yl)bicyclo[1.1.1]pentan-1-yl)pyrrolidine-1-carboxylate (3aq)**

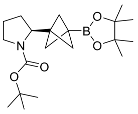

^{1}H NMR (600 MHz, $CDCl_3$)

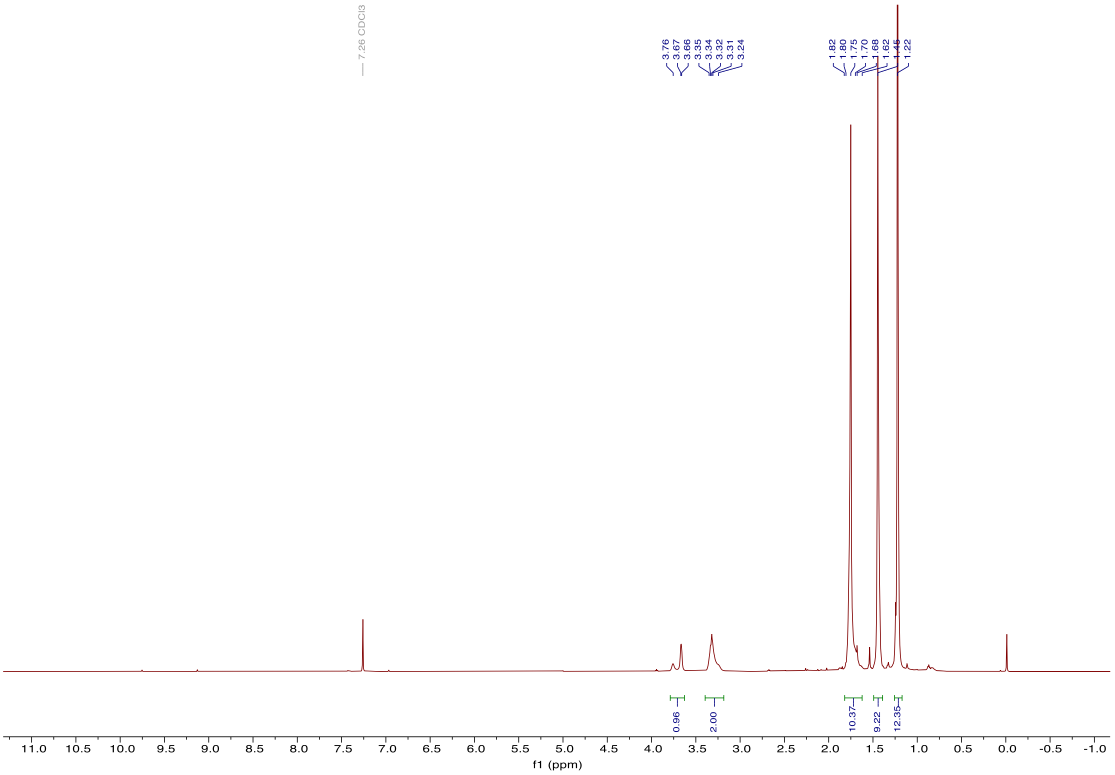

^{13}C NMR (151 MHz, $CDCl_3$)

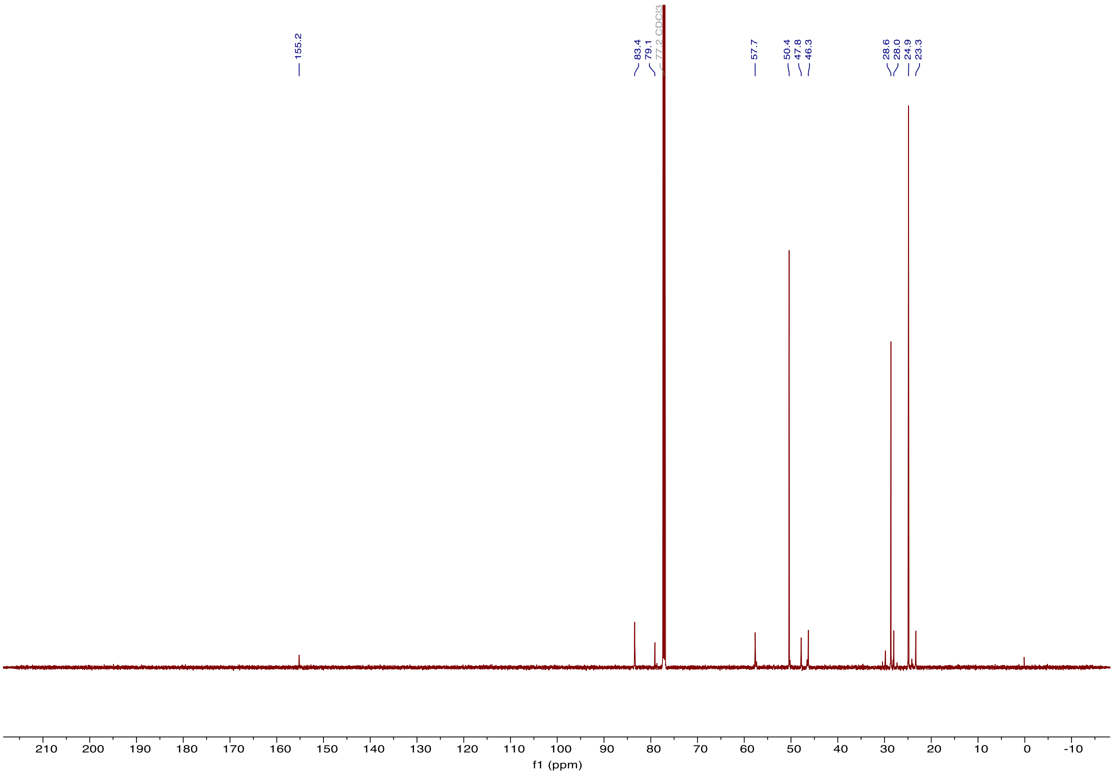

^{11}B NMR (192 MHz, $CDCl_3$)

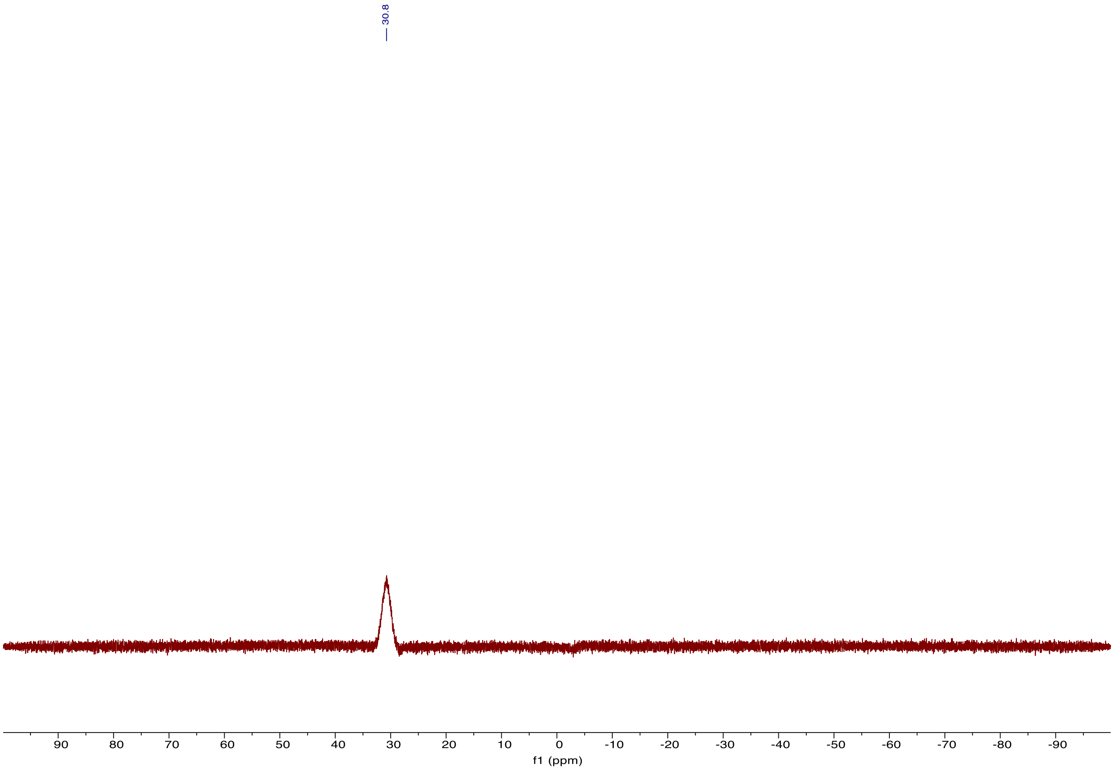

5. References.

1. Pickford, H. D.; Nugent, J.; Owen, B.; Mousseau, James. J.; Smith, R. C.; Anderson, E. A. Twofold Radical-Based Synthesis of *N*,*C*-Difunctionalized Bicyclo[1.1.1]pentanes. *J. Am. Chem. Soc.* **143**, 9729-9736 (2021).

2. Trammel, G. L.; Kannangara, P. B.; Vasko D.; Datsenko O.; Mykhailiuk, P.; Brown, M. K. Arylboration of Enecarbamates for the Synthesis of Borylated Saturated N-Heterocycles. *Angew. Chem. Int. Ed.* **61**, 12117–12124 (2022).

3. Sardini, S. R.; Lambright, A. L.; Trammel, G. L.; Omer, H. M.; Liu, P.; Brown M. K. Ni-Catalyzed Arylboration of Unactivated Alkenes: Scope and Mechanistic Studies. *J. Am. Chem. Soc.* **141**, 9391–9400 (2019).

4. Zhu, Z.; Chan, W.- C.; Gao, B.; Hu, G.-W.; Zhang, P.-Q.; Fu, Y.-Y., Ly, K. S.; Lin, Z.-Y.; Quan, Y.-J. Borenium-Catalyzed “Boron Walking” for Remote Site-Selective Hydroboration. *J. Am. Chem. Soc.* **147**, 880-888 (2025).
